# Pairing of Parental Noroviruses with Unequal Competitiveness Provides a Clear Advantage for Emergence of Progeny Recombinants

**DOI:** 10.1128/AEM.02015-20

**Published:** 2021-01-15

**Authors:** Eung Seo Koo, Yong Seok Jeong

**Affiliations:** a Molecular Virology Laboratory, Department of Biology, Kyung Hee University, Seoul, Republic of Korea; Centers for Disease Control and Prevention

**Keywords:** norovirus, recombination, novel recombinants, competitiveness, human population, sewage

## Abstract

Genetic recombination plays a pivotal role in the appearance of human norovirus recombinants that cause global epidemics. However, the factors responsible for the appearance of these recombinants remains largely unknown. In this study, we revealed a selective pressure that restricts parental combinations leading to the emergence of norovirus recombinants. To investigate traces of emerging novel recombinants and their parents in the human population, we isolated mass nucleotide sequence clones of human norovirus genogroups I and II in sewage-affected waters over a 4-year sampling period. Fourteen different phylogenetic combinations of recombinants and their parents were defined from the dozens of phylogenetic lineages circulating in the human population. To evaluate the probability of these combinations, parental lineages of each recombinant were categorized into two groups as HP (relatively higher-competitiveness parents) and LP (relatively lower-competitiveness parents), according to their relative detection frequency. Strong categorization of HP and LP was confirmed by tests with modified data and additional variables. An algorithm that was developed in this study to visualize the chance of mixed infection between parents revealed that HP lineages have a higher chance of mixed infection than LP lineages in the human population. Three parental pairing types in recombinants were defined: HP-HP, HP-LP, and LP-LP. Among these, most recombinants were identified as HP-LP, despite the prediction of dominant emergence of HP-HP-type recombinants. These results suggest that nature favors recombinants of human norovirus that originate from parental pairing of heterogeneous competitiveness.

**IMPORTANCE** Novel recombinants, generated from inter- and intraspecies recombination of norovirus lineages, often emerge and pose a threat to public health. However, the factors determining emergence of these particular recombinants from all possible combinations of parental lineages remain largely unknown. Therefore, current investigations on these recombinants are inevitably limited to postepidemic analyses, which merely identify genetic or phenotypic changes in the newly emerged recombinants compared to their parents. Here, we provide a new theoretical concept that emergence of novel recombinants could be explained by a combination of parental noroviruses thriving in the human population and those circulating at lower levels. This study could provide an additional and important rationale for the proactive environmental monitoring of potential future epidemics due to viral recombinants.

## Abstract (precis)

Novel recombinants, generated from inter- and intraspecies recombination of norovirus lineages, often emerge and pose a threat to public health. However, the factors determining emergence of these particular recombinants from all possible combinations of parental lineages remain largely unknown.

## INTRODUCTION

The human norovirus (family *Caliciviridae*) is considered to be the most common pathogenic agent causing nonbacterial acute gastroenteritis (1). A norovirus particle consists of 38-nm icosahedral nonenveloped capsids containing a VPg protein-linked positive-sense RNA genome of 7.3 to 7.5 kb (2). The human norovirus genome consists of three open reading frames (ORFs), ORF1, ORF2, and ORF3, which encode nonstructural proteins, major capsid (VP1), and minor capsid (VP2), respectively (2). ORF1 of the human norovirus genome is translated as a polyprotein, which is cleaved by viral proteases to produce six nonstructural proteins (p48, NTPase, p22, VPg, Pro, and Pol) (3). Subgenomic RNA, composed of both ORF2 and ORF3 generated from the genome, primarily contributes to the translation of both ORF2 and ORF3 (4). To date, phylogenetic classification of noroviruses has been conducted using either ORF1 or ORF2 alone or in combination (5). As full-genome sequencing of the norovirus genome is not conducted routinely, smaller regions throughout ORF1 and ORF2 (regions A, B, C, D, and E) have been targeted for phylogenetic classification (5, 6). Recently, noroviruses have been classified into 10 genogroups (GI to GX), of which six genogroups (GI, GII, GIV, GVII, GVIII, and GIX) infect humans (5, 7). These six genogroups have been further classified into genotypes, based on both ORF1 (14 GI P types, 37 GII P types, and 1 P type each for GIV and GVII) and ORF2 (9 GI types, 27 GII types, 2 GIV types, and 1 type each for GVII, GVIII, and GIX) (7).

For decades, GII variants possessing a genotype 4 (GII.4) ORF2 have been the most common variants detected in both clinical samples and environmental water sources (8, 9). Owing to the high evolutionary rate and rapid changing of surface antigenicity of the major capsid, variants associated with GII.4 ORF2 were considered to possess high epidemic potential (10, 11). However, increased norovirus detections have also been reported in association with different ORF1 genotypes (12, 13), which, in turn, suggests that any studies related to the emergence of novel norovirus variants must include ORF1, while taking into account the impact of the recombination of different genotypic sequences.

Recombination breakpoints of human norovirus occur at an “overlapped region of ORF1 and ORF2” and other nearby sites (<0.5 kb in general) from the overlapped region (14). However, the mechanisms underlying recombination at most sites remain unclear (14). A suggested recombination mechanism at an overlapped region between ORF1 and ORF2 revealed that the RNA-dependent RNA polymerase (RdRp) protein could dissociate from the full-length negative-strand template at the overlapped region and continue positive-strand synthesis at the subgenomic ORF2-ORF3 negative-strand template originating from a coinfected variant (15).

Noroviruses can be carried asymptomatically in human populations (16). At present, hospitalized cases of symptomatic human norovirus infections have been the source of most studies, except for a few planned experiments that investigated asymptomatic infections (17, 18). Such one-sided clinical monitoring is concerning, as it indicates that data from hospitalized cases may rarely reveal the preepidemic state of novel norovirus recombinants emerging in the human population.

Environmental waters contaminated by domestic sewage contain genetic information regarding norovirus infections in human populations affecting sewershed, combining both symptomatic and asymptomatic infections (8, 19). A variety of genetic variants rarely observed in clinical samples have been continuously detected in these waters (20), and even preepidemic states of the GII.17 variant have often been previously isolated from water samples (21). Studies of these waters have shown consecutive annual replacement (which is equivalent to peak changes of detection frequencies) of major epidemic variants (GII.4[P31], GII.17[P17], and GII.4[P16]) (19, 20, 22). The identical replacement of these variants in environmental waters with concurrent clinical cases (23, 24) revealed that detection frequencies of genetic variants in these waters represent the relative epidemiological competitiveness of each variant in neighboring human populations.

Mixed infection in an individual is an essential requirement for the occurrence of genetic recombination in human noroviruses (14). As described above, both GII.4 and GII.17 variants have caused major epidemics for many years. This epidemiological history indicates that major epidemic variants have likely caused more significant human infections by any scale of measure than other genotypic variants. This suggests that the major epidemic variants have worked together to cause most mixed infections in the human population. However, a variety of combinations of ORF1/2 genotypic recombinants have occurred throughout the world, and their parental sequences were rarely associated with major epidemic variants (14), suggesting that parental variants not associated with public illness have overcome their rare chance of mixed infection with other variant species.

To gain insight into the pairing of different parental variants exhibiting various prevalences in human populations, we isolated nucleotide sequences of both genotypic recombinants of the human norovirus as well as their parental variants from environmental water sources, including sewage in South Korea over 4 years (from March 2014 to April 2018). We then compared both detection frequencies and related measures of parental variants of each recombinant and investigated relationships between the relative epidemiological competitiveness of parents and the pairing modes for recombination.

## RESULTS

### Dozens of genotypic/subgenotypic noroviruses in the human population.

**FIG 1:**
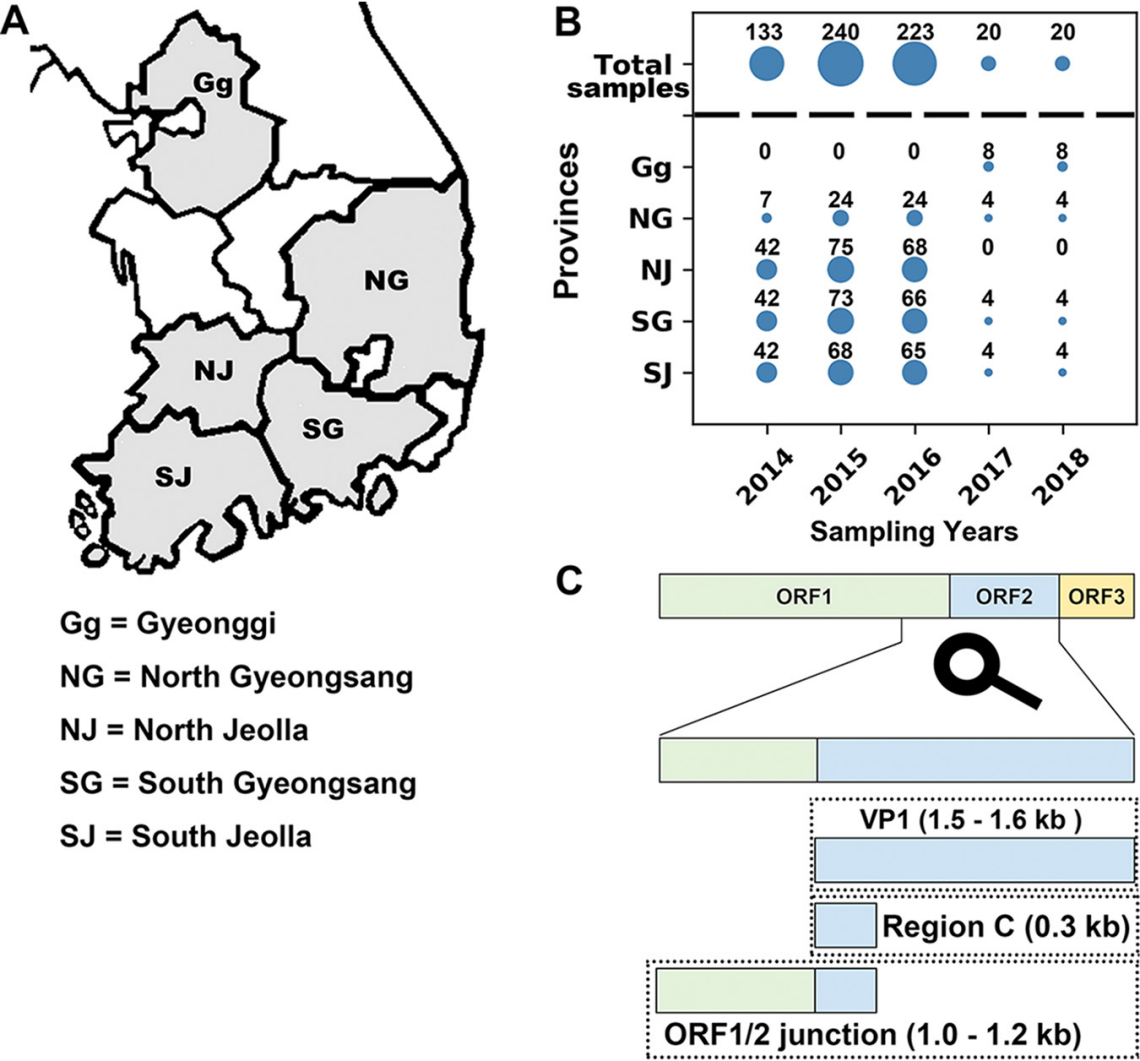
Sampling provinces and target regions associated with the norovirus genome for conventional RT-PCR. (A) Water samples were harvested from five provinces in the Republic of Korea. The five sampling provinces are depicted in gray on the map. Abbreviated names of the provinces are located within the gray sections. The full names of the provinces are shown below the map. (B) The number of water samples harvested each year from the provinces is depicted by both the relative sizes of the circles and the numerals above the circles. (C) Coverage of three amplicon types (region C, VP1, and ORF1/2 junction) of the human norovirus genome is shown.

From March 2014 to April 2018, water samples (stream water, raw sewage, and treated sewage effluent) affected by domestic sewage in five provinces of South Korea were collected (Fig. 1A and B). A total of 1,781 nucleotide sequences of norovirus genogroups I and II (GI and GII) were isolated and categorized according to amplicon types as follows: a full-length (or a nearly full-length) ORF2 termed VP1 (1.6 kb, GI; 1.5 kb, GII); region C (0.3 kb, both GI and GII) located at the 5′ end of VP1 and the ORF1/ORF2 junction (partial ORF1 plus region C: 1.2 kb, GI; 1.0 kb, GII) (Fig. 1C).

**FIG 2:**
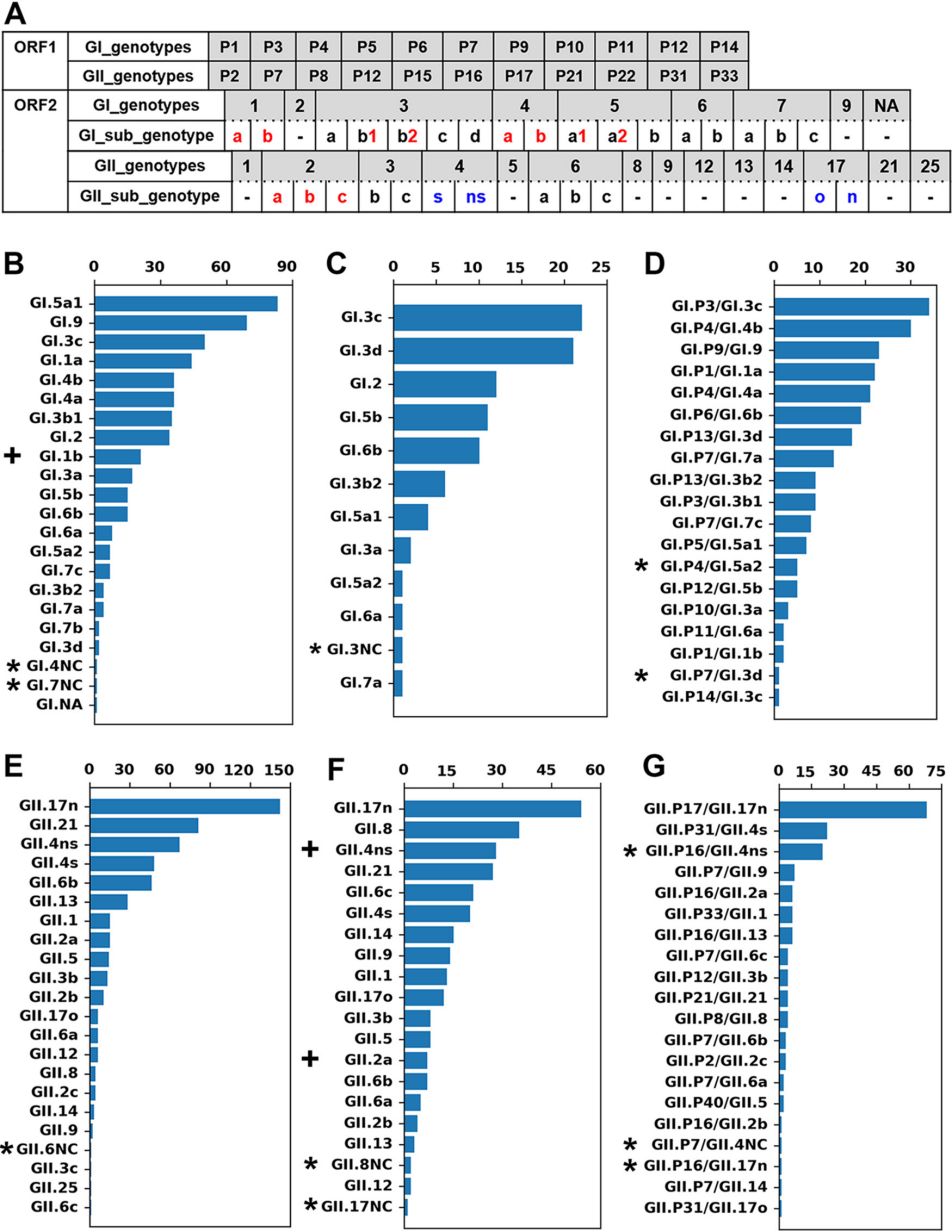
Total number of isolated nucleotide sequences of norovirus after subgenotyping of common region C. (A) Genotypes of both ORF1 and ORF2 of isolated nucleotide sequences are shown in the gray boxes (NA, not assigned). Subgenotypes of ORF2 region C are shown in white boxes below the name of each name genotype; the symbol “-” indicates no positive result from subgenotyping. Subgenotypes suggested previously are shown as black numerals or black letters in the white boxes. Newly defined subgenotype lineages in this study are shown as red numerals or red letters. For analytical feasibility, previously used subgenotypic names without abbreviated names for both GII.4 and GII.17 are newly abbreviated in blue letters. (B to G) Total numbers of lineages obtained from isolated sequences of region C amplicons (GI, panel B; GII, panel E), VP1 amplicons (GI, panel C; GII, panel F), and ORF1/2 junction amplicons (GI, panel D; GII, panel G) are shown as horizontal bar plots. In each bar plot, the *y* axis indicates the name of the lineages, while the *x* axis indicates the total number of sequences of the lineages isolated during the study period. Genetic recombinants identified via subsequent analyses are marked by asterisks or plus symbols. An asterisk indicates the recombinant sequence itself; the plus symbol indicates a sequence group consisting of the recombinant and its parent.

Genotypes of the isolated norovirus sequences were identified using the web-based norovirus genotyping tool (Noronet) (25) (see Fig. S1 in the supplemental material). The commonly included region C of the three amplicon types was additionally analyzed to define its subgenotypes. By this process, subsequent analyses were carried out on the assumption that the three amplicon types, including identical subgenotypic region C sequences, had originated from the genome of a single subgenotypic variant (22). In this study, we used the term “lineage” to specify a phylogenetic group. We divided the 23 genotypes of region C that emerged during the study period into 41 lineages (GI, 20 lineages; GII, 21 lineages) based on previously suggested ORF2 subgenotypes (5) (Fig. 2; Fig. S2 to S4). Two maximum clade credibility (MCC) trees (Fig. S5) were constructed to confirm the large subgenotypic clusters of both GII.4 and GII.17 (Fig. S3D and L). These phylogenetic analyses yielded possible additional subgenotypes that were not included in the previously suggested subgenotypes. Each newly defined subgenotype of region C showed distinguishable sequence homology in within- and between-group distance analyses (*P* < 0.001, Mann-Whitney U test [Table S1]). Both GI.NA and GII.25 were excluded from subgenotyping because of the rarity of available isolated sequences (one isolated sequence each). Sequences in which subgenotypic clusters remained undefined were labeled “not clustered” (NC). All NCs were categorized as inter-or intragenotypic recombinants in subsequent results. Considering the consistency of ORF order between the term ORF1/2 junction and the previous dual-typing nomenclature (P type/vp1 genotype), this study used the previous dual-typing nomenclature instead of the recently suggested one (vp1 genotype [P type]) (7).

We described above that different amplicons, including identical subgenotypic region C sequences, originated from a single subgenotypic variant (22). To verify whether this was applicable to our data, common region C sequences were excised from both ORF1/ORF2 junction sequences and VP1 sequences, and both were then examined for subgenotypic clustering in relation to the subgenotypic lineages of common region C (Fig. S6). In these phylogenetic trees, subgenotypic clusters of both partial ORF1 sequences and partial VP1 sequences appeared simultaneously in the list of subgenotypic lineages excised from common region C. Such clustering, dependent on the subgenotypic lineages of common region C, was confirmed by verifying differences between within- and between-distances of subgenotypic ORF1/VP1 clusters (*P* < 0.001; Mann-Whitney U test). Thus, in subsequent analyses isolated sequences of three amplicon types sharing the same subgenotypic region C were regarded as sequences from the same subgenotypic viral variant.

### Isolation of recombinant sequences as evidence of mixed infection between parental noroviruses.

To identify recombinants in the isolated nucleotide sequences, phylogenetic tree analyses were performed based on recombination break points obtained from Recombination Detection Program 4 (RDP4). Fourteen possible recombinants, assumed to have emerged since 2014 and their possible parental lineages were identified (Table 1; Fig. S7 and S8).

**TABLE 1:** Genetic recombinants of genogroup I and genogroup II of norovirus isolated from water

Amplicon types of recombinants	Recombinant name^*a*^	Name of parental lineage 1^*b*^	Name of parental lineage 2^*b*^
Region C	GI.1R (*n* = 1)	[GI.P1]/GI.1b	[GI.P1]/GI.1a
	GI.4R (*n* = 1)	[GI.P4]/GI.4a	[GI.P4]/GI.4b
	GI.7R (*n* = 1)	[GI.P7]/GI.7a	[GI.P7]/GI.7c
	GII.6-13R (*n* = 1)	[GII.P7]/GII.6b	[GII.P16]/GII.13
VP1	GI.6-3R_LPV^*c*^ (*n* = 1)	[GI.P11]/GI.6a	[GI.P13]/GI.3d
	GII.2R_LPV (*n* = 1)	[GII.P16]/GII.2a	[GII.P16]/GII.2b
	GII.4R_LPV (*n* = 1)	[GII.P16]/GII.4ns	[GII.P31]/GII.4s
	GII.17R_LPV (*n* = 1)	[GII.P17]/GII.17n	[GII.P31]/GII.17o
	GII.4-8R_LPV (*n* = 2)	[GII.P31]/GII.4s	[GII.P8]/GII.8
ORF1/2 junction	GI.P4/GI.5a2 (*n* = 5)	GI.P4/[GI.4b]	[GI.P5]/GI.5a1
	GI.P7/GI.3d (*n* = 1)	GI.P7/[GI.7c]	[GI.P13]/GI.3d
	GII.P7/GII.6-4R (*n* = 1)	GII.P7/GII.6c	[GII.P16]/GII.4ns
	GII.P16/GII.4ns (*n* = 20)	GII.P16/[GII.2a]	[GII.P31]/GII.4s
	GII.P16-P17/GII.17nR (*n* = 1)	GII.P16/[GII.4ns]	GII.P17/GII.17n

a Both GenBank accession numbers and nucleotide sequences of the recombinants are provided in Data Set S2. Numbers in parentheses are numbers of isolated recombinant sequences.

b Each lineage name is composed of ORF1 lineage, slash, and ORF2 lineage. ORF lineages in brackets were indirectly inferred using the phylogenetic trees in Fig. S4 and S6.

c LPV, long PCR amplicon of VP1.

**FIG 3:**
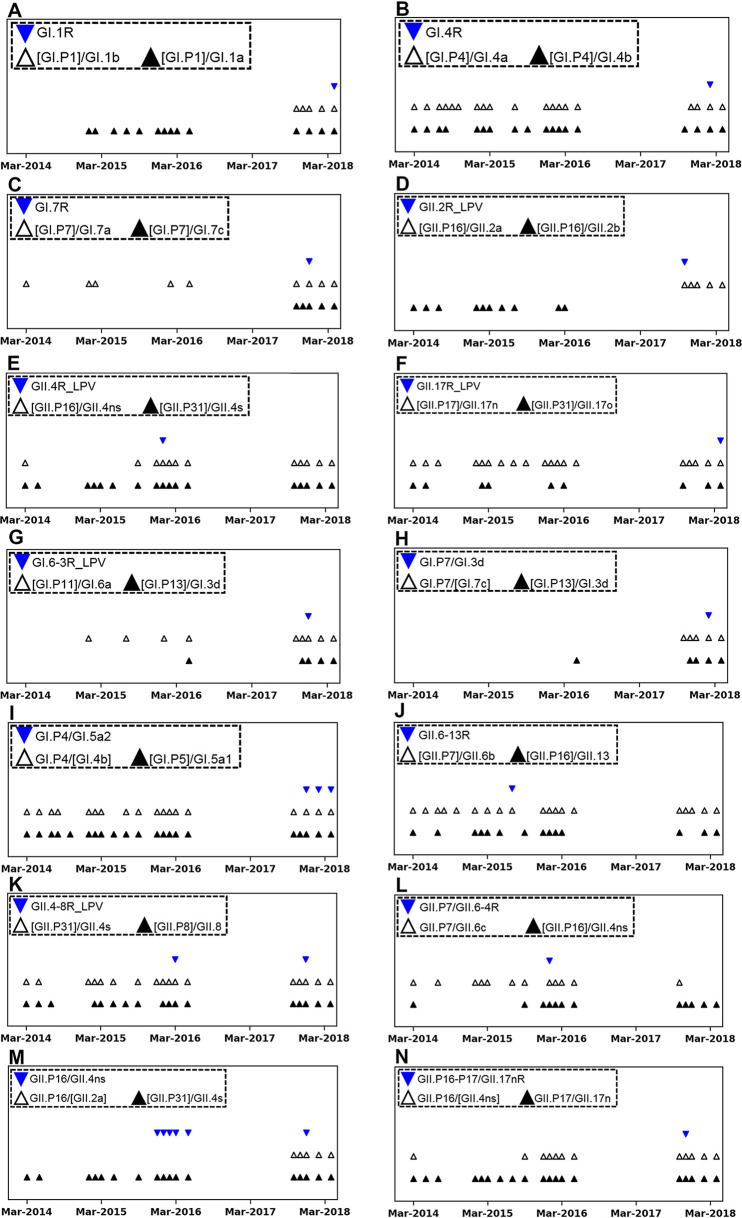
Timetable plots of sampling months positive for both genetic recombinants and their parental lineages. Marks on the *x* axis indicate March of each year in the study period (March 2014 to April 2018), including the no-sampling intraperiod from June 2016 to September 2017. Sampling months positive for recombinants are indicated as blue inverted triangles; sampling months positive for each parent of a recombinant are indicated as white triangles or black triangles. The names of both a recombinant and its parents are shown in the dotted box with corresponding symbols. The square bracket associated with each parental name indicates the name of an ORF lineage indirectly inferred from the phylogenetic analysis of common region C in sequences from the three amplicon types (region C, ORF1/2 junction, and VP1) in Fig. S4.

Genetic parents of norovirus recombinants must exist in the human population in advance of their recombinants (26). To verify whether our data also comply with the preexistence of parents, we designed timetable plots combining sampling periods with detected parental sequence cases (Fig. 3). The nucleotide sequences of the parents had occurred either before or simultaneously with the emergence of the recombinants during the study period. However, a possible ORF1 parent, GII.P16/GII.2a, of the recombinant, GII.P16/GII.4ns, was detected following the emergence of the recombinant (Fig. 3M). A past sequence (GenBank accession number MF972309; isolation year: 2007) closely related to the parental GII.P16/GII.2a in phylogenetic trees indicated that GII.P16/GII.2a existed below the detection limit in the water samples of the study area before recombinant emergence (Fig. S9). The timetable plots were redesigned to show detected cases of the parents in each province (Fig. S10), supporting the preexistence of both parents in the human population in each recombinant-positive province.

### Evaluation of possible mixed infection of noroviruses in the human population.

**FIG 4:**
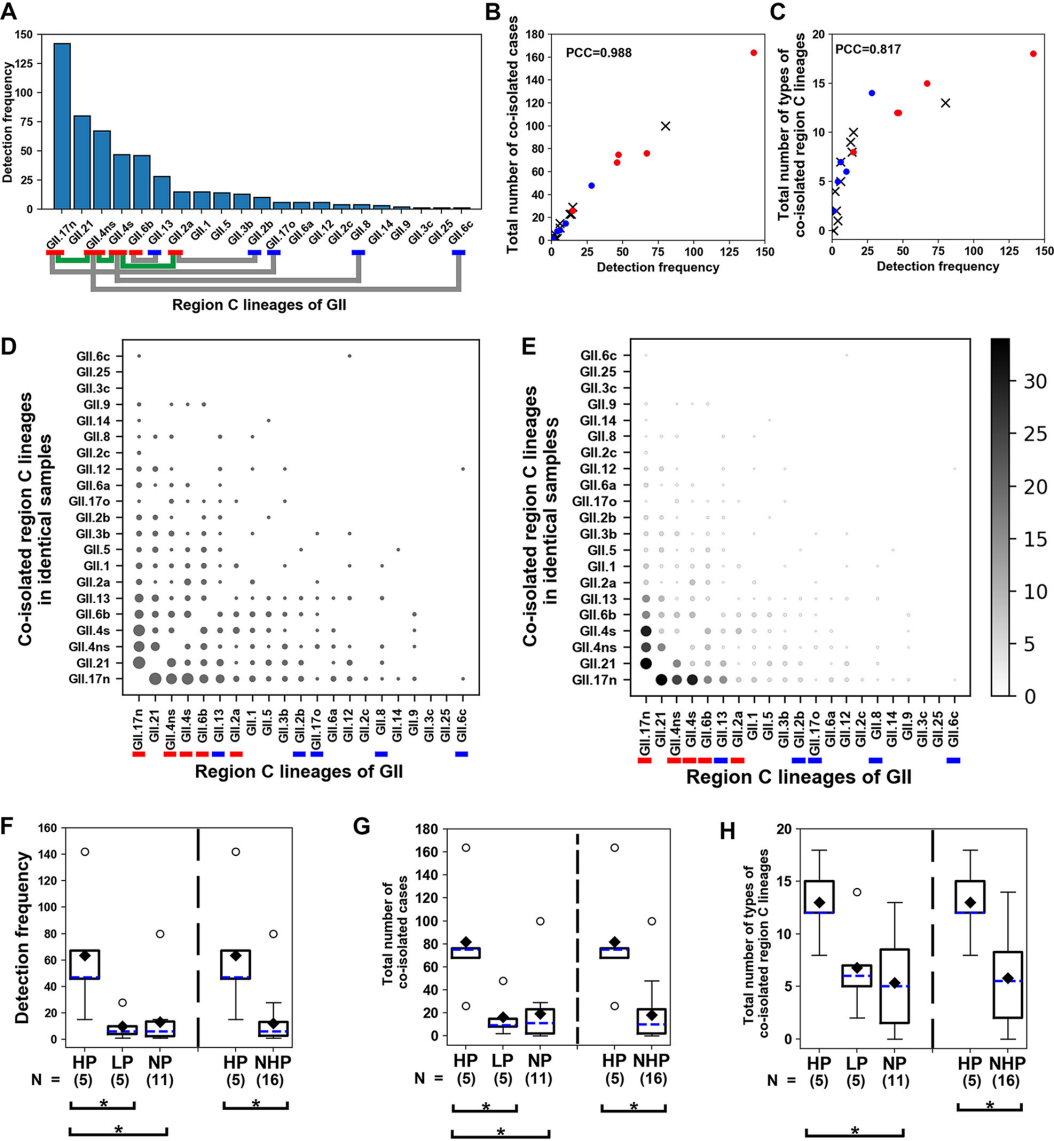
Relationships between detection frequencies and coisolation of GII region C amplicon lineages (2014 to 2018). (A) A vertical bar plot depicting detection frequencies of region C amplicon lineages isolated in the study period. Detection frequencies are arranged in descending order. Colored markers below the lineage names indicate HP (red) or LP (blue). A colored polygonal line connects parental pairs of each recombinant lineage described in Table 1 (green, HP-HP; gray, HP-LP). (B and C) Scatterplots describing both the total number of coisolated cases and the total number of types of coisolated lineages against the detection frequencies of each region C lineage in identical water samples. Red circles, HP; blue circles, LP; ×, nonparent (NP). PCC, Pearson’s correlation coefficient. (D and E) Bubble charts. Names of the lineages at both *x* axis and *y* axis are arranged in the same order of detection frequencies as in panel A. Colored markers below lineage names indicate either HP (red) or LP (blue). Names of the lineages at the *y* axis indicate lineages coisolated in identical water samples plotted against lineages on the *x* axis. The relative sizes of circles depict the number of cases of coisolated lineages on the *y* axis. To emphasize circle sizes, brightness was employed along with circle size in panel E. The number of cases of coisolated lineages and corresponding brightness are shown on the right side of the plot. (F to H) Box plots for comparing detection frequencies, the total numbers of coisolated cases, and the total numbers of the types of coisolated lineages in identical samples for the four groups, HP, LP, NP, and NHP. The vertical dashed line separates compared groups in each box plot. Sample size (N) of each group is indicated below the group names. Both mean (black diamonds) and median (blue dashes) are shown in each box. Asterisks describe the statistical significance in the differences of mean ranks between the two compared groups (Mann-Whitney U test).

Detection frequencies of the lineages of GII region C amplicons (Fig. 2) were redescribed as a vertical bar plot without recombinant sequences (Fig. 4). In this plot, polygonal lines connect between the genetic parents of each recombinant (Table 1), which were inferred common region C lineage of the three amplicon types. This vertical bar plot revealed that several highly prevalent lineages in the water samples (GII.17n, GII.4s, and GII.4ns) had generated recombinants with each other. However, rarely occurring parents such as GII.8 and GII.6c (2.8% and 0.7% of the detection frequency of GII.17n, respectively) were also able to generate recombinants, despite assumption of the relatively low number of available patients carrying them in the human population.

For the lineages rarely occurring in the human population to be the parents of a recombinant, their partner lineages had to produce a higher number of patients for a reasonable chance of generating a mixed infection. To test whether this scenario was probable, we categorized region C amplicon sequences of the parents into two groups (Fig. 4A): HP (high-competitiveness parents; a group of lineages showing higher detection frequencies, at least once, than the partner parent of each recombinant in the study period [GII.17n, GII.4ns, GII.4s, GII.6b, and GII.2a]) and LP (low-competitiveness parents; a group of lineages that did not show a higher detection frequency than the partner parent of each recombinant during the study period [GII.13, GII.2b, GII.17o, GII.8, and GII.6c]). All members of HP, except GII.2a, were ranked higher than the members of LP.

This study coisolated different norovirus lineages from identical water samples, and it was assumed that coisolation is indirectly interpreted as coexistence of different lineages in the human population that affected viral gene contamination of the water samples. To visualize coisolation data, we designed a plot (bubble chart) composed of circles (bubbles) in each column arranged against lineages that were isolated by the same order of detection frequency (Fig. 4D). The size of each circle in each lineage column reflects the total number of cases coisolated with other lineages in the same sample (Fig. S11). To assess relationship between the number and size of circles more efficiently in each column, an additional bubble chart was constructed, which correlated the size of the circle with its brightness (Fig. 4E). The resulting HP columns generally displayed more and larger circles than LP columns. To clarify this tendency, we created scatterplots illustrating the total sum of the number of coisolated cases in each column of the bubble chart (sum of circle sizes in each column [Fig. 4B]) or describing the total number of coisolated region C lineage types in each column of the bubble chart (sum of the number of circles in each column [Fig. 4C]) versus detection frequency of each lineage. These two scatterplots show that a higher detection frequency of a lineage strongly correlates with a higher chance of coisolation with other lineages (both cases and types) in the same water samples (Pearson’s correlation coefficients = 0.988 and 0.847). The scatterplots also showed that most HP lineages were located at higher values than LP lineages in both total numbers of coisolated cases and total numbers of the types of coisolated lineages.

Box plots were generated to compare the net differences between HP and LP for the three variables mentioned above (detection frequencies, total numbers of coisolated cases, and total numbers of the types of coisolated lineages) (Fig. 4F to H). We applied two additional groups, NP (nonparental lineages) and NHP (non-HP; LP plus NP), showing that the values of detection frequencies and total numbers of coisolated cases of HP were higher than those of LP, whereas the three values of LP were not different from those of NP. All HP values were different from those of NHP (Table S2). These results suggested that some GII lineages in NHP had generated recombinants owing to partner HP, which possesses a high chance of mixed infection with various lineages.

**FIG 5:**
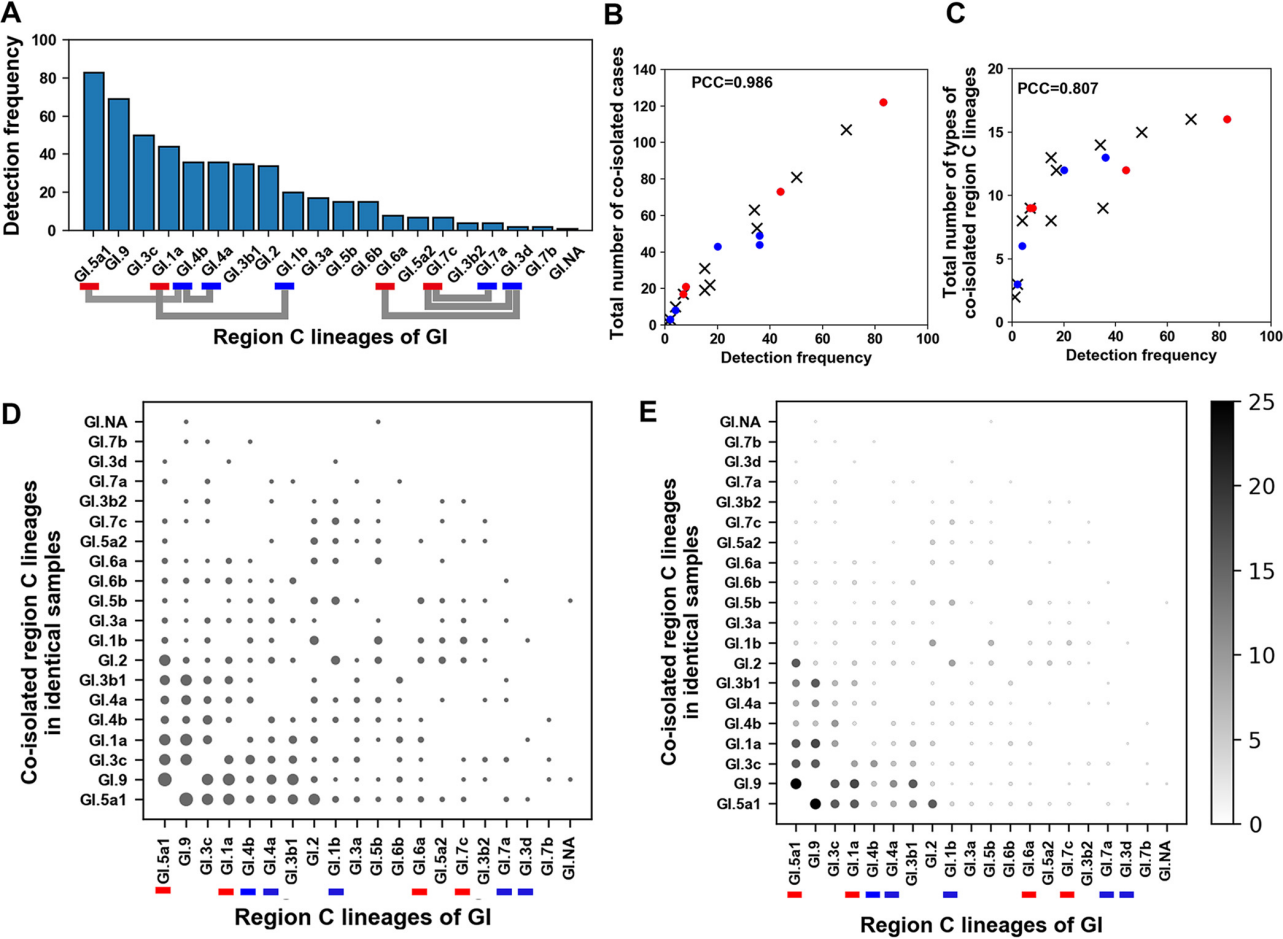
Relationships between detection frequencies and coisolation of GI region C amplicon lineages (2014 to 2018). Information for the panels is similar to that for the corresponding panels in Fig. 4.

To describe both the HP and LP of GI, a bar plot was generated (Fig. 5A) showing the detection frequencies of region C amplicon lineages, where parents were classified into HP and LP in the same manner as in Fig. 4A for GII.

In the bubble charts (Fig. 5D and E), however, we can see that at least five columns of GI lineages (GI.1b, GI.5b, GI.6a. GI.5a2, and GI.7c) to the right of GI.3b1 on the *x* axis included bigger circles in higher rows (upper rows of the GI.3b1 on the *y* axis), indicating that these lineages had coisolated with each other instead of coisolating with the lineages of higher detection frequencies (below GI.2 rows on the *y* axis). A possible explanation for such an inversion of circle sizes would be that in a short period, some GI lineages (GI.1b, GI.5b, GI.6a, GI.5a2, and GI.7c) showing higher detection frequencies than most other GI lineages had emerged in the waters. We concluded that the final 7 months of the study period (from October 2017 to April 2018) were associated with this inversion event, because GI.1b, GI.6a, and GI.7c of the parents occurred mostly during the final 7 months (Fig. 3 and Fig. S10).

Box plots drawn for comparing the three values among HP, LP, NP, and NHP did not differ significantly (Fig. S12). A possible factor responsible for the lack of significant differences may be the underestimation of data from 40 samples obtained during the final 7 months of the study period, due to the influence of data from the previous water samples (596 samples). Although the two lineages of HP, GI.5a1 and GI.1a, ranked 1st and 4th among the 20 detection frequency ranks, the other members of HP, such as GI.6a and GI.7c, ranked 13th and 15th, respectively, despite the inversion event. Thus, we focused on the data from the final 7 months for subsequent analyses of GI.

**FIG 6:**
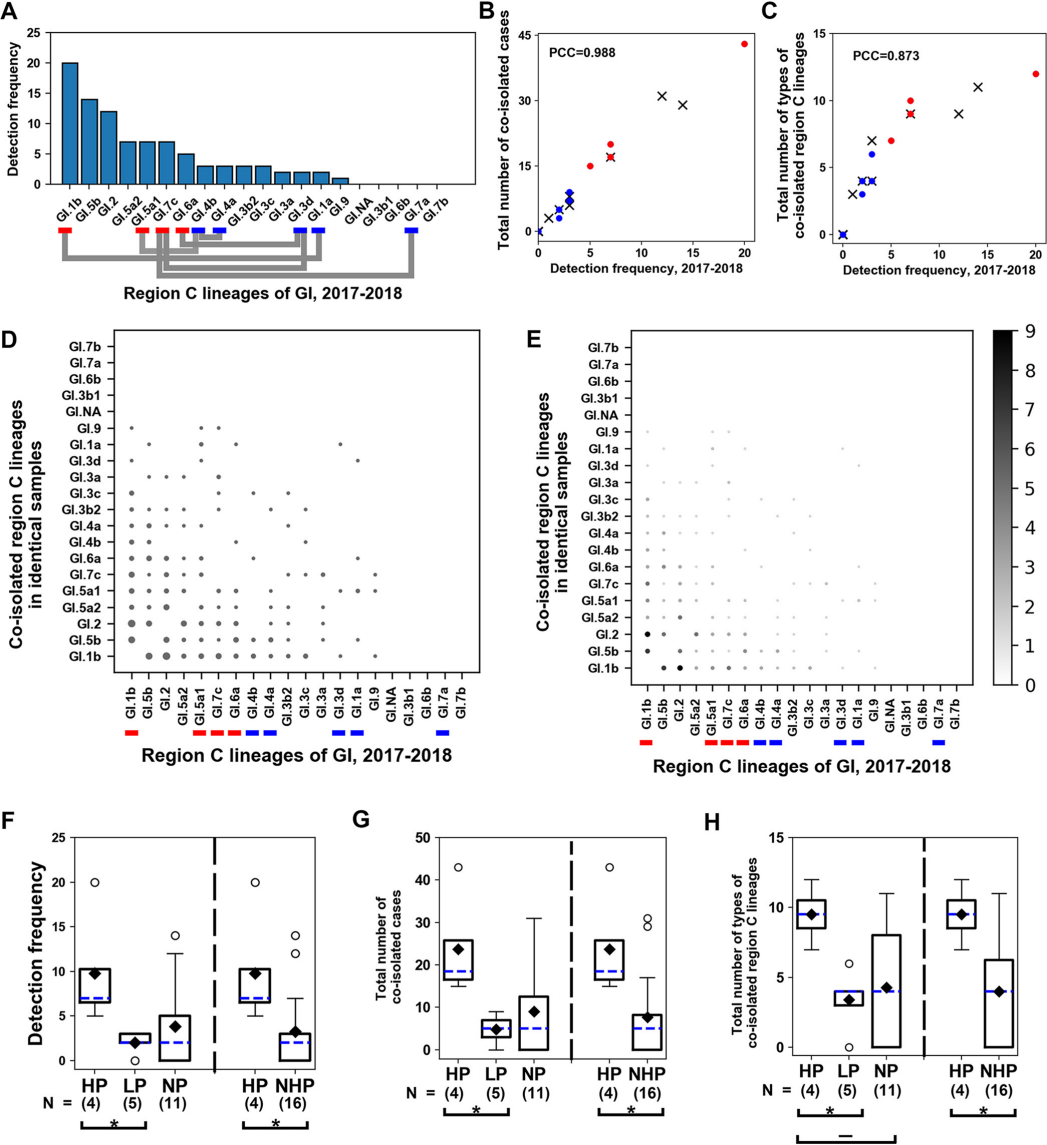
Relationships between detection frequencies and coisolation of GI region C amplicon lineages (2017 to 2018). Information for panels in this figure are similar to those of corresponding panel in Fig. 4. A dash above the bracket in panel H indicates the absence of significance following the Bonferroni correction, despite a *P* value of <0.05.

Using the GI data from the final 7 months, the plots were reconstructed, and the four groups, HP, LP, NP, and NHP, were sorted (Fig. 6). Because all novel GI recombinants first emerged in the final 7 months, we categorized the parents (HP and LP) without removing any recombinants. In the bar plot showing the lineages of GI region C amplicons, all lineages of HP ranked higher than those of LP (Fig. 6A). It was impossible to define HP between the two lineages because the detection frequencies of both parent GI.4a and its partner parent GI.4b were equal. Although the categorization strategy for HP and LP was designed not to consider the pairing within LP, the pairing between GI.4a and GI.4b was set as an alternative pair of within LP because GI.4b was LP compared with its HP partner GI.5a1. The scatterplots showed that HP lineages were located at higher values than LP lineages for both the number of coisolated cases and the number of coisolated lineage types (Fig. 6B and C). Box plots revealed that similar to the case with GII, some GI lineages in NHP had generated recombinants due to the partner HP’s high chance of mixed infection with various lineages (Fig. 6F–H; Table S3).

To check possible bias in the detection frequencies caused by mismatches between degenerate reverse transcription-PCR (RT-PCR) primers and their target sequences, we compared the six region C primers of the two genogroups and their target sequences on the isolated sequences and an additional 1,021 GenBank sequences (Fig. S13). Because mismatches in the 5′ half of the primers generally exerted a milder effect on nucleic acid amplification than those in the 3′ half (27), we focused on seven nucleotides from the 3′ end of the primers that would be most closely associated with DNA polymerase elongation deficiency (28). No lineage was related to consistent mismatches in the whole-sequence pool, revealing that no significant disturbance of nucleic acid amplification occurred for the isolated lineages.

### Emergence of recombinants is primarily driven by pairing between the HP and the LP, not by pairing within the HP.

To test the robustness of the categorization of both HP and LP, we recategorized the parental groups by modifying the raw detection frequency data from region C amplicon lineages. Random sampling was conducted on the supposition that the waters had been harvested from one sampling site in each province during the entire sampling period (Fig. S14). Subsequently, we created vertical bar plots describing two sets of mean values from randomized sampling as follows: (i) the number of positive sampling sites and (ii) the number of positive provinces against each parent during the study period (Fig. S15). The randomly sampled data of the two parents in each recombinant were significantly different from each other (*P* < 0.001 in the Mann-Whitney U test) except for the pairing between GI.4a and the GI.4b. Overlap of the 99% confidence intervals for the mean values was observed only once, in parental pair GI.4a and GI.4b, which revealed that most recombinants (13 of 14) were generated by unequal competitiveness combinations of the parents (Fig. S15C and F). Although changes in rank were observed for several lineages of the recategorized group (HP or LP) compared to the previous raw detection frequencies shown in Fig. 4A and 6A, there was no switching of lineages between parental groups, which indicated the lack of risk of weak categorization against the two parental groups (Fig. S15).

**FIG 7:**
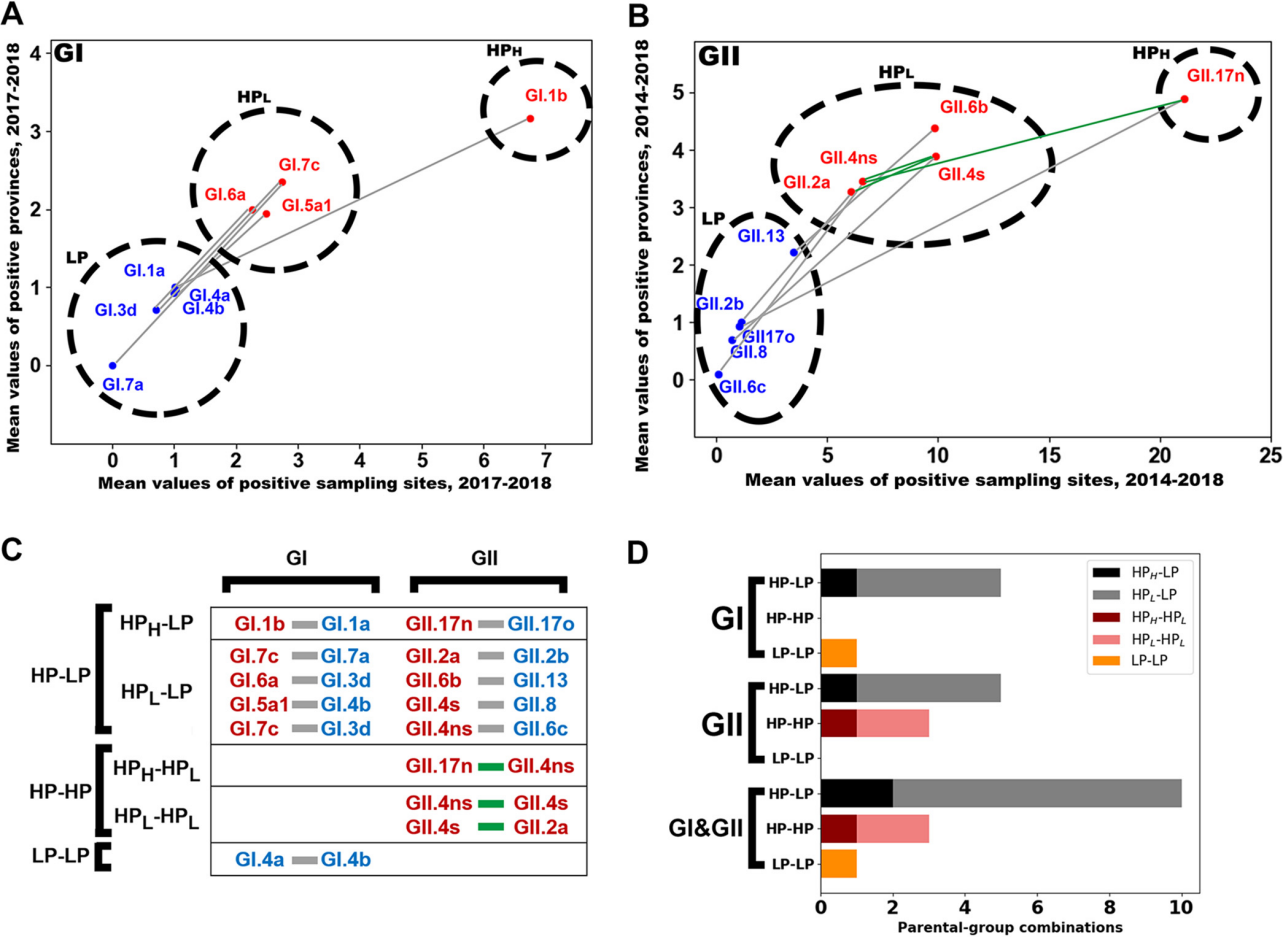
Emergence of parental groups with differential competitiveness, and ratio of pairs between the parental groups. (A and B) Scatterplots for mean values of two data sets of randomized samples (*x* axis = the number of positive sampling sites; *y* axis = the number of positive sampling provinces) against each parent. The name of the genogroup (GI or GII) corresponding to the analysis is shown on the upper left side of the scatterplot. Red dots indicate HP lineages, while blue dots indicate LP lineages. The lineage name is located near each corresponding dot. A pair of parents of each recombinant lineage is indicated by a colored line (green or gray) that connects the two parental lineage dots in a manner similar to that in Fig. 4A and 6A. The groups HP_H,_ HP_L,_ and LP, recategorized by both hierarchical clustering analysis and K-means clustering analysis, are shown by dashed circles. (C) Parental pairs that are connected by colored lines are shown in boxes, categorized by both genogroups (columns) and combinations of parental groups (rows). (D) A horizontal bar plot of emerging frequencies of different recombinants from three combinations of the parental groups (HP-LP, HP-HP, and LP-LP) of the two genogroups during the study period.

To ascertain whether a different categorization method would also support the HP and LP categorization, independent of our categorization strategy in Fig. 4 and 6, we constructed scatterplots describing combination of the two-typed mean values of randomly sampled data as two coordinates (Fig. 7A and B). The dots on the scatterplots were clustered by a combination of both hierarchical and K-means cluster algorithms (Fig. S16). Although there was no observed switching of lineages between two parental groups, clustering analysis indicated that the existence of at least two separate clusters in each HP of the two genogroups was essential to satisfy significance (*P* < 0.05) of the clustering (Table S4; Fig. S16E and F). Thus, we subdivided HP into HP_H_ (GI.1b and GII.17; a subgroup of high competitiveness in HP) and HP_L_ (a subgroup of low competitiveness in HP).

In the scatterplots, the randomly sampled data among the parental groups, HP_H_, HP_L_, and LP, were significantly different (*P* < 0.001, Mann-Whitney U test [Fig. S16B and D]). These results revealed that the assumed mixed infection cases within HP (HP_H_-HP_L_ and HP_L_-HP_L_) in the human populations could be greater than those of the other combinations (HP_H_-LP, HP_L_-LP, and LP-LP). However, the number of paired cases between parental groups of the two genogroups were as follows: 10 cases of HP-LP (8 HP_L_-LP and 2 HP_H_-LP), 3 cases of HP-HP (2 HP_L_-HP_L_ and 1 HP_H_-HP_L_), and 1 case of LP-LP (Fig. 7C). In contrast to the abundance of paired cases between the HP and the LP, two cases of HP_L_-HP_L_ and only one case of HP_H_-HP_L_ were recorded from the two genogroups. To determine possible interruption of RT-PCR amplification toward HP-HP recombinants in the two amplicon types, ORF1/2 junction and VP1, which were not investigated for Fig. S13, mismatches between primers and target sequences of parental sequences available in GenBank (GI ORF1, *n* = 41; GI ORF2, *n* = 94; GII ORF1, *n* = 653; GII ORF2, *n* = 757) were estimated (Fig. S17). There was no apparent predominance of mismatches in the seven critical nucleotides from the 3′ end of the primers against target sequences of HP. Thus, we concluded that the scarcity of pairing events within HP did not significantly correlate with primer mismatches. Considering that the preexistence of HP (GII.P16/GII.2a) in the water samples was below the detection limit required for emergence of recombinant GII.P16/GII.4ns, the HP-HP pair between GII.4s and GII.2a could be strictly interpreted as an HP-LP pair, leaving two HP-HP pairs in the whole data. Therefore, these results suggested that recombined HP genes have a higher chance of emergence when paired with LP genes than when paired with HP genes.

## DISCUSSION

In this study, we identified 14 recombinants assumed to have emerged since 2014, indicating that at least 14 different combinations of phylogenetically different lineages would have caused mixed infections in the human population. We divided the parents of the recombinants into HP and LP according to detection frequencies (raw measures of the competitiveness) in the water samples. Subsequent results suggested that norovirus lineages producing patients rarely (LP) generated recombinants owing to partner HP possessing a high chance of mixed infection with various lineages. These results also suggested a hypothesis that most recombinants had been generated from HP-HP pairing due to the assumption of more mixed infections than other combinations (HP-LP and LP-LP). However, there were more recombinant cases due to HP-LP pairing than those due to HP-HP pairing in the two genogroups.

Herd immunity against HP might be the cause of discrepancies observed between the assumed enrichment of mixed infections of HP and the rarity of emerging recombinants due to pairing within HP lineages. Studies associated with the herd immunity to human noroviruses have focused on the structural protein, VP1 (29), leaving unanswered queries regarding the herd immunity to the nonstructural proteins of human noroviruses. A recent report, however, has suggested that secreted extracellular nonstructural protein NS1 of the murine norovirus may regulate the interferon gamma-mediated immune response (30). Another study indicated that human norovirus infections might also cause antibody responses against nonstructural fusion proteins (31). Although many of the pathological roles associated with extracellular nonstructural proteins of norovirus remain unclear, these results suggest the possibility that antigenic targets of herd immunity against norovirus in the human population are likely not limited to structural proteins but also extend to extracellular nonstructural proteins. According to this hypothesis, any novel recombinant in the human population will be affected by already established herd immunity against the genetic products of each parent regardless of the nature of genes in recombinants. The more opportunities for parental norovirus exposure to the host’s immune system in the human population, the lower the chances for the progeny recombinants to be competitive in survival. Although recombinants generated from HP-HP pairing may possess higher chances to acquire a more competitive genetic profile than any other combinations (HP-LP and LP-LP), a previous study indicated that recombination of norovirus leads to a decrease in the number of infectious viral particles of novel recombinant in feces compared with those obtained with its parents (32). Thus, newly generated HP-HP recombinants, reduced in the excretion of infectious viral particles, would be more vulnerable to preestablished herd immunity against their parents. Our understanding of the heterogeneous pairing is that lineages dominant in the human population (HP) lead to recombinant generation owing to a high chance of mixed infection with various lineages, and in contrast, a certain genetic portion from a weakly maintained lineage (LP) provides a new genetic makeup for the survival of the HP-LP recombinant.

In addition to the involvement of herd immunity against the parental lineages, the difference in susceptibility to each parental norovirus lineage, based on unique receptor expression profiles in human cells, would be another factor affecting the emergence of recombinants based on reasonable chances of mixed infection between parental lineages (33). Future in-depth investigations on receptor susceptibilities will be helpful to more accurately estimate their impact on possible parental pairings. Future clinical trials dealing with mixed infection of parental lineages in strictly enclosed settings may provide a better chance of excluding recombinants that might have originated outside the human population. Additionally, future studies for long-term investigation of the fate of these norovirus recombinants and the possibility of differential herd immunity against recombinants based on antigenic similarity between parents may provide insights into the roles of HP-LP pairing in the emergence of novel recombinants of human noroviruses.

## MATERIALS AND METHODS

### Study areas, sample collection, and RNA extraction.

Stream water, raw sewage, and treated sewage effluent water from five provinces in the Republic of Korea (Gyeonggi, North Gyeongsang, South Gyeongsang, North Jeolla, and South Jeolla) were collected during two time periods (March 2014 to May 2016 and October 2017 to April 2018) (Fig. 1 and Data Set S1). To harvest both the sewage influent and the treated sewage effluent, one or two wastewater treatment plants were selected per province. A total of 636 water samples (wastewater-contaminated streams, 558; sewage influent, 28; and treated sewage effluent, 50) were collected during the study period. Sample collection and subsequent elution and concentration processes were performed following the U.S. Environmental Protection Agency method 1615 (34). Viral RNA was extracted from 140 μl of the water concentrates using a QIAamp viral RNA minikit (Qiagen, Hilden, Germany) according to the manufacturer’s protocol.

### Nucleic acid amplification, cloning, and sequence analysis.

The ORF1/ORF2 junction (GI, 1.2 kb; GII, 1.0 kb), VP1 (GI, 1.6 kb, full ORF2; GII, 1.5 kb, nearly full ORF2), and region C (0.3 kb, region C in VP1) of human norovirus genogroup I (GI) and genogroup II (GII) were amplified from RNA extracts using combinations of degenerate primers according to the amplification conditions described in Table 2 and Tables S5 and S6. Amplified products of the target gene were purified using either a FavorPrep GEL/PCR purification minikit (Favorgen, Taiwan) or a MiniBEST agarose gel DNA extraction kit version 4.0 (TaKaRa, Kusatsu, Japan) and cloned using a Mighty TA-cloning kit (TaKaRa) with chemically competent Escherichia coli DH5α (Enzynomics, Daejeon, Republic of Korea) according to the manufacturer’s protocol. Cloned genes in multiple colonies per amplicon (region C, 6 colonies; both ORF1/ORF2 junction and VP1, 6 to 36 colonies) were purified using either a FavorPrep plasmid extraction minikit (Favorgen) or a HiYield plasmid minikit (RBC, Banqiao, Taiwan) according to the manufacturer’s protocol. Plasmid extracts required for Sanger sequencing (Macrogen, Seoul, Republic of Korea) were carried out using a 3730xl DNA analyzer (Thermo Fisher Scientific, Waltham, MA) with a primer set (M13R, 5′-GCGGATAACAATTTCACACAGG-3′, and pMD20_R, 5′-TTCGCTATTACGCCAGCTG-3′). Nucleotide sequences from false-positive clones were filtered out via the BLASTn program (NCBI).

**TABLE 2:** Primers used in the study

Genogroup	Primer ID	Sequences (5′→3′)^*a*^	Polarity	Positions^*b*^	Reference
I	GI-FIM	CTGCCCGAATTYGTAAATGATGAT	Sense	5342–5365	20
	GI-RIM	CCAACCCARCCATTRTACATYTG	Antisense	5648–5671	20
	GI-F2	ATGATGATGGCGTCTAAGGACGC	Sense	5358–5380	20
	GICRVP2	ATDGCWCCDADDAYDGCTTGRGCCATTAT	Antisense	6947–6975	Current study
	GICR600	CGVARNGGNGTRTANARCAT	Antisense	5909–5929	Current study
	GIPF1000	AGGAAGAATGATGAYTGGAA	Sense	4220–4239	Current study
	GIPF800	CCNTTYTGTGANGCYATAAA	Sense	4445–4464	Current study
II	GII-F1M	GGGAGGGCGATCGCAATCT	Sense	5049–5068	20
	GII-R1M	CCRCCNGCATRNCCRTTRTACAT	Antisense	5367–5389	20
	GII-F3	TTGTGAATGAAGATGGCGTCGART	Sense	5079–5102	20
	GIICR1450	ACCCARGMNTCAAAYCTRAART	Antisense	6622–6643	19
	GIIPF800M	GATGCWGAYTAYTCYMGNTGGGA	Sense	4289–4311	19
	GIIPF750M	CNGCHHTAGARRTNATGGT	Sense	4341–4359	19

a R, A/G; Y, C/T; W, A/T; H, A/C/T; V, A/C/G; D, A/G/T; N, A/C/G/T; M, A/C.

b Primer positions of norovirus genogroup I are based on GenBank accession number JX023285, and primer positions for norovirus genogroup II are based on GenBank accession number X86557.

### Defining subgenotypic lineages of common region C in isolated norovirus nucleotide sequences.

Genotypes of nucleotide sequences of both GI and GII were identified using the Norovirus Genotyping Tool (25). Subgenotypic lineages of the common region C in the three amplicon types were further identified via phylogenetic tree construction using reference sequences suggested in a previous study (5). To construct the phylogenetic tree, nucleotide sequences of region C of the three amplicon types were aligned using Clustal Omega (windows version 1.2.2) (35). The tree was constructed using the neighbor-joining method with the p-distance model and 1,000 bootstrap replicates on Molecular Evolutionary Genetic Analysis version 7.0 (MEGA7) (36). A cluster of at least three sequences with a bootstrap value of ≥50 was used as a criterion for selecting a candidate for a new subgenotype in this study. To confirm sequence homologies of subgenotype candidates, the Mann-Whitney U test was used to compare “within-group distances” with “between-group distances” of intragenotypic clusters.

### Identification of possible recombinants and their parents from norovirus sequences isolated in this study.

The identification of both recombinants and their parents was conducted in two steps. In step one, possible recombinant sequence candidates, their parental sequence candidates, and recombination breakpoints were identified using seven methods (RDP, GENECONV, BootScan, MaxiChi, Chimaera, SiScan, and 3Seq) (37) available in RDP4 with default settings (38). In step two, sequences of each of the recombinants and their parental candidates were split into two- or three-fragment groups based on recombination breakpoints. The existence of both possible recombinants and their parental lineages was confirmed by constructing a phylogenetic tree for each fragment group using the p-distance method with 1,000 bootstrap values.

To reduce possible false positives in the RDP4 result before carrying out the second step, namely, phylogenetic tree analysis, we combined two acceptance limits derived from standard recombinants (STRs) in RDP4 analyses as follows: (i) the number of positive STR signals and (ii) positive signals satisfying a *P* value of <0.01. This *P* value limit was derived from the highest *P* value acceptances of a previous study using RDP4 (39). STRs were constructed by cutting-off-and-joining suggested reference sequences (5) to mimic inter- and intragenotypic recombinants. Flowcharts depicting the strategy used to construct STRs are available in the protocol in the supplemental material.

STR sequences were prepared by combining fragments of norovirus reference sequences suggested in a previous study (5) rather than using natural recombinant sequences deposited in GenBank (protocol in the supplemental material). Thus far, natural recombinant sequences have been reported to emphasize the high significance of positive signals originating from parents evolutionarily distant to each other (26). If natural recombinant sequences were used in RDP4 analysis, as the STRs for the lowest acceptance limit of the number of positive signals, any true recombinant originating from parents evolutionarily close to each other would lead to a false-negative decision. Thus, we prepared STRs by cutting-off-and-joining phylogenetically distinct reference sequences that exhibited the highest identity homology with each other (minimum length of a branch between phylogenetically different reference sequences) in a neighbor-joining tree.

To select sequences for the STRs, phylogenetic reference sequences of both ORF1 and ORF2 of the two genogroups (5) were prepared according to coverages of the isolated sequences in the study area (ORF1 of ORF1/2 junction, 0.7 to 0.9 kb; region C of VP1, 0.3 kb; and VP1, 1.5 to 1.6 kb). A total of six neighbor-joining trees (GI ORF1, GII ORF1, GI region C, GII region C, GI VP1, and GII VP1) were constructed using prepared reference nucleotide sequences of both ORF1 (GI, seven P genotypes; GII, 25 P genotypes) and ORF2 (GI, 16 lineages, including the subgenotypes; GII, 29 lineages, including the subgenotypes). In each tree, the pair of different genotype sequences, or the pair of different subgenotype sequences, that showed the highest identity homology with each other was selected. A pair of subgenotype reference sequences of the GII region C (GenBank accession numbers AJ277620 and HM633213) was also chosen despite showing the lowest degree of shared identity, as the STRs of other subgenotype pairs could not be positively determined via RDP4 analysis. For the same reason, a pair of subgenotypic VP1 of GII (GenBank accession numbers AB445395 and GQ845367) showing the second-highest shared identity was chosen. Each prepared STR was assigned an identifier (ID) from A0 to R1 (protocol in the supplemental material).

In the Clustal Omega alignment for RDP4 analysis, one of the STRs and its two parental reference sequences were aligned with isolated norovirus sequences. Combinations between the aligned STR and the aligned isolated sequences were adjusted according to the purpose behind the estimation of the recombination type (inter- or intragenotypic recombination) and the target gene of the isolated sequences (Data Set S2 and protocol in the supplemental material).

Besides filtering out possible false positives, using STR-based cutoff values, RDP4 results showing recombinant candidates with unknown parental sequences (uncertainty of parent discrimination among the isolated sequences) were also filtered out. To reduce the risk of false-positive breakpoints caused by common RT-PCR primer sequences near both ends of the recombinant candidate sequences, estimated breakpoints located up to 60 nucleotides (nt) from each end of the sequences were ignored. To filter out possible nonnovel recombinants that emerged before 2014 from recombinant candidates, any GenBank sequences (isolated before 2014) showing over 95% identity with recombinant candidate sequences in NCBI BLAST were investigated via both RDP4 analysis and the neighbor-joining tree analysis described above (Data Set S2).

### Statistical analyses.

The Statistical Package for the Social Sciences software (SPSS, version 25.0.0; IBM Corporation, USA) was used to perform statistical analyses. To test the normality of data, a Kolmogorov-Smirnov test-based Lilliefors test was used. Because most data sets used in this study were not normally distributed, we used nonparametric tests to determine statistical significance. To compare multiple data sets, the Kruskal-Wallis H test was used, whereas the Mann-Whitney U test (two-tailed) was used to compare pairs in multiple data sets. In this case, the upper limit of the *P* value (0.05) used for indicating significance was adjusted via the Bonferroni correction (0.05/total number of the multiple data sets). To compare only two data sets using the Mann-Whitney U test without the Bonferroni correction, statistical significance was set at a *P* value of <0.05. SPSS results yielding a *P* value of 0.000 are depicted as *P* < 0.001. In plots, error bars indicate standard deviations, or 99% confidence intervals of each mean (40, 41). Pearson’s correlation coefficient was calculated as described previously (42).

### Cluster analysis.

SPSS was used to conduct both hierarchical cluster analysis and K-means cluster analysis. In hierarchical cluster analysis, within-group linkage with Euclidean distance was used to differentiate subgroups as per *x* and *y* coordinates of two variables (the number of positive sampling sites and the number of positive provinces [Fig. 7]). In K-means cluster analysis, one-way analysis of variance (ANOVA) was performed to verify the significance of differences between specified initial groups. According to the Elbow method, defining the point of inflection on the curve, the optimal number of partitions in K-means cluster analysis was set (https://doi.org/10.5281/zenodo.3687330) (43).

### Construction of maximum clade credibility tree.

The Bayesian Evolutionary Analysis Sampling Trees (version 1.8.10) program was used to analyze phylogenetic relationships between the isolated nucleotide sequences (44). Convergence was checked for an effective sample size (ESS) which accepted parameters with an ESS of >200. Following a 10% burn-in, the MCC tree was selected using Tree Annotator (version 1.8.2). The time-scaled phylogenetic tree was visualized in FigTree (version 1.4.2).

To analyze both GII.4 and GII.17 region C sequences, nucleotide sequences from region C in GII.4 or GII.17 ORF2 isolated in this study were aligned using Clustal Omega and subsequently processed via MEGA7 to determine the best nucleotide substitution model. Next, the “GTR + gamma + Invariant” model (for GII.4) or “GTR + gamma” model (for GII.17) was selected. The Markov chain Monte Carlo (MCMC) chains were run for 1 billion steps using both the strict clock model and the coalescent constant tree model.

To analyze partial GII.P16 sequences, nucleotide sequences of partial GII.P16 in the NCBI GenBank (*n* = 788; date of data collection: March 2019) were collected and aligned using Clustal Omega and subsequently processed via MEGA7 to determine the best nucleotide substitution model. Next, the TN93 model was selected. To select the best clock model for the data set, stepping-stone sampling was performed using 1 billion chains constructed by combining both clock models (strict and uncorrelated lognormal) and tree models (coalescent constant, coalescent exponential, expansion, and logistic). After combining the best clock model with the best tree model (the uncorrelated lognormal relaxed/coalescent exponential), MCMC chains were run for 10 billion steps with log parameters of 10,000. Two independent tree files were combined using LogCombiner with a resampling state of 200,000.

### Plots for data presentation.

The Python (version 3.6 for Windows 7) program was used to generate all plots in this study. PyCharm (2019.1.1, community edition; JetBrains s.r.o.) was used to construct Python scripts.

### Bubble charts for coisolation data.

Module “matplotlib.pyplot.scatter” in the Python script was used to generate bubble charts (Fig. S11). To differentiate circle sizes, coisolation cases (number of cases coisolated with the other lineages plotted against a specific lineage) were linked with the marker size parameter “s” of the module.

### Randomized sampling to modify raw detection frequencies by normalizing geographical/seasonal imbalance in the number of available sampling sites.

A single sampling site in each province was randomly sampled each sampling month using the module “random” in the Python script (Fig. S14). This process was performed against each lineage of region C amplicons that emerged during the study period. If the randomly sampled site on a specific date corresponded with a real positive case against a certain lineage, the value 1 (number of the corresponding positive sites) was recorded cumulatively through each sampling month in the study period, leading to a cumulative value. Two different cumulative values, where one represents the number of positive sampling sites in the study period and the other represents the number of positive provinces in the study period, were obtained per lineage, and this process was repeated until 1,000 cumulative values were obtained for each lineage. Two mean values from both the 1,000 cumulative values of the number of positive sampling sites (*x* coordinate) and the 1,000 cumulative values of the number of positive provinces (*y* coordinate) against each lineage were used to describe each dot in the scatterplots (Fig. 7). To define the 99% confidence interval (Fig. S15), a set of 1,000 resamplings from 1,000 cumulative values was repeated via 1,000 bootstraps. A sampled mean value was calculated from each set of 1,000 resamplings, leaving 1,000 sampled mean values from the 1,000-bootstrap set. The 99% confidence interval was calculated via multiplication of the *z* value (2.58) with the standard deviation of 1,000 sampled mean values (41).

### Data availability.

Python scripts for Fig. 4, 6, and 7 are available at https://github.com/KESvirol/codes_for_publication. GenBank accession numbers of whole isolated norovirus sequences in the study areas are provided in Data Set S1. The raw data underlying Fig. 1, 3, 4, 6, and 7 and Fig. S1, S9, S10, S13, S15, S16, and S17 are provided in Data Set S1. Raw data relevant to the RDP4 analysis are provided in Data Set S2.

## Supplementary Material

Supplemental file 1

Supplemental file 2

Supplemental file 3
