# Supplementary material for: Pairing of Parental Noroviruses with Unequal Competitiveness Provides a Clear Advantage for Emergence of Progeny Recombinants
Source: Appl Environ Microbiol. 2021 Jan 15;87(3):e02015-20. doi: 10.1128/AEM.02015-20 (PMC7848925; doi:10.1128/AEM.02015-20)
Supplement: Supplemental file 1 [file AEM.02015-20-s0001.pdf]

Supplemental Material

FIG S1 [Pages 1-2]

FIG S2 [Pages 3-4]

FIG S3 [Pages 5-6]

FIG S4 [Pages 7-39]

FIG S5 [Pages 40-41]

FIG S6 [Pages 42-44]

FIG S7 [Pages 45-48]

FIG S8 [Pages 49-75]

FIG S9 [Pages 76-78]

FIG S10 [Pages 79-93]

FIG S11 [Pages 94-95]

FIG S12 [Pages 96-97]

FIG S13 [Pages 98-100]

FIG S14 [Pages 101-102]

FIG S15 [Pages 103-105]

FIG S16 [Pages 106-107]

FIG S17 [Pages 108-110]

TABLES S1-S6 [Pages 111-116]

Protocol [Pages 117-127]

References [Page 128]

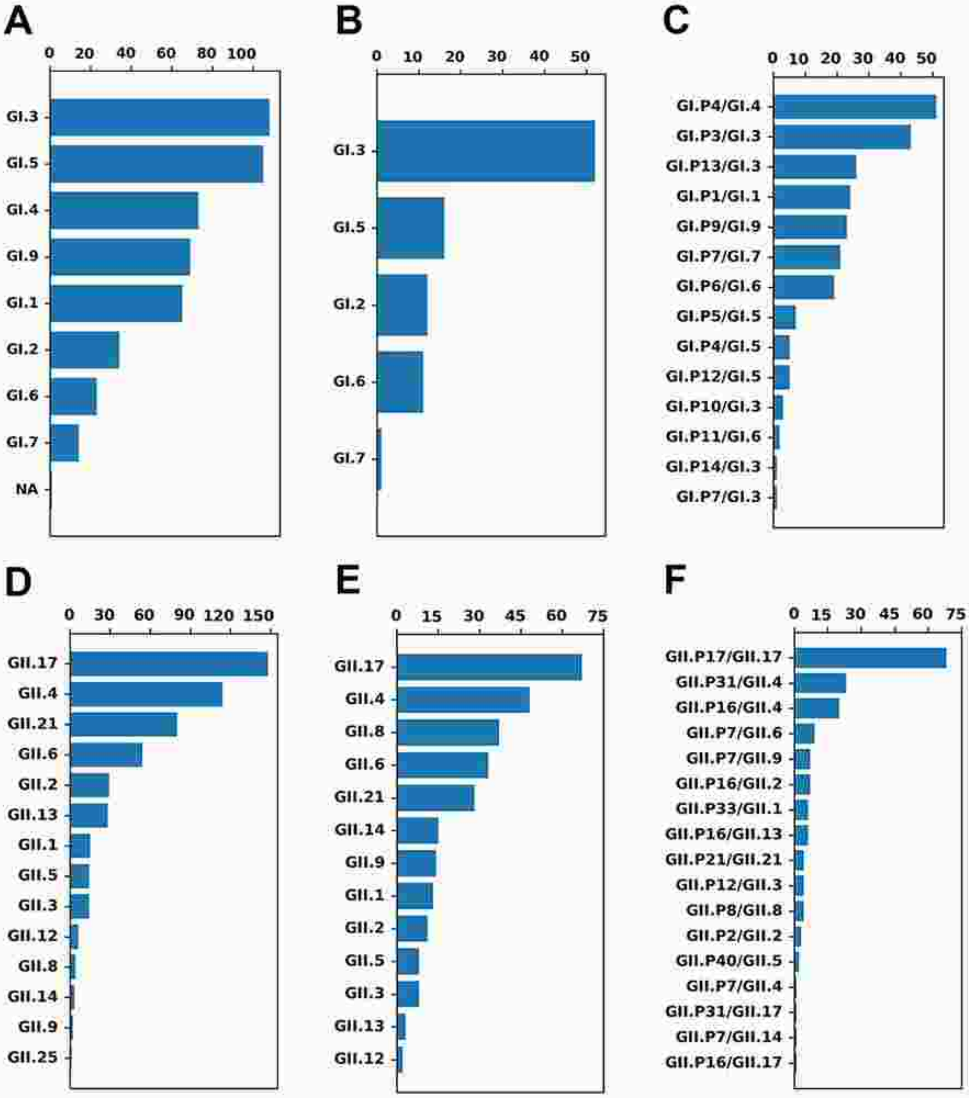
A
0 20 40 60 80 100
GI.3
GI.5
GI.4
GI.9
GI.1
GI.2
GI.6
GI.7
NA
B
0 10 20 30 40 50
GI.3
GI.5
GI.2
GI.6
GI.7
C
0 10 20 30 40 50
GI.P4/GI.4
GI.P3/GI.3
GI.P13/GI.3
GI.P1/GI.1
GI.P9/GI.9
GI.P7/GI.7
GI.P6/GI.6
GI.P5/GI.5
GI.P4/GI.5
GI.P12/GI.5
GI.P10/GI.3
GI.P11/GI.6
GI.P14/GI.3
GI.P7/GI.3
D
0 30 60 90 120 150
GII.17
GII.4
GII.21
GII.6
GII.2
GII.13
GII.1
GII.5
GII.3
GII.12
GII.8
GII.14
GII.9
GII.25
E
0 15 30 45 60 75
GII.17
GII.4
GII.8
GII.6
GII.21
GII.14
GII.9
GII.1
GII.2
GII.5
GII.3
GII.13
GII.12
F
0 15 30 45 60 75
GII.P17/GII.17
GII.P31/GII.4
GII.P16/GII.4
GII.P7/GII.6
GII.P7/GII.9
GII.P16/GII.2
GII.P33/GII.1
GII.P16/GII.13
GII.P21/GII.21
GII.P12/GII.3
GII.P8/GII.8
GII.P2/GII.2
GII.P40/GII.5
GII.P7/GII.4
GII.P31/GII.17
GII.P7/GII.14
GII.P16/GII.17

Fig S1

FIG S1 The total number of nucleotide sequences of two open reading frames (ORF) genotypes isolated from both norovirus genogroup I (GI) and genogroup II (GII). **(A)**–**(F)** Horizontal bar plots for the total number of isolated genotypic sequences of region C amplicons (0.3 kb; GI, panel **A**; GII, panel **D**), VP1 amplicons (1.5-1.6 kb; GI, panel **B**; GII, panel **E**), or ORF1/2 junction amplicons (1.0-1.2 kb; GI, panel **C**; GII, panel **F**). In each plot, the y-axis indicates the names of genotypic lineages; the x-axis indicates the total number of isolated sequences. Considering the consistency of ORF order between the term ORF1/2 junction and the previous dual-typing nomenclature (P-type / vp1 genotype), panels **C** and **F** used the previous dual-typing nomenclature instead of the recently suggested one (vp1 genotype [P-type]) (1).

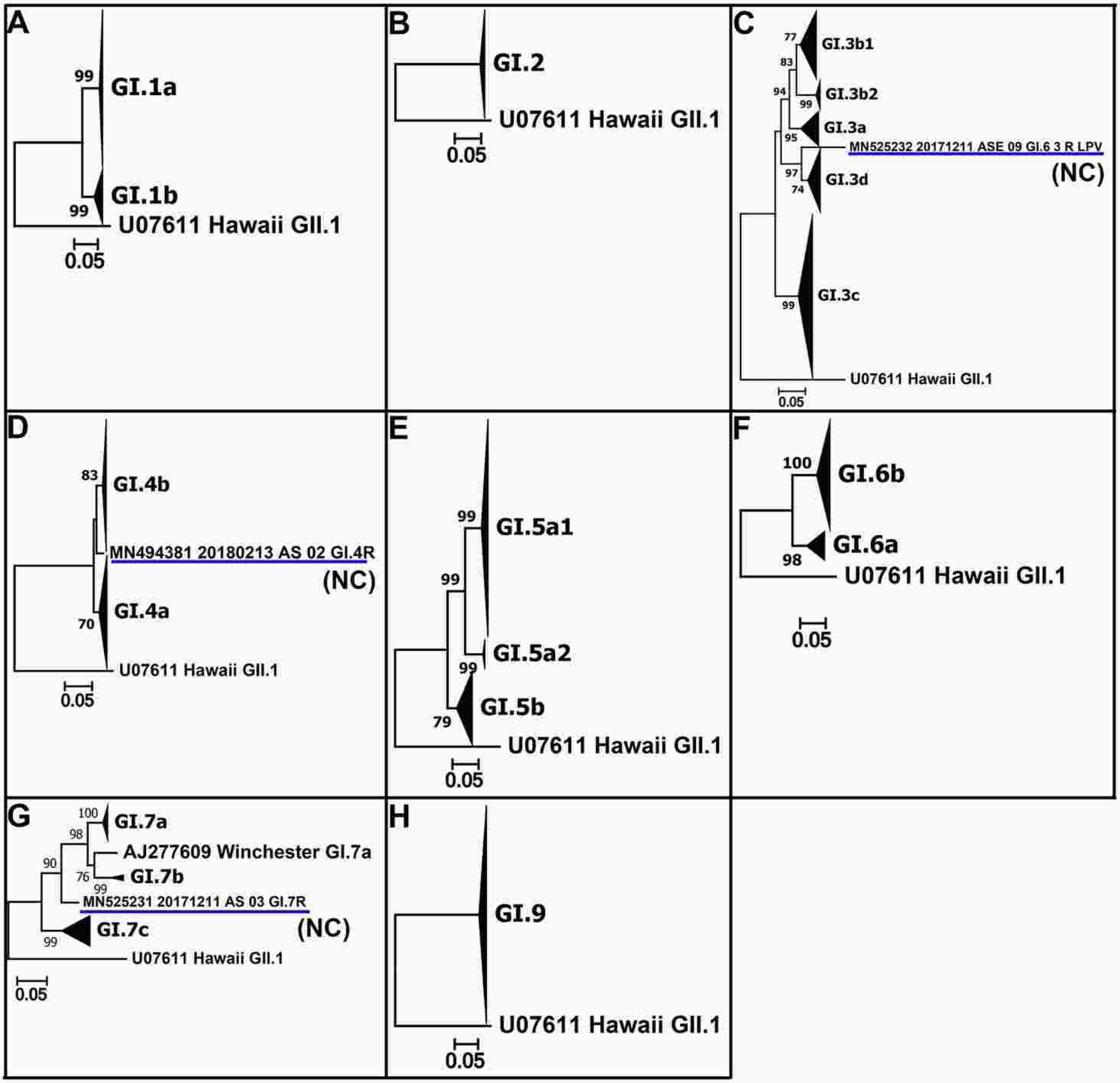
A
99
GI.1a
GI.1b
99
U07611 Hawaii GII.1
0.05
B
GI.2
U07611 Hawaii GII.1
0.05
C
77
GI.3b1
83
94
GI.3b2
99
GI.3a
95
MN525232 20171211 ASE 09 GI.6 3 R LPV
(NC)
97
GI.3d
74
GI.3c
99
U07611 Hawaii GII.1
0.05
D
83
GI.4b
MN494381 20180213 AS 02 GI.4R
(NC)
GI.4a
70
U07611 Hawaii GII.1
0.05
E
99
GI.5a1
99
GI.5a2
99
GI.5b
79
U07611 Hawaii GII.1
0.05
F
100
GI.6b
GI.6a
98
U07611 Hawaii GII.1
0.05
G
100
GI.7a
98
AJ277609 Winchester GI.7a
90
76
GI.7b
99
MN525231 20171211 AS 03 GI.7R
(NC)
GI.7c
99
U07611 Hawaii GII.1
0.05
H
GI.9
U07611 Hawaii GII.1
0.05

Fig S2

FIG S2 Compressed phylogenetic trees of region C (partial ORF2; 0.3 kb) nucleotide sequences isolated from norovirus genogroup I (GI). **(A)–(H)** Compressed trees for sub-genotyping of common region C sequences of the isolated three amplicon types (region C, VP1, and ORF1/2 junction) of the GI (panel **A**, GI.1; panel **B**, GI.2; panel **C**, GI.3; panel **D**, GI.4; panel **E**, GI.5; panel **F**, GI.6; panel **G**, GI.7; and panel **H**, GI.9). An outgroup reference strain (GII.1; GenBank ID: U07611) is shown in each tree. Names of sub-genotypes or names of genotypes are marked next to the tree clusters. Bootstrap values ≥ 65 are shown next to sub-genotype branches. Sequences underlined in blue indicate sequences that are independent of the other clusters (NC = not clustered) in the tree. Expanded views of the compressed trees are shown in Fig. S4. A scale bar (p-distance, 0.05) is shown for each tree.

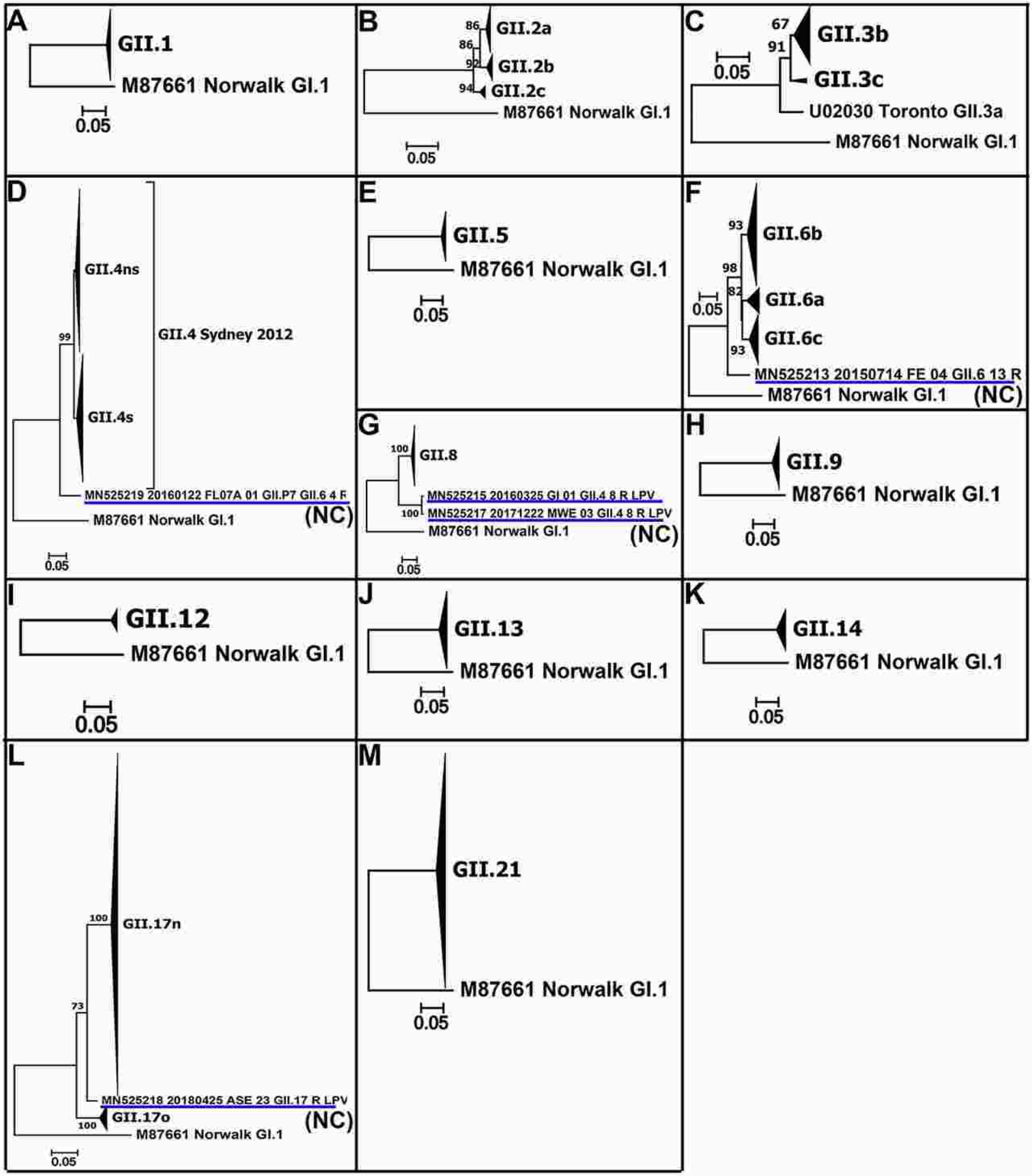
A
GII.1
M87661 Norwalk GI.1
0.05
B
86
GII.2a
86
92
GII.2b
94
GII.2c
M87661 Norwalk GI.1
0.05
C
67
GII.3b
91
GII.3c
0.05
U02030 Toronto GII.3a
M87661 Norwalk GI.1
D
GII.4ns
99
GII.4 Sydney 2012
GII.4s
MN525219 20160122 FL07A 01 GII.P7 GII.6 4 R
M87661 Norwalk GI.1
(NC)
0.05
E
GII.5
M87661 Norwalk GI.1
0.05
F
93
GII.6b
98
82
GII.6a
0.05
GII.6c
93
MN525213 20150714 FE 04 GII.6 13 R
M87661 Norwalk GI.1
(NC)
G
100
GII.8
MN525215 20160325 GI 01 GII.4 8 R LPV
100
MN525217 20171222 MWE 03 GII.4 8 R LPV
M87661 Norwalk GI.1
(NC)
0.05
H
GII.9
M87661 Norwalk GI.1
0.05
I
GII.12
M87661 Norwalk GI.1
0.05
J
GII.13
M87661 Norwalk GI.1
0.05
K
GII.14
M87661 Norwalk GI.1
0.05
L
100
GII.17n
73
MN525218 20180425 ASE 23 GII.17 R LPV
GII.17o
100
M87661 Norwalk GI.1
(NC)
0.05
M
GII.21
M87661 Norwalk GI.1
0.05

Fig S3

FIG S3 Compressed phylogenetic trees of region C (partial ORF2; 0.3 kb) nucleotide sequences isolated from norovirus genogroup II (GII). **(A)–(M)** Compressed trees for sub-genotyping of common region C sequences isolated from three amplicon types (region C, VP1, and ORF1/2 junction) of the GII (panel **A**, GII.1; panel **B**, GII.2; panel **C**, GII.3; panel **D**, GII.4; panel **E**, GII.5; panel **F**, GII.6; panel **G**, GII.8; panel **H**, GII.9; panel **I**, GII.12; panel **J**, GII.13; panel **K**, GII.14; panel **L**, GII.17; and panel **M**, GII.21). An outgroup reference strain (GI.1; GenBank ID: M87661) is shown in each tree. Names of sub-genotypes or names of genotypes are marked next to tree clusters. Bootstrap values of ≥ 65 are shown next to the sub-genotype branches. Sequences underlined in blue indicate sequences that are independent of the other clusters (NC = not clustered) in the tree. Expanded views of the compressed trees are shown in Fig. S4. A scale bar (p-distance, 0.05) is shown for each tree.

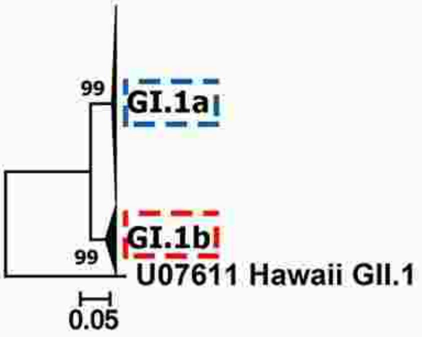

Marker	Amplicon type	Name of lineage
○	Region C	GI.1a
□	ORF1/2 junction	GI.P1/GI.1a
●	Region C	GI.1b
■	ORF1/2 junction	GI.P1/GI.1b

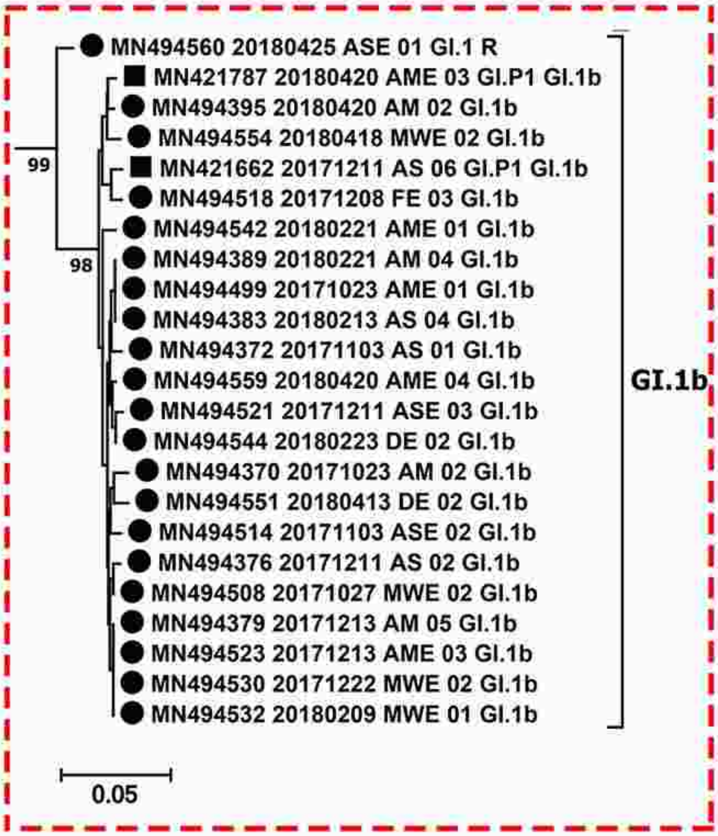

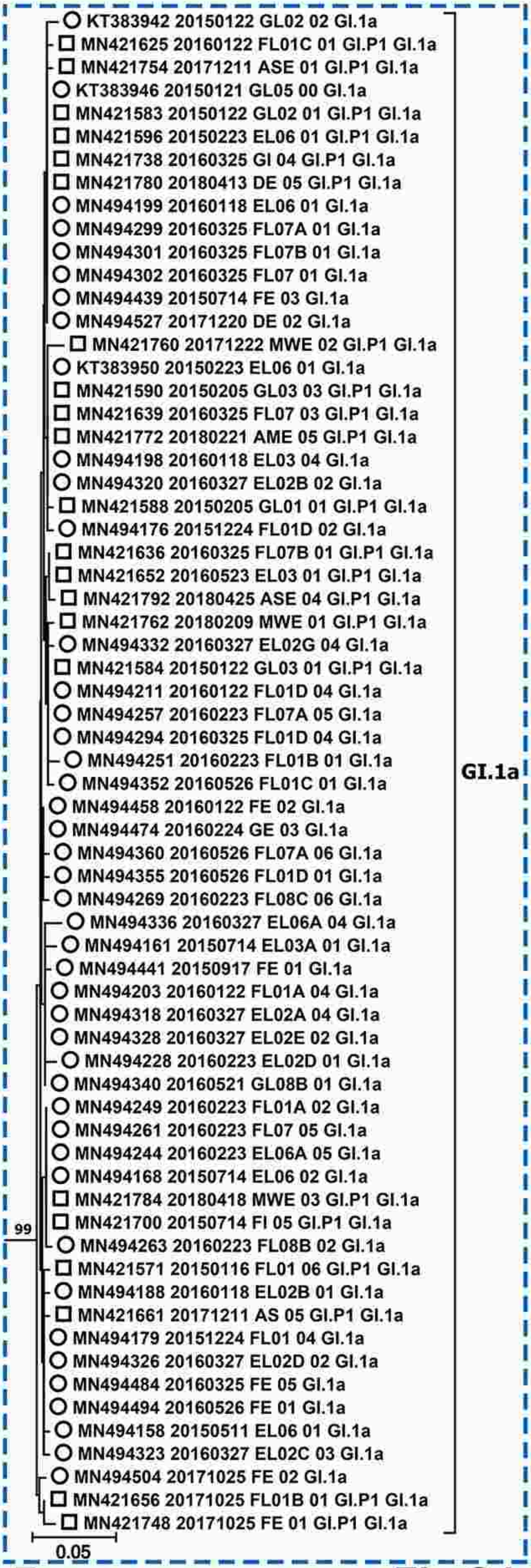

Fig S4

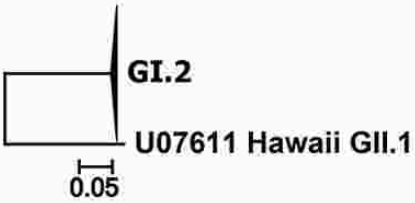

Marker	Amplicon type	Name of lineage
○	Region C	GI.2
△	VP1	GI.2

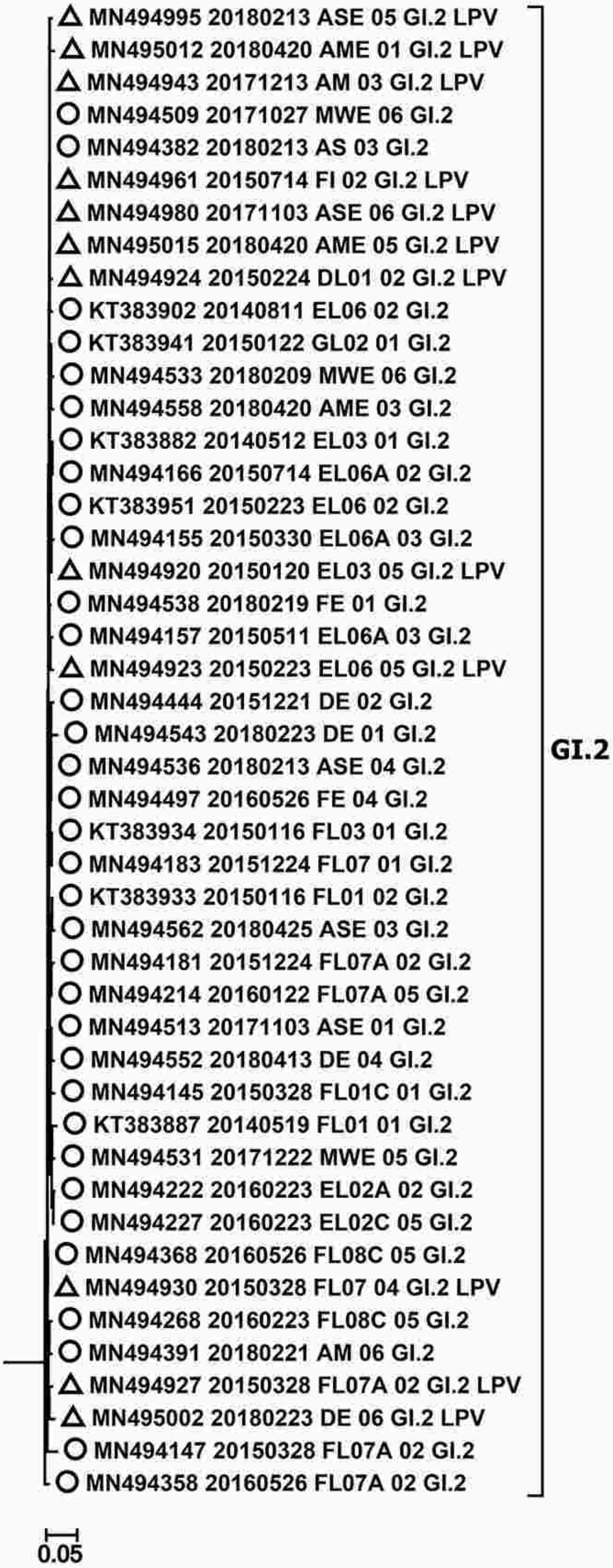

Fig S4

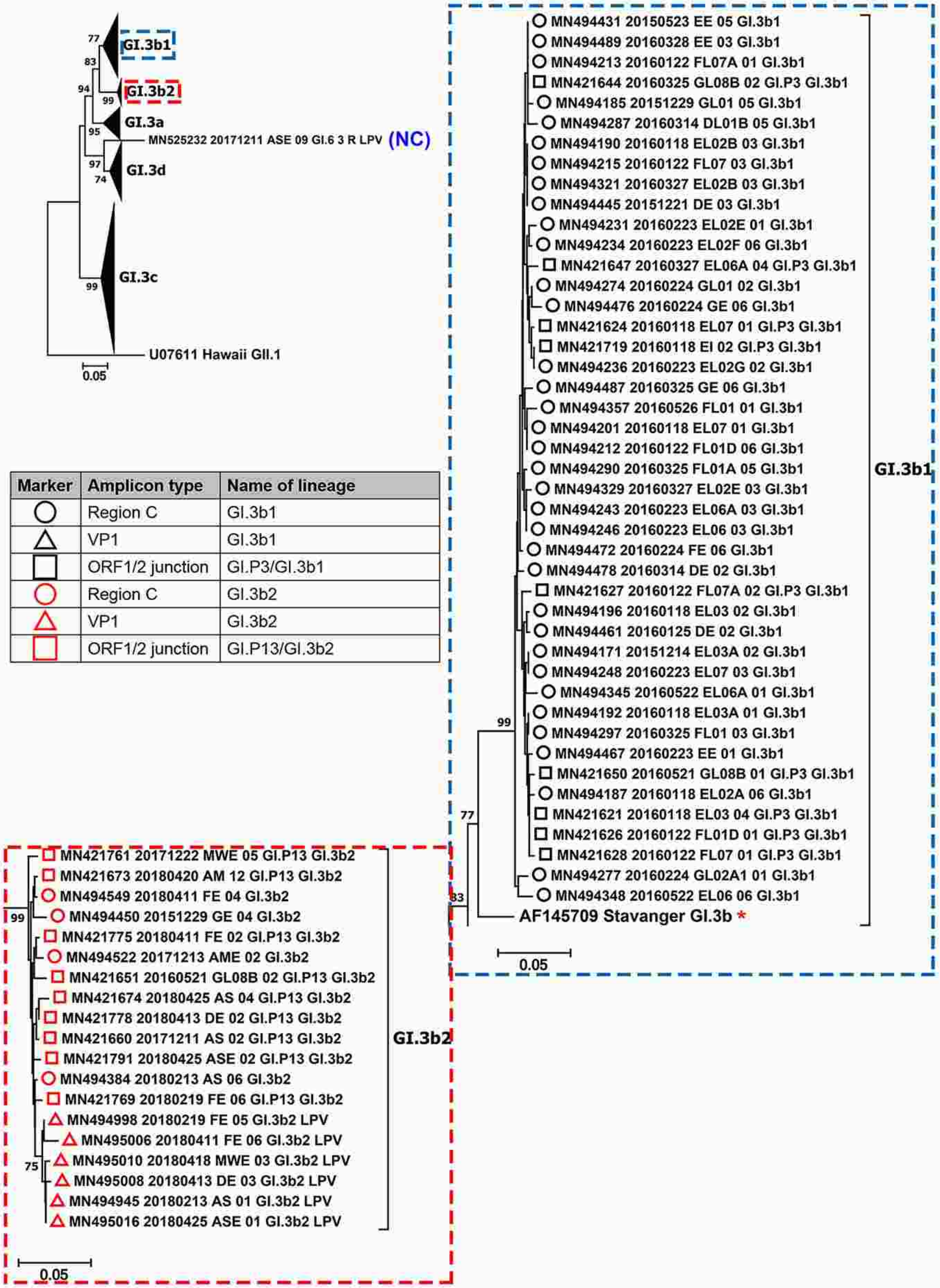

Marker	Amplicon type	Name of lineage
○	Region C	GI.3b1
△	VP1	GI.3b1
□	ORF1/2 junction	GI.P3/GI.3b1
○ (red)	Region C	GI.3b2
△ (red)	VP1	GI.3b2
□ (red)	ORF1/2 junction	GI.P13/GI.3b2

Fig S4

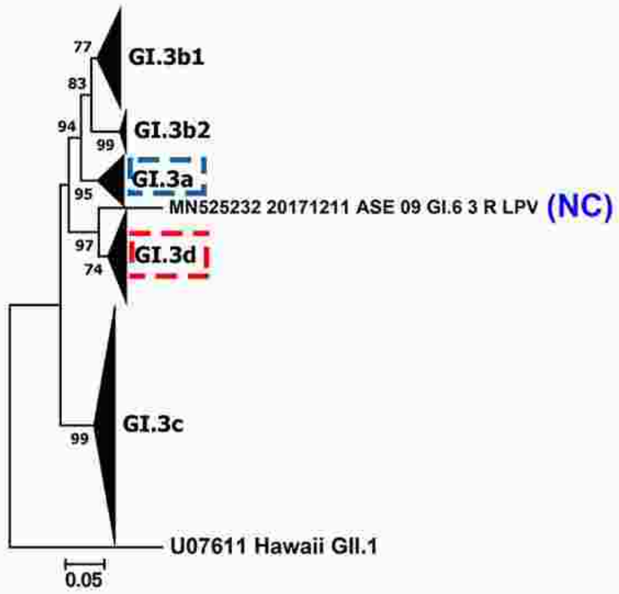

Marker	Amplicon type	Name of lineage
● (black)	Region C	GI.3a
▲ (black)	VP1	GI.3a
■ (black)	ORF1/2 junction	GI.P10/GI.3a
● (red)	Region C	GI.3d
▲ (red)	VP1	GI.3d
■ (red)	ORF1/2 junction	GI.P13/GI.3d, GI.P7/GI.3d

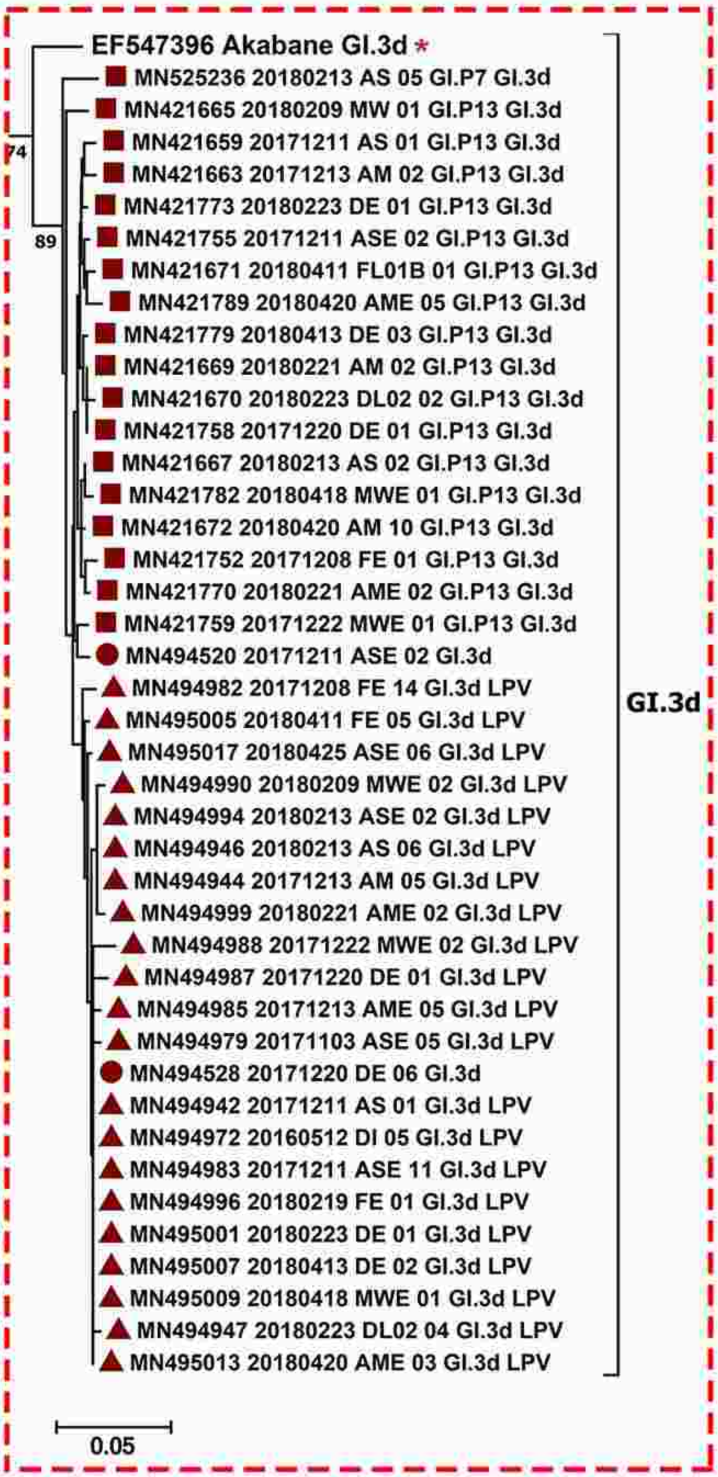

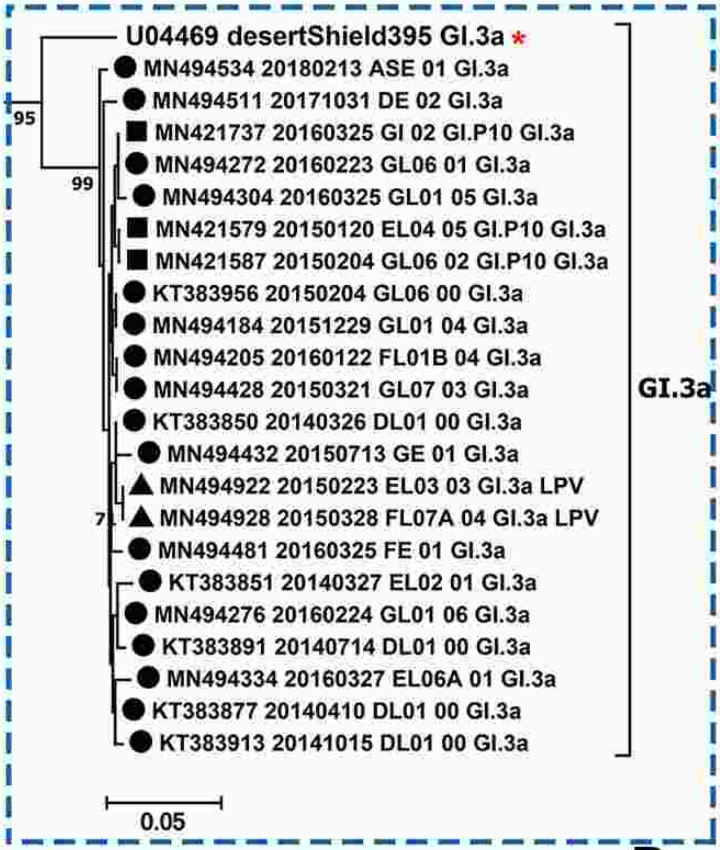

Fig S4

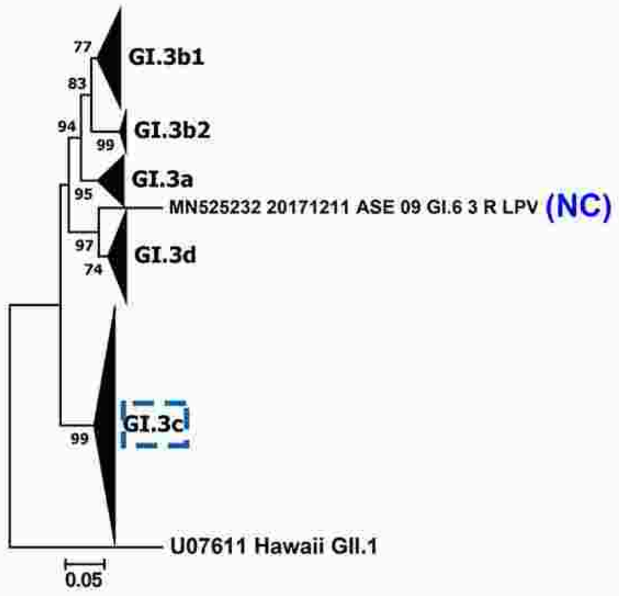

Marker	Amplicon type	Name of lineage
●	Region C	GI.3c
▲	VP1	GI.3c
■	ORF1/2 junction	GI.P3/GI.3c, GI.P14/GI.3c

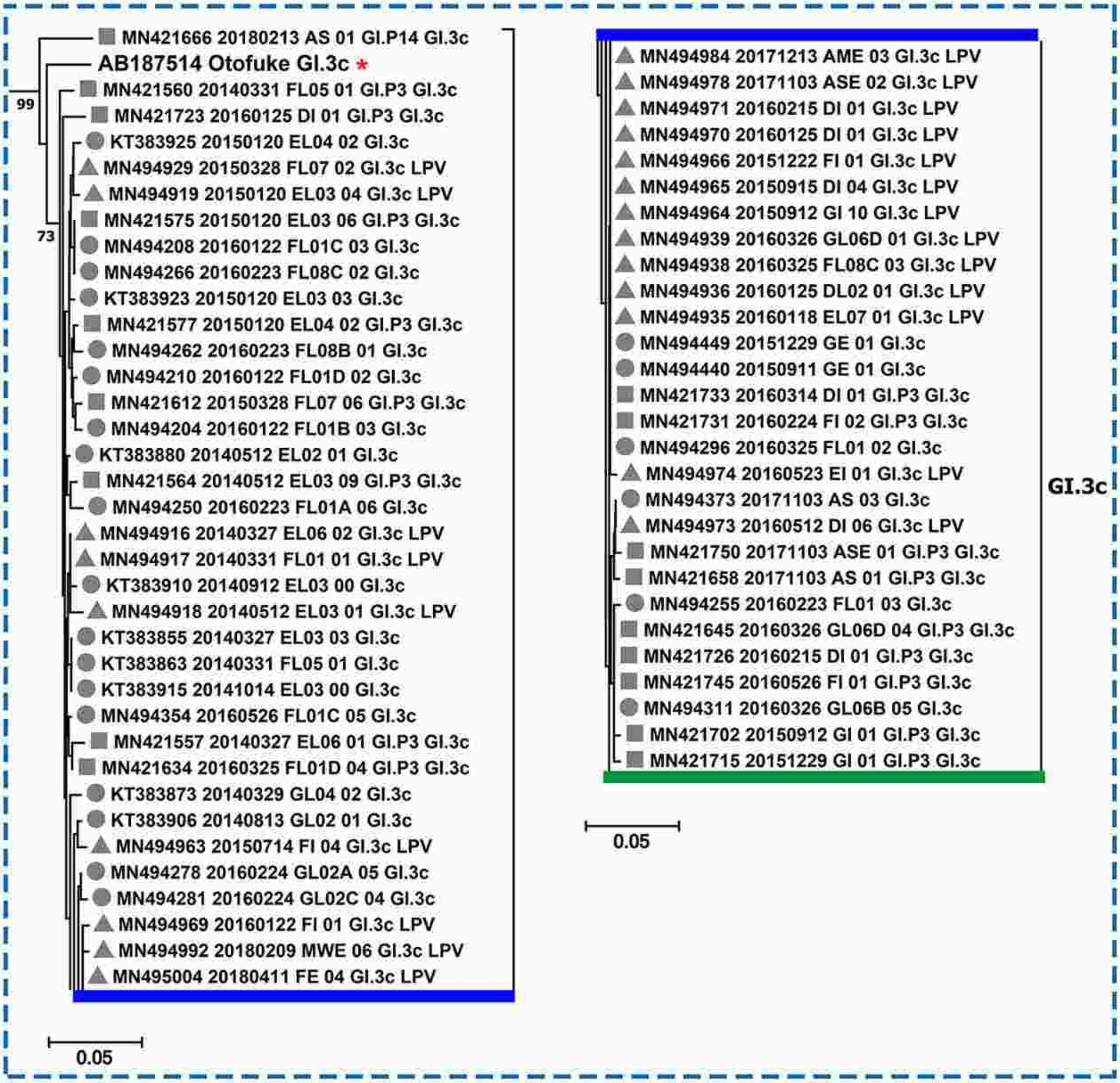

Fig S4

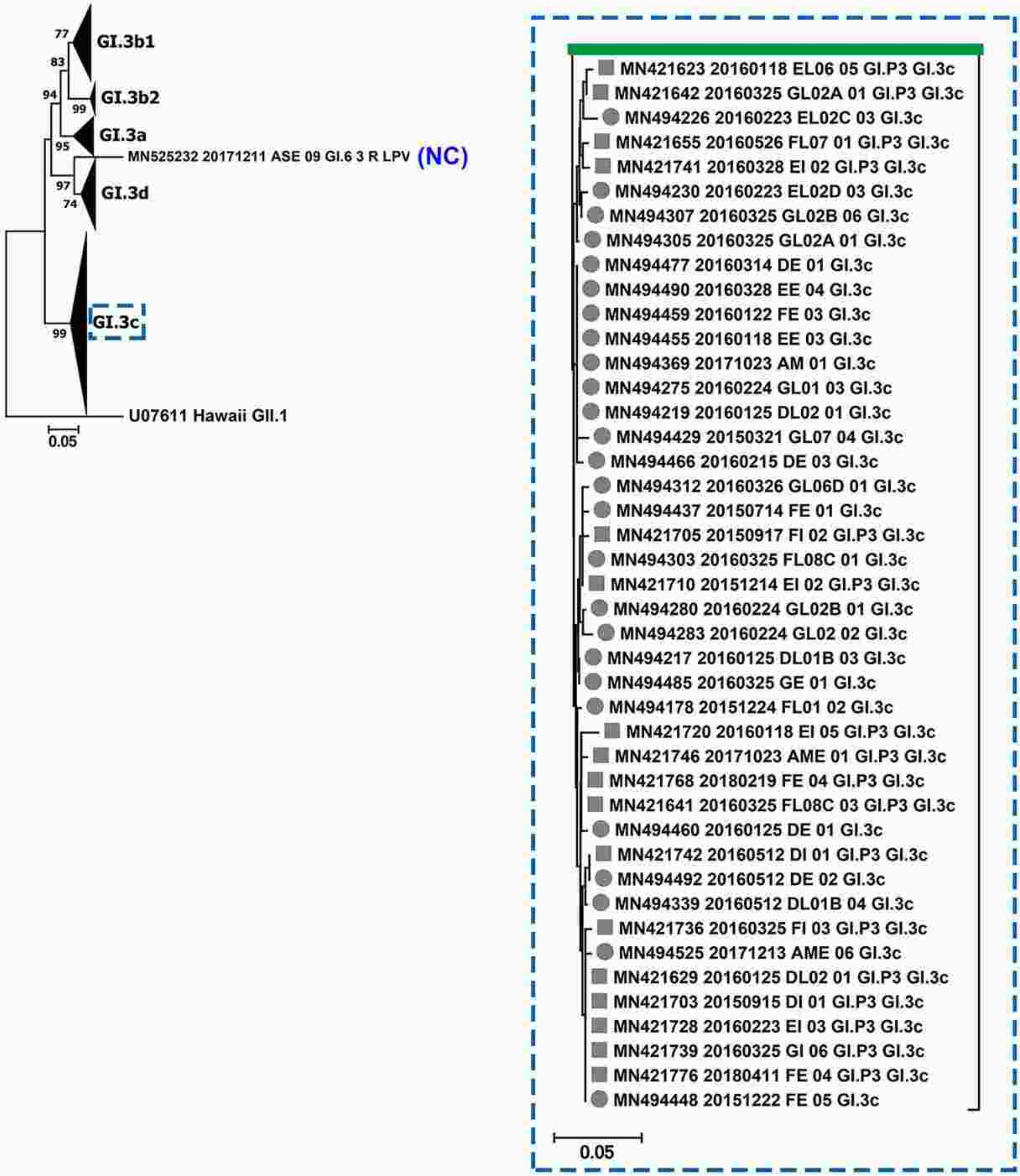

77
83
94
99
95
97
74
99
GI.3b1
GI.3b2
GI.3a
MN525232 20171211 ASE 09 GI.6 3 R LPV (NC)
GI.3d
GI.3c
U07611 Hawaii GII.1
0.05
MN421623 20160118 EL06 05 GI.P3 GI.3c
MN421642 20160325 GL02A 01 GI.P3 GI.3c
MN494226 20160223 EL02C 03 GI.3c
MN421655 20160526 FL07 01 GI.P3 GI.3c
MN421741 20160328 EI 02 GI.P3 GI.3c
MN494230 20160223 EL02D 03 GI.3c
MN494307 20160325 GL02B 06 GI.3c
MN494305 20160325 GL02A 01 GI.3c
MN494477 20160314 DE 01 GI.3c
MN494490 20160328 EE 04 GI.3c
MN494459 20160122 FE 03 GI.3c
MN494455 20160118 EE 03 GI.3c
MN494369 20171023 AM 01 GI.3c
MN494275 20160224 GL01 03 GI.3c
MN494219 20160125 DL02 01 GI.3c
MN494429 20150321 GL07 04 GI.3c
MN494466 20160215 DE 03 GI.3c
MN494312 20160326 GL06D 01 GI.3c
MN494437 20150714 FE 01 GI.3c
MN421705 20150917 FI 02 GI.P3 GI.3c
MN494303 20160325 FL08C 01 GI.3c
MN421710 20151214 EI 02 GI.P3 GI.3c
MN494280 20160224 GL02B 01 GI.3c
MN494283 20160224 GL02 02 GI.3c
MN494217 20160125 DL01B 03 GI.3c
MN494485 20160325 GE 01 GI.3c
MN494178 20151224 FL01 02 GI.3c
MN421720 20160118 EI 05 GI.P3 GI.3c
MN421746 20171023 AME 01 GI.P3 GI.3c
MN421768 20180219 FE 04 GI.P3 GI.3c
MN421641 20160325 FL08C 03 GI.P3 GI.3c
MN494460 20160125 DE 01 GI.3c
MN421742 20160512 DI 01 GI.P3 GI.3c
MN494492 20160512 DE 02 GI.3c
MN494339 20160512 DL01B 04 GI.3c
MN421736 20160325 FI 03 GI.P3 GI.3c
MN494525 20171213 AME 06 GI.3c
MN421629 20160125 DL02 01 GI.P3 GI.3c
MN421703 20150915 DI 01 GI.P3 GI.3c
MN421728 20160223 EI 03 GI.P3 GI.3c
MN421739 20160325 GI 06 GI.P3 GI.3c
MN421776 20180411 FE 04 GI.P3 GI.3c
MN494448 20151222 FE 05 GI.3c
0.05

Fig S4

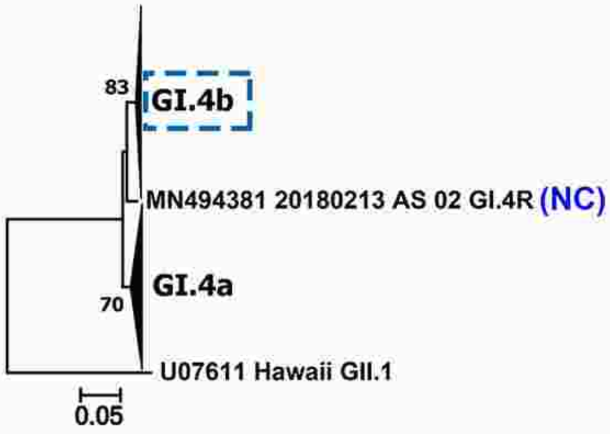

Marker	Amplicon type	Name of lineage
●	Region C	GI.4b
■	ORF1/2 junction	GI.P4/GI.4b

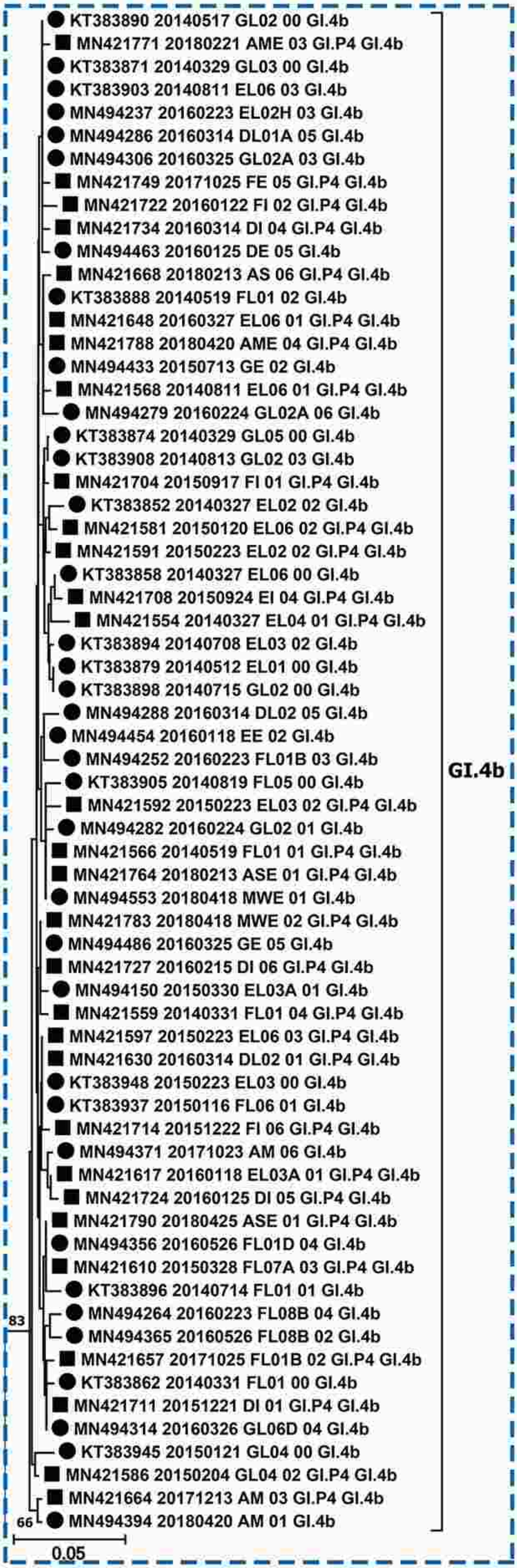

Fig S4

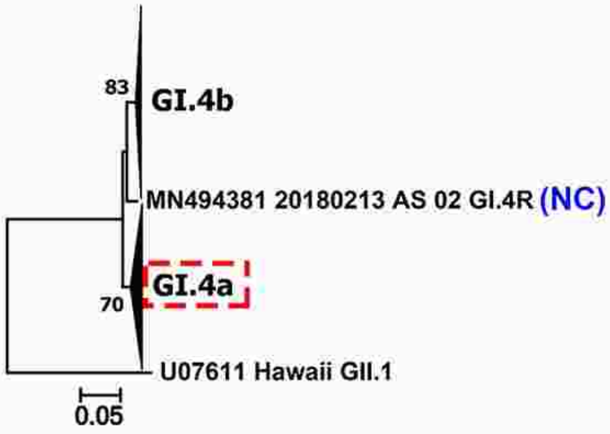

Marker	Amplicon type	Name of lineage
○	Region C	GI.4a
□	ORF1/2 junction	GI.P4/GI.4a

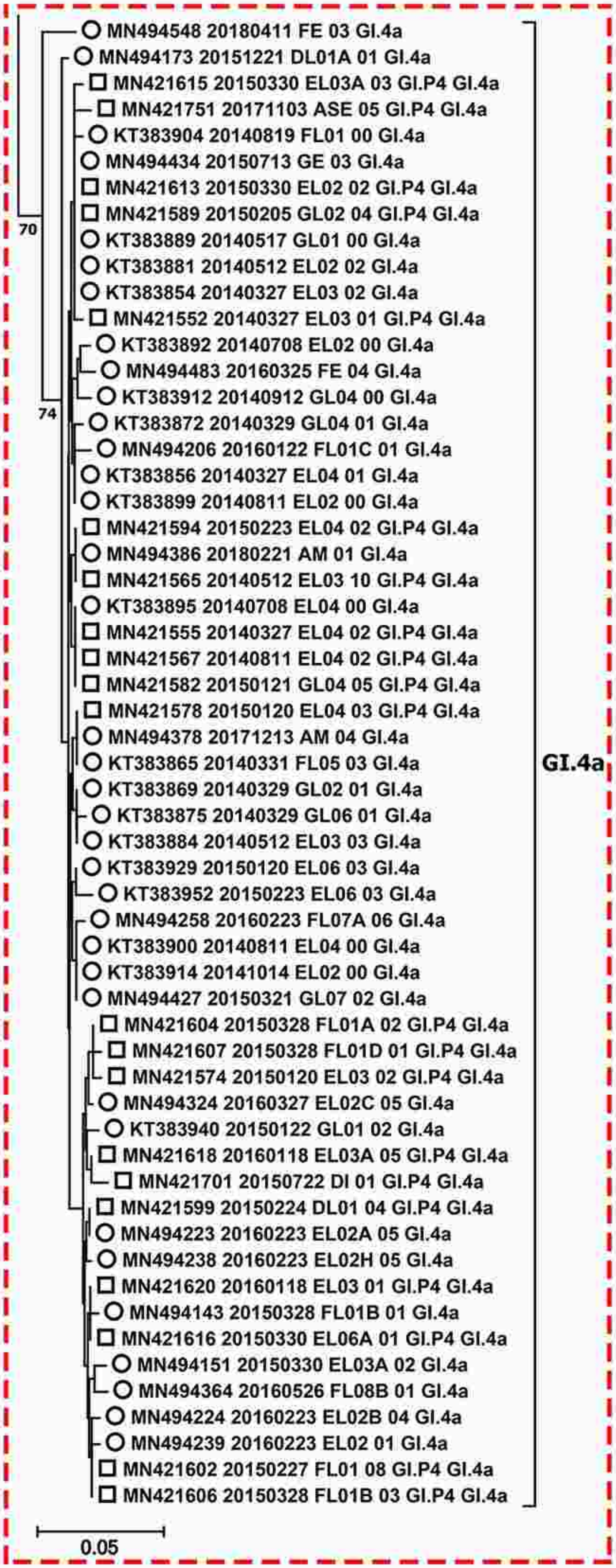

Fig S4

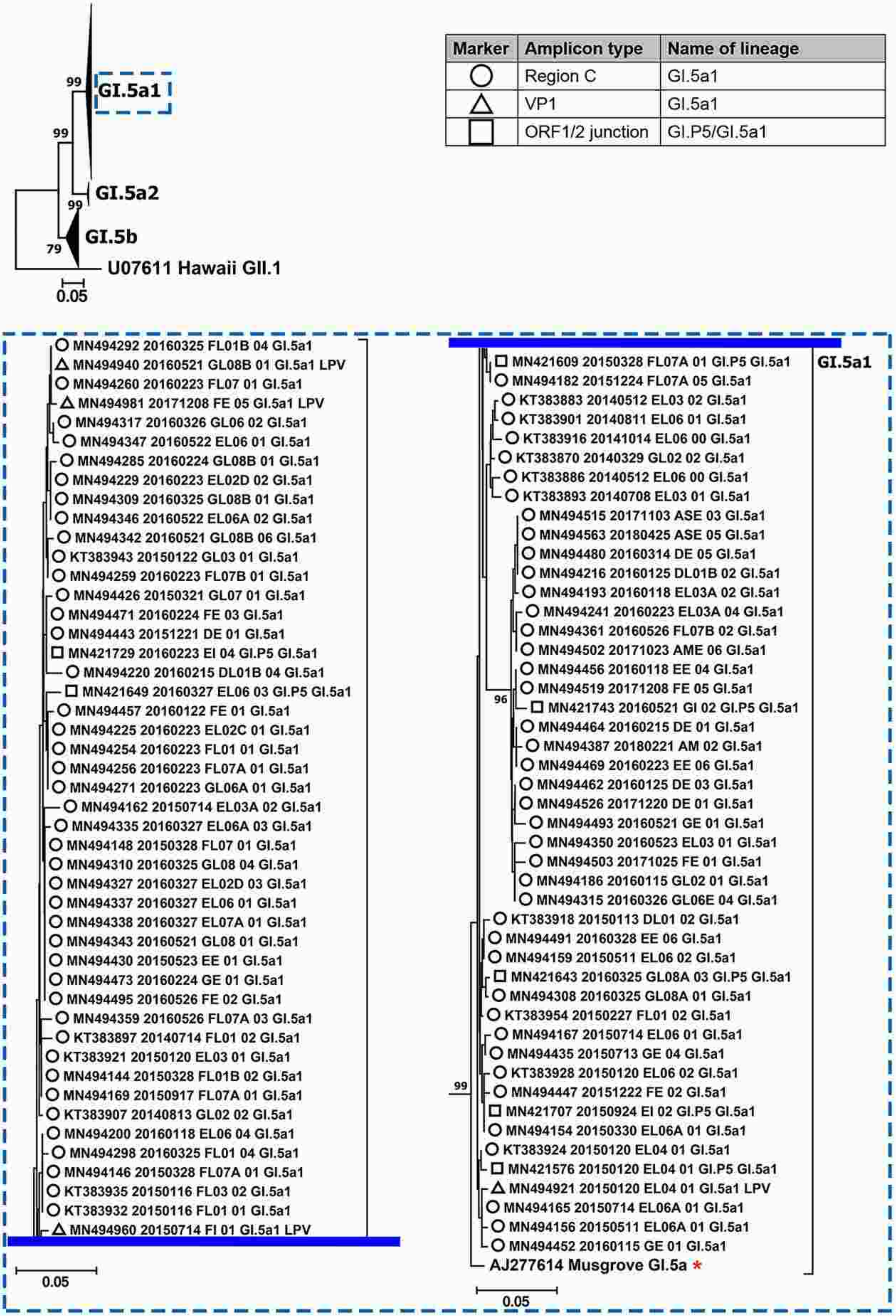

Marker | Amplicon type | Name of lineage
○ | Region C | GI.5a1
△ | VP1 | GI.5a1
□ | ORF1/2 junction | GI.P5/GI.5a1
99
GI.5a1
99
GI.5a2
99
GI.5b
79
U07611 Hawaii GII.1
0.05
MN494292 20160325 FL01B 04 GI.5a1
MN494940 20160521 GL08B 01 GI.5a1 LPV
MN494260 20160223 FL07 01 GI.5a1
MN494981 20171208 FE 05 GI.5a1 LPV
MN494317 20160326 GL06 02 GI.5a1
MN494347 20160522 EL06 01 GI.5a1
MN494285 20160224 GL08B 01 GI.5a1
MN494229 20160223 EL02D 02 GI.5a1
MN494309 20160325 GL08B 01 GI.5a1
MN494346 20160522 EL06A 02 GI.5a1
MN494342 20160521 GL08B 06 GI.5a1
KT383943 20150122 GL03 01 GI.5a1
MN494259 20160223 FL07B 01 GI.5a1
MN494426 20150321 GL07 01 GI.5a1
MN494471 20160224 FE 03 GI.5a1
MN494443 20151221 DE 01 GI.5a1
MN421729 20160223 EI 04 GI.P5 GI.5a1
MN494220 20160215 DL01B 04 GI.5a1
MN421649 20160327 EL06 03 GI.P5 GI.5a1
MN494457 20160122 FE 01 GI.5a1
MN494225 20160223 EL02C 01 GI.5a1
MN494254 20160223 FL01 01 GI.5a1
MN494256 20160223 FL07A 01 GI.5a1
MN494271 20160223 GL06A 01 GI.5a1
MN494162 20150714 EL03A 02 GI.5a1
MN494335 20160327 EL06A 03 GI.5a1
MN494148 20150328 FL07 01 GI.5a1
MN494310 20160325 GL08 04 GI.5a1
MN494327 20160327 EL02D 03 GI.5a1
MN494337 20160327 EL06 01 GI.5a1
MN494338 20160327 EL07A 01 GI.5a1
MN494343 20160521 GL08 01 GI.5a1
MN494430 20150523 EE 01 GI.5a1
MN494473 20160224 GE 01 GI.5a1
MN494495 20160526 FE 02 GI.5a1
MN494359 20160526 FL07A 03 GI.5a1
KT383897 20140714 FL01 02 GI.5a1
KT383921 20150120 EL03 01 GI.5a1
MN494144 20150328 FL01B 02 GI.5a1
MN494169 20150917 FL07A 01 GI.5a1
KT383907 20140813 GL02 02 GI.5a1
MN494200 20160118 EL06 04 GI.5a1
MN494298 20160325 FL01 04 GI.5a1
MN494146 20150328 FL07A 01 GI.5a1
KT383935 20150116 FL03 02 GI.5a1
KT383932 20150116 FL01 01 GI.5a1
MN494960 20150714 FI 01 GI.5a1 LPV
0.05
MN421609 20150328 FL07A 01 GI.P5 GI.5a1
GI.5a1
MN494182 20151224 FL07A 05 GI.5a1
KT383883 20140512 EL03 02 GI.5a1
KT383901 20140811 EL06 01 GI.5a1
KT383916 20141014 EL06 00 GI.5a1
KT383870 20140329 GL02 02 GI.5a1
KT383886 20140512 EL06 00 GI.5a1
KT383893 20140708 EL03 01 GI.5a1
MN494515 20171103 ASE 03 GI.5a1
MN494563 20180425 ASE 05 GI.5a1
MN494480 20160314 DE 05 GI.5a1
MN494216 20160125 DL01B 02 GI.5a1
MN494193 20160118 EL03A 02 GI.5a1
MN494241 20160223 EL03A 04 GI.5a1
MN494361 20160526 FL07B 02 GI.5a1
MN494502 20171023 AME 06 GI.5a1
MN494456 20160118 EE 04 GI.5a1
MN494519 20171208 FE 05 GI.5a1
96
MN421743 20160521 GI 02 GI.P5 GI.5a1
MN494464 20160215 DE 01 GI.5a1
MN494387 20180221 AM 02 GI.5a1
MN494469 20160223 EE 06 GI.5a1
MN494462 20160125 DE 03 GI.5a1
MN494526 20171220 DE 01 GI.5a1
MN494493 20160521 GE 01 GI.5a1
MN494350 20160523 EL03 01 GI.5a1
MN494503 20171025 FE 01 GI.5a1
MN494186 20160115 GL02 01 GI.5a1
MN494315 20160326 GL06E 04 GI.5a1
KT383918 20150113 DL01 02 GI.5a1
MN494491 20160328 EE 06 GI.5a1
MN494159 20150511 EL06 02 GI.5a1
MN421643 20160325 GL08A 03 GI.P5 GI.5a1
MN494308 20160325 GL08A 01 GI.5a1
KT383954 20150227 FL01 02 GI.5a1
MN494167 20150714 EL06 01 GI.5a1
MN494435 20150713 GE 04 GI.5a1
KT383928 20150120 EL06 02 GI.5a1
99
MN494447 20151222 FE 02 GI.5a1
MN421707 20150924 EI 02 GI.P5 GI.5a1
MN494154 20150330 EL06A 01 GI.5a1
KT383924 20150120 EL04 01 GI.5a1
MN421576 20150120 EL04 01 GI.P5 GI.5a1
MN494921 20150120 EL04 01 GI.5a1 LPV
MN494165 20150714 EL06A 01 GI.5a1
MN494156 20150511 EL06A 01 GI.5a1
MN494452 20160115 GE 01 GI.5a1
AJ277614 Musgrove GI.5a *
0.05

Fig S4

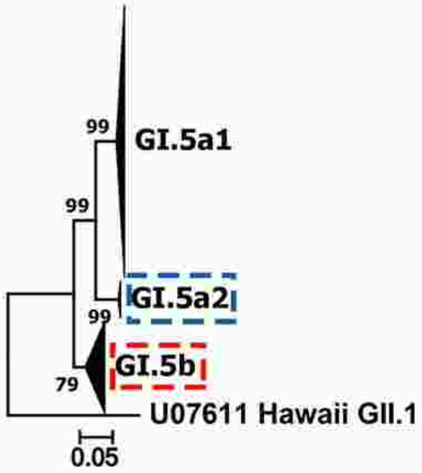

Marker	Amplicon type	Name of lineage
○	Region C	GI.5a2
△	VP1	GI.5a2
□	ORF1/2 junction	GI.P4/GI.5a2
●	Region C	GI.5b
▲	VP1	GI.5b
■	ORF1/2 junction	GI.P12/GI.5b

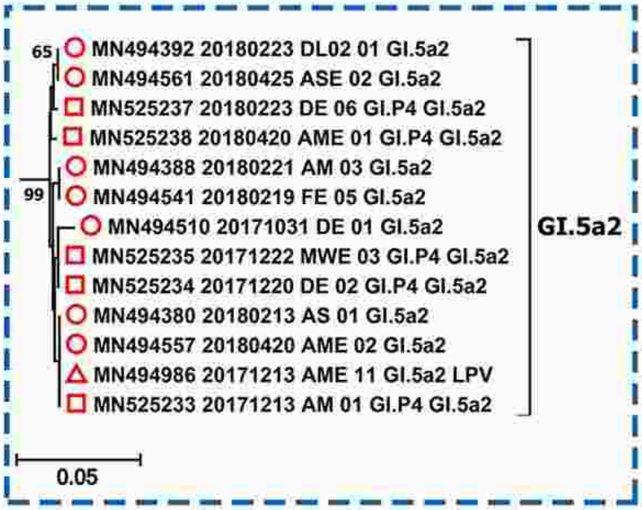

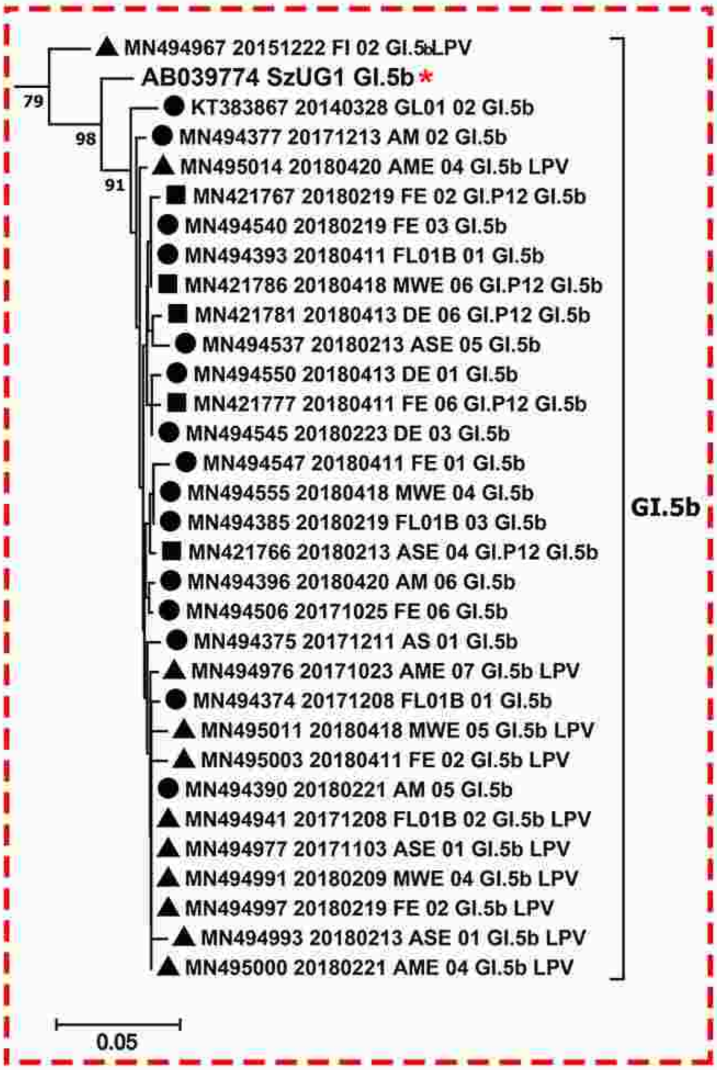

Fig S4

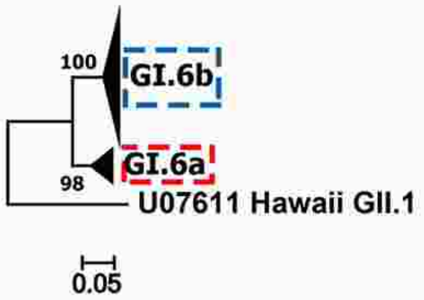

Marker	Amplicon type	Name of lineage
○	Region C	GI.6a
△	VP1	GI.6a
□	ORF1/2 junction	GI.P11/GI.6a
●	Region C	GI.6b
▲	VP1	GI.6b
■	ORF1/2 junction	GI.P6/GI.6b

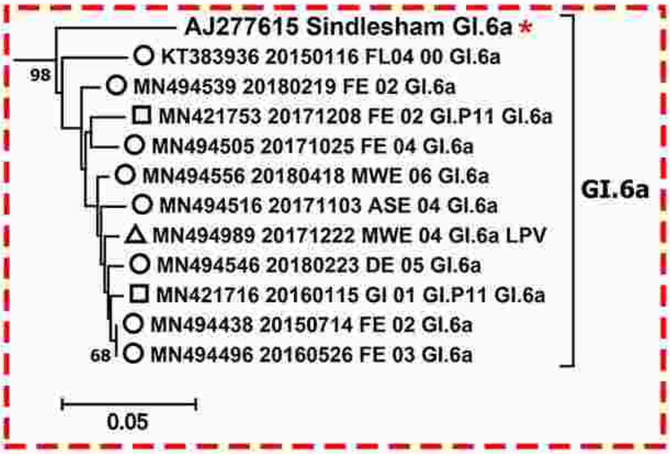

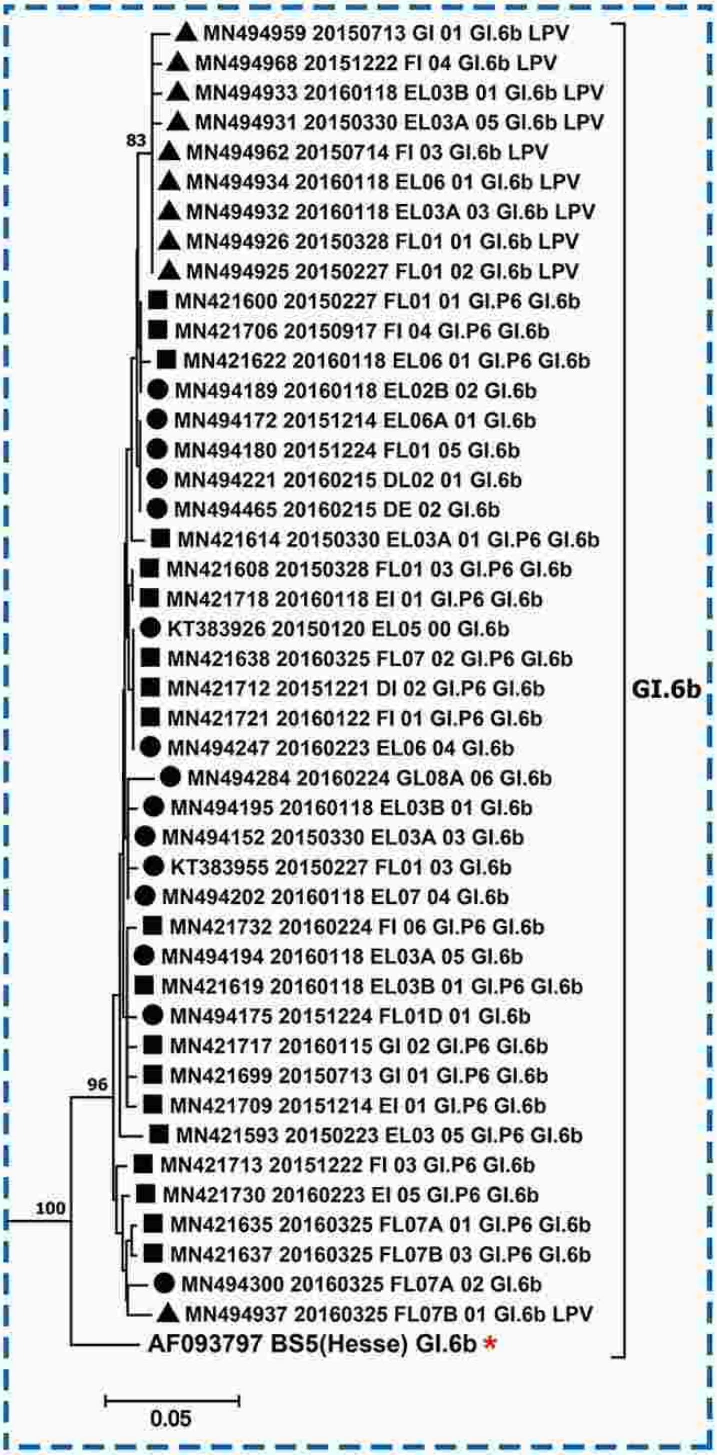

Fig S4

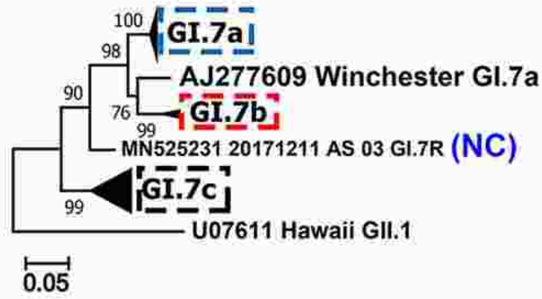

Marker	Amplicon type	Name of lineage
○	Region C	GI.7a
△	VP1	GI.7a
□	ORF1/2 junction	GI.P7/GI.7a
●	Region C	GI.7b
● (grey)	Region C	GI.7c
■ (grey)	ORF1/2 junction	GI.P7/GI.7c

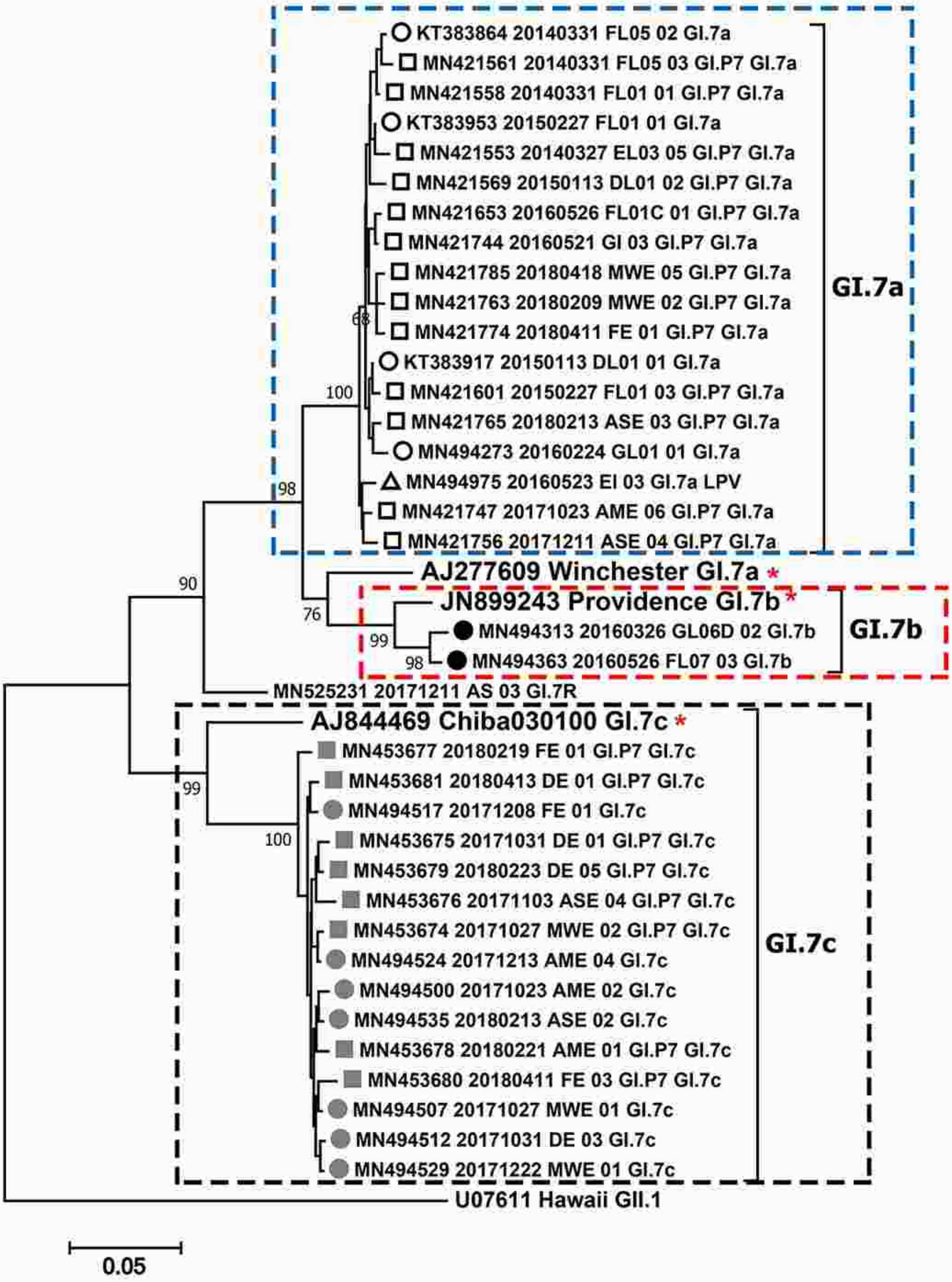

Fig S4

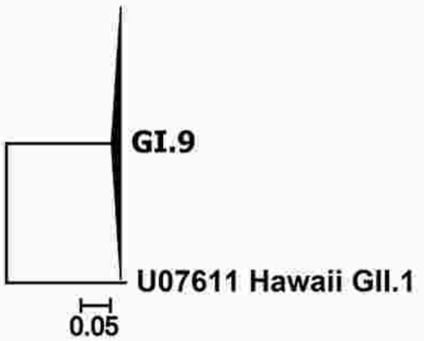

Marker	Amplicon type	Name of lineage
○	Region C	GI.9
□	ORF1/2 junction	GI.P9/GI.9

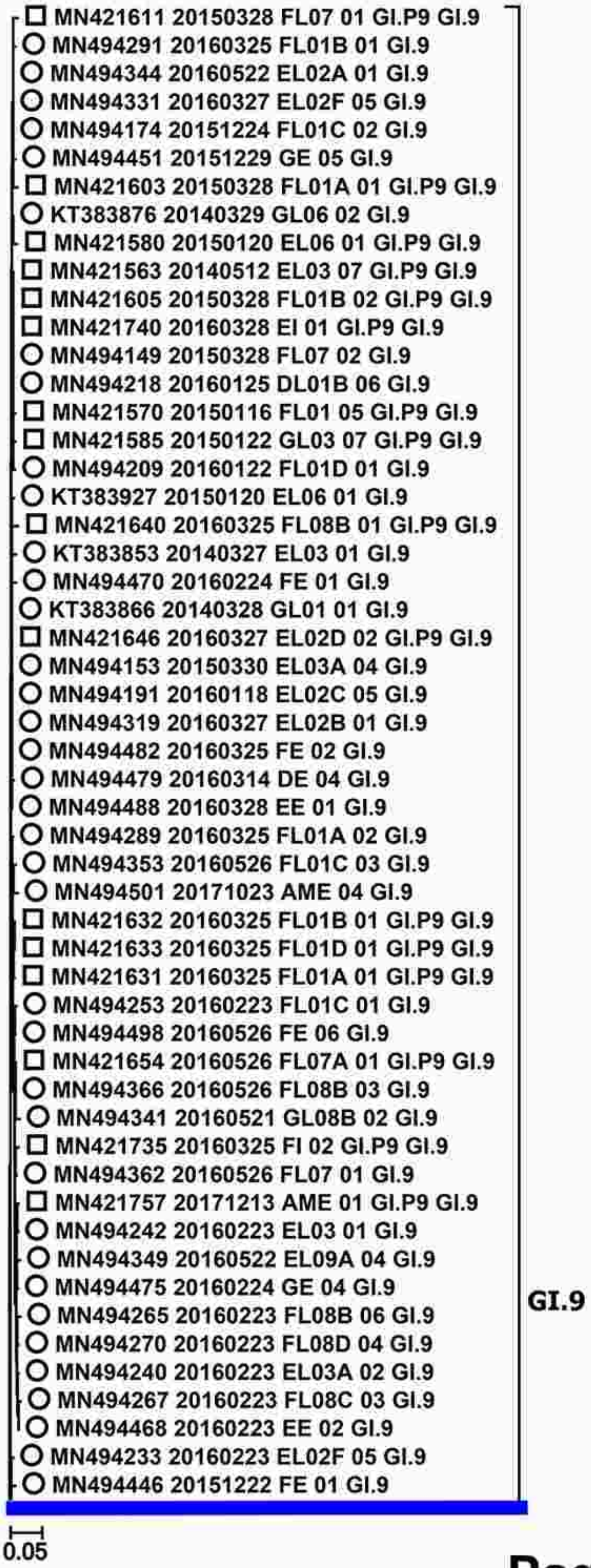

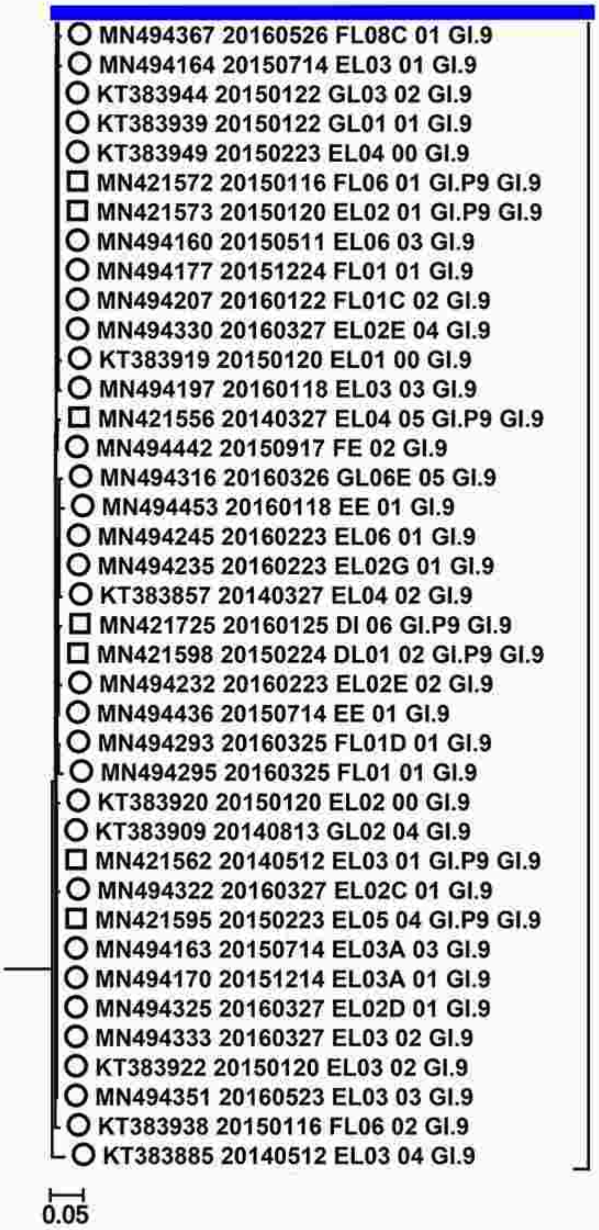

Fig S4

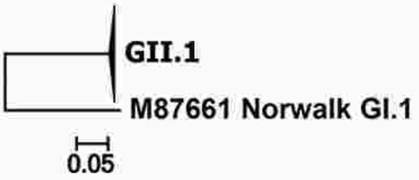

Marker	Amplicon type	Name of lineage
○	Region C	GII.1
△	VP1	GII.1
□	ORF1/2 junction	GII.P33/GII.1

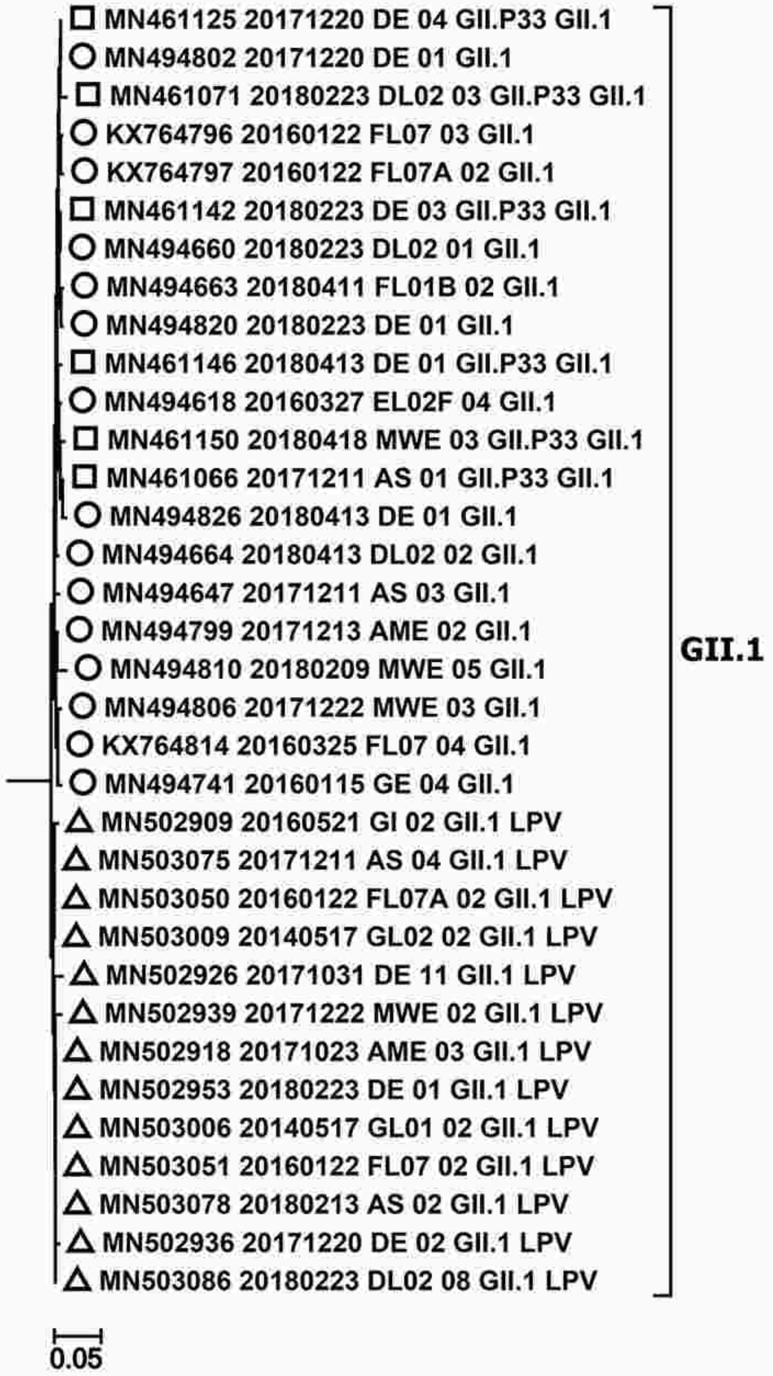

Fig S4

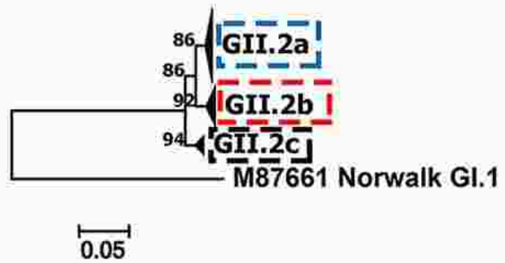

Marker	Amplicon type	Name of lineage
○	Region C	GII.2a
△	VP1	GII.2a
□	ORF1/2 junction	GI.P16/GII.2a
●	Region C	GII.2b
▲	VP1	GII.2b
■	ORF1/2 junction	GII.P16/GII.2b
● (grey)	Region C	GII.2c
■ (grey)	ORF1/2 junction	GII.P2/GII.2c

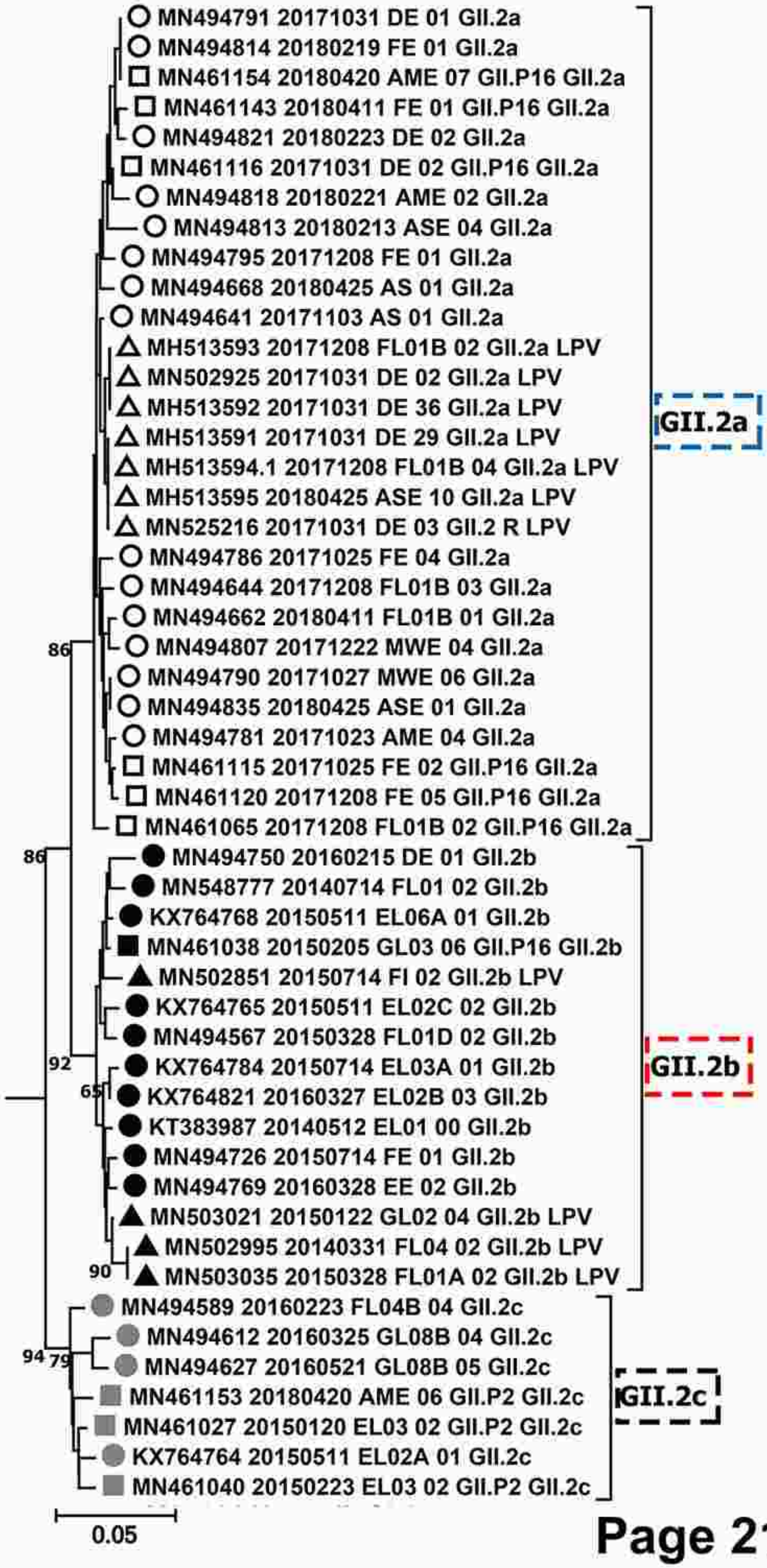

Fig S4

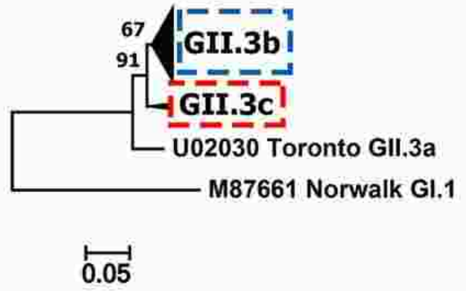

Marker	Amplicon type	Name of lineage
○	Region C	GII.3b
△	VP1	GII.3b
□	ORF1/2 junction	GII.P12/GII.3b
●	Region C	GII.3c

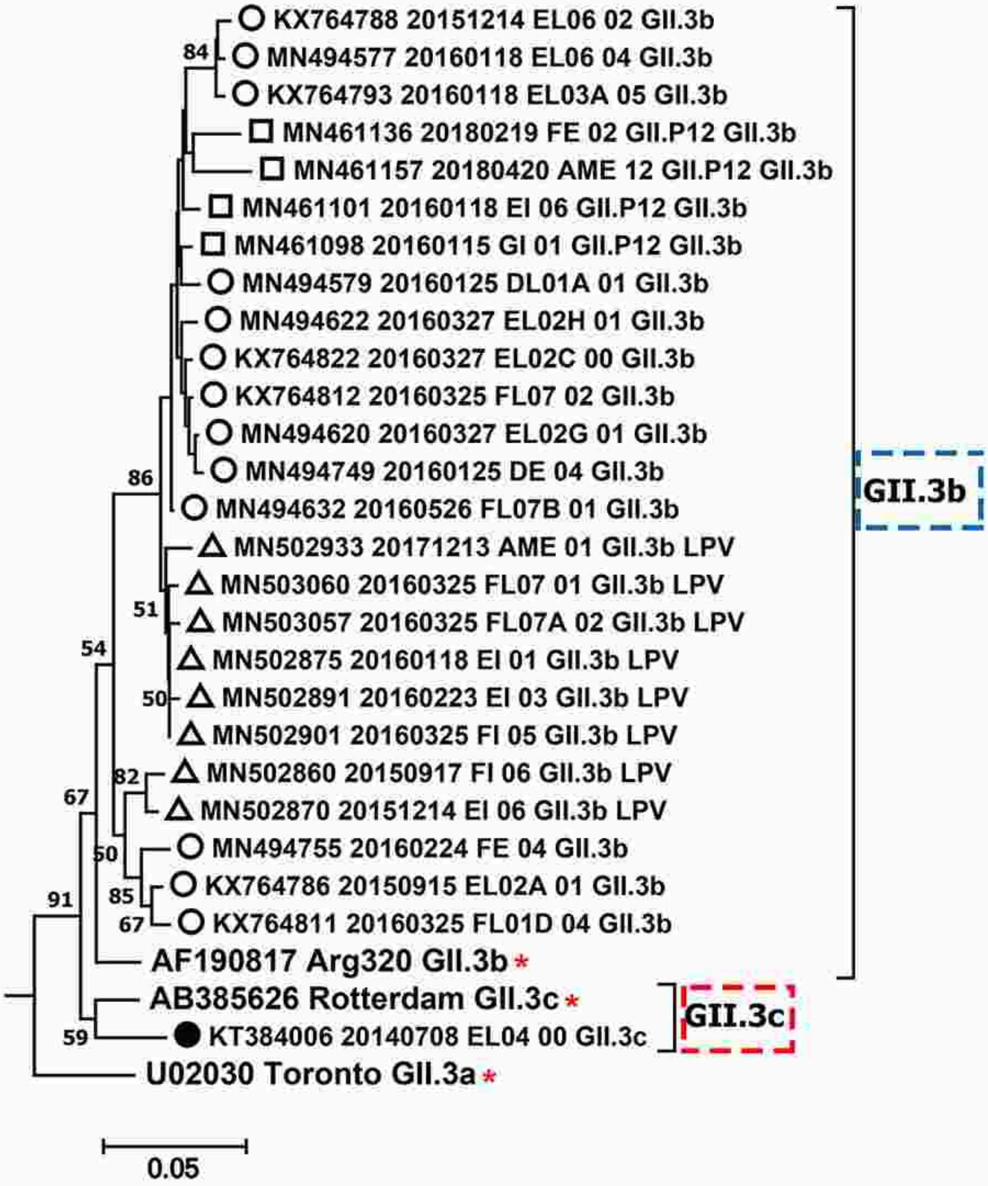

Fig S4

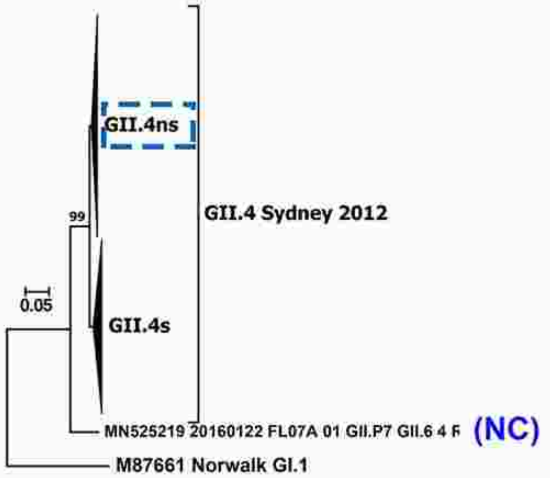

Marker	Amplicon type	Name of lineage
○	Region C	GII.4ns
△	VP1	GII.4ns
□	ORF1/2 junction	GII.P16/GII.4ns

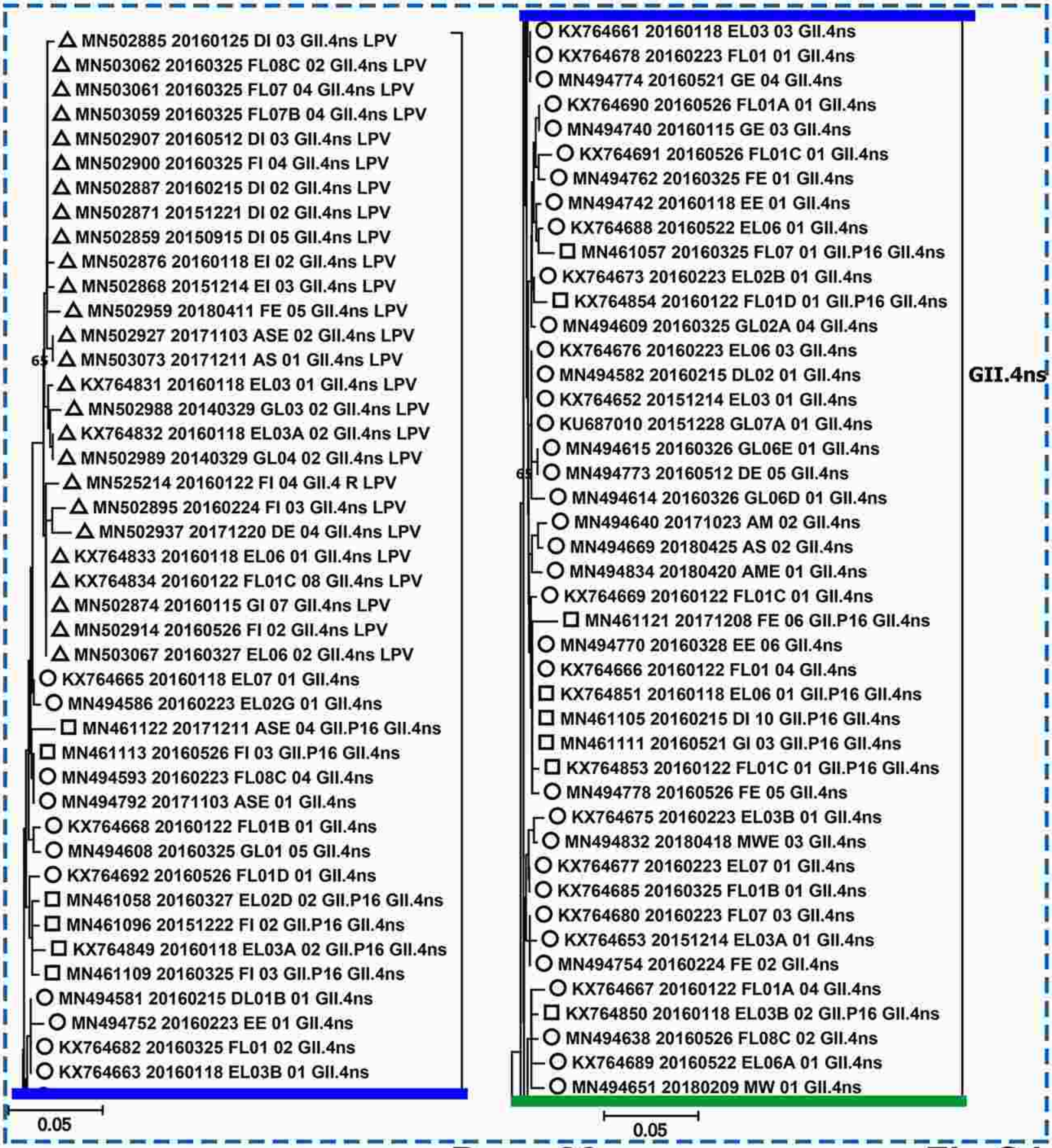

Fig S4

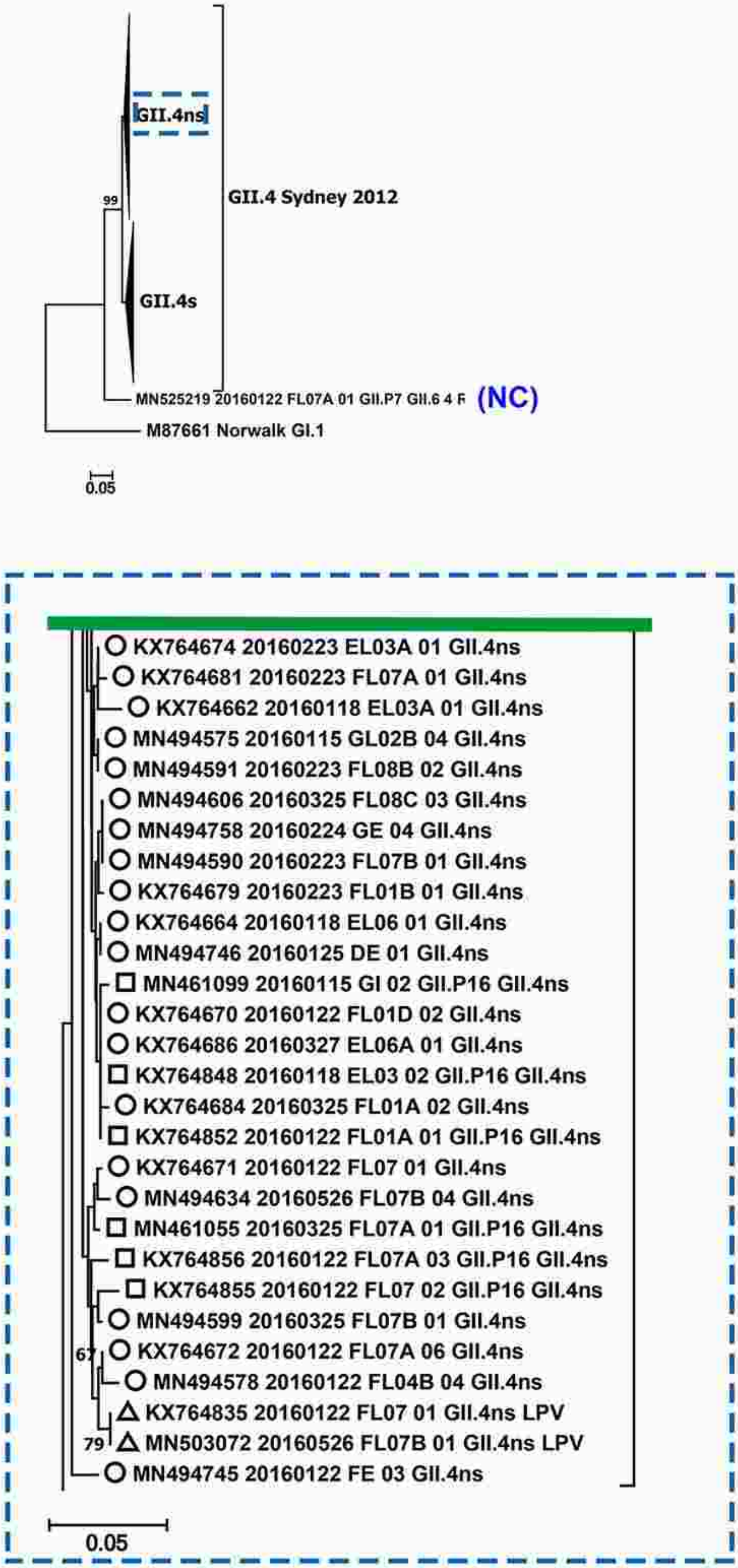
GII.4ns
99
GII.4 Sydney 2012
GII.4s
MN525219 20160122 FL07A 01 GII.P7 GII.6 4 F
(NC)
M87661 Norwalk GI.1
0.05
KX764674 20160223 EL03A 01 GII.4ns
KX764681 20160223 FL07A 01 GII.4ns
KX764662 20160118 EL03A 01 GII.4ns
MN494575 20160115 GL02B 04 GII.4ns
MN494591 20160223 FL08B 02 GII.4ns
MN494606 20160325 FL08C 03 GII.4ns
MN494758 20160224 GE 04 GII.4ns
MN494590 20160223 FL07B 01 GII.4ns
KX764679 20160223 FL01B 01 GII.4ns
KX764664 20160118 EL06 01 GII.4ns
MN494746 20160125 DE 01 GII.4ns
MN461099 20160115 GI 02 GII.P16 GII.4ns
KX764670 20160122 FL01D 02 GII.4ns
KX764686 20160327 EL06A 01 GII.4ns
KX764848 20160118 EL03 02 GII.P16 GII.4ns
KX764684 20160325 FL01A 02 GII.4ns
KX764852 20160122 FL01A 01 GII.P16 GII.4ns
KX764671 20160122 FL07 01 GII.4ns
MN494634 20160526 FL07B 04 GII.4ns
MN461055 20160325 FL07A 01 GII.P16 GII.4ns
KX764856 20160122 FL07A 03 GII.P16 GII.4ns
KX764855 20160122 FL07 02 GII.P16 GII.4ns
MN494599 20160325 FL07B 01 GII.4ns
67
KX764672 20160122 FL07A 06 GII.4ns
MN494578 20160122 FL04B 04 GII.4ns
KX764835 20160122 FL07 01 GII.4ns LPV
79
MN503072 20160526 FL07B 01 GII.4ns LPV
MN494745 20160122 FE 03 GII.4ns
0.05

Fig S4

Marker	Amplicon type	Name of lineage
●	Region C	GII.4s
▲	VP1	GII.4s
■	ORF1/2 junction	GII.P31/GII.4s

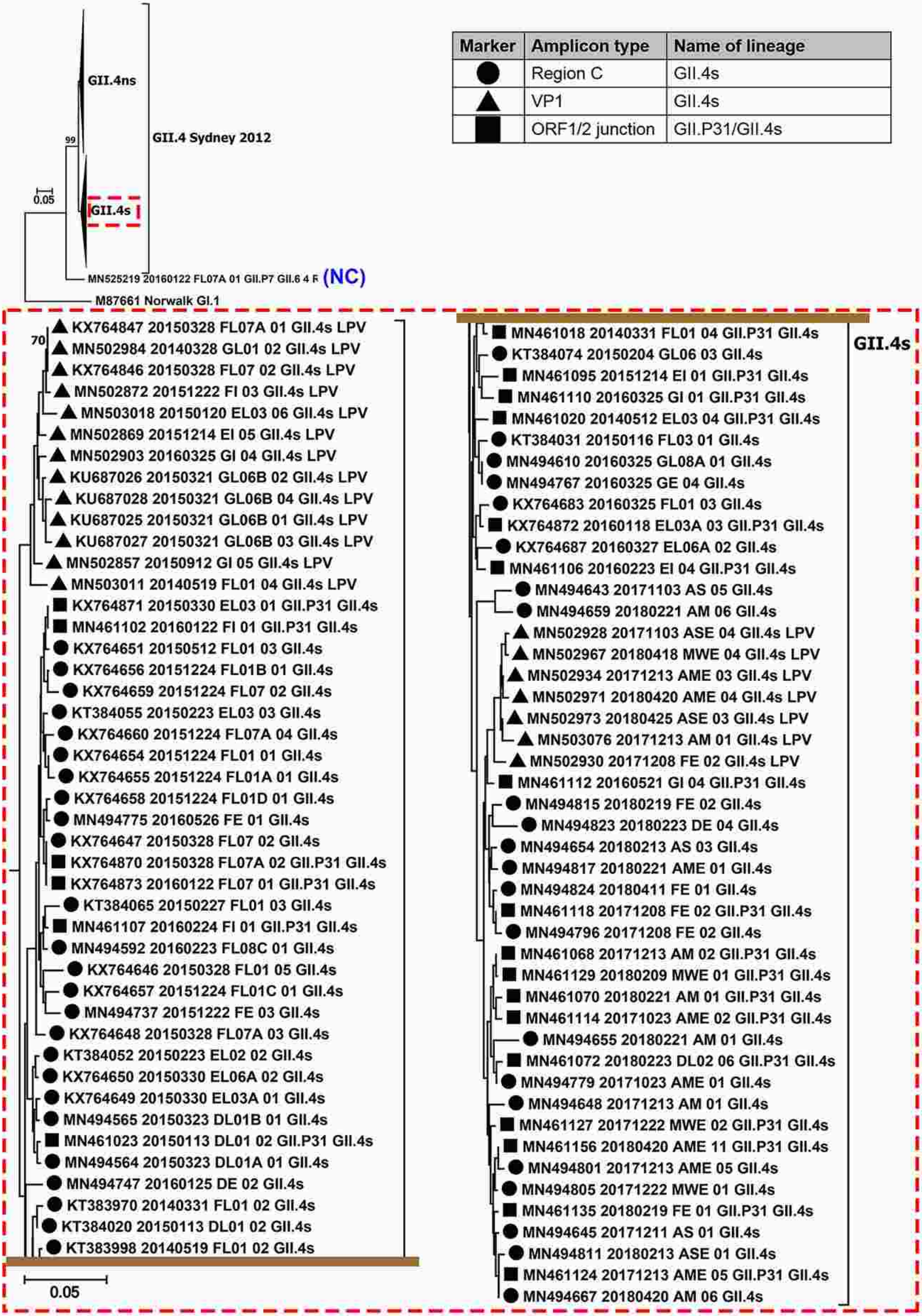

Fig S4

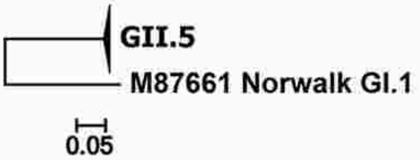

Marker	Amplicon type	Name of lineage
○	Region C	GII.5
△	VP1	GII.5
□	ORF1/2 junction	GII.P40/GII.5

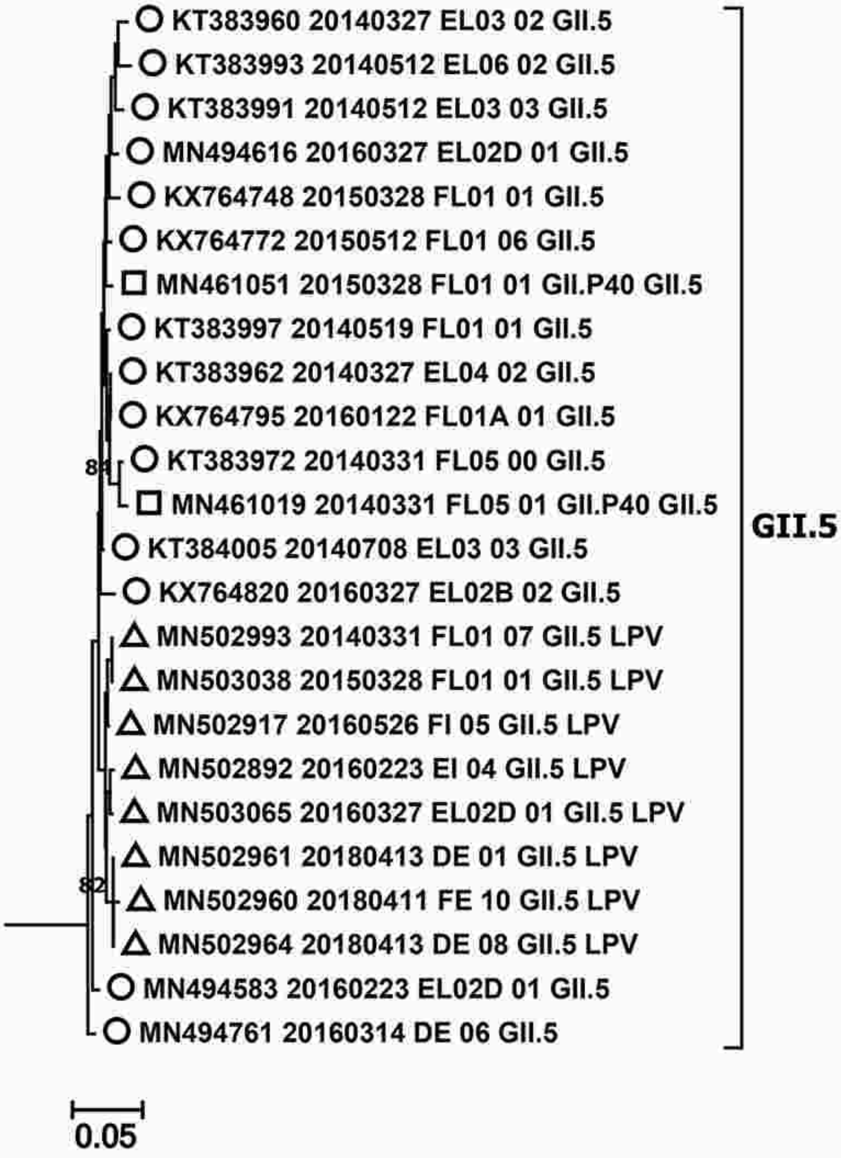

Fig S4

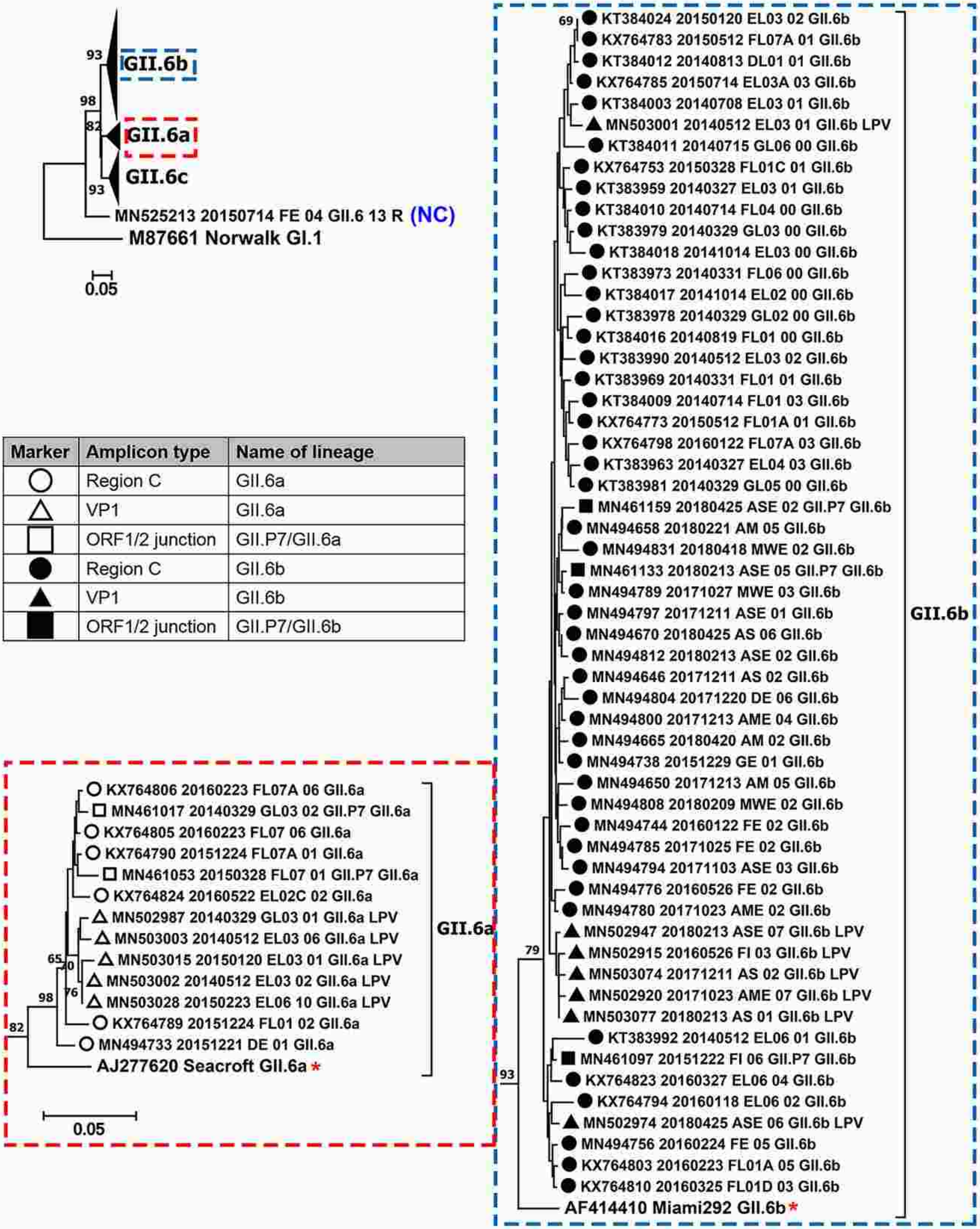

Marker	Amplicon type	Name of lineage
○	Region C	GII.6a
△	VP1	GII.6a
□	ORF1/2 junction	GII.P7/GII.6a
●	Region C	GII.6b
▲	VP1	GII.6b
■	ORF1/2 junction	GII.P7/GII.6b

Fig S4

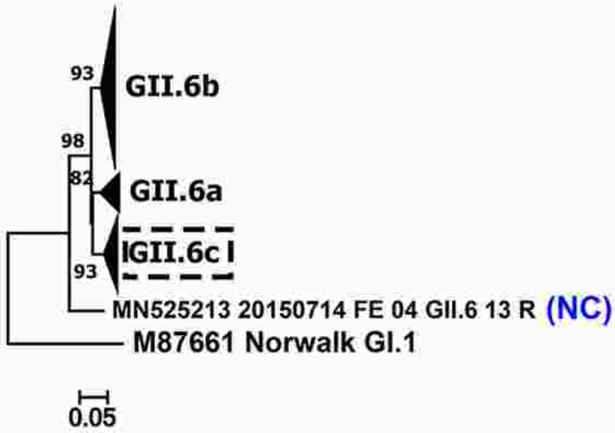

Marker	Amplicon type	Name of lineage
●	Region C	GII.6c
▲	VP1	GII.6c
■	ORF1/2 junction	GII.P7/GII.6c

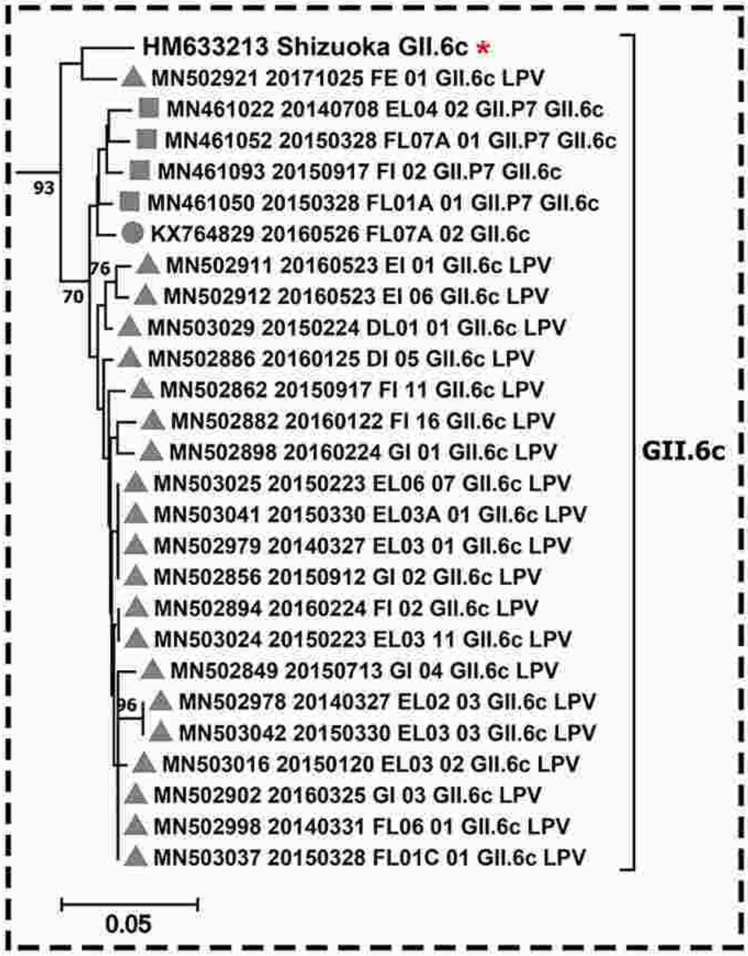

Fig S4

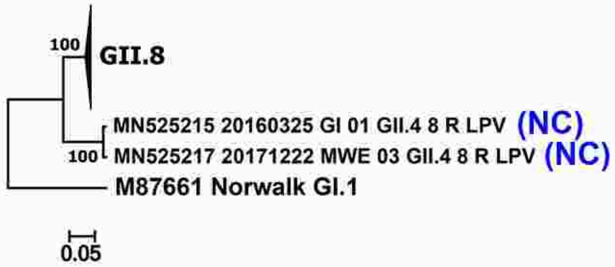

Marker	Amplicon type	Name of lineage
○	Region C	GII.8
△	VP1	GII.8
□	ORF1/2 junction	GII.P8/GII.8

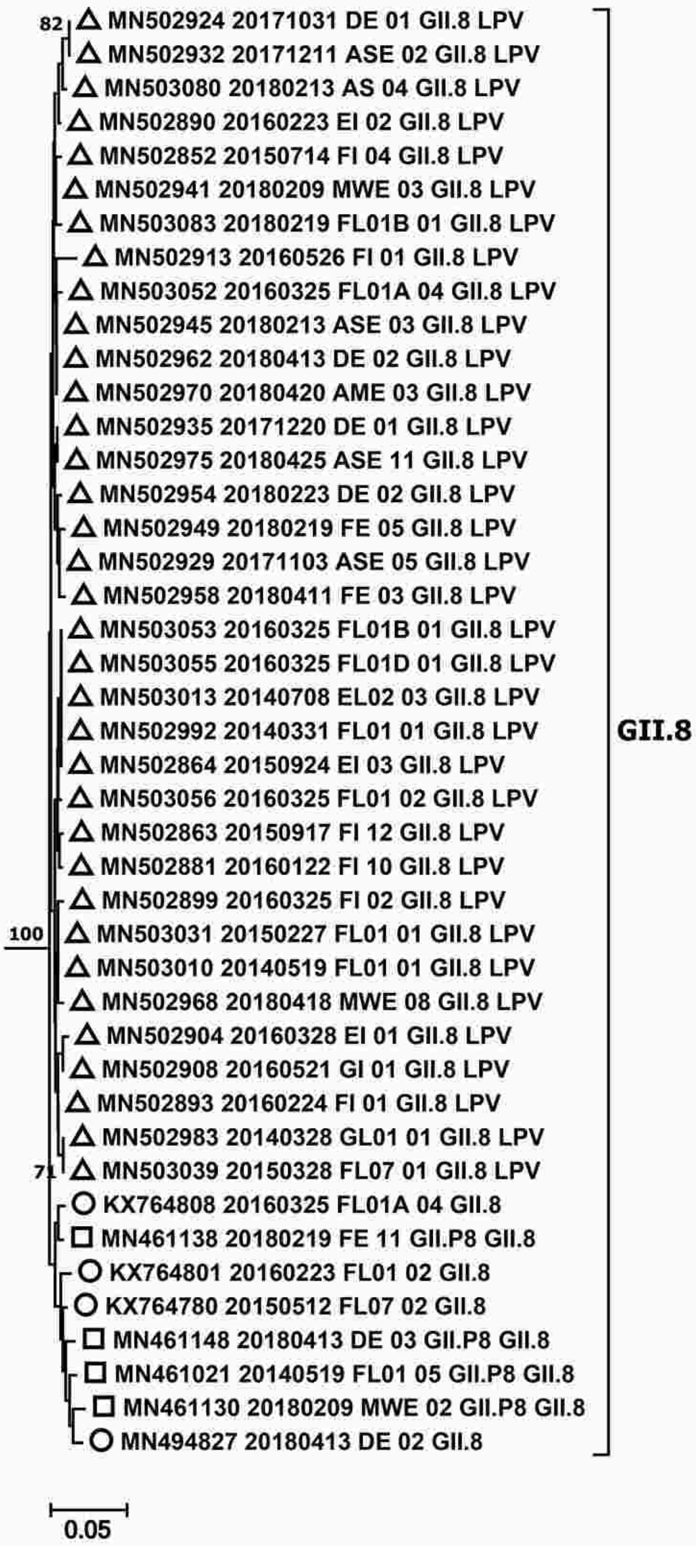

Fig S4

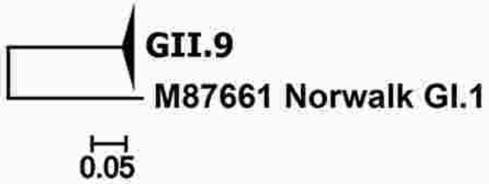

Marker	Amplicon type	Name of lineage
○	Region C	GII.9
△	VP1	GII.9
□	ORF1/2 junction	GII.P7/GII.9

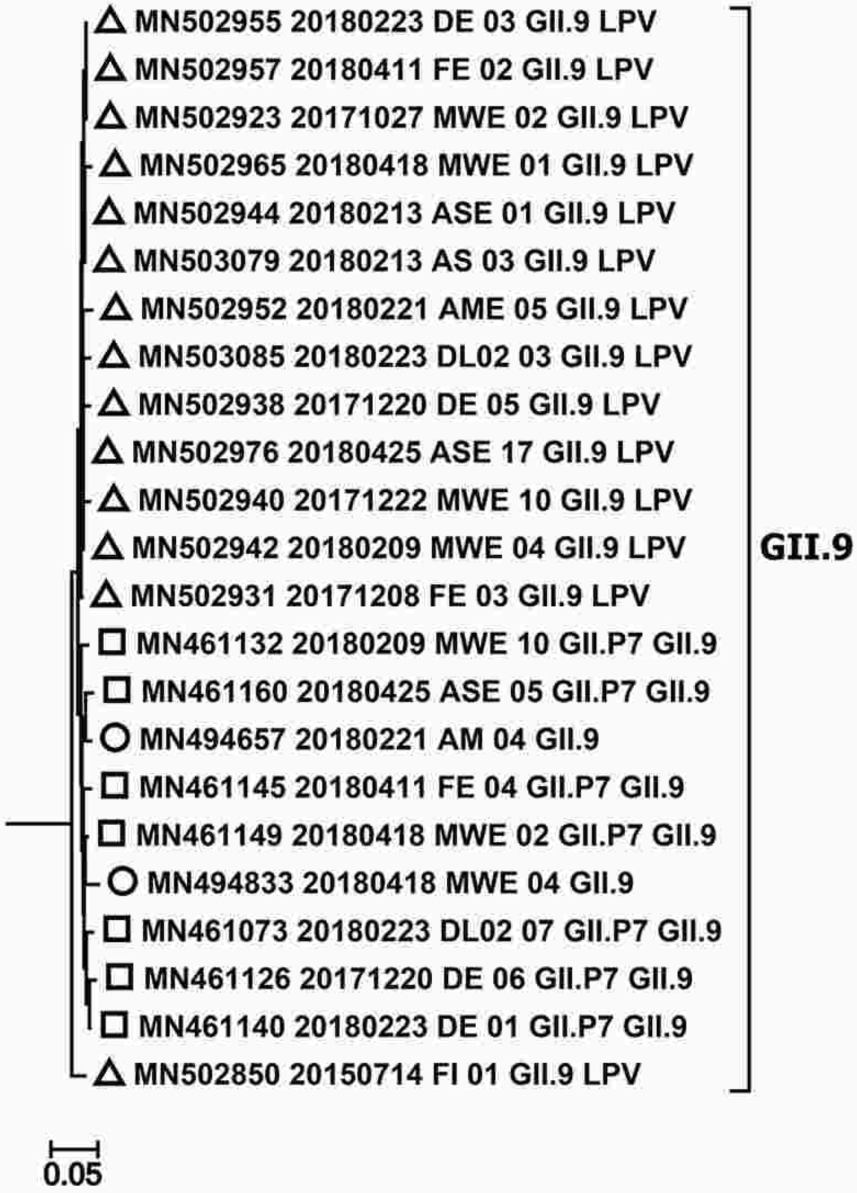

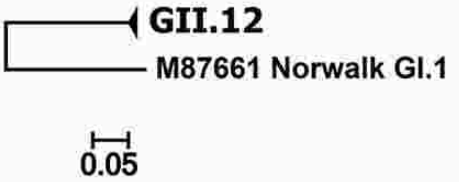

Marker	Amplicon type	Name of lineage
○	Region C	GII.12
△	VP1	GII.12

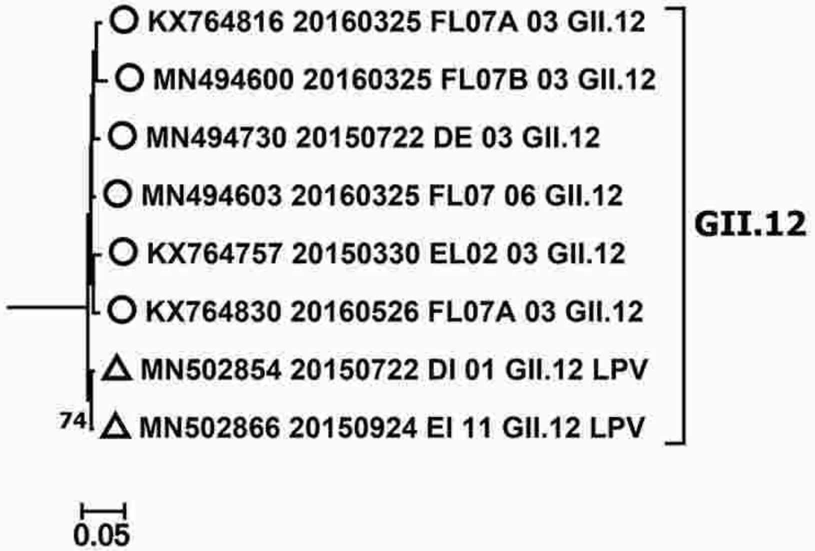

Fig S4

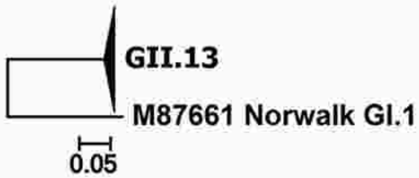

Marker	Amplicon type	Name of lineage
○	Region C	GII.13
△	VP1	GII.13
□	ORF1/2 junction	GII.P16/GII.13

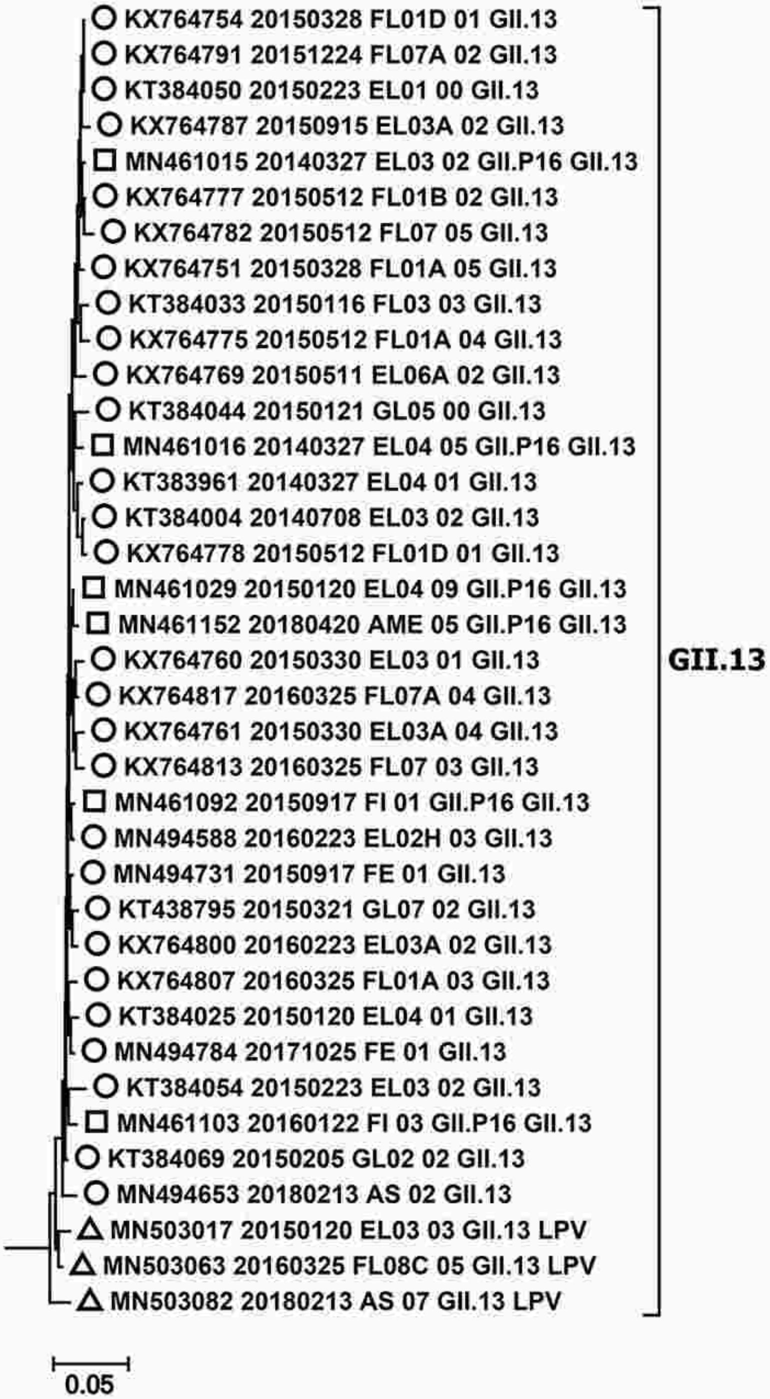

Fig S4

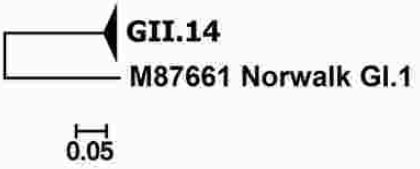

Marker	Amplicon type	Name of lineage
○	Region C	GII.14
△	VP1	GII.14
□	ORF1/2 junction	GII.P7/GII.14

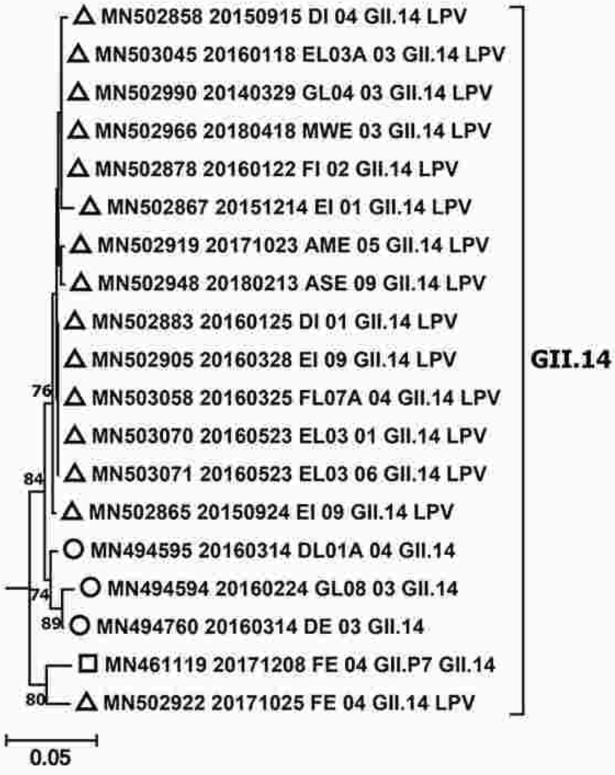

Fig S4

Marker	Amplicon type	Name of lineage
○	Region C	GII.17n
△	VP1	GII.17n
□	ORF1/2 junction	GII.P17/GII.17n

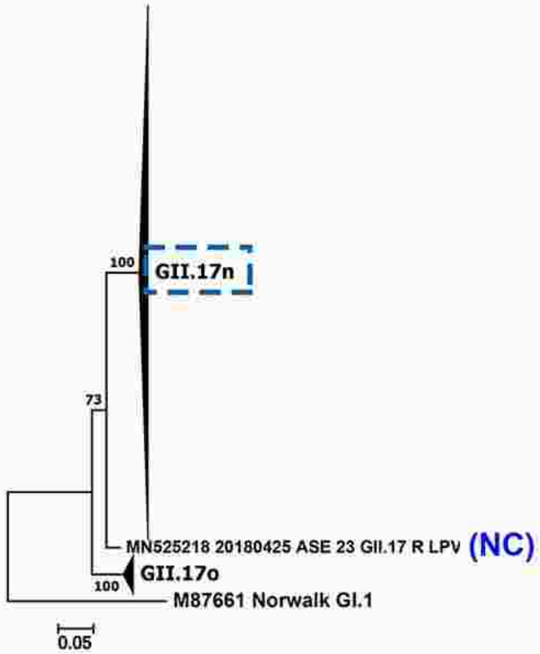

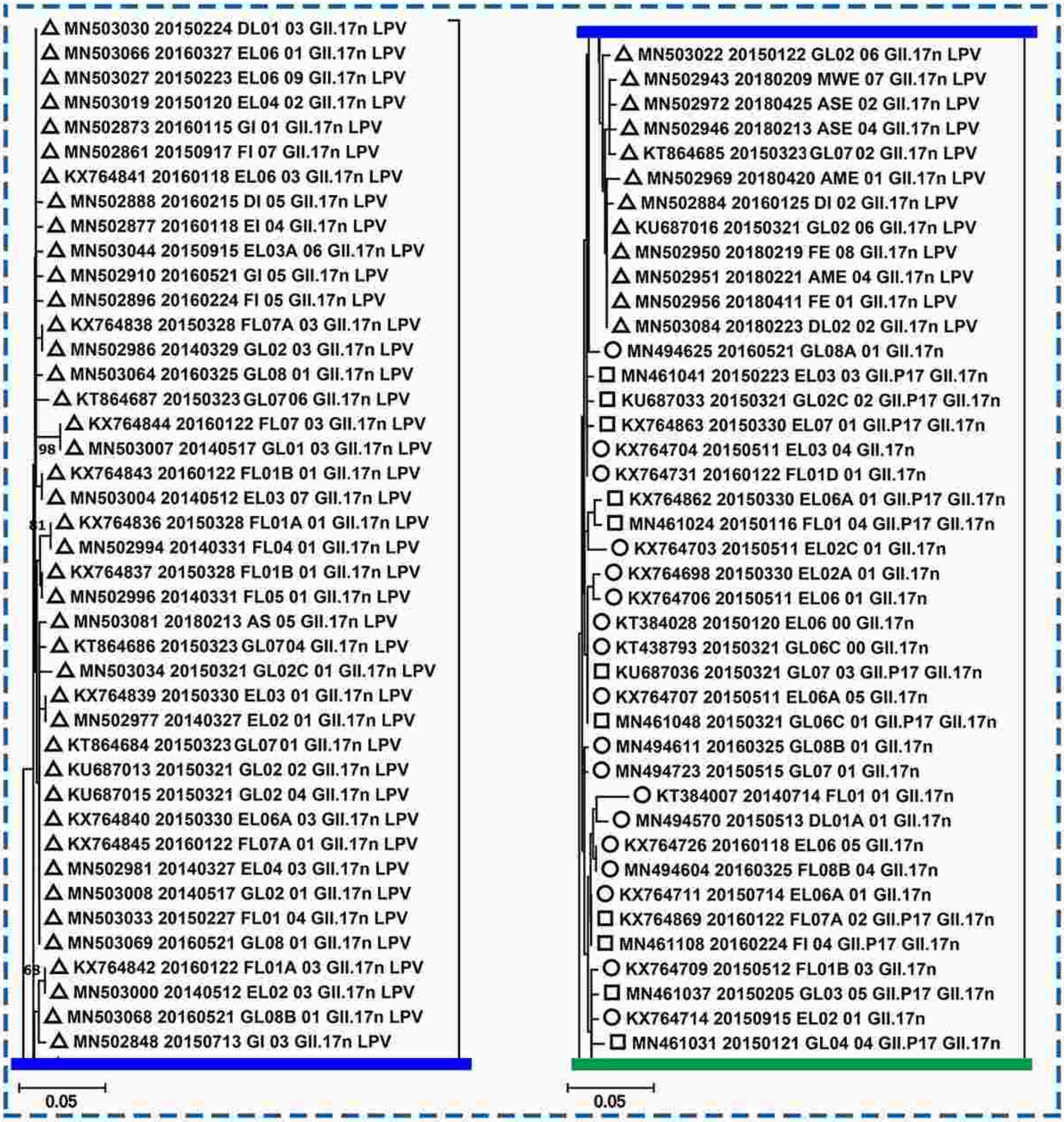

Fig S4

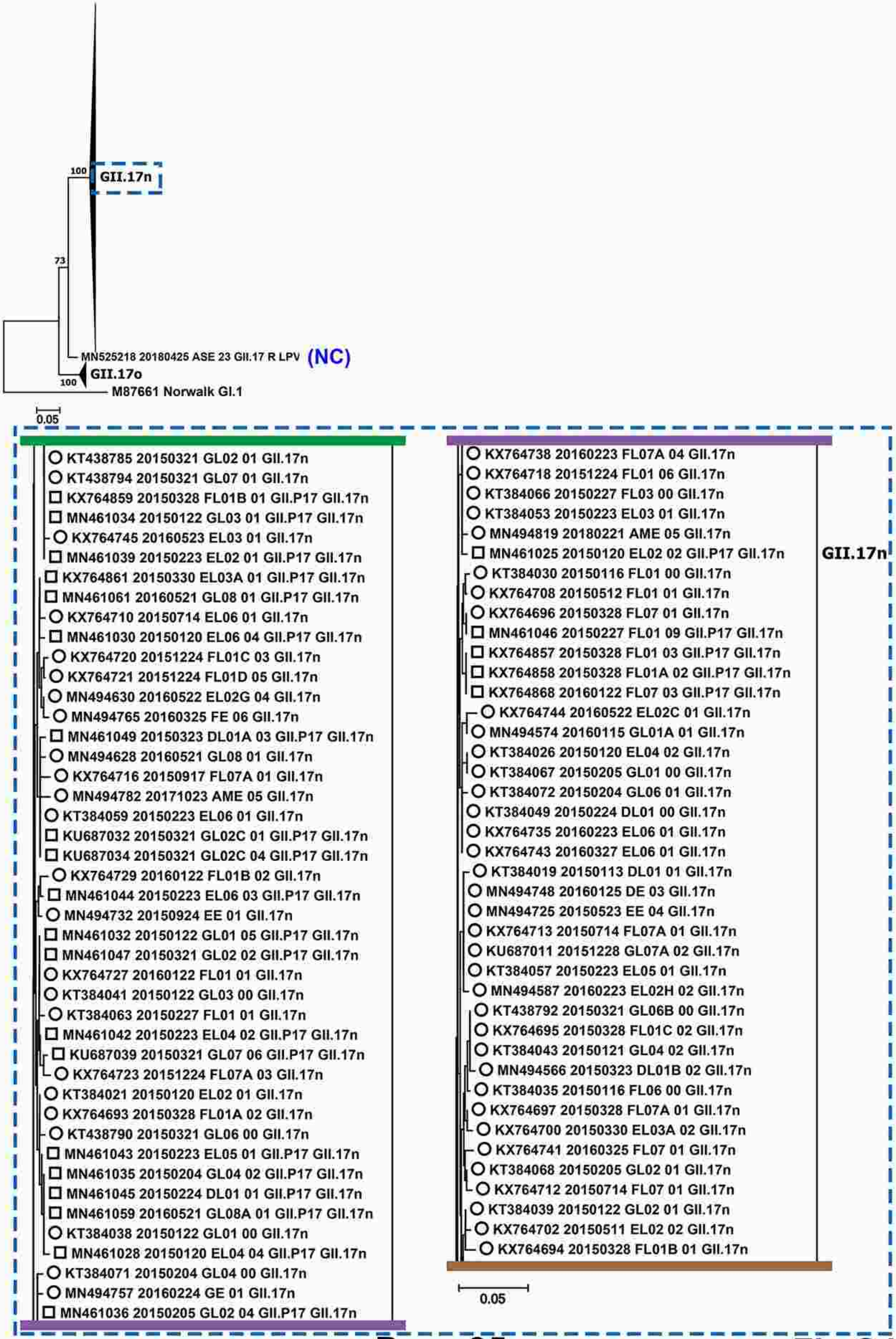
100
GII.17n
73
MN525218 20180425 ASE 23 GII.17 R LPV
(NC)
GII.17o
100
M87661 Norwalk GI.1
0.05
KT438785 20150321 GL02 01 GII.17n
KT438794 20150321 GL07 01 GII.17n
KX764859 20150328 FL01B 01 GII.P17 GII.17n
MN461034 20150122 GL03 01 GII.P17 GII.17n
KX764745 20160523 EL03 01 GII.17n
MN461039 20150223 EL02 01 GII.P17 GII.17n
KX764861 20150330 EL03A 01 GII.P17 GII.17n
MN461061 20160521 GL08 01 GII.P17 GII.17n
KX764710 20150714 EL06 01 GII.17n
MN461030 20150120 EL06 04 GII.P17 GII.17n
KX764720 20151224 FL01C 03 GII.17n
KX764721 20151224 FL01D 05 GII.17n
MN494630 20160522 EL02G 04 GII.17n
MN494765 20160325 FE 06 GII.17n
MN461049 20150323 DL01A 03 GII.P17 GII.17n
MN494628 20160521 GL08 01 GII.17n
KX764716 20150917 FL07A 01 GII.17n
MN494782 20171023 AME 05 GII.17n
KT384059 20150223 EL06 01 GII.17n
KU687032 20150321 GL02C 01 GII.P17 GII.17n
KU687034 20150321 GL02C 04 GII.P17 GII.17n
KX764729 20160122 FL01B 02 GII.17n
MN461044 20150223 EL06 03 GII.P17 GII.17n
MN494732 20150924 EE 01 GII.17n
MN461032 20150122 GL01 05 GII.P17 GII.17n
MN461047 20150321 GL02 02 GII.P17 GII.17n
KX764727 20160122 FL01 01 GII.17n
KT384041 20150122 GL03 00 GII.17n
KT384063 20150227 FL01 01 GII.17n
MN461042 20150223 EL04 02 GII.P17 GII.17n
KU687039 20150321 GL07 06 GII.P17 GII.17n
KX764723 20151224 FL07A 03 GII.17n
KT384021 20150120 EL02 01 GII.17n
KX764693 20150328 FL01A 02 GII.17n
KT438790 20150321 GL06 00 GII.17n
MN461043 20150223 EL05 01 GII.P17 GII.17n
MN461035 20150204 GL04 02 GII.P17 GII.17n
MN461045 20150224 DL01 01 GII.P17 GII.17n
MN461059 20160521 GL08A 01 GII.P17 GII.17n
KT384038 20150122 GL01 00 GII.17n
MN461028 20150120 EL04 04 GII.P17 GII.17n
KT384071 20150204 GL04 00 GII.17n
MN494757 20160224 GE 01 GII.17n
MN461036 20150205 GL02 04 GII.P17 GII.17n
KX764738 20160223 FL07A 04 GII.17n
KX764718 20151224 FL01 06 GII.17n
KT384066 20150227 FL03 00 GII.17n
KT384053 20150223 EL03 01 GII.17n
MN494819 20180221 AME 05 GII.17n
MN461025 20150120 EL02 02 GII.P17 GII.17n
GII.17n
KT384030 20150116 FL01 00 GII.17n
KX764708 20150512 FL01 01 GII.17n
KX764696 20150328 FL07 01 GII.17n
MN461046 20150227 FL01 09 GII.P17 GII.17n
KX764857 20150328 FL01 03 GII.P17 GII.17n
KX764858 20150328 FL01A 02 GII.P17 GII.17n
KX764868 20160122 FL07 03 GII.P17 GII.17n
KX764744 20160522 EL02C 01 GII.17n
MN494574 20160115 GL01A 01 GII.17n
KT384026 20150120 EL04 02 GII.17n
KT384067 20150205 GL01 00 GII.17n
KT384072 20150204 GL06 01 GII.17n
KT384049 20150224 DL01 00 GII.17n
KX764735 20160223 EL06 01 GII.17n
KX764743 20160327 EL06 01 GII.17n
KT384019 20150113 DL01 01 GII.17n
MN494748 20160125 DE 03 GII.17n
MN494725 20150523 EE 04 GII.17n
KX764713 20150714 FL07A 01 GII.17n
KU687011 20151228 GL07A 02 GII.17n
KT384057 20150223 EL05 01 GII.17n
MN494587 20160223 EL02H 02 GII.17n
KT438792 20150321 GL06B 00 GII.17n
KX764695 20150328 FL01C 02 GII.17n
KT384043 20150121 GL04 02 GII.17n
MN494566 20150323 DL01B 02 GII.17n
KT384035 20150116 FL06 00 GII.17n
KX764697 20150328 FL07A 01 GII.17n
KX764700 20150330 EL03A 02 GII.17n
KX764741 20160325 FL07 01 GII.17n
KT384068 20150205 GL02 01 GII.17n
KX764712 20150714 FL07 01 GII.17n
KT384039 20150122 GL02 01 GII.17n
KX764702 20150511 EL02 02 GII.17n
KX764694 20150328 FL01B 01 GII.17n
0.05

Fig S4

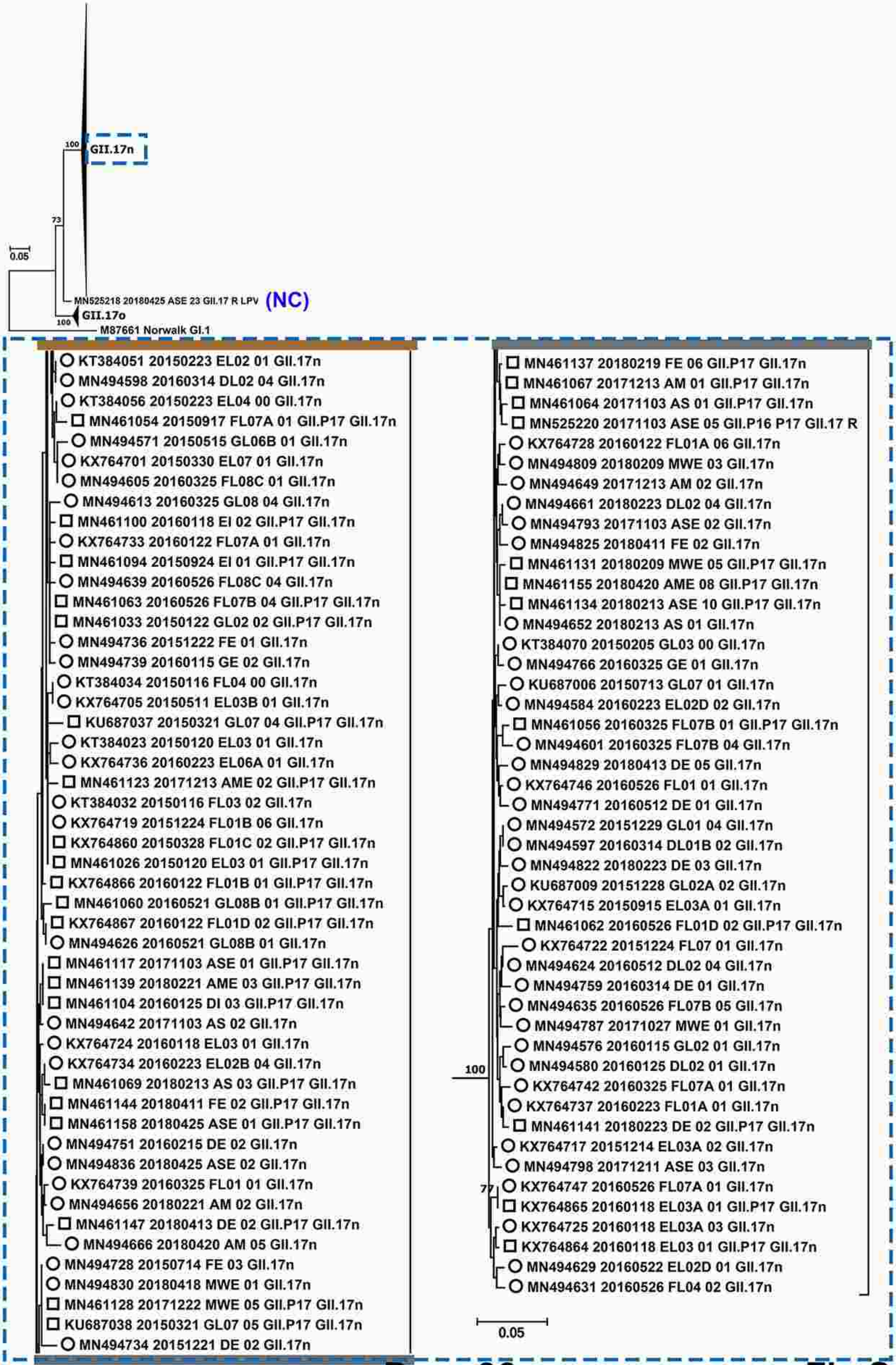
100
GII.17n
73
0.05
MN525218 20180425 ASE 23 GII.17 R LPV
(NC)
GII.17o
100
M87661 Norwalk GI.1
KT384051 20150223 EL02 01 GII.17n
MN494598 20160314 DL02 04 GII.17n
KT384056 20150223 EL04 00 GII.17n
MN461054 20150917 FL07A 01 GII.P17 GII.17n
MN494571 20150515 GL06B 01 GII.17n
KX764701 20150330 EL07 01 GII.17n
MN494605 20160325 FL08C 01 GII.17n
MN494613 20160325 GL08 04 GII.17n
MN461100 20160118 EI 02 GII.P17 GII.17n
KX764733 20160122 FL07A 01 GII.17n
MN461094 20150924 EI 01 GII.P17 GII.17n
MN494639 20160526 FL08C 04 GII.17n
MN461063 20160526 FL07B 04 GII.P17 GII.17n
MN461033 20150122 GL02 02 GII.P17 GII.17n
MN494736 20151222 FE 01 GII.17n
MN494739 20160115 GE 02 GII.17n
KT384034 20150116 FL04 00 GII.17n
KX764705 20150511 EL03B 01 GII.17n
KU687037 20150321 GL07 04 GII.P17 GII.17n
KT384023 20150120 EL03 01 GII.17n
KX764736 20160223 EL06A 01 GII.17n
MN461123 20171213 AME 02 GII.P17 GII.17n
KT384032 20150116 FL03 02 GII.17n
KX764719 20151224 FL01B 06 GII.17n
KX764860 20150328 FL01C 02 GII.P17 GII.17n
MN461026 20150120 EL03 01 GII.P17 GII.17n
KX764866 20160122 FL01B 01 GII.P17 GII.17n
MN461060 20160521 GL08B 01 GII.P17 GII.17n
KX764867 20160122 FL01D 02 GII.P17 GII.17n
MN494626 20160521 GL08B 01 GII.17n
MN461117 20171103 ASE 01 GII.P17 GII.17n
MN461139 20180221 AME 03 GII.P17 GII.17n
MN461104 20160125 DI 03 GII.P17 GII.17n
MN494642 20171103 AS 02 GII.17n
KX764724 20160118 EL03 01 GII.17n
KX764734 20160223 EL02B 04 GII.17n
MN461069 20180213 AS 03 GII.P17 GII.17n
MN461144 20180411 FE 02 GII.P17 GII.17n
MN461158 20180425 ASE 01 GII.P17 GII.17n
MN494751 20160215 DE 02 GII.17n
MN494836 20180425 ASE 02 GII.17n
KX764739 20160325 FL01 01 GII.17n
MN494656 20180221 AM 02 GII.17n
MN461147 20180413 DE 02 GII.P17 GII.17n
MN494666 20180420 AM 05 GII.17n
MN494728 20150714 FE 03 GII.17n
MN494830 20180418 MWE 01 GII.17n
MN461128 20171222 MWE 05 GII.P17 GII.17n
KU687038 20150321 GL07 05 GII.P17 GII.17n
MN494734 20151221 DE 02 GII.17n
MN461137 20180219 FE 06 GII.P17 GII.17n
MN461067 20171213 AM 01 GII.P17 GII.17n
MN461064 20171103 AS 01 GII.P17 GII.17n
MN525220 20171103 ASE 05 GII.P16 P17 GII.17 R
KX764728 20160122 FL01A 06 GII.17n
MN494809 20180209 MWE 03 GII.17n
MN494649 20171213 AM 02 GII.17n
MN494661 20180223 DL02 04 GII.17n
MN494793 20171103 ASE 02 GII.17n
MN494825 20180411 FE 02 GII.17n
MN461131 20180209 MWE 05 GII.P17 GII.17n
MN461155 20180420 AME 08 GII.P17 GII.17n
MN461134 20180213 ASE 10 GII.P17 GII.17n
MN494652 20180213 AS 01 GII.17n
KT384070 20150205 GL03 00 GII.17n
MN494766 20160325 GE 01 GII.17n
KU687006 20150713 GL07 01 GII.17n
MN494584 20160223 EL02D 02 GII.17n
MN461056 20160325 FL07B 01 GII.P17 GII.17n
MN494601 20160325 FL07B 04 GII.17n
MN494829 20180413 DE 05 GII.17n
KX764746 20160526 FL01 01 GII.17n
MN494771 20160512 DE 01 GII.17n
MN494572 20151229 GL01 04 GII.17n
MN494597 20160314 DL01B 02 GII.17n
MN494822 20180223 DE 03 GII.17n
KU687009 20151228 GL02A 02 GII.17n
KX764715 20150915 EL03A 01 GII.17n
MN461062 20160526 FL01D 02 GII.P17 GII.17n
KX764722 20151224 FL07 01 GII.17n
MN494624 20160512 DL02 04 GII.17n
MN494759 20160314 DE 01 GII.17n
MN494635 20160526 FL07B 05 GII.17n
MN494787 20171027 MWE 01 GII.17n
MN494576 20160115 GL02 01 GII.17n
100
MN494580 20160125 DL02 01 GII.17n
KX764742 20160325 FL07A 01 GII.17n
KX764737 20160223 FL01A 01 GII.17n
MN461141 20180223 DE 02 GII.P17 GII.17n
KX764717 20151214 EL03A 02 GII.17n
MN494798 20171211 ASE 03 GII.17n
77
KX764747 20160526 FL07A 01 GII.17n
KX764865 20160118 EL03A 01 GII.P17 GII.17n
KX764725 20160118 EL03A 03 GII.17n
KX764864 20160118 EL03 01 GII.P17 GII.17n
MN494629 20160522 EL02D 01 GII.17n
MN494631 20160526 FL04 02 GII.17n
0.05

Fig S4

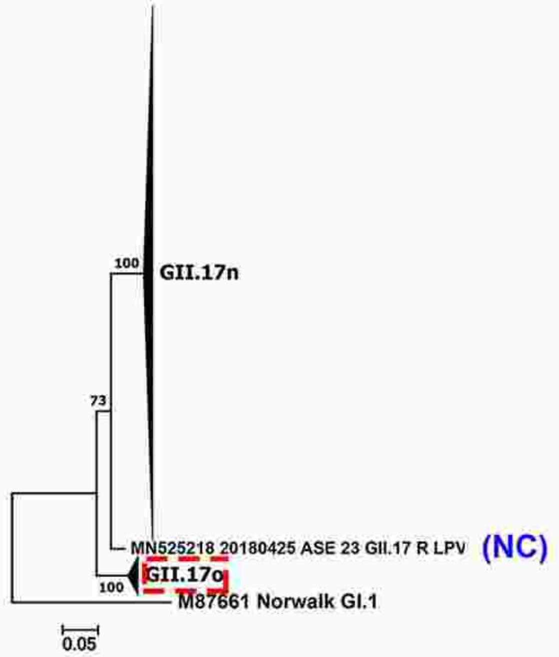

Marker	Amplicon type	Name of lineage
●	Region C	GII.17o
▲	VP1	GII.17o
■	ORF1/2 junction	GII.P31/GII.17o

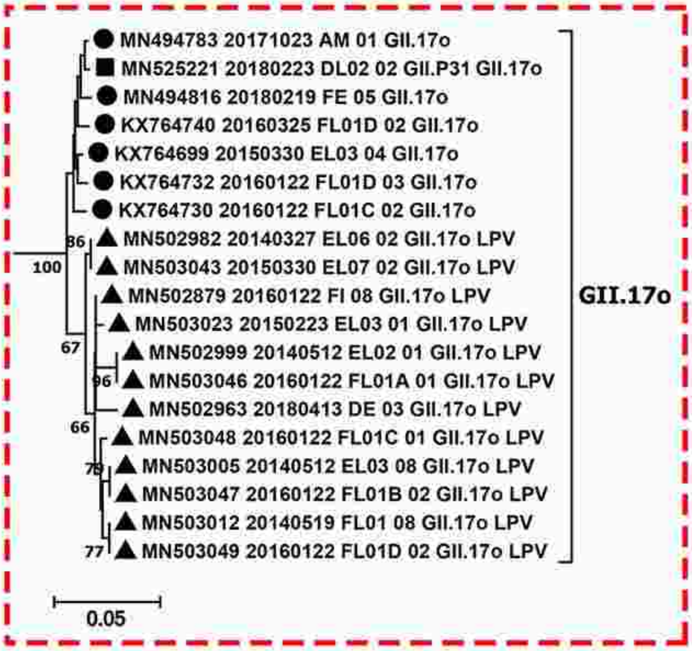

Fig S4

a

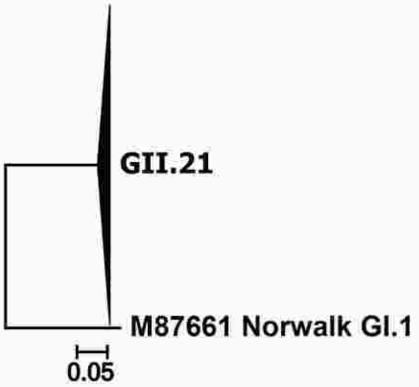

b

Marker	Amplicon type	Name of region C lineage
○	Region C	GII.21
△	VP1	GII.21
□	ORF1/2 junction	GII.P21/GII.21

c

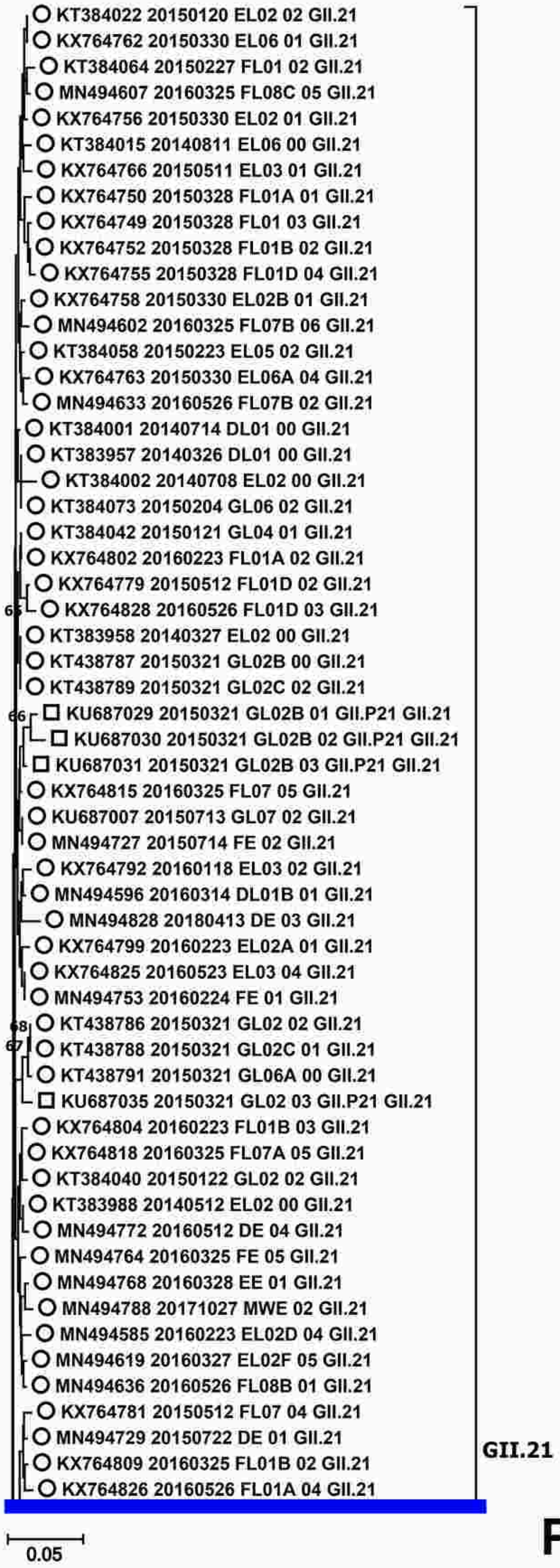

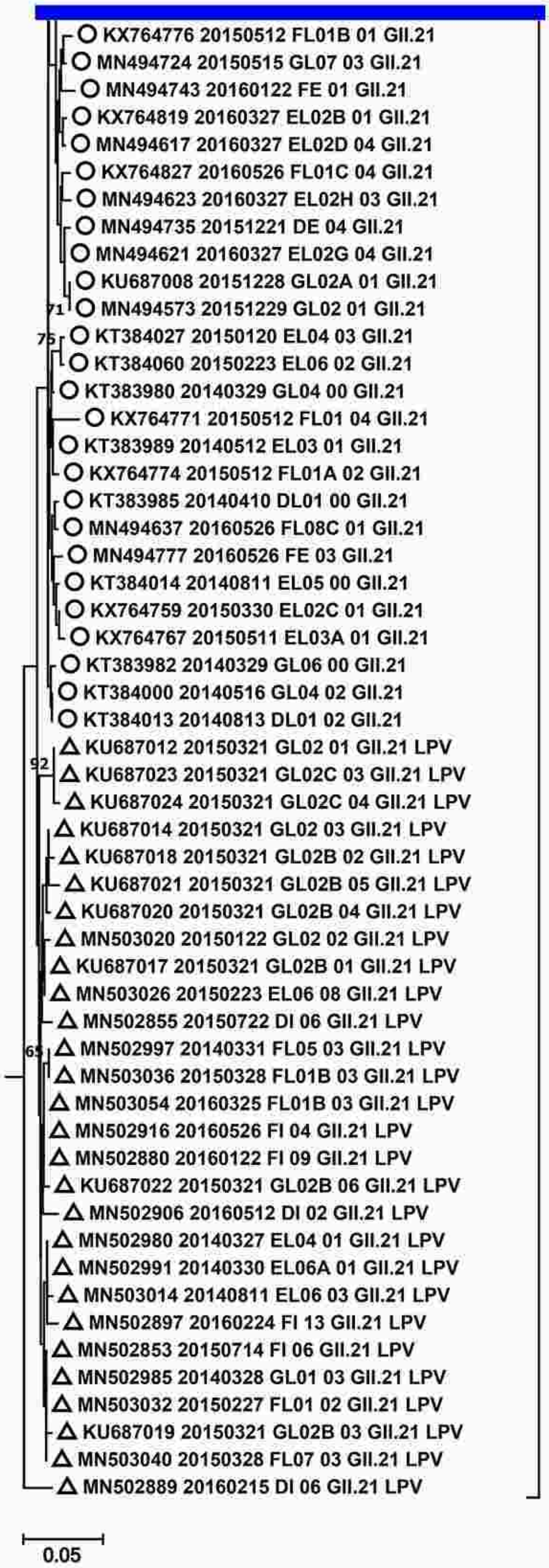

Fig S4

FIG S4 Expanded view of the compressed phylogenetic trees used for defining sub-genotypic region C of isolated nucleotide sequences from both norovirus genogroup I (GI) and genogroup II (GII). The compressed trees in S2 and S3 Figs, and their expanded views are shown together. Dashed-line red, blue, or black boxes surrounding sub-genotypic clusters in the compressed tree indicate expanded views of these on the same page. Markers, amplicon type, and names of region C lineages in the clusters of the expanded view are shown in a table on the same page. In the table, names of region C lineages of ORF1/2 junction amplicon sequences are indicated together with their ORF1 genotypes. In the expanded view, previously suggested sub-genotypic reference sequences are indicated by red asterisks (*). The name of each isolated sequence in the expanded views is composed in the following order: GenBank ID, isolation date (YYYYMMDD), name of sampling site (e.g. FL07A), clone number (e.g. 06), and lineage name (e.g. GII.6a). The name of each analyzed genotype is shown at the bottom of each page. Colored horizontal bars located at the bottom or top of several expanded views (GI.3, GI.5, GI.9, GII.4, GII.17, and GII.21) indicate that the two identically colored regions of the trees are vertically connected in the original tree.

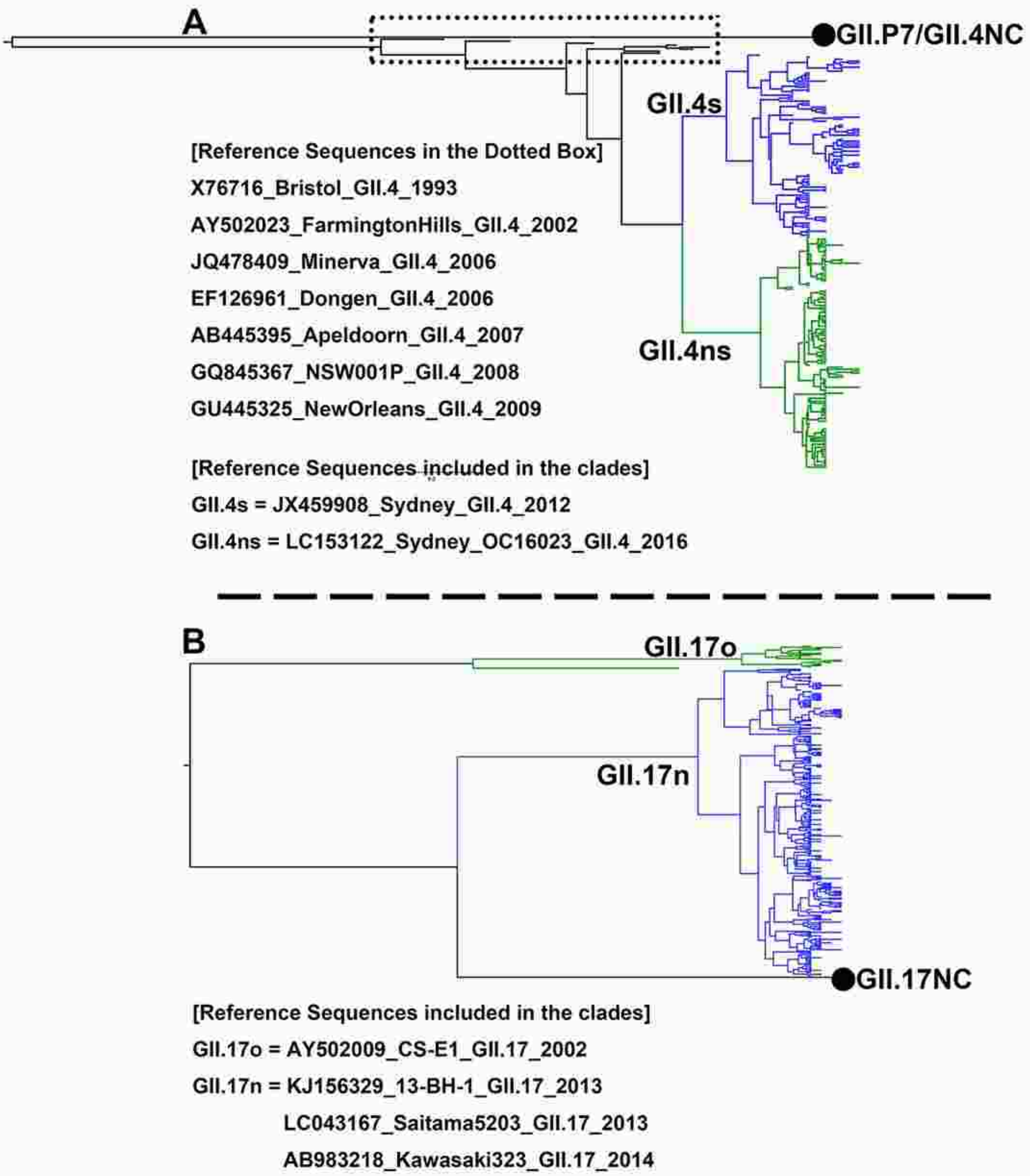
A
GII.P7/GII.4NC
GII.4s
GII.4ns
[Reference Sequences in the Dotted Box]
X76716_Bristol_GII.4_1993
AY502023_FarmingtonHills_GII.4_2002
JQ478409_Minerva_GII.4_2006
EF126961_Dongen_GII.4_2006
AB445395_Apeldoorn_GII.4_2007
GQ845367_NSW001P_GII.4_2008
GU445325_NewOrleans_GII.4_2009
[Reference Sequences included in the clades]
GII.4s = JX459908_Sydney_GII.4_2012
GII.4ns = LC153122_Sydney_OC16023_GII.4_2016
B
GII.17o
GII.17n
GII.17NC
[Reference Sequences included in the clades]
GII.17o = AY502009_CS-E1_GII.17_2002
GII.17n = KJ156329_13-BH-1_GII.17_2013
LC043167_Saitama5203_GII.17_2013
AB983218_Kawasaki323_GII.17_2014

Fig S5

FIG S5 Maximum clade credibility (MCC) trees of common region C (0.3 kb) for both GII.4 and GII.17 of three amplicon types (region C, VP1, and ORF1/2 junction). **(A)** MCC tree for the common region C GII.4. A dotted box in panel indicates reference sequences isolated before the emergence of the GII.4 Sydney clades. The name of each reference sequence is described in the following order: GenBank ID, strain name, genotype, and isolation year. **(B)** MCC tree for the common region C GII.17. In each tree of the two respective panels, the two clades are shown in different colors: both GII.4s (blue) and GII.4ns (green); both GII.17o (green) and GII.17n (blue). Names of the reference sequences included in the clades are shown below each tree. The closed circles (●) indicate an NC branch in each tree as follows; GII.P7/GII.4NC (ORF1/2 junction amplicon) or GII.17NC (VP1 amplicon); (Fig. 2).

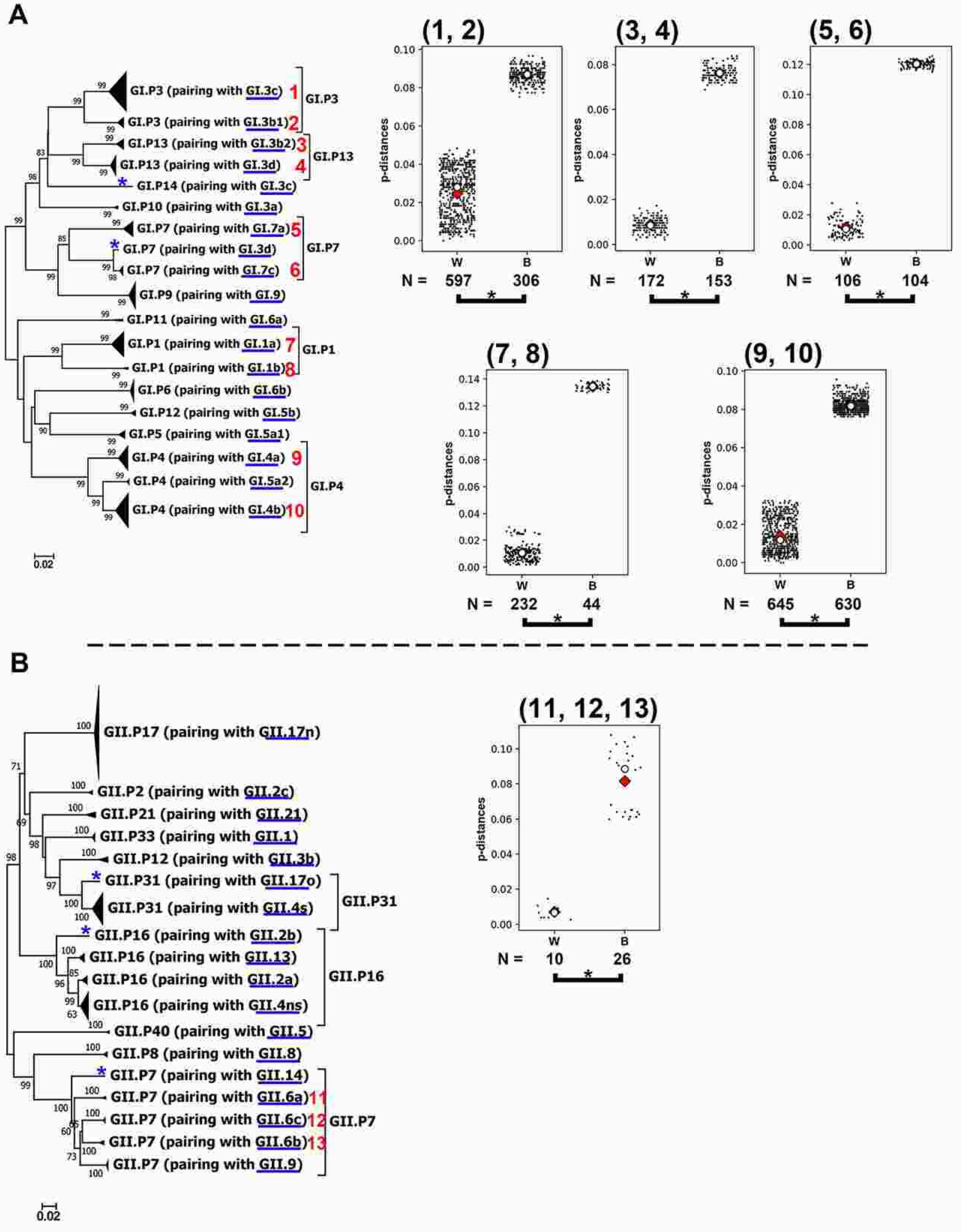
A
GI.P3 (pairing with GI.3c) 1
GI.P3 (pairing with GI.3b1) 2
GI.P3
GI.P13 (pairing with GI.3b2) 3
GI.P13 (pairing with GI.3d) 4
GI.P13
GI.P14 (pairing with GI.3c)
GI.P10 (pairing with GI.3a)
GI.P7 (pairing with GI.7a) 5
GI.P7 (pairing with GI.3d)
GI.P7 (pairing with GI.7c) 6
GI.P7
GI.P9 (pairing with GI.9)
GI.P11 (pairing with GI.6a)
GI.P1 (pairing with GI.1a) 7
GI.P1 (pairing with GI.1b) 8
GI.P1
GI.P6 (pairing with GI.6b)
GI.P12 (pairing with GI.5b)
GI.P5 (pairing with GI.5a1)
GI.P4 (pairing with GI.4a) 9
GI.P4 (pairing with GI.5a2)
GI.P4 (pairing with GI.4b) 10
GI.P4
0.02
(1, 2)
(3, 4)
(5, 6)
(7, 8)
(9, 10)
p-distances
W
B
N = 597 306
N = 172 153
N = 106 104
N = 232 44
N = 645 630
B
GII.P17 (pairing with GII.17n)
GII.P2 (pairing with GII.2c)
GII.P21 (pairing with GII.21)
GII.P33 (pairing with GII.1)
GII.P12 (pairing with GII.3b)
GII.P31 (pairing with GII.17o)
GII.P31 (pairing with GII.4s)
GII.P31
GII.P16 (pairing with GII.2b)
GII.P16 (pairing with GII.13)
GII.P16 (pairing with GII.2a)
GII.P16 (pairing with GII.4ns)
GII.P16
GII.P40 (pairing with GII.5)
GII.P8 (pairing with GII.8)
GII.P7 (pairing with GII.14)
GII.P7 (pairing with GII.6a) 11
GII.P7 (pairing with GII.6c) 12
GII.P7 (pairing with GII.6b) 13
GII.P7 (pairing with GII.9)
GII.P7
0.02
(11, 12, 13)
p-distances
W
B
N = 10 26

Fig S6

C

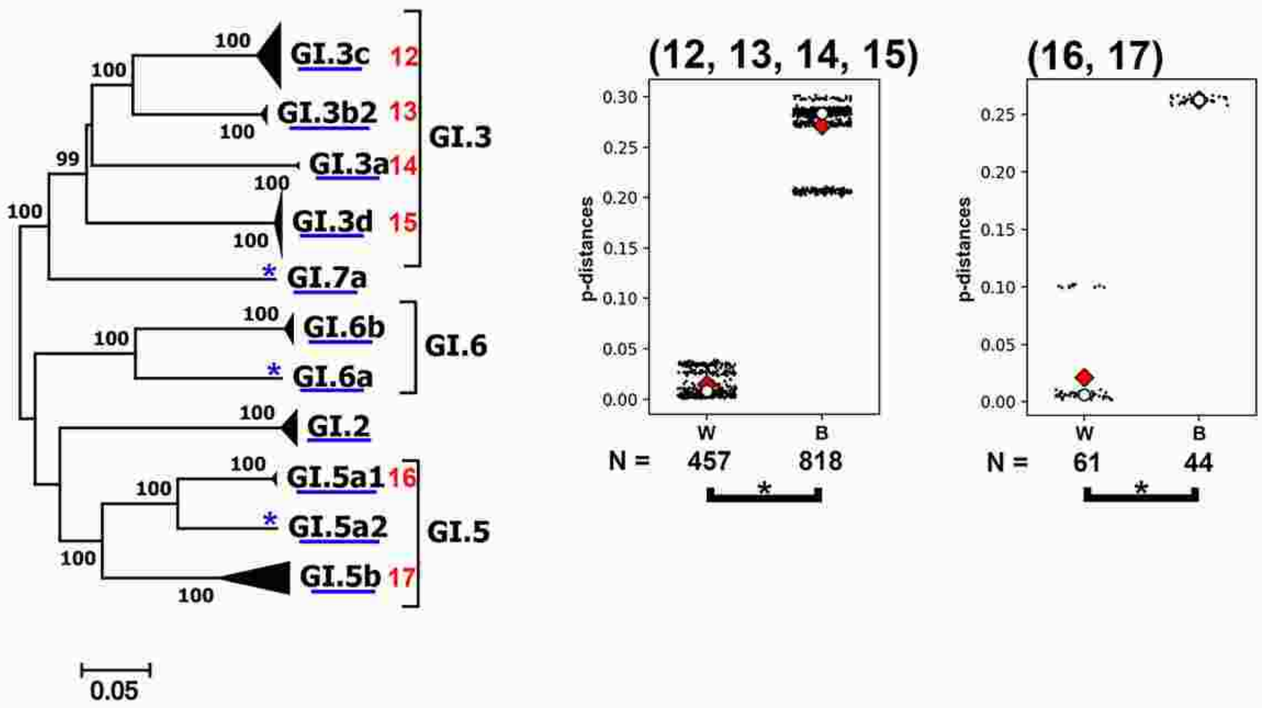
GI.3c 12
GI.3b2 13
GI.3a 14
GI.3d 15
GI.3
GI.7a
GI.6b
GI.6a
GI.6
GI.2
GI.5a1 16
GI.5a2
GI.5b 17
GI.5
0.05
(12, 13, 14, 15)
(16, 17)
p-distances
W
B
N = 457 818
N = 61 44
*

D

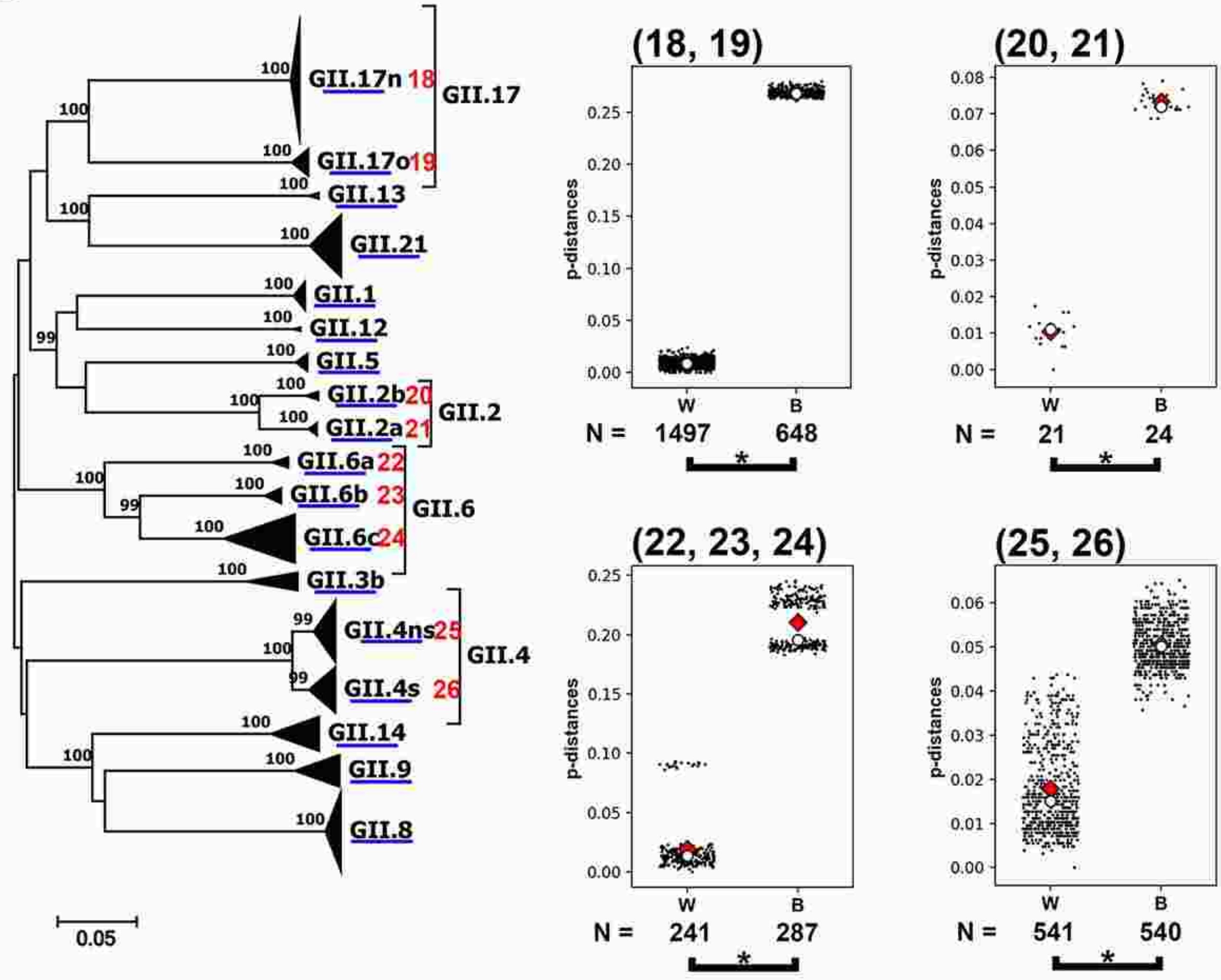
GII.17n 18
GII.17o 19
GII.17
GII.13
GII.21
GII.1
GII.12
GII.5
GII.2b 20
GII.2a 21
GII.2
GII.6a 22
GII.6b 23
GII.6c 24
GII.6
GII.3b
GII.4ns 25
GII.4s 26
GII.4
GII.14
GII.9
GII.8
0.05
(18, 19)
(20, 21)
(22, 23, 24)
(25, 26)
p-distances
W
B
N = 1497 648
N = 21 24
N = 241 287
N = 541 540
*

Fig S6

FIG S6 Sub-genotypic common region C dependent clustering of both ORF1 and ORF2 sequences from the two genogroups. Neighbor-joining trees of both ORF1/2 junction amplicons (0.7-0.9 kb; panels **A** and **B**) and VP1 amplicons (1.2-1.3 kb; panels **C** and **D**) without common region C are shown. In each tree, names of common region C lineages of ORF1/ORF2 junction amplicons or VP1 amplicons are underlined in blue. Bootstrap values are shown next to the branches of each cluster. Single sequences diversified from a neighboring cluster are depicted by a blue asterisk (*) on the branch. Sub-genotypic clusters used to compare within- and between-group distances are marked as red numerals at the right side of the names of the sub-genotypic clusters. Both within (W) nucleotide p-distances and between (B) nucleotide p-distances of sub-genotypic clusters in a genotype are shown in the vertical scatter plots on the right side of the trees. Numerals on the top of each vertical scatter plot denote the compared sub-genotypic clusters as indicated by corresponding numerals in the tree. Means (◆) and medians (○) of the distance groups are shown in each vertical scatter plot. Sample sizes (N) of the compared distances are indicated below the vertical scatter plots. Asterisks (*) below the vertical scatter plots describe the statistical significance ($P < 0.05$) in the differences of mean ranks between the compared groups (Mann-Whitney U test).

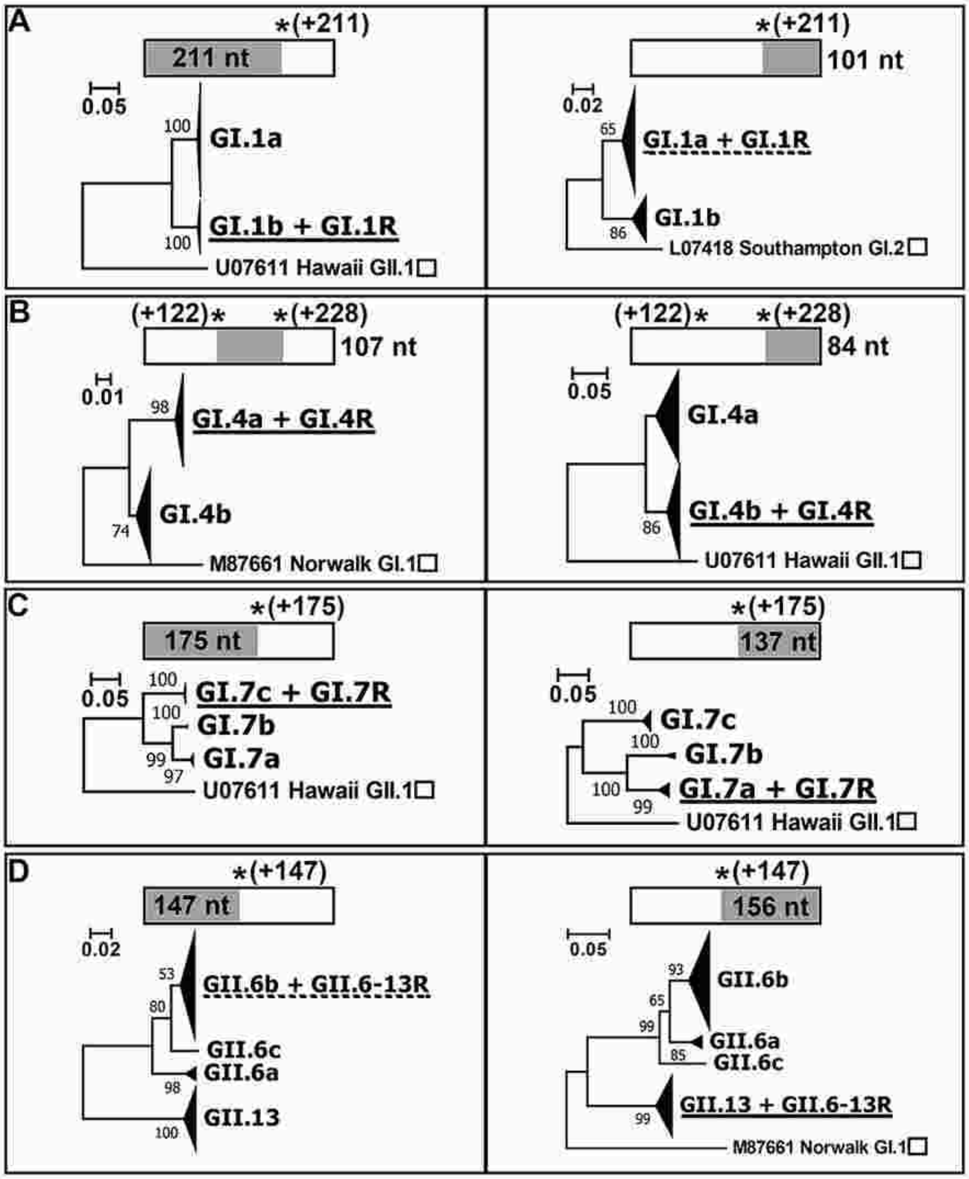
A
*(+211)
211 nt
0.05
100
GI.1a
GI.1b + GI.1R
100
U07611 Hawaii GII.1
*(+211)
101 nt
0.02
65
GI.1a + GI.1R
GI.1b
86
L07418 Southampton GI.2
B
(+122)* *(+228)
107 nt
0.01
98
GI.4a + GI.4R
GI.4b
74
M87661 Norwalk GI.1
(+122)* *(+228)
84 nt
0.05
GI.4a
GI.4b + GI.4R
86
U07611 Hawaii GII.1
C
*(+175)
175 nt
0.05
100
GI.7c + GI.7R
100
GI.7b
GI.7a
99
97
U07611 Hawaii GII.1
*(+175)
137 nt
0.05
100
GI.7c
100
GI.7b
100
GI.7a + GI.7R
99
U07611 Hawaii GII.1
D
*(+147)
147 nt
0.02
53
GII.6b + GII.6-13R
80
GII.6c
GII.6a
98
GII.13
100
*(+147)
156 nt
0.05
93
GII.6b
65
99
GII.6a
85
GII.6c
GII.13 + GII.6-13R
99
M87661 Norwalk GI.1

Fig S7

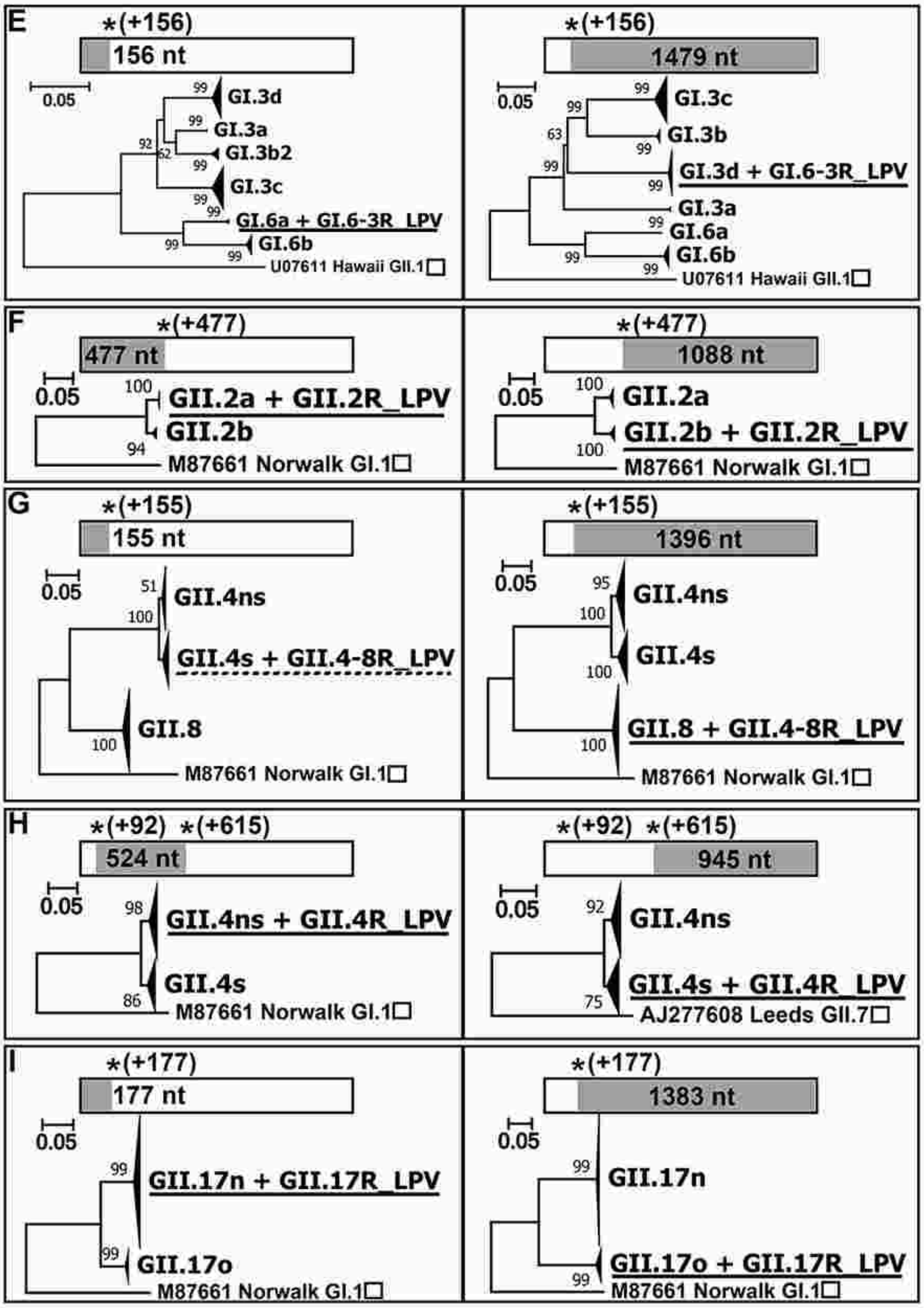
E
*(+156)
156 nt
0.05
GI.3d
GI.3a
GI.3b2
GI.3c
GI.6a + GI.6-3R_LPV
GI.6b
U07611 Hawaii GII.1
*(+156)
1479 nt
GI.3c
GI.3b
GI.3d + GI.6-3R_LPV
GI.3a
GI.6a
GI.6b
U07611 Hawaii GII.1
F
*(+477)
477 nt
GII.2a + GII.2R_LPV
GII.2b
M87661 Norwalk GI.1
1088 nt
GII.2a
GII.2b + GII.2R_LPV
M87661 Norwalk GI.1
G
*(+155)
155 nt
GII.4ns
GII.4s + GII.4-8R_LPV
GII.8
M87661 Norwalk GI.1
1396 nt
GII.4ns
GII.4s
GII.8 + GII.4-8R_LPV
M87661 Norwalk GI.1
H
*(+92) *(+615)
524 nt
GII.4ns + GII.4R_LPV
GII.4s
M87661 Norwalk GI.1
945 nt
GII.4ns
GII.4s + GII.4R_LPV
AJ277608 Leeds GII.7
I
*(+177)
177 nt
GII.17n + GII.17R_LPV
GII.17o
M87661 Norwalk GI.1
1383 nt
GII.17n
GII.17o + GII.17R_LPV
M87661 Norwalk GI.1

 Fig S7

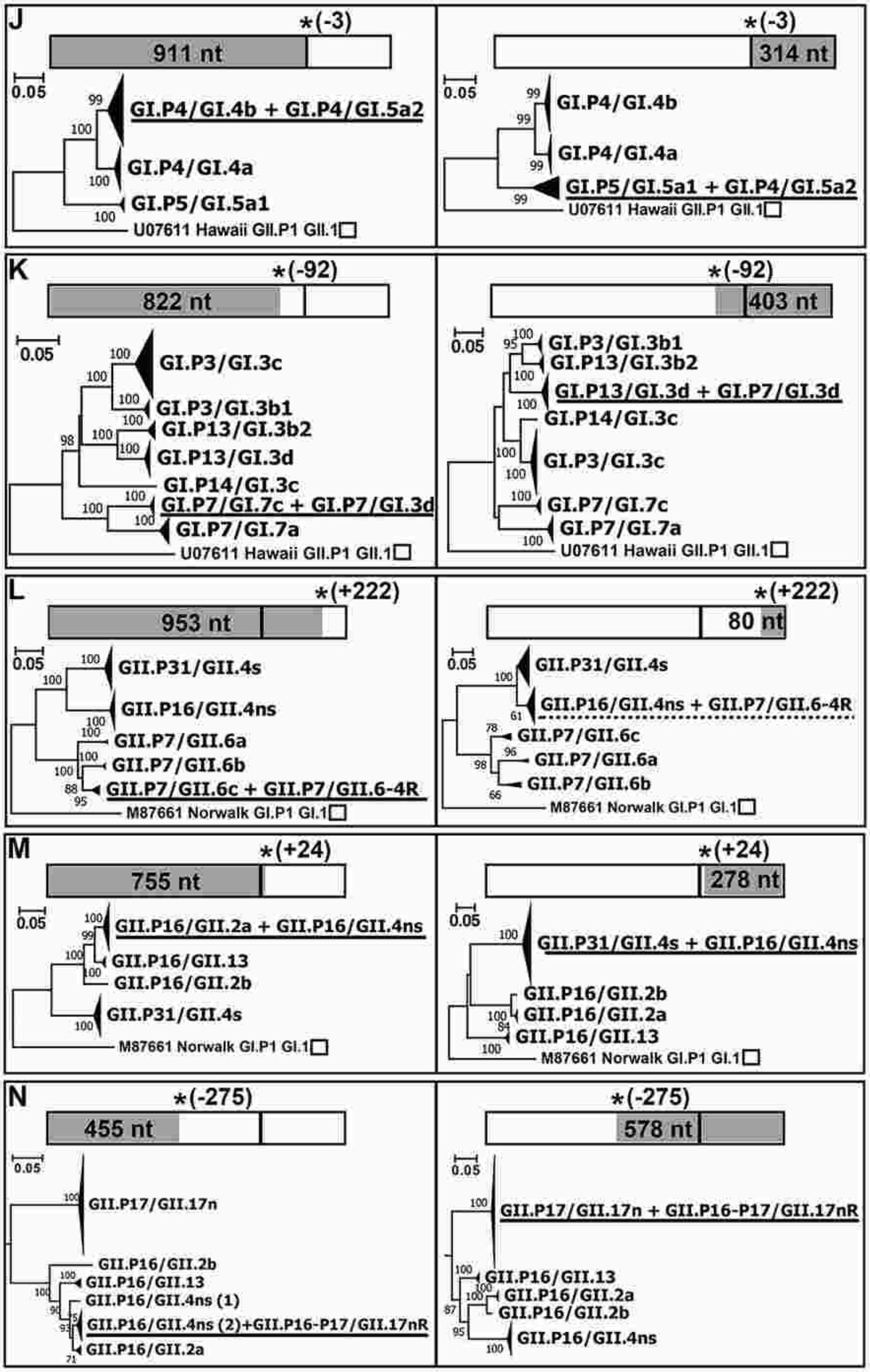
J
*(-3)
911 nt
314 nt
0.05
GI.P4/GI.4b + GI.P4/GI.5a2
GI.P4/GI.4a
GI.P5/GI.5a1
U07611 Hawaii GII.P1 GII.1
GI.P4/GI.4b
GI.P5/GI.5a1 + GI.P4/GI.5a2
K
*(-92)
822 nt
403 nt
GI.P3/GI.3c
GI.P3/GI.3b1
GI.P13/GI.3b2
GI.P13/GI.3d
GI.P14/GI.3c
GI.P7/GI.7c + GI.P7/GI.3d
GI.P7/GI.7a
GI.P13/GI.3d + GI.P7/GI.3d
GI.P7/GI.7c
L
*(+222)
953 nt
80 nt
GII.P31/GII.4s
GII.P16/GII.4ns
GII.P7/GII.6a
GII.P7/GII.6b
GII.P7/GII.6c + GII.P7/GII.6-4R
M87661 Norwalk GI.P1 GI.1
GII.P16/GII.4ns + GII.P7/GII.6-4R
GII.P7/GII.6c
M
*(+24)
755 nt
278 nt
GII.P16/GII.2a + GII.P16/GII.4ns
GII.P16/GII.13
GII.P16/GII.2b
GII.P31/GII.4s + GII.P16/GII.4ns
GII.P16/GII.2a
N
*(-275)
455 nt
578 nt
GII.P17/GII.17n
GII.P16/GII.4ns (1)
GII.P16/GII.4ns (2)+GII.P16-P17/GII.17nR
GII.P17/GII.17n + GII.P16-P17/GII.17nR

Fig S7

FIG S7 Compressed phylogenetic trees, based on recombination break points, of recombinant sequences with their parental sequences. Neighbor-joining trees of the isolated recombinants of three amplicon types are shown (panels **A-D**, region C amplicon; panels **E-I**, VP1 amplicon; panels **J-N**, ORF1/2 junction amplicon). Two compressed trees per recombinant are described in each panel (**A**, GI.1R; **B**, GI.4R; **C**, GI.7R; **D**, GII.6-13R, **E**, GI.6-3R_LPV; **F**, GII.2R_LPV; **G**, GII.4-8R_LPV; **H**, GII.4R_LPV; **I**, GII.17R_LPV; **J**, GI.P4/GI.5a2; **K**, GI.P7/GI.3d; **L**, GII.P7/GII.6-4R; **M**, GII.P16/GII.4ns; **N**, GII.P16-P17/GII.17nR). A tree in each box consists of a group of sequence fragments of a recombinant and their related genotypic sequences. The region of fragments used in each tree is indicated as a grey region in the whole amplicon scale above each tree, and fragments are defined based on recombination break points (asterisk, *). Numerals next to asterisks (*) indicate positions of recombination break points from adenine (A) of the start codon of ORF2. An open square (□) indicates an outgroup reference strain in each tree. Nucleotide lengths of sequence fragments are described as the number of nucleotides (nt). Bootstrap values ≥ 50 are shown next to branches of the clusters. In each tree, a parental lineage cluster including the recombinant is underlined (name of the parental lineage plus name of the recombinant lineage); bootstrap values < 75 of the parental lineage cluster are depicted using a dotted underline (---). Additional vertical scatter plots for complementing the low reliability of bootstrap values < 75 are described (Fig. S8). These scatter plots in Fig. S8 compare minimum nucleotide distances between the recombinant sequences and the parental/non-parental clusters, thus showing the existence of parental sequences most closely related to recombinant sequences in parental clusters.

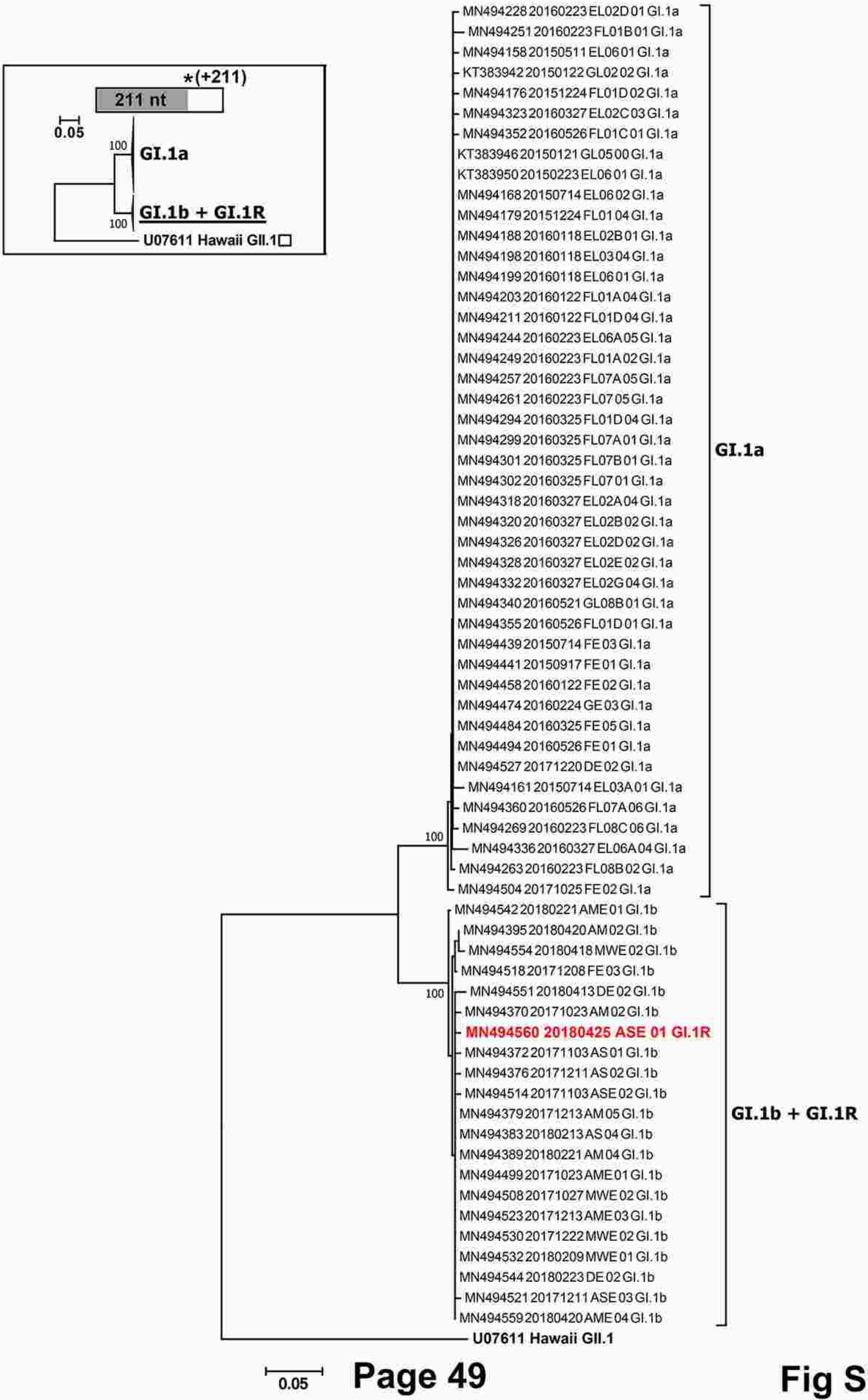

Page 49

Fig S8

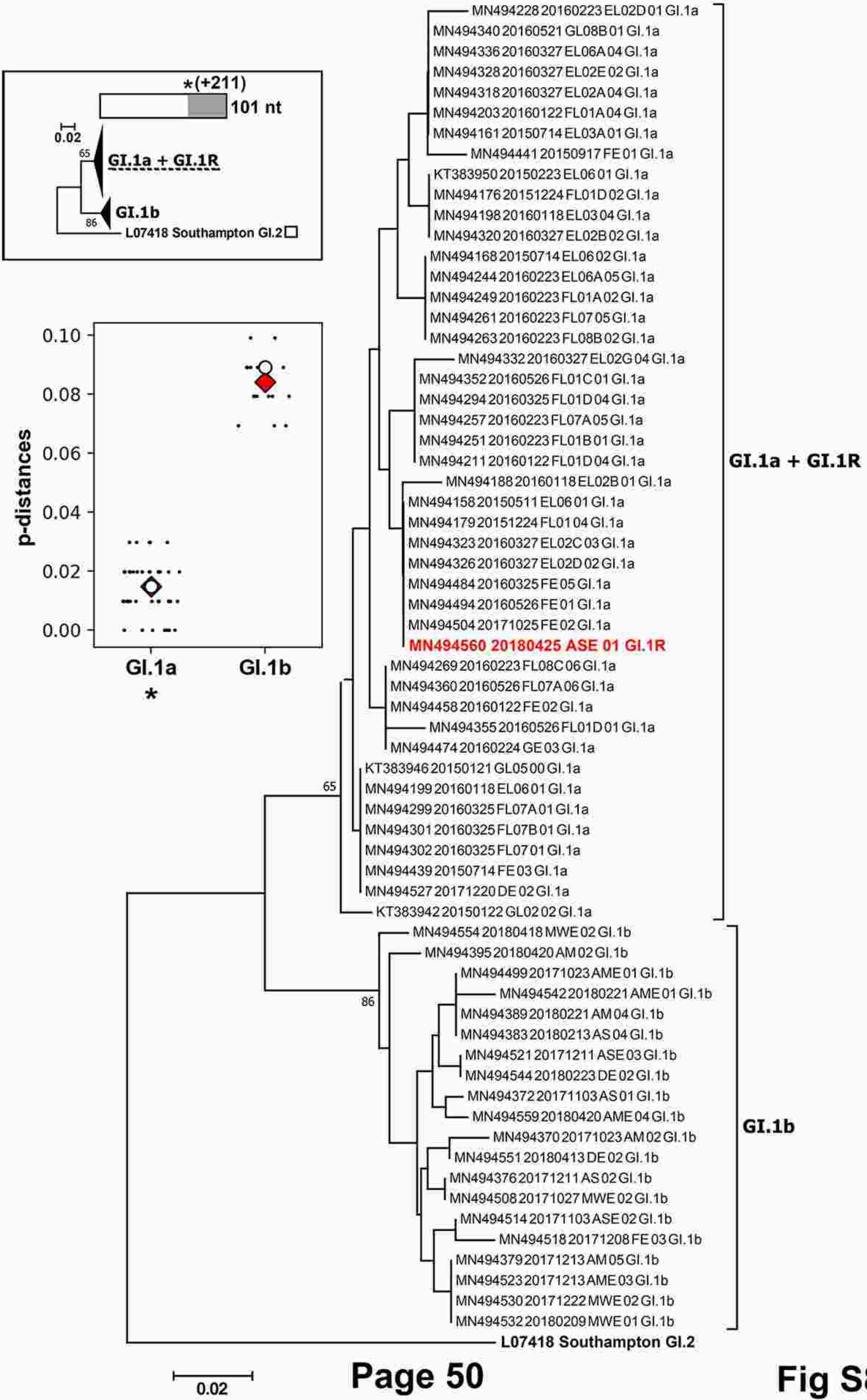
*(+211)
101 nt
0.02
65
GI.1a + GI.1R
GI.1b
86
L07418 Southampton GI.2
p-distances
0.10
0.08
0.06
0.04
0.02
0.00
GI.1a
*
GI.1b
MN494228 20160223 EL02D 01 GI.1a
MN494340 20160521 GL08B 01 GI.1a
MN494336 20160327 EL06A 04 GI.1a
MN494328 20160327 EL02E 02 GI.1a
MN494318 20160327 EL02A 04 GI.1a
MN494203 20160122 FL01A 04 GI.1a
MN494161 20150714 EL03A 01 GI.1a
MN494441 20150917 FE 01 GI.1a
KT383950 20150223 EL06 01 GI.1a
MN494176 20151224 FL01D 02 GI.1a
MN494198 20160118 EL03 04 GI.1a
MN494320 20160327 EL02B 02 GI.1a
MN494168 20150714 EL06 02 GI.1a
MN494244 20160223 EL06A 05 GI.1a
MN494249 20160223 FL01A 02 GI.1a
MN494261 20160223 FL07 05 GI.1a
MN494263 20160223 FL08B 02 GI.1a
MN494332 20160327 EL02G 04 GI.1a
MN494352 20160526 FL01C 01 GI.1a
MN494294 20160325 FL01D 04 GI.1a
MN494257 20160223 FL07A 05 GI.1a
MN494251 20160223 FL01B 01 GI.1a
MN494211 20160122 FL01D 04 GI.1a
MN494188 20160118 EL02B 01 GI.1a
MN494158 20150511 EL06 01 GI.1a
MN494179 20151224 FL01 04 GI.1a
MN494323 20160327 EL02C 03 GI.1a
MN494326 20160327 EL02D 02 GI.1a
MN494484 20160325 FE 05 GI.1a
MN494494 20160526 FE 01 GI.1a
MN494504 20171025 FE 02 GI.1a
MN494560 20180425 ASE 01 GI.1R
MN494269 20160223 FL08C 06 GI.1a
MN494360 20160526 FL07A 06 GI.1a
MN494458 20160122 FE 02 GI.1a
MN494355 20160526 FL01D 01 GI.1a
MN494474 20160224 GE 03 GI.1a
KT383946 20150121 GL05 00 GI.1a
MN494199 20160118 EL06 01 GI.1a
MN494299 20160325 FL07A 01 GI.1a
MN494301 20160325 FL07B 01 GI.1a
MN494302 20160325 FL07 01 GI.1a
MN494439 20150714 FE 03 GI.1a
MN494527 20171220 DE 02 GI.1a
KT383942 20150122 GL02 02 GI.1a
65
GI.1a + GI.1R
MN494554 20180418 MWE 02 GI.1b
MN494395 20180420 AM 02 GI.1b
MN494499 20171023 AME 01 GI.1b
MN494542 20180221 AME 01 GI.1b
MN494389 20180221 AM 04 GI.1b
MN494383 20180213 AS 04 GI.1b
MN494521 20171211 ASE 03 GI.1b
MN494544 20180223 DE 02 GI.1b
MN494372 20171103 AS 01 GI.1b
MN494559 20180420 AME 04 GI.1b
MN494370 20171023 AM 02 GI.1b
MN494551 20180413 DE 02 GI.1b
MN494376 20171211 AS 02 GI.1b
MN494508 20171027 MWE 02 GI.1b
MN494514 20171103 ASE 02 GI.1b
MN494518 20171208 FE 03 GI.1b
MN494379 20171213 AM 05 GI.1b
MN494523 20171213 AME 03 GI.1b
MN494530 20171222 MWE 02 GI.1b
MN494532 20180209 MWE 01 GI.1b
86
GI.1b
L07418 Southampton GI.2
0.02

Fig S8

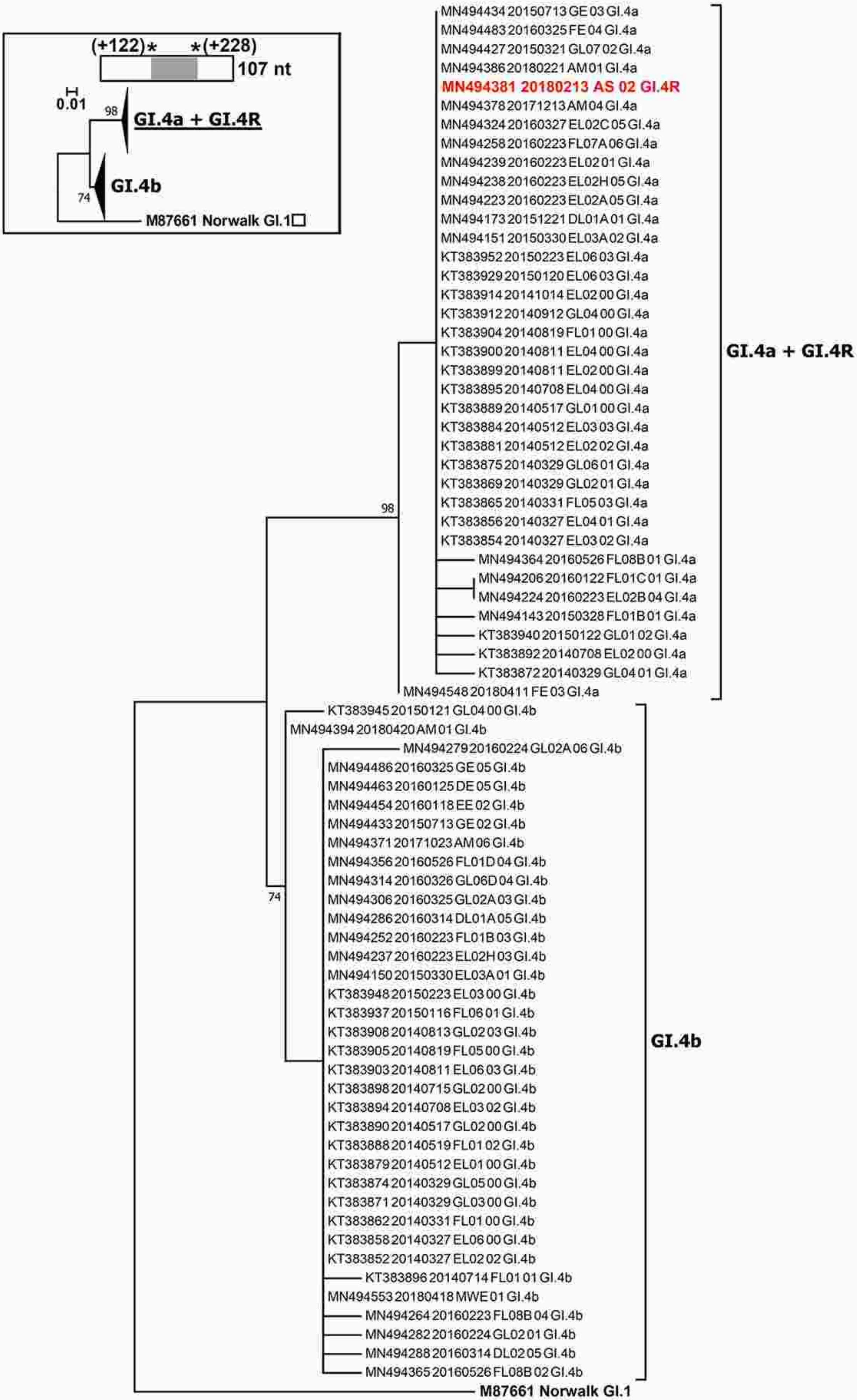

(+122)* *(+228)
107 nt
0.01
98
GI.4a + GI.4R
GI.4b
74
M87661 Norwalk GI.1
MN494434 20150713 GE 03 GI.4a
MN494483 20160325 FE 04 GI.4a
MN494427 20150321 GL07 02 GI.4a
MN494386 20180221 AM 01 GI.4a
MN494381 20180213 AS 02 GI.4R
MN494378 20171213 AM 04 GI.4a
MN494324 20160327 EL02C 05 GI.4a
MN494258 20160223 FL07A 06 GI.4a
MN494239 20160223 EL02 01 GI.4a
MN494238 20160223 EL02H 05 GI.4a
MN494223 20160223 EL02A 05 GI.4a
MN494173 20151221 DL01A 01 GI.4a
MN494151 20150330 EL03A 02 GI.4a
KT383952 20150223 EL06 03 GI.4a
KT383929 20150120 EL06 03 GI.4a
KT383914 20141014 EL02 00 GI.4a
KT383912 20140912 GL04 00 GI.4a
KT383904 20140819 FL01 00 GI.4a
KT383900 20140811 EL04 00 GI.4a
KT383899 20140811 EL02 00 GI.4a
KT383895 20140708 EL04 00 GI.4a
KT383889 20140517 GL01 00 GI.4a
KT383884 20140512 EL03 03 GI.4a
KT383881 20140512 EL02 02 GI.4a
KT383875 20140329 GL06 01 GI.4a
KT383869 20140329 GL02 01 GI.4a
KT383865 20140331 FL05 03 GI.4a
KT383856 20140327 EL04 01 GI.4a
KT383854 20140327 EL03 02 GI.4a
MN494364 20160526 FL08B 01 GI.4a
MN494206 20160122 FL01C 01 GI.4a
MN494224 20160223 EL02B 04 GI.4a
MN494143 20150328 FL01B 01 GI.4a
KT383940 20150122 GL01 02 GI.4a
KT383892 20140708 EL02 00 GI.4a
KT383872 20140329 GL04 01 GI.4a
MN494548 20180411 FE 03 GI.4a
98
GI.4a + GI.4R
KT383945 20150121 GL04 00 GI.4b
MN494394 20180420 AM 01 GI.4b
MN494279 20160224 GL02A 06 GI.4b
MN494486 20160325 GE 05 GI.4b
MN494463 20160125 DE 05 GI.4b
MN494454 20160118 EE 02 GI.4b
MN494433 20150713 GE 02 GI.4b
MN494371 20171023 AM 06 GI.4b
MN494356 20160526 FL01D 04 GI.4b
MN494314 20160326 GL06D 04 GI.4b
MN494306 20160325 GL02A 03 GI.4b
MN494286 20160314 DL01A 05 GI.4b
MN494252 20160223 FL01B 03 GI.4b
MN494237 20160223 EL02H 03 GI.4b
MN494150 20150330 EL03A 01 GI.4b
KT383948 20150223 EL03 00 GI.4b
KT383937 20150116 FL06 01 GI.4b
KT383908 20140813 GL02 03 GI.4b
KT383905 20140819 FL05 00 GI.4b
KT383903 20140811 EL06 03 GI.4b
KT383898 20140715 GL02 00 GI.4b
KT383894 20140708 EL03 02 GI.4b
KT383890 20140517 GL02 00 GI.4b
KT383888 20140519 FL01 02 GI.4b
KT383879 20140512 EL01 00 GI.4b
KT383874 20140329 GL05 00 GI.4b
KT383871 20140329 GL03 00 GI.4b
KT383862 20140331 FL01 00 GI.4b
KT383858 20140327 EL06 00 GI.4b
KT383852 20140327 EL02 02 GI.4b
KT383896 20140714 FL01 01 GI.4b
MN494553 20180418 MWE 01 GI.4b
MN494264 20160223 FL08B 04 GI.4b
MN494282 20160224 GL02 01 GI.4b
MN494288 20160314 DL02 05 GI.4b
MN494365 20160526 FL08B 02 GI.4b
74
GI.4b
M87661 Norwalk GI.1
0.01

Fig S8

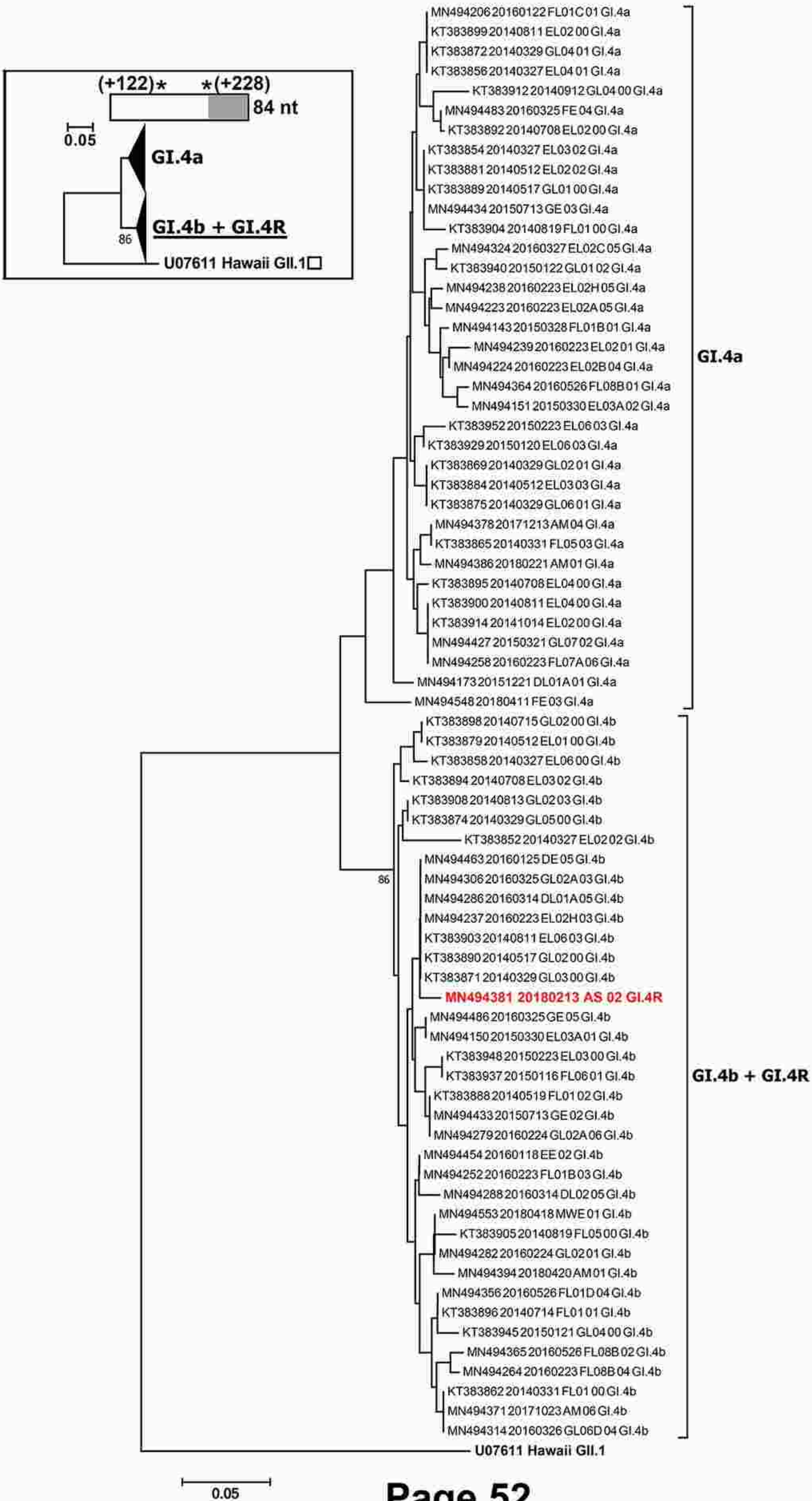

(+122)*
*(+228)
84 nt
0.05
GI.4a
GI.4b + GI.4R
86
U07611 Hawaii GII.1
MN494206 20160122 FL01C 01 GI.4a
KT383899 20140811 EL02 00 GI.4a
KT383872 20140329 GL04 01 GI.4a
KT383856 20140327 EL04 01 GI.4a
KT383912 20140912 GL04 00 GI.4a
MN494483 20160325 FE 04 GI.4a
KT383892 20140708 EL02 00 GI.4a
KT383854 20140327 EL03 02 GI.4a
KT383881 20140512 EL02 02 GI.4a
KT383889 20140517 GL01 00 GI.4a
MN494434 20150713 GE 03 GI.4a
KT383904 20140819 FL01 00 GI.4a
MN494324 20160327 EL02C 05 GI.4a
KT383940 20150122 GL01 02 GI.4a
MN494238 20160223 EL02H 05 GI.4a
MN494223 20160223 EL02A 05 GI.4a
MN494143 20150328 FL01B 01 GI.4a
MN494239 20160223 EL02 01 GI.4a
MN494224 20160223 EL02B 04 GI.4a
MN494364 20160526 FL08B 01 GI.4a
MN494151 20150330 EL03A 02 GI.4a
KT383952 20150223 EL06 03 GI.4a
KT383929 20150120 EL06 03 GI.4a
KT383869 20140329 GL02 01 GI.4a
KT383884 20140512 EL03 03 GI.4a
KT383875 20140329 GL06 01 GI.4a
MN494378 20171213 AM 04 GI.4a
KT383865 20140331 FL05 03 GI.4a
MN494386 20180221 AM 01 GI.4a
KT383895 20140708 EL04 00 GI.4a
KT383900 20140811 EL04 00 GI.4a
KT383914 20141014 EL02 00 GI.4a
MN494427 20150321 GL07 02 GI.4a
MN494258 20160223 FL07A 06 GI.4a
MN494173 20151221 DL01A 01 GI.4a
MN494548 20180411 FE 03 GI.4a
GI.4a
KT383898 20140715 GL02 00 GI.4b
KT383879 20140512 EL01 00 GI.4b
KT383858 20140327 EL06 00 GI.4b
KT383894 20140708 EL03 02 GI.4b
KT383908 20140813 GL02 03 GI.4b
KT383874 20140329 GL05 00 GI.4b
KT383852 20140327 EL02 02 GI.4b
MN494463 20160125 DE 05 GI.4b
MN494306 20160325 GL02A 03 GI.4b
86
MN494286 20160314 DL01A 05 GI.4b
MN494237 20160223 EL02H 03 GI.4b
KT383903 20140811 EL06 03 GI.4b
KT383890 20140517 GL02 00 GI.4b
KT383871 20140329 GL03 00 GI.4b
MN494381 20180213 AS 02 GI.4R
MN494486 20160325 GE 05 GI.4b
MN494150 20150330 EL03A 01 GI.4b
KT383948 20150223 EL03 00 GI.4b
KT383937 20150116 FL06 01 GI.4b
KT383888 20140519 FL01 02 GI.4b
MN494433 20150713 GE 02 GI.4b
MN494279 20160224 GL02A 06 GI.4b
MN494454 20160118 EE 02 GI.4b
MN494252 20160223 FL01B 03 GI.4b
MN494288 20160314 DL02 05 GI.4b
MN494553 20180418 MWE 01 GI.4b
KT383905 20140819 FL05 00 GI.4b
MN494282 20160224 GL02 01 GI.4b
MN494394 20180420 AM 01 GI.4b
MN494356 20160526 FL01D 04 GI.4b
KT383896 20140714 FL01 01 GI.4b
KT383945 20150121 GL04 00 GI.4b
MN494365 20160526 FL08B 02 GI.4b
MN494264 20160223 FL08B 04 GI.4b
KT383862 20140331 FL01 00 GI.4b
MN494371 20171023 AM 06 GI.4b
MN494314 20160326 GL06D 04 GI.4b
GI.4b + GI.4R
U07611 Hawaii GII.1
0.05

Fig S8

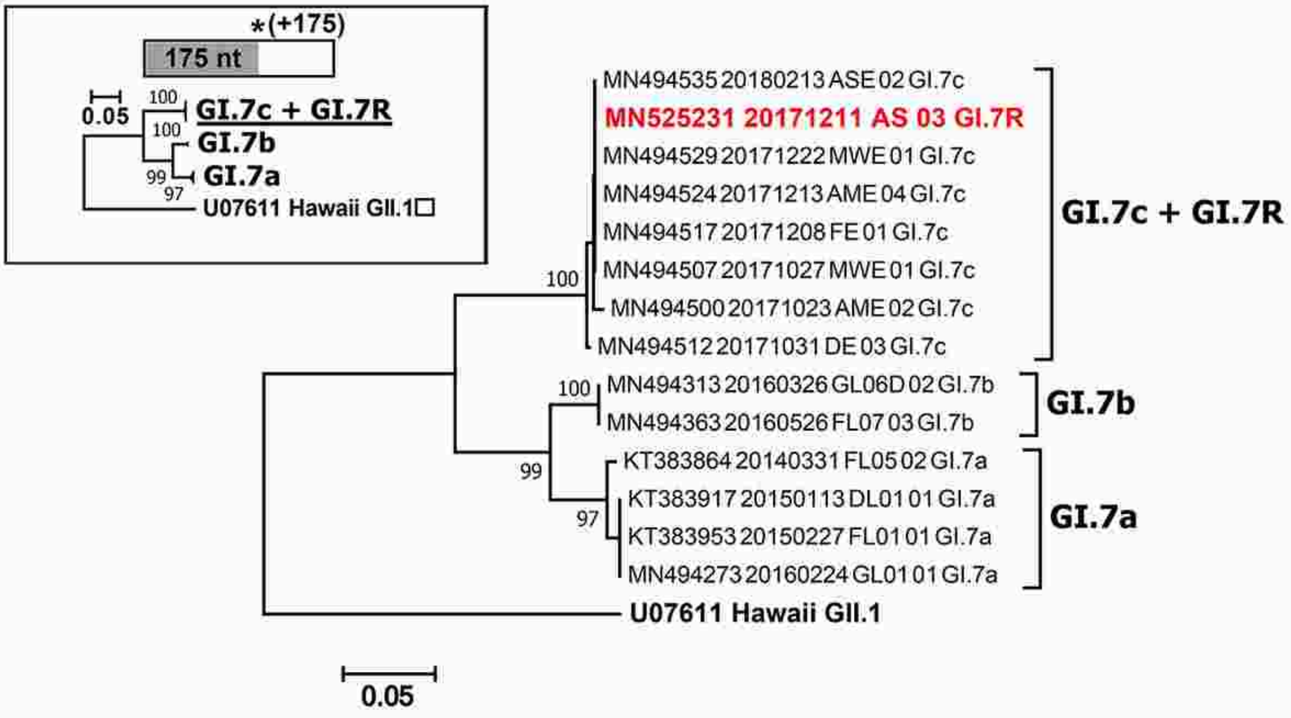
*(+175)
175 nt
0.05
100
GI.7c + GI.7R
100
GI.7b
99
GI.7a
97
U07611 Hawaii GII.1
MN494535 20180213 ASE 02 GI.7c
MN525231 20171211 AS 03 GI.7R
MN494529 20171222 MWE 01 GI.7c
MN494524 20171213 AME 04 GI.7c
MN494517 20171208 FE 01 GI.7c
MN494507 20171027 MWE 01 GI.7c
MN494500 20171023 AME 02 GI.7c
MN494512 20171031 DE 03 GI.7c
GI.7c + GI.7R
100
MN494313 20160326 GL06D 02 GI.7b
MN494363 20160526 FL07 03 GI.7b
GI.7b
99
KT383864 20140331 FL05 02 GI.7a
KT383917 20150113 DL01 01 GI.7a
97
KT383953 20150227 FL01 01 GI.7a
MN494273 20160224 GL01 01 GI.7a
GI.7a
U07611 Hawaii GII.1
0.05

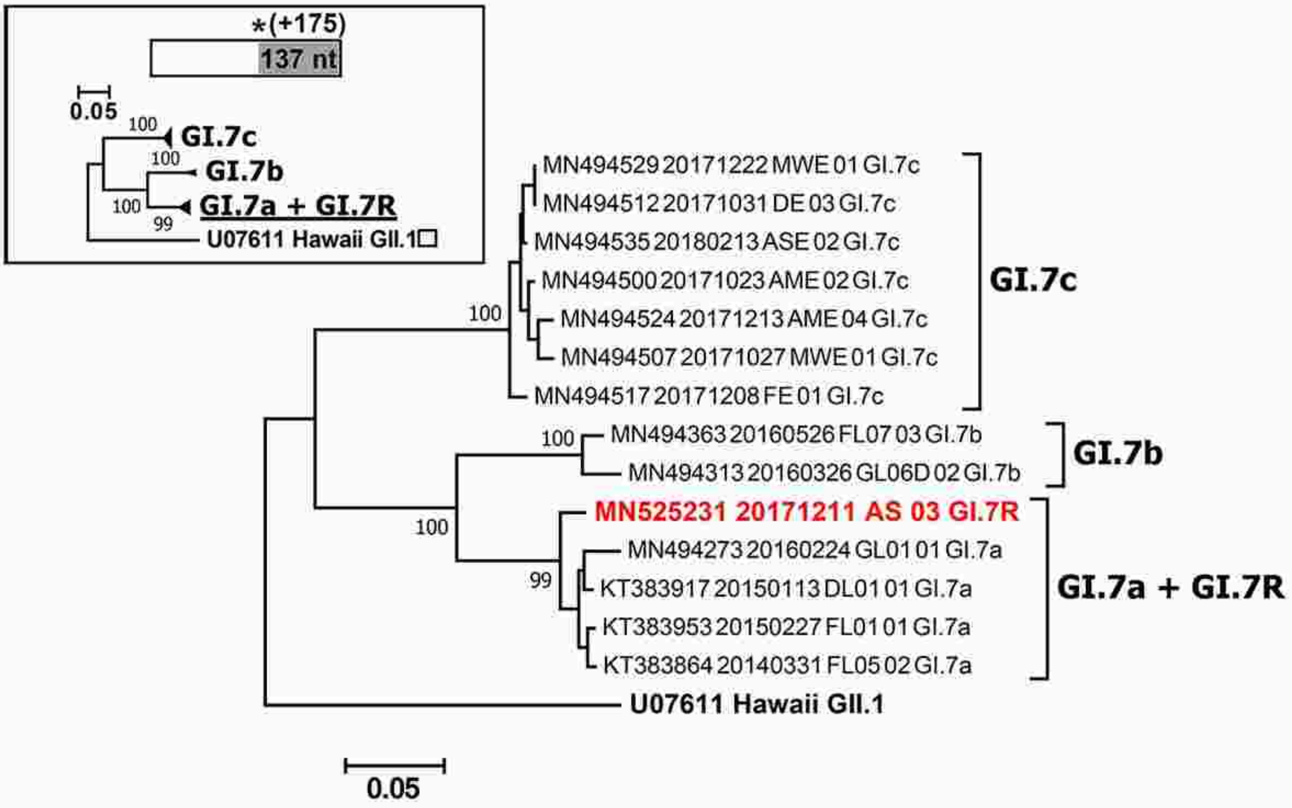
*(+175)
137 nt
0.05
100
GI.7c
100
GI.7b
100
GI.7a + GI.7R
99
U07611 Hawaii GII.1
MN494529 20171222 MWE 01 GI.7c
MN494512 20171031 DE 03 GI.7c
MN494535 20180213 ASE 02 GI.7c
MN494500 20171023 AME 02 GI.7c
MN494524 20171213 AME 04 GI.7c
MN494507 20171027 MWE 01 GI.7c
MN494517 20171208 FE 01 GI.7c
GI.7c
100
MN494363 20160526 FL07 03 GI.7b
MN494313 20160326 GL06D 02 GI.7b
GI.7b
100
MN525231 20171211 AS 03 GI.7R
MN494273 20160224 GL01 01 GI.7a
99
KT383917 20150113 DL01 01 GI.7a
KT383953 20150227 FL01 01 GI.7a
KT383864 20140331 FL05 02 GI.7a
GI.7a + GI.7R
U07611 Hawaii GII.1
0.05

Fig S8

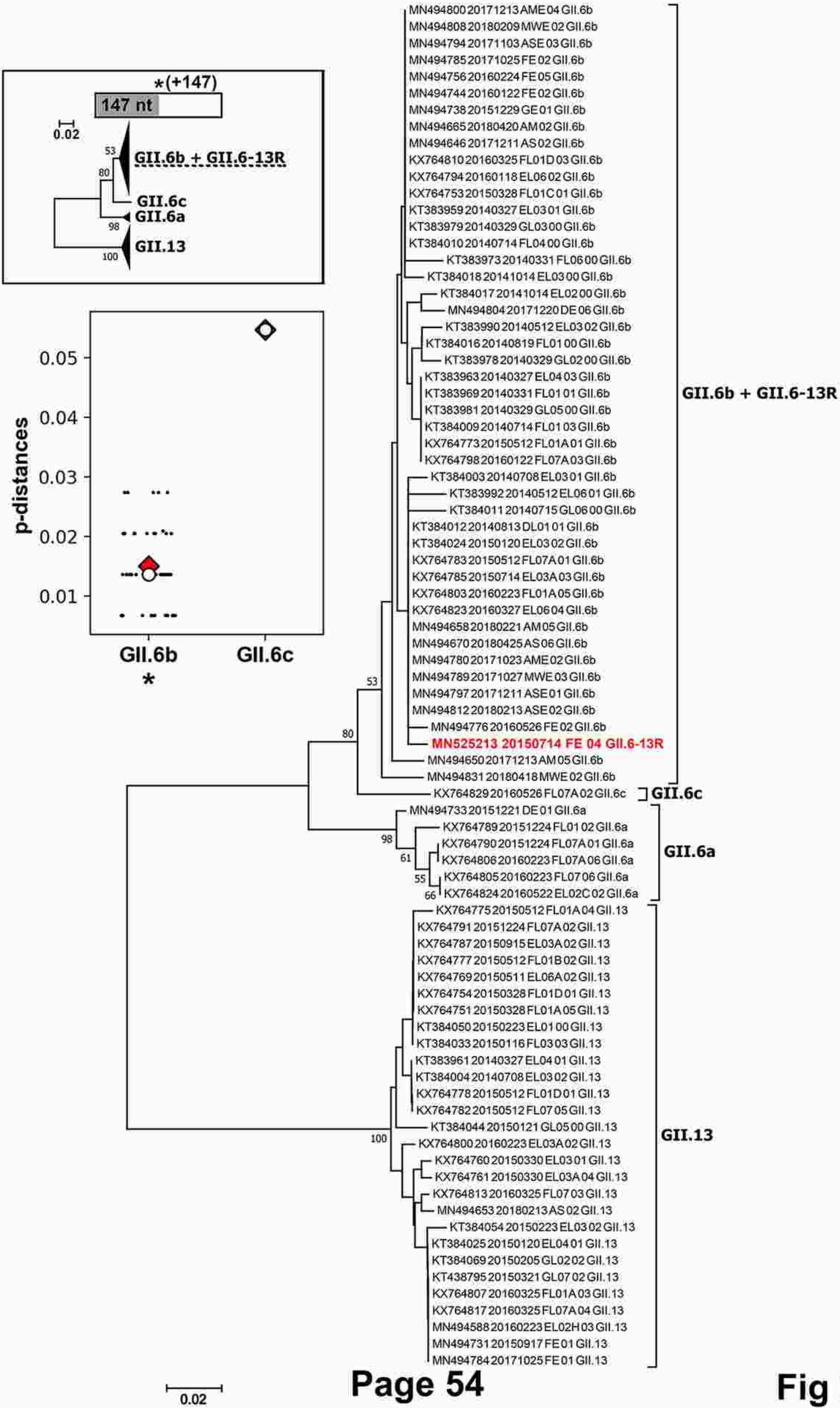
*(+147)
147 nt
0.02
53
GII.6b + GII.6-13R
80
GII.6c
GII.6a
98
GII.13
100
p-distances
0.05
0.04
0.03
0.02
0.01
GII.6b
*
GII.6c
MN494800 20171213 AME 04 GII.6b
MN494808 20180209 MWE 02 GII.6b
MN494794 20171103 ASE 03 GII.6b
MN494785 20171025 FE 02 GII.6b
MN494756 20160224 FE 05 GII.6b
MN494744 20160122 FE 02 GII.6b
MN494738 20151229 GE 01 GII.6b
MN494665 20180420 AM 02 GII.6b
MN494646 20171211 AS 02 GII.6b
KX764810 20160325 FL01D 03 GII.6b
KX764794 20160118 EL06 02 GII.6b
KX764753 20150328 FL01C 01 GII.6b
KT383959 20140327 EL03 01 GII.6b
KT383979 20140329 GL03 00 GII.6b
KT384010 20140714 FL04 00 GII.6b
KT383973 20140331 FL06 00 GII.6b
KT384018 20141014 EL03 00 GII.6b
KT384017 20141014 EL02 00 GII.6b
MN494804 20171220 DE 06 GII.6b
KT383990 20140512 EL03 02 GII.6b
KT384016 20140819 FL01 00 GII.6b
KT383978 20140329 GL02 00 GII.6b
KT383963 20140327 EL04 03 GII.6b
KT383969 20140331 FL01 01 GII.6b
KT383981 20140329 GL05 00 GII.6b
KT384009 20140714 FL01 03 GII.6b
KX764773 20150512 FL01A 01 GII.6b
KX764798 20160122 FL07A 03 GII.6b
KT384003 20140708 EL03 01 GII.6b
KT383992 20140512 EL06 01 GII.6b
KT384011 20140715 GL06 00 GII.6b
KT384012 20140813 DL01 01 GII.6b
KT384024 20150120 EL03 02 GII.6b
KX764783 20150512 FL07A 01 GII.6b
KX764785 20150714 EL03A 03 GII.6b
KX764803 20160223 FL01A 05 GII.6b
KX764823 20160327 EL06 04 GII.6b
MN494658 20180221 AM 05 GII.6b
MN494670 20180425 AS 06 GII.6b
MN494780 20171023 AME 02 GII.6b
MN494789 20171027 MWE 03 GII.6b
MN494797 20171211 ASE 01 GII.6b
MN494812 20180213 ASE 02 GII.6b
MN494776 20160526 FE 02 GII.6b
MN525213 20150714 FE 04 GII.6-13R
MN494650 20171213 AM 05 GII.6b
MN494831 20180418 MWE 02 GII.6b
KX764829 20160526 FL07A 02 GII.6c
MN494733 20151221 DE 01 GII.6a
KX764789 20151224 FL01 02 GII.6a
KX764790 20151224 FL07A 01 GII.6a
KX764806 20160223 FL07A 06 GII.6a
KX764805 20160223 FL07 06 GII.6a
KX764824 20160522 EL02C 02 GII.6a
61
55
66
KX764775 20150512 FL01A 04 GII.13
KX764791 20151224 FL07A 02 GII.13
KX764787 20150915 EL03A 02 GII.13
KX764777 20150512 FL01B 02 GII.13
KX764769 20150511 EL06A 02 GII.13
KX764754 20150328 FL01D 01 GII.13
KX764751 20150328 FL01A 05 GII.13
KT384050 20150223 EL01 00 GII.13
KT384033 20150116 FL03 03 GII.13
KT383961 20140327 EL04 01 GII.13
KT384004 20140708 EL03 02 GII.13
KX764778 20150512 FL01D 01 GII.13
KX764782 20150512 FL07 05 GII.13
KT384044 20150121 GL05 00 GII.13
KX764800 20160223 EL03A 02 GII.13
KX764760 20150330 EL03 01 GII.13
KX764761 20150330 EL03A 04 GII.13
KX764813 20160325 FL07 03 GII.13
MN494653 20180213 AS 02 GII.13
KT384054 20150223 EL03 02 GII.13
KT384025 20150120 EL04 01 GII.13
KT384069 20150205 GL02 02 GII.13
KT438795 20150321 GL07 02 GII.13
KX764807 20160325 FL01A 03 GII.13
KX764817 20160325 FL07A 04 GII.13
MN494588 20160223 EL02H 03 GII.13
MN494731 20150917 FE 01 GII.13
MN494784 20171025 FE 01 GII.13
GII.6b + GII.6-13R
GII.6c
GII.6a
GII.13
0.02

Fig S8

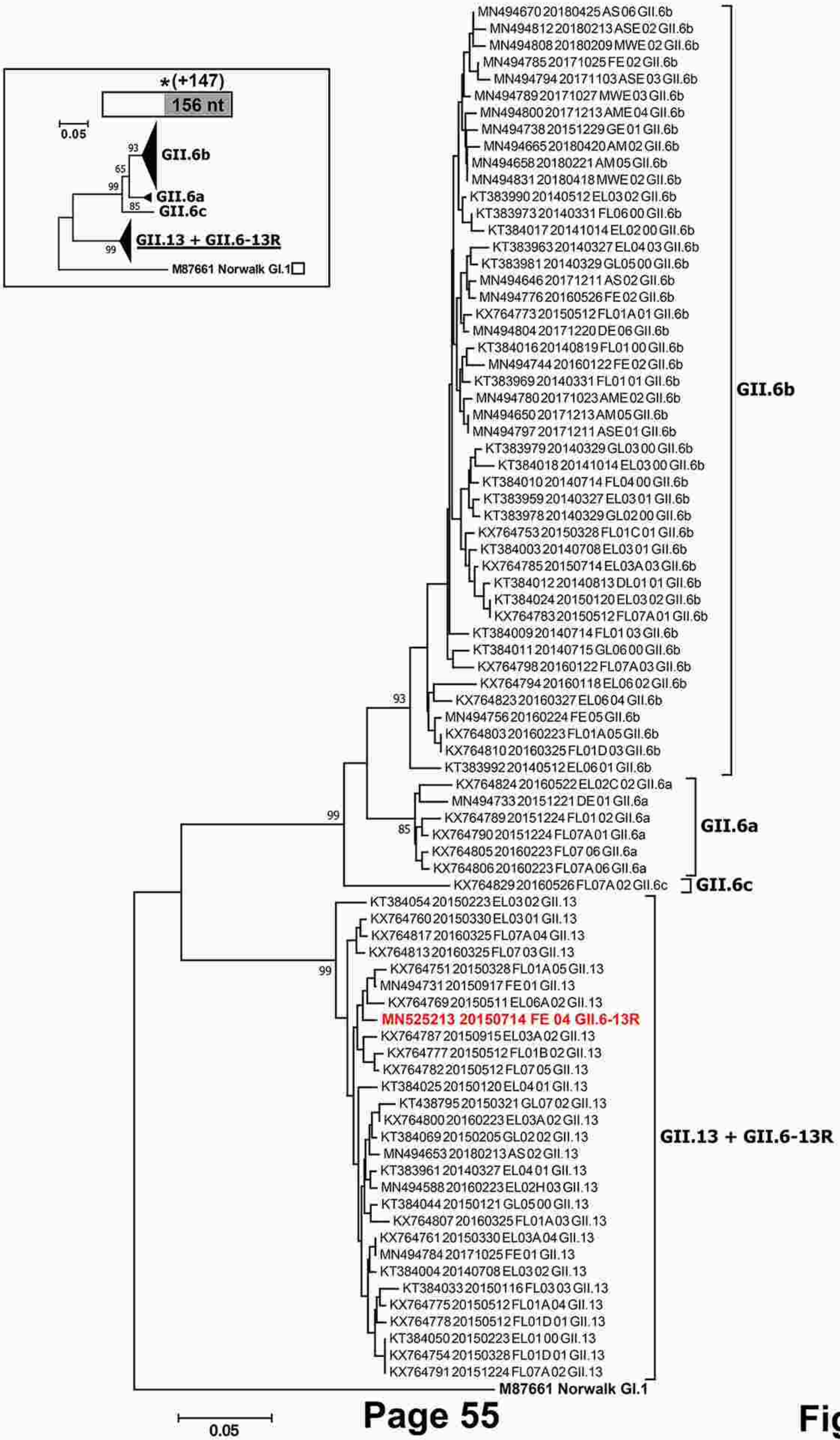

*(+147)
156 nt
0.05
93
GII.6b
65
99
GII.6a
85
GII.6c
99
GII.13 + GII.6-13R
M87661 Norwalk GI.1
MN494670 20180425 AS 06 GII.6b
MN494812 20180213 ASE 02 GII.6b
MN494808 20180209 MWE 02 GII.6b
MN494785 20171025 FE 02 GII.6b
MN494794 20171103 ASE 03 GII.6b
MN494789 20171027 MWE 03 GII.6b
MN494800 20171213 AME 04 GII.6b
MN494738 20151229 GE 01 GII.6b
MN494665 20180420 AM 02 GII.6b
MN494658 20180221 AM 05 GII.6b
MN494831 20180418 MWE 02 GII.6b
KT383990 20140512 EL03 02 GII.6b
KT383973 20140331 FL06 00 GII.6b
KT384017 20141014 EL02 00 GII.6b
KT383963 20140327 EL04 03 GII.6b
KT383981 20140329 GL05 00 GII.6b
MN494646 20171211 AS 02 GII.6b
MN494776 20160526 FE 02 GII.6b
KX764773 20150512 FL01A 01 GII.6b
MN494804 20171220 DE 06 GII.6b
KT384016 20140819 FL01 00 GII.6b
MN494744 20160122 FE 02 GII.6b
KT383969 20140331 FL01 01 GII.6b
MN494780 20171023 AME 02 GII.6b
MN494650 20171213 AM 05 GII.6b
MN494797 20171211 ASE 01 GII.6b
KT383979 20140329 GL03 00 GII.6b
KT384018 20141014 EL03 00 GII.6b
KT384010 20140714 FL04 00 GII.6b
KT383959 20140327 EL03 01 GII.6b
KT383978 20140329 GL02 00 GII.6b
KX764753 20150328 FL01C 01 GII.6b
KT384003 20140708 EL03 01 GII.6b
KX764785 20150714 EL03A 03 GII.6b
KT384012 20140813 DL01 01 GII.6b
KT384024 20150120 EL03 02 GII.6b
KX764783 20150512 FL07A 01 GII.6b
KT384009 20140714 FL01 03 GII.6b
KT384011 20140715 GL06 00 GII.6b
KX764798 20160122 FL07A 03 GII.6b
KX764794 20160118 EL06 02 GII.6b
KX764823 20160327 EL06 04 GII.6b
MN494756 20160224 FE 05 GII.6b
KX764803 20160223 FL01A 05 GII.6b
KX764810 20160325 FL01D 03 GII.6b
KT383992 20140512 EL06 01 GII.6b
GII.6b
93
KX764824 20160522 EL02C 02 GII.6a
MN494733 20151221 DE 01 GII.6a
KX764789 20151224 FL01 02 GII.6a
KX764790 20151224 FL07A 01 GII.6a
KX764805 20160223 FL07 06 GII.6a
KX764806 20160223 FL07A 06 GII.6a
85
99
GII.6a
KX764829 20160526 FL07A 02 GII.6c
GII.6c
KT384054 20150223 EL03 02 GII.13
KX764760 20150330 EL03 01 GII.13
KX764817 20160325 FL07A 04 GII.13
KX764813 20160325 FL07 03 GII.13
99
KX764751 20150328 FL01A 05 GII.13
MN494731 20150917 FE 01 GII.13
KX764769 20150511 EL06A 02 GII.13
MN525213 20150714 FE 04 GII.6-13R
KX764787 20150915 EL03A 02 GII.13
KX764777 20150512 FL01B 02 GII.13
KX764782 20150512 FL07 05 GII.13
KT384025 20150120 EL04 01 GII.13
KT438795 20150321 GL07 02 GII.13
KX764800 20160223 EL03A 02 GII.13
KT384069 20150205 GL02 02 GII.13
MN494653 20180213 AS 02 GII.13
KT383961 20140327 EL04 01 GII.13
MN494588 20160223 EL02H 03 GII.13
KT384044 20150121 GL05 00 GII.13
KX764807 20160325 FL01A 03 GII.13
KX764761 20150330 EL03A 04 GII.13
MN494784 20171025 FE 01 GII.13
KT384004 20140708 EL03 02 GII.13
KT384033 20150116 FL03 03 GII.13
KX764775 20150512 FL01A 04 GII.13
KX764778 20150512 FL01D 01 GII.13
KT384050 20150223 EL01 00 GII.13
KX764754 20150328 FL01D 01 GII.13
KX764791 20151224 FL07A 02 GII.13
GII.13 + GII.6-13R
M87661 Norwalk GI.1
0.05

Fig S8

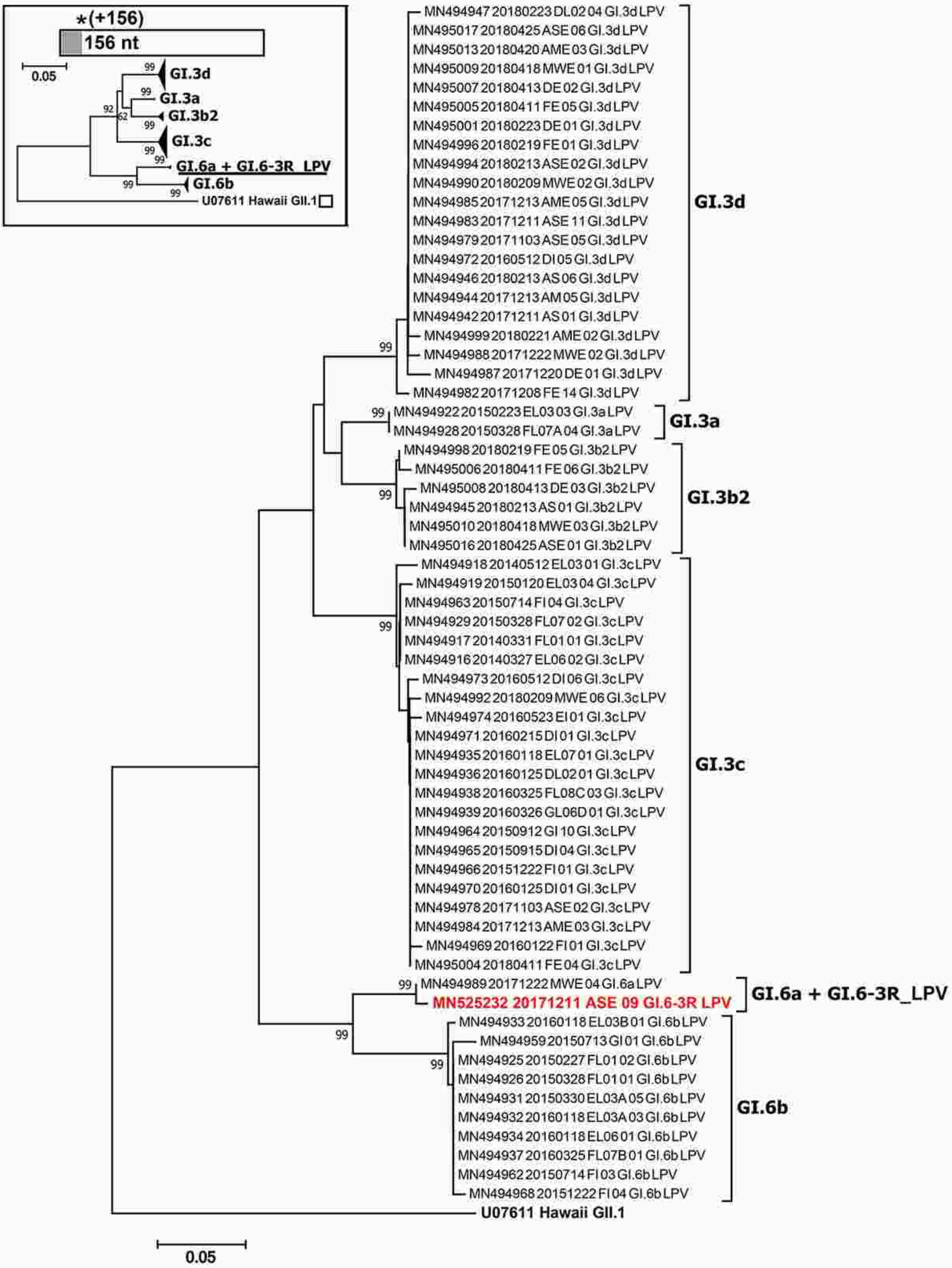
*(+156)
156 nt
0.05
99
GI.3d
99
GI.3a
92
62
GI.3b2
99
GI.3c
99
99
GI.6a + GI.6-3R_LPV
99
GI.6b
99
U07611 Hawaii GII.1
MN494947 20180223 DL02 04 GI.3d LPV
MN495017 20180425 ASE 06 GI.3d LPV
MN495013 20180420 AME 03 GI.3d LPV
MN495009 20180418 MWE 01 GI.3d LPV
MN495007 20180413 DE 02 GI.3d LPV
MN495005 20180411 FE 05 GI.3d LPV
MN495001 20180223 DE 01 GI.3d LPV
MN494996 20180219 FE 01 GI.3d LPV
MN494994 20180213 ASE 02 GI.3d LPV
MN494990 20180209 MWE 02 GI.3d LPV
MN494985 20171213 AME 05 GI.3d LPV
MN494983 20171211 ASE 11 GI.3d LPV
MN494979 20171103 ASE 05 GI.3d LPV
MN494972 20160512 DI 05 GI.3d LPV
MN494946 20180213 AS 06 GI.3d LPV
MN494944 20171213 AM 05 GI.3d LPV
MN494942 20171211 AS 01 GI.3d LPV
MN494999 20180221 AME 02 GI.3d LPV
MN494988 20171222 MWE 02 GI.3d LPV
MN494987 20171220 DE 01 GI.3d LPV
MN494982 20171208 FE 14 GI.3d LPV
99
GI.3d
MN494922 20150223 EL03 03 GI.3a LPV
MN494928 20150328 FL07A 04 GI.3a LPV
99
GI.3a
MN494998 20180219 FE 05 GI.3b2 LPV
MN495006 20180411 FE 06 GI.3b2 LPV
MN495008 20180413 DE 03 GI.3b2 LPV
MN494945 20180213 AS 01 GI.3b2 LPV
MN495010 20180418 MWE 03 GI.3b2 LPV
MN495016 20180425 ASE 01 GI.3b2 LPV
99
GI.3b2
MN494918 20140512 EL03 01 GI.3c LPV
MN494919 20150120 EL03 04 GI.3c LPV
MN494963 20150714 FI 04 GI.3c LPV
MN494929 20150328 FL07 02 GI.3c LPV
MN494917 20140331 FL01 01 GI.3c LPV
MN494916 20140327 EL06 02 GI.3c LPV
MN494973 20160512 DI 06 GI.3c LPV
MN494992 20180209 MWE 06 GI.3c LPV
MN494974 20160523 EI 01 GI.3c LPV
MN494971 20160215 DI 01 GI.3c LPV
MN494935 20160118 EL07 01 GI.3c LPV
MN494936 20160125 DL02 01 GI.3c LPV
MN494938 20160325 FL08C 03 GI.3c LPV
MN494939 20160326 GL06D 01 GI.3c LPV
MN494964 20150912 GI 10 GI.3c LPV
MN494965 20150915 DI 04 GI.3c LPV
MN494966 20151222 FI 01 GI.3c LPV
MN494970 20160125 DI 01 GI.3c LPV
MN494978 20171103 ASE 02 GI.3c LPV
MN494984 20171213 AME 03 GI.3c LPV
MN494969 20160122 FI 01 GI.3c LPV
MN495004 20180411 FE 04 GI.3c LPV
99
GI.3c
MN494989 20171222 MWE 04 GI.6a LPV
MN525232 20171211 ASE 09 GI.6-3R LPV
99
GI.6a + GI.6-3R_LPV
99
MN494933 20160118 EL03B 01 GI.6b LPV
MN494959 20150713 GI 01 GI.6b LPV
MN494925 20150227 FL01 02 GI.6b LPV
MN494926 20150328 FL01 01 GI.6b LPV
MN494931 20150330 EL03A 05 GI.6b LPV
MN494932 20160118 EL03A 03 GI.6b LPV
MN494934 20160118 EL06 01 GI.6b LPV
MN494937 20160325 FL07B 01 GI.6b LPV
MN494962 20150714 FI 03 GI.6b LPV
MN494968 20151222 FI 04 GI.6b LPV
99
GI.6b
U07611 Hawaii GII.1
0.05

Fig S8

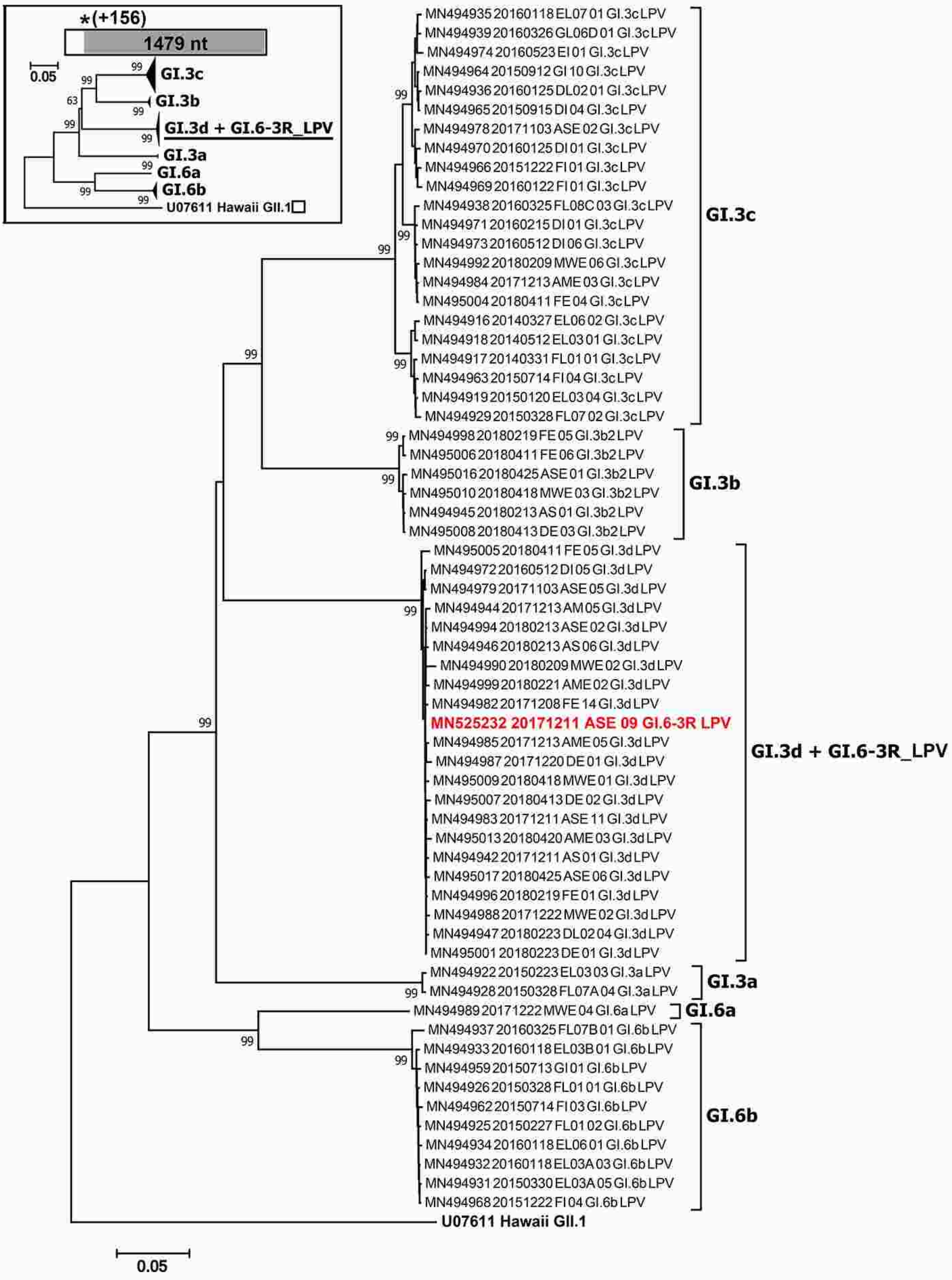
*(+156)
1479 nt
0.05
GI.3c
GI.3b
GI.3d + GI.6-3R_LPV
GI.3a
GI.6a
GI.6b
U07611 Hawaii GII.1
MN494935 20160118 EL07 01 GI.3c LPV
MN494939 20160326 GL06D 01 GI.3c LPV
MN494974 20160523 EI 01 GI.3c LPV
MN494964 20150912 GI 10 GI.3c LPV
MN494936 20160125 DL02 01 GI.3c LPV
MN494965 20150915 DI 04 GI.3c LPV
MN494978 20171103 ASE 02 GI.3c LPV
MN494970 20160125 DI 01 GI.3c LPV
MN494966 20151222 FI 01 GI.3c LPV
MN494969 20160122 FI 01 GI.3c LPV
MN494938 20160325 FL08C 03 GI.3c LPV
MN494971 20160215 DI 01 GI.3c LPV
MN494973 20160512 DI 06 GI.3c LPV
MN494992 20180209 MWE 06 GI.3c LPV
MN494984 20171213 AME 03 GI.3c LPV
MN495004 20180411 FE 04 GI.3c LPV
MN494916 20140327 EL06 02 GI.3c LPV
MN494918 20140512 EL03 01 GI.3c LPV
MN494917 20140331 FL01 01 GI.3c LPV
MN494963 20150714 FI 04 GI.3c LPV
MN494919 20150120 EL03 04 GI.3c LPV
MN494929 20150328 FL07 02 GI.3c LPV
GI.3c
MN494998 20180219 FE 05 GI.3b2 LPV
MN495006 20180411 FE 06 GI.3b2 LPV
MN495016 20180425 ASE 01 GI.3b2 LPV
MN495010 20180418 MWE 03 GI.3b2 LPV
MN494945 20180213 AS 01 GI.3b2 LPV
MN495008 20180413 DE 03 GI.3b2 LPV
GI.3b
MN495005 20180411 FE 05 GI.3d LPV
MN494972 20160512 DI 05 GI.3d LPV
MN494979 20171103 ASE 05 GI.3d LPV
MN494944 20171213 AM 05 GI.3d LPV
MN494994 20180213 ASE 02 GI.3d LPV
MN494946 20180213 AS 06 GI.3d LPV
MN494990 20180209 MWE 02 GI.3d LPV
MN494999 20180221 AME 02 GI.3d LPV
MN494982 20171208 FE 14 GI.3d LPV
MN525232 20171211 ASE 09 GI.6-3R LPV
MN494985 20171213 AME 05 GI.3d LPV
MN494987 20171220 DE 01 GI.3d LPV
MN495009 20180418 MWE 01 GI.3d LPV
MN495007 20180413 DE 02 GI.3d LPV
MN494983 20171211 ASE 11 GI.3d LPV
MN495013 20180420 AME 03 GI.3d LPV
MN494942 20171211 AS 01 GI.3d LPV
MN495017 20180425 ASE 06 GI.3d LPV
MN494996 20180219 FE 01 GI.3d LPV
MN494988 20171222 MWE 02 GI.3d LPV
MN494947 20180223 DL02 04 GI.3d LPV
MN495001 20180223 DE 01 GI.3d LPV
GI.3d + GI.6-3R_LPV
MN494922 20150223 EL03 03 GI.3a LPV
MN494928 20150328 FL07A 04 GI.3a LPV
GI.3a
MN494989 20171222 MWE 04 GI.6a LPV
GI.6a
MN494937 20160325 FL07B 01 GI.6b LPV
MN494933 20160118 EL03B 01 GI.6b LPV
MN494959 20150713 GI 01 GI.6b LPV
MN494926 20150328 FL01 01 GI.6b LPV
MN494962 20150714 FI 03 GI.6b LPV
MN494925 20150227 FL01 02 GI.6b LPV
MN494934 20160118 EL06 01 GI.6b LPV
MN494932 20160118 EL03A 03 GI.6b LPV
MN494931 20150330 EL03A 05 GI.6b LPV
MN494968 20151222 FI 04 GI.6b LPV
GI.6b
U07611 Hawaii GII.1
0.05

Fig S8

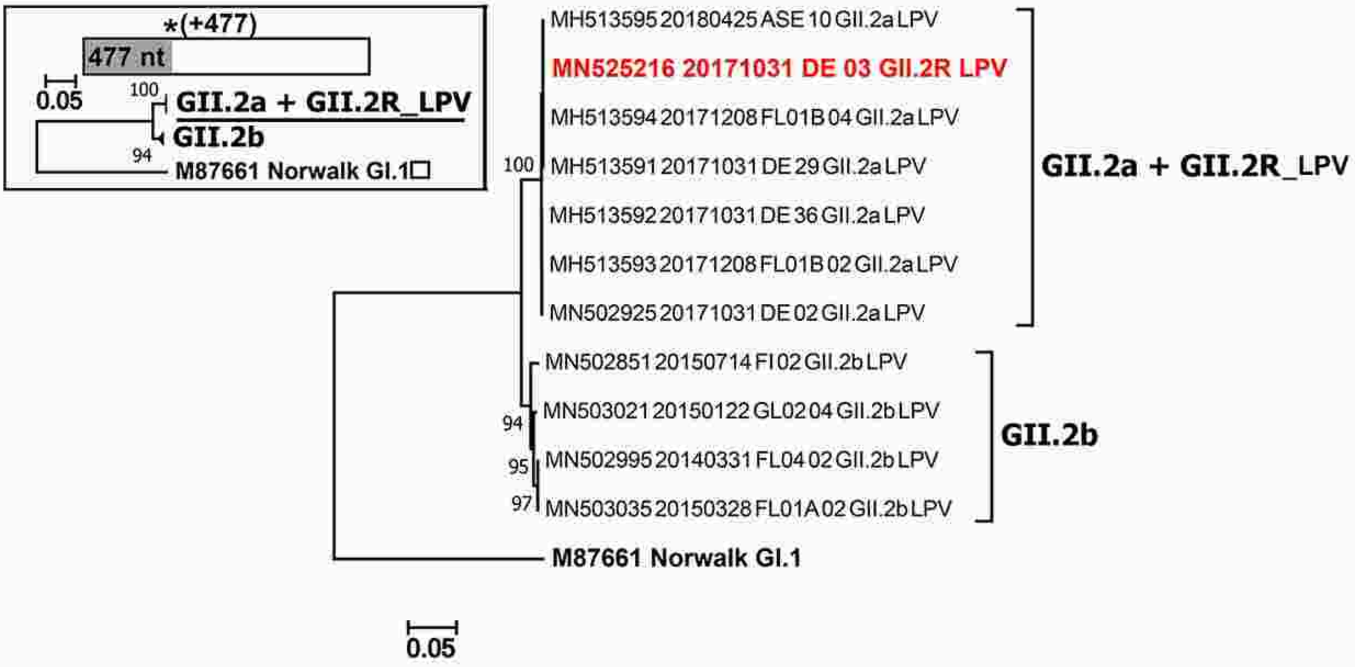
*(+477)
477 nt
0.05
100
GII.2a + GII.2R_LPV
GII.2b
94
M87661 Norwalk GI.1
MH513595 20180425 ASE 10 GII.2a LPV
MN525216 20171031 DE 03 GII.2R LPV
MH513594 20171208 FL01B 04 GII.2a LPV
100
MH513591 20171031 DE 29 GII.2a LPV
MH513592 20171031 DE 36 GII.2a LPV
MH513593 20171208 FL01B 02 GII.2a LPV
MN502925 20171031 DE 02 GII.2a LPV
GII.2a + GII.2R_LPV
MN502851 20150714 FI 02 GII.2b LPV
94
MN503021 20150122 GL02 04 GII.2b LPV
95
MN502995 20140331 FL04 02 GII.2b LPV
97
MN503035 20150328 FL01A 02 GII.2b LPV
GII.2b
M87661 Norwalk GI.1
0.05

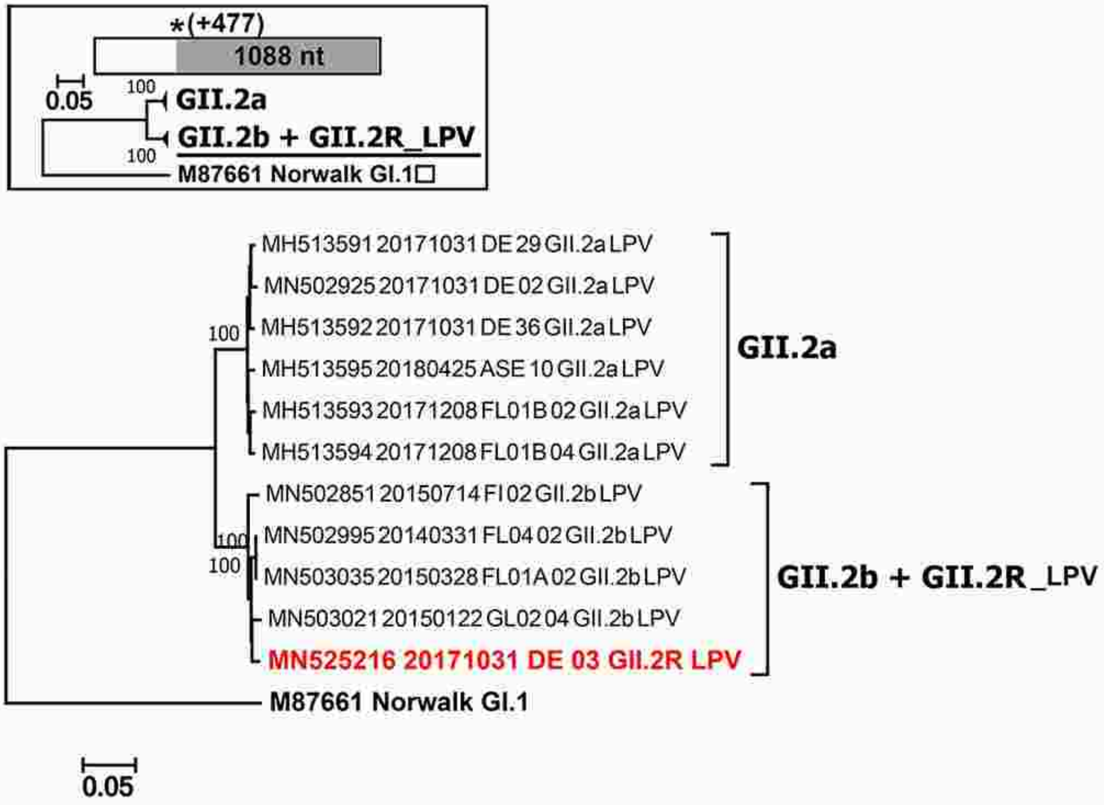
*(+477)
1088 nt
0.05
100
GII.2a
GII.2b + GII.2R_LPV
100
M87661 Norwalk GI.1
MH513591 20171031 DE 29 GII.2a LPV
MN502925 20171031 DE 02 GII.2a LPV
100
MH513592 20171031 DE 36 GII.2a LPV
MH513595 20180425 ASE 10 GII.2a LPV
MH513593 20171208 FL01B 02 GII.2a LPV
MH513594 20171208 FL01B 04 GII.2a LPV
GII.2a
MN502851 20150714 FI 02 GII.2b LPV
100
MN502995 20140331 FL04 02 GII.2b LPV
100
MN503035 20150328 FL01A 02 GII.2b LPV
MN503021 20150122 GL02 04 GII.2b LPV
MN525216 20171031 DE 03 GII.2R LPV
GII.2b + GII.2R_LPV
M87661 Norwalk GI.1
0.05

Fig S8

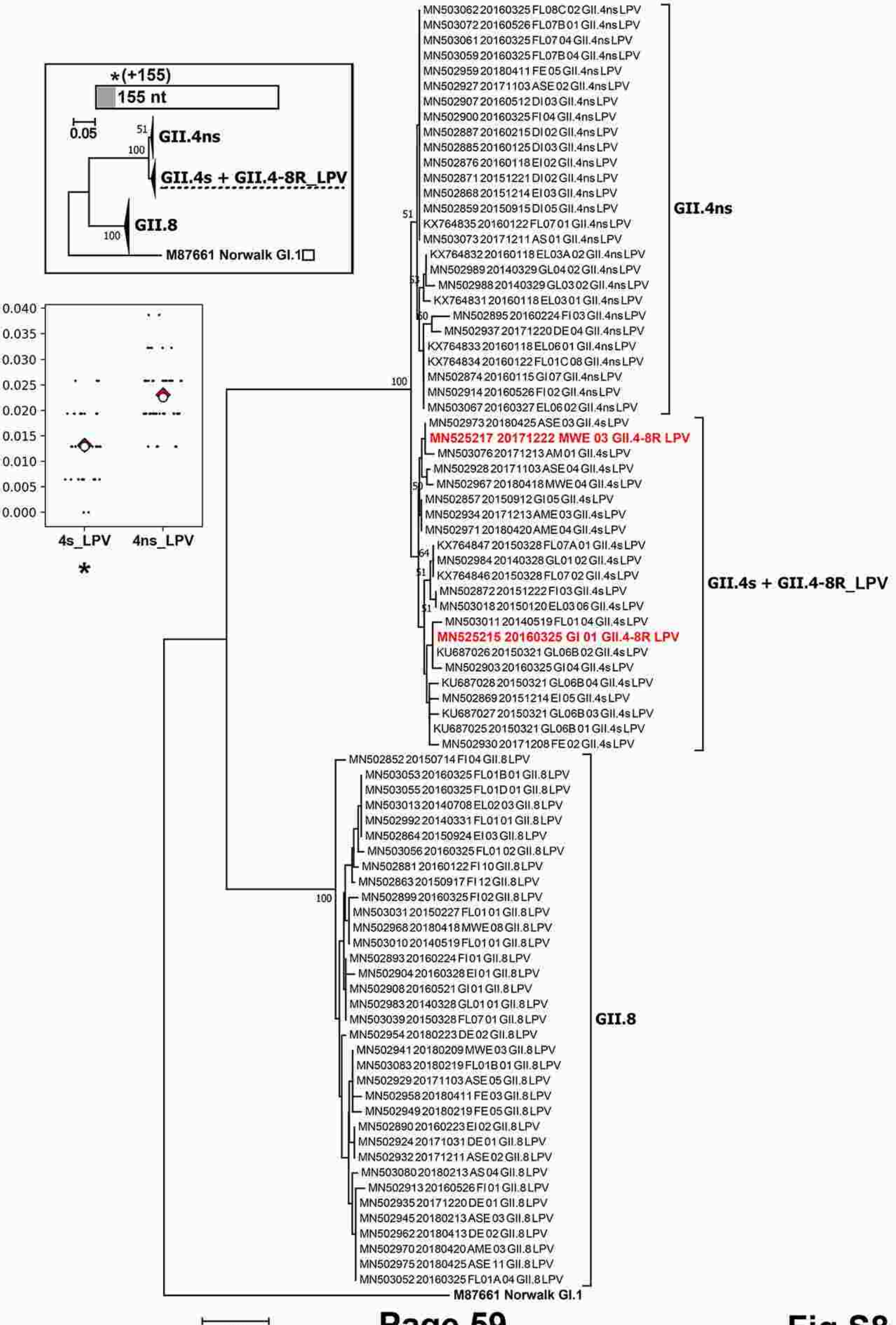
*(+155)
155 nt
0.05
51
100
GII.4ns
GII.4s + GII.4-8R_LPV
GII.8
100
M87661 Norwalk GI.1
0.040
0.035
0.030
0.025
0.020
0.015
0.010
0.005
0.000
p-distances
4s_LPV
4ns_LPV
*
MN503062 20160325 FL08C 02 GII.4ns LPV
MN503072 20160526 FL07B 01 GII.4ns LPV
MN503061 20160325 FL07 04 GII.4ns LPV
MN503059 20160325 FL07B 04 GII.4ns LPV
MN502959 20180411 FE 05 GII.4ns LPV
MN502927 20171103 ASE 02 GII.4ns LPV
MN502907 20160512 DI 03 GII.4ns LPV
MN502900 20160325 FI 04 GII.4ns LPV
MN502887 20160215 DI 02 GII.4ns LPV
MN502885 20160125 DI 03 GII.4ns LPV
MN502876 20160118 EI 02 GII.4ns LPV
MN502871 20151221 DI 02 GII.4ns LPV
MN502868 20151214 EI 03 GII.4ns LPV
MN502859 20150915 DI 05 GII.4ns LPV
KX764835 20160122 FL07 01 GII.4ns LPV
MN503073 20171211 AS 01 GII.4ns LPV
KX764832 20160118 EL03A 02 GII.4ns LPV
MN502989 20140329 GL04 02 GII.4ns LPV
MN502988 20140329 GL03 02 GII.4ns LPV
KX764831 20160118 EL03 01 GII.4ns LPV
MN502895 20160224 FI 03 GII.4ns LPV
MN502937 20171220 DE 04 GII.4ns LPV
KX764833 20160118 EL06 01 GII.4ns LPV
KX764834 20160122 FL01C 08 GII.4ns LPV
MN502874 20160115 GI 07 GII.4ns LPV
MN502914 20160526 FI 02 GII.4ns LPV
MN503067 20160327 EL06 02 GII.4ns LPV
GII.4ns
51
53
50
100
MN502973 20180425 ASE 03 GII.4s LPV
MN525217 20171222 MWE 03 GII.4-8R LPV
MN503076 20171213 AM 01 GII.4s LPV
MN502928 20171103 ASE 04 GII.4s LPV
MN502967 20180418 MWE 04 GII.4s LPV
MN502857 20150912 GI 05 GII.4s LPV
MN502934 20171213 AME 03 GII.4s LPV
MN502971 20180420 AME 04 GII.4s LPV
KX764847 20150328 FL07A 01 GII.4s LPV
MN502984 20140328 GL01 02 GII.4s LPV
KX764846 20150328 FL07 02 GII.4s LPV
MN502872 20151222 FI 03 GII.4s LPV
MN503018 20150120 EL03 06 GII.4s LPV
MN503011 20140519 FL01 04 GII.4s LPV
MN525215 20160325 GI 01 GII.4-8R LPV
KU687026 20150321 GL06B 02 GII.4s LPV
MN502903 20160325 GI 04 GII.4s LPV
KU687028 20150321 GL06B 04 GII.4s LPV
MN502869 20151214 EI 05 GII.4s LPV
KU687027 20150321 GL06B 03 GII.4s LPV
KU687025 20150321 GL06B 01 GII.4s LPV
MN502930 20171208 FE 02 GII.4s LPV
50
64
51
51
GII.4s + GII.4-8R_LPV
MN502852 20150714 FI 04 GII.8 LPV
MN503053 20160325 FL01B 01 GII.8 LPV
MN503055 20160325 FL01D 01 GII.8 LPV
MN503013 20140708 EL02 03 GII.8 LPV
MN502992 20140331 FL01 01 GII.8 LPV
MN502864 20150924 EI 03 GII.8 LPV
MN503056 20160325 FL01 02 GII.8 LPV
MN502881 20160122 FI 10 GII.8 LPV
MN502863 20150917 FI 12 GII.8 LPV
MN502899 20160325 FI 02 GII.8 LPV
MN503031 20150227 FL01 01 GII.8 LPV
MN502968 20180418 MWE 08 GII.8 LPV
MN503010 20140519 FL01 01 GII.8 LPV
MN502893 20160224 FI 01 GII.8 LPV
MN502904 20160328 EI 01 GII.8 LPV
MN502908 20160521 GI 01 GII.8 LPV
MN502983 20140328 GL01 01 GII.8 LPV
MN503039 20150328 FL07 01 GII.8 LPV
MN502954 20180223 DE 02 GII.8 LPV
MN502941 20180209 MWE 03 GII.8 LPV
MN503083 20180219 FL01B 01 GII.8 LPV
MN502929 20171103 ASE 05 GII.8 LPV
MN502958 20180411 FE 03 GII.8 LPV
MN502949 20180219 FE 05 GII.8 LPV
MN502890 20160223 EI 02 GII.8 LPV
MN502924 20171031 DE 01 GII.8 LPV
MN502932 20171211 ASE 02 GII.8 LPV
MN503080 20180213 AS 04 GII.8 LPV
MN502913 20160526 FI 01 GII.8 LPV
MN502935 20171220 DE 01 GII.8 LPV
MN502945 20180213 ASE 03 GII.8 LPV
MN502962 20180413 DE 02 GII.8 LPV
MN502970 20180420 AME 03 GII.8 LPV
MN502975 20180425 ASE 11 GII.8 LPV
MN503052 20160325 FL01A 04 GII.8 LPV
100
GII.8
100
M87661 Norwalk GI.1
0.05

Fig S8

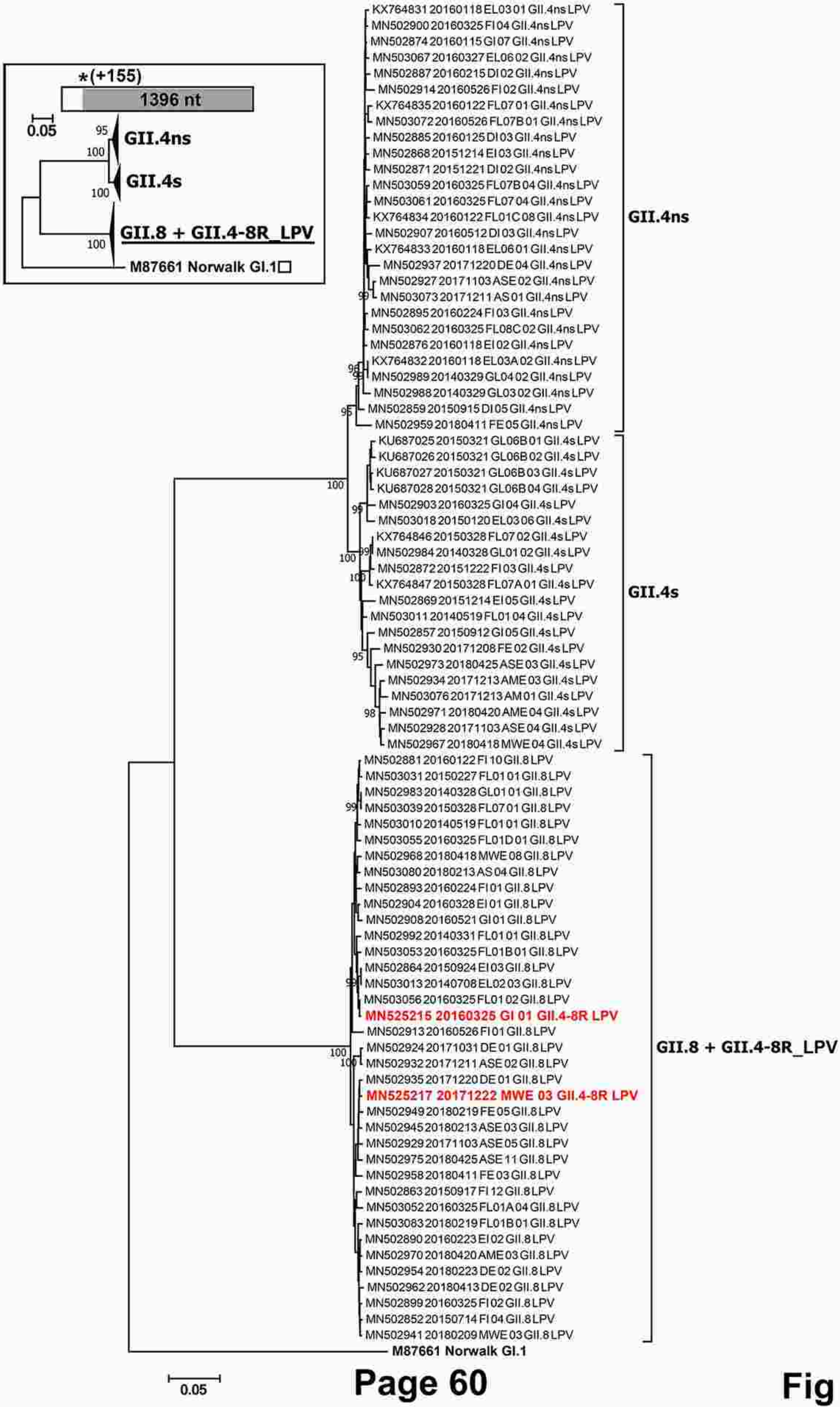
*(+155)
1396 nt
0.05
95
GII.4ns
100
GII.4s
100
GII.8 + GII.4-8R_LPV
100
M87661 Norwalk GI.1□
KX764831 20160118 EL03 01 GII.4ns LPV
MN502900 20160325 FI 04 GII.4ns LPV
MN502874 20160115 GI 07 GII.4ns LPV
MN503067 20160327 EL06 02 GII.4ns LPV
MN502887 20160215 DI 02 GII.4ns LPV
MN502914 20160526 FI 02 GII.4ns LPV
KX764835 20160122 FL07 01 GII.4ns LPV
MN503072 20160526 FL07B 01 GII.4ns LPV
MN502885 20160125 DI 03 GII.4ns LPV
MN502868 20151214 EI 03 GII.4ns LPV
MN502871 20151221 DI 02 GII.4ns LPV
MN503059 20160325 FL07B 04 GII.4ns LPV
MN503061 20160325 FL07 04 GII.4ns LPV
KX764834 20160122 FL01C 08 GII.4ns LPV
MN502907 20160512 DI 03 GII.4ns LPV
KX764833 20160118 EL06 01 GII.4ns LPV
MN502937 20171220 DE 04 GII.4ns LPV
MN502927 20171103 ASE 02 GII.4ns LPV
99
MN503073 20171211 AS 01 GII.4ns LPV
MN502895 20160224 FI 03 GII.4ns LPV
MN503062 20160325 FL08C 02 GII.4ns LPV
MN502876 20160118 EI 02 GII.4ns LPV
KX764832 20160118 EL03A 02 GII.4ns LPV
96
99
MN502989 20140329 GL04 02 GII.4ns LPV
MN502988 20140329 GL03 02 GII.4ns LPV
MN502859 20150915 DI 05 GII.4ns LPV
96
MN502959 20180411 FE 05 GII.4ns LPV
GII.4ns
KU687025 20150321 GL06B 01 GII.4s LPV
KU687026 20150321 GL06B 02 GII.4s LPV
KU687027 20150321 GL06B 03 GII.4s LPV
KU687028 20150321 GL06B 04 GII.4s LPV
100
MN502903 20160325 GI 04 GII.4s LPV
99
MN503018 20150120 EL03 06 GII.4s LPV
KX764846 20150328 FL07 02 GII.4s LPV
99
MN502984 20140328 GL01 02 GII.4s LPV
100
MN502872 20151222 FI 03 GII.4s LPV
100
KX764847 20150328 FL07A 01 GII.4s LPV
MN502869 20151214 EI 05 GII.4s LPV
GII.4s
MN503011 20140519 FL01 04 GII.4s LPV
MN502857 20150912 GI 05 GII.4s LPV
MN502930 20171208 FE 02 GII.4s LPV
95
MN502973 20180425 ASE 03 GII.4s LPV
MN502934 20171213 AME 03 GII.4s LPV
MN503076 20171213 AM 01 GII.4s LPV
98
MN502971 20180420 AME 04 GII.4s LPV
MN502928 20171103 ASE 04 GII.4s LPV
MN502967 20180418 MWE 04 GII.4s LPV
MN502881 20160122 FI 10 GII.8 LPV
MN503031 20150227 FL01 01 GII.8 LPV
MN502983 20140328 GL01 01 GII.8 LPV
99
MN503039 20150328 FL07 01 GII.8 LPV
MN503010 20140519 FL01 01 GII.8 LPV
MN503055 20160325 FL01D 01 GII.8 LPV
MN502968 20180418 MWE 08 GII.8 LPV
MN503080 20180213 AS 04 GII.8 LPV
MN502893 20160224 FI 01 GII.8 LPV
MN502904 20160328 EI 01 GII.8 LPV
MN502908 20160521 GI 01 GII.8 LPV
MN502992 20140331 FL01 01 GII.8 LPV
MN503053 20160325 FL01B 01 GII.8 LPV
MN502864 20150924 EI 03 GII.8 LPV
99
MN503013 20140708 EL02 03 GII.8 LPV
MN503056 20160325 FL01 02 GII.8 LPV
MN525215 20160325 GI 01 GII.4-8R LPV
MN502913 20160526 FI 01 GII.8 LPV
100
MN502924 20171031 DE 01 GII.8 LPV
GII.8 + GII.4-8R_LPV
100
MN502932 20171211 ASE 02 GII.8 LPV
MN502935 20171220 DE 01 GII.8 LPV
MN525217 20171222 MWE 03 GII.4-8R LPV
MN502949 20180219 FE 05 GII.8 LPV
MN502945 20180213 ASE 03 GII.8 LPV
MN502929 20171103 ASE 05 GII.8 LPV
MN502975 20180425 ASE 11 GII.8 LPV
MN502958 20180411 FE 03 GII.8 LPV
MN502863 20150917 FI 12 GII.8 LPV
MN503052 20160325 FL01A 04 GII.8 LPV
MN503083 20180219 FL01B 01 GII.8 LPV
MN502890 20160223 EI 02 GII.8 LPV
MN502970 20180420 AME 03 GII.8 LPV
MN502954 20180223 DE 02 GII.8 LPV
MN502962 20180413 DE 02 GII.8 LPV
MN502899 20160325 FI 02 GII.8 LPV
MN502852 20150714 FI 04 GII.8 LPV
MN502941 20180209 MWE 03 GII.8 LPV
M87661 Norwalk GI.1
0.05

Fig S8

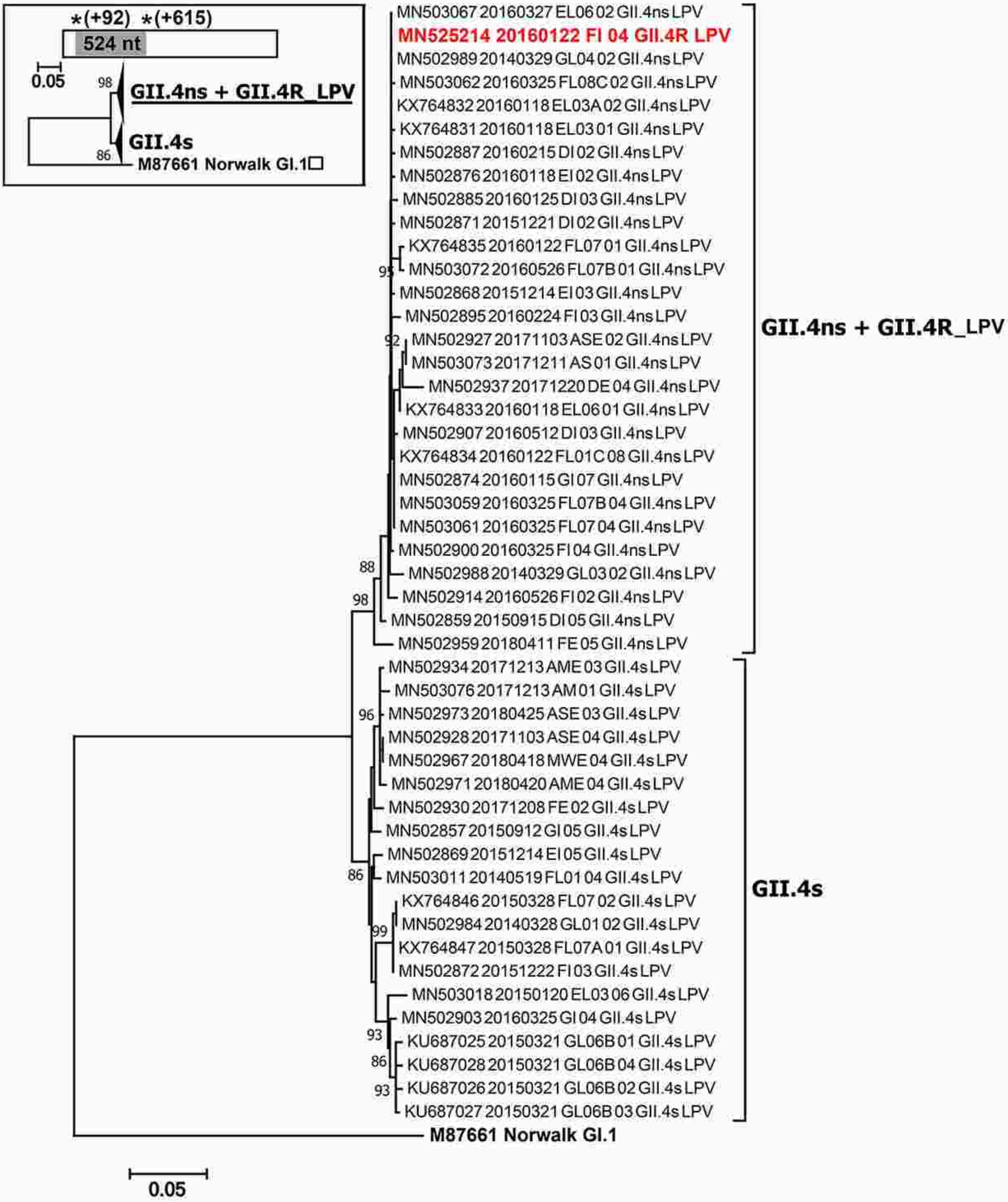

*(+92) *(+615)
524 nt
0.05
98
GII.4ns + GII.4R_LPV
GII.4s
86
M87661 Norwalk GI.1
MN503067 20160327 EL06 02 GII.4ns LPV
MN525214 20160122 FI 04 GII.4R LPV
MN502989 20140329 GL04 02 GII.4ns LPV
MN503062 20160325 FL08C 02 GII.4ns LPV
KX764832 20160118 EL03A 02 GII.4ns LPV
KX764831 20160118 EL03 01 GII.4ns LPV
MN502887 20160215 DI 02 GII.4ns LPV
MN502876 20160118 EI 02 GII.4ns LPV
MN502885 20160125 DI 03 GII.4ns LPV
MN502871 20151221 DI 02 GII.4ns LPV
KX764835 20160122 FL07 01 GII.4ns LPV
95
MN503072 20160526 FL07B 01 GII.4ns LPV
MN502868 20151214 EI 03 GII.4ns LPV
MN502895 20160224 FI 03 GII.4ns LPV
92
MN502927 20171103 ASE 02 GII.4ns LPV
MN503073 20171211 AS 01 GII.4ns LPV
MN502937 20171220 DE 04 GII.4ns LPV
KX764833 20160118 EL06 01 GII.4ns LPV
MN502907 20160512 DI 03 GII.4ns LPV
KX764834 20160122 FL01C 08 GII.4ns LPV
MN502874 20160115 GI 07 GII.4ns LPV
MN503059 20160325 FL07B 04 GII.4ns LPV
MN503061 20160325 FL07 04 GII.4ns LPV
MN502900 20160325 FI 04 GII.4ns LPV
88
MN502988 20140329 GL03 02 GII.4ns LPV
MN502914 20160526 FI 02 GII.4ns LPV
98
MN502859 20150915 DI 05 GII.4ns LPV
MN502959 20180411 FE 05 GII.4ns LPV
GII.4ns + GII.4R_LPV
MN502934 20171213 AME 03 GII.4s LPV
MN503076 20171213 AM 01 GII.4s LPV
96
MN502973 20180425 ASE 03 GII.4s LPV
MN502928 20171103 ASE 04 GII.4s LPV
MN502967 20180418 MWE 04 GII.4s LPV
MN502971 20180420 AME 04 GII.4s LPV
MN502930 20171208 FE 02 GII.4s LPV
MN502857 20150912 GI 05 GII.4s LPV
MN502869 20151214 EI 05 GII.4s LPV
86
MN503011 20140519 FL01 04 GII.4s LPV
KX764846 20150328 FL07 02 GII.4s LPV
MN502984 20140328 GL01 02 GII.4s LPV
99
KX764847 20150328 FL07A 01 GII.4s LPV
MN502872 20151222 FI 03 GII.4s LPV
MN503018 20150120 EL03 06 GII.4s LPV
MN502903 20160325 GI 04 GII.4s LPV
93
KU687025 20150321 GL06B 01 GII.4s LPV
86
KU687028 20150321 GL06B 04 GII.4s LPV
93
KU687026 20150321 GL06B 02 GII.4s LPV
KU687027 20150321 GL06B 03 GII.4s LPV
GII.4s
M87661 Norwalk GI.1
0.05

Fig S8

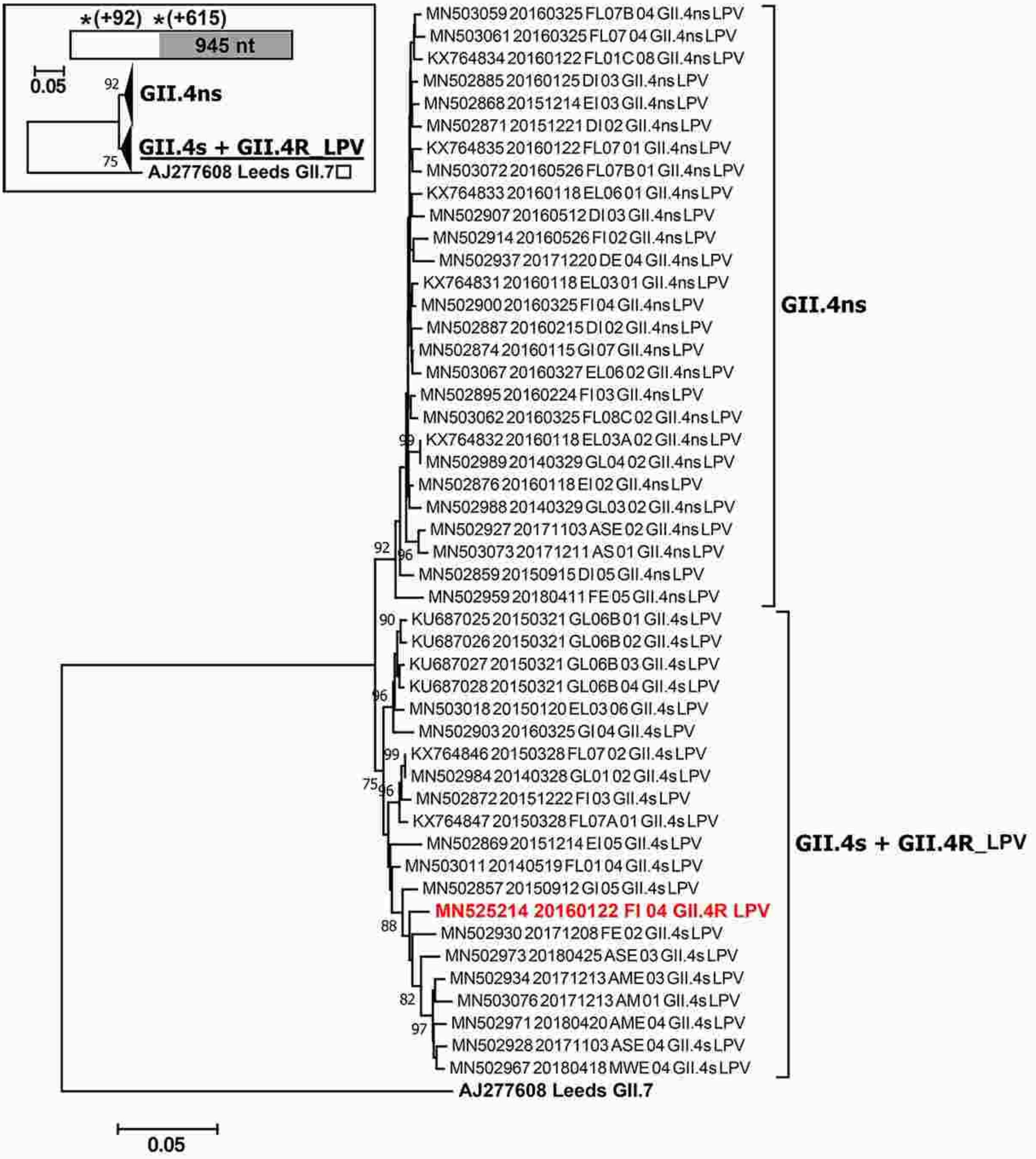

*(+92) *(+615)
945 nt
0.05
92
GII.4ns
GII.4s + GII.4R_LPV
75
AJ277608 Leeds GII.7
MN503059 20160325 FL07B 04 GII.4ns LPV
MN503061 20160325 FL07 04 GII.4ns LPV
KX764834 20160122 FL01C 08 GII.4ns LPV
MN502885 20160125 DI 03 GII.4ns LPV
MN502868 20151214 EI 03 GII.4ns LPV
MN502871 20151221 DI 02 GII.4ns LPV
KX764835 20160122 FL07 01 GII.4ns LPV
MN503072 20160526 FL07B 01 GII.4ns LPV
KX764833 20160118 EL06 01 GII.4ns LPV
MN502907 20160512 DI 03 GII.4ns LPV
MN502914 20160526 FI 02 GII.4ns LPV
MN502937 20171220 DE 04 GII.4ns LPV
KX764831 20160118 EL03 01 GII.4ns LPV
MN502900 20160325 FI 04 GII.4ns LPV
MN502887 20160215 DI 02 GII.4ns LPV
MN502874 20160115 GI 07 GII.4ns LPV
MN503067 20160327 EL06 02 GII.4ns LPV
MN502895 20160224 FI 03 GII.4ns LPV
MN503062 20160325 FL08C 02 GII.4ns LPV
99
KX764832 20160118 EL03A 02 GII.4ns LPV
MN502989 20140329 GL04 02 GII.4ns LPV
MN502876 20160118 EI 02 GII.4ns LPV
MN502988 20140329 GL03 02 GII.4ns LPV
MN502927 20171103 ASE 02 GII.4ns LPV
92
96
MN503073 20171211 AS 01 GII.4ns LPV
MN502859 20150915 DI 05 GII.4ns LPV
MN502959 20180411 FE 05 GII.4ns LPV
GII.4ns
90
KU687025 20150321 GL06B 01 GII.4s LPV
KU687026 20150321 GL06B 02 GII.4s LPV
KU687027 20150321 GL06B 03 GII.4s LPV
KU687028 20150321 GL06B 04 GII.4s LPV
96
MN503018 20150120 EL03 06 GII.4s LPV
MN502903 20160325 GI 04 GII.4s LPV
99
KX764846 20150328 FL07 02 GII.4s LPV
MN502984 20140328 GL01 02 GII.4s LPV
75
96
MN502872 20151222 FI 03 GII.4s LPV
KX764847 20150328 FL07A 01 GII.4s LPV
MN502869 20151214 EI 05 GII.4s LPV
MN503011 20140519 FL01 04 GII.4s LPV
MN502857 20150912 GI 05 GII.4s LPV
MN525214 20160122 FI 04 GII.4R LPV
88
MN502930 20171208 FE 02 GII.4s LPV
MN502973 20180425 ASE 03 GII.4s LPV
MN502934 20171213 AME 03 GII.4s LPV
82
MN503076 20171213 AM 01 GII.4s LPV
MN502971 20180420 AME 04 GII.4s LPV
97
MN502928 20171103 ASE 04 GII.4s LPV
MN502967 20180418 MWE 04 GII.4s LPV
GII.4s + GII.4R_LPV
AJ277608 Leeds GII.7
0.05

Fig S8

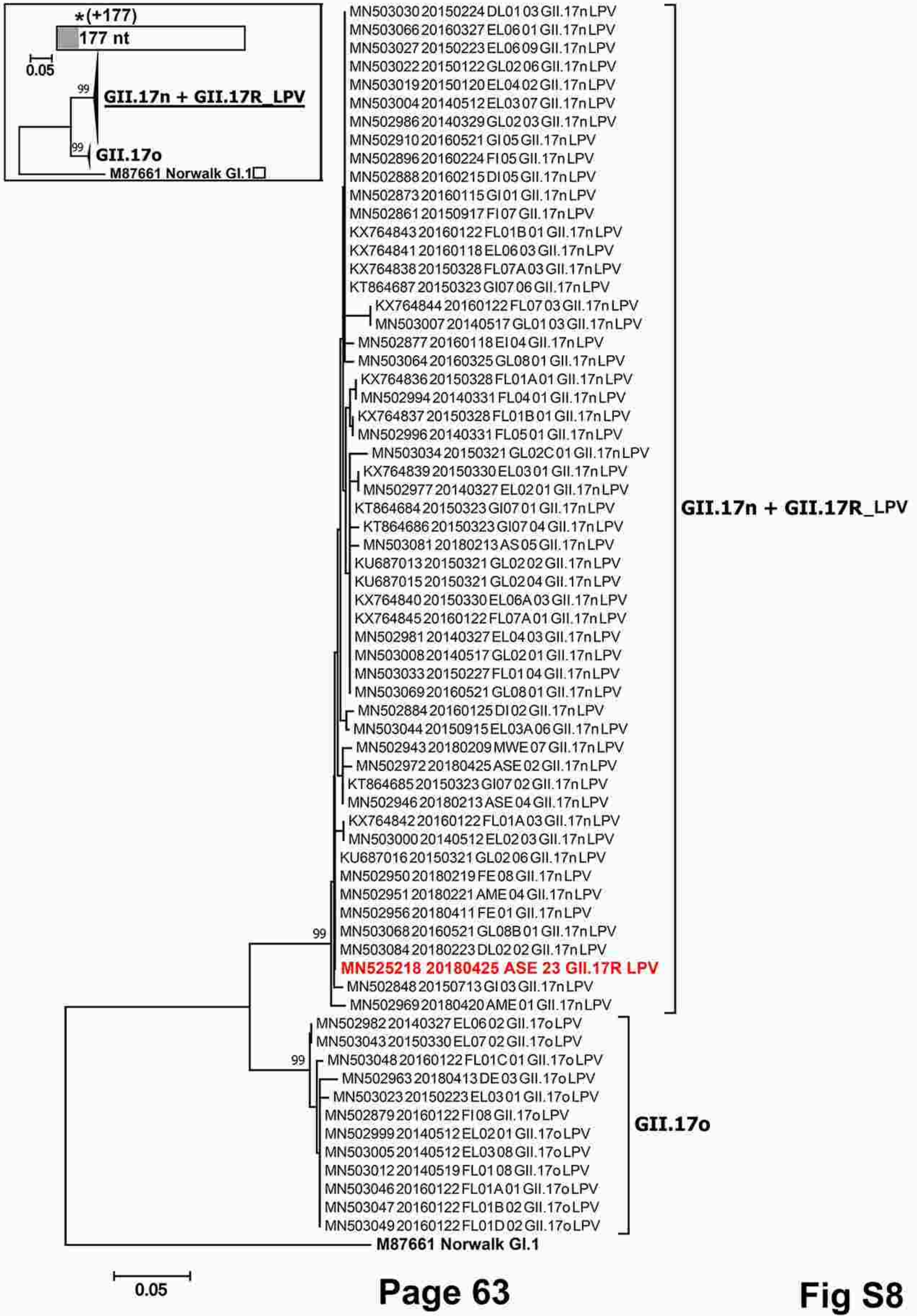

*(+177)
177 nt
0.05
99
GII.17n + GII.17R_LPV
99
GII.17o
M87661 Norwalk GI.1
MN503030 20150224 DL01 03 GII.17n LPV
MN503066 20160327 EL06 01 GII.17n LPV
MN503027 20150223 EL06 09 GII.17n LPV
MN503022 20150122 GL02 06 GII.17n LPV
MN503019 20150120 EL04 02 GII.17n LPV
MN503004 20140512 EL03 07 GII.17n LPV
MN502986 20140329 GL02 03 GII.17n LPV
MN502910 20160521 GI 05 GII.17n LPV
MN502896 20160224 FI 05 GII.17n LPV
MN502888 20160215 DI 05 GII.17n LPV
MN502873 20160115 GI 01 GII.17n LPV
MN502861 20150917 FI 07 GII.17n LPV
KX764843 20160122 FL01B 01 GII.17n LPV
KX764841 20160118 EL06 03 GII.17n LPV
KX764838 20150328 FL07A 03 GII.17n LPV
KT864687 20150323 GI07 06 GII.17n LPV
KX764844 20160122 FL07 03 GII.17n LPV
MN503007 20140517 GL01 03 GII.17n LPV
MN502877 20160118 EI 04 GII.17n LPV
MN503064 20160325 GL08 01 GII.17n LPV
KX764836 20150328 FL01A 01 GII.17n LPV
MN502994 20140331 FL04 01 GII.17n LPV
KX764837 20150328 FL01B 01 GII.17n LPV
MN502996 20140331 FL05 01 GII.17n LPV
MN503034 20150321 GL02C 01 GII.17n LPV
KX764839 20150330 EL03 01 GII.17n LPV
MN502977 20140327 EL02 01 GII.17n LPV
KT864684 20150323 GI07 01 GII.17n LPV
KT864686 20150323 GI07 04 GII.17n LPV
MN503081 20180213 AS 05 GII.17n LPV
KU687013 20150321 GL02 02 GII.17n LPV
KU687015 20150321 GL02 04 GII.17n LPV
KX764840 20150330 EL06A 03 GII.17n LPV
KX764845 20160122 FL07A 01 GII.17n LPV
MN502981 20140327 EL04 03 GII.17n LPV
MN503008 20140517 GL02 01 GII.17n LPV
MN503033 20150227 FL01 04 GII.17n LPV
MN503069 20160521 GL08 01 GII.17n LPV
MN502884 20160125 DI 02 GII.17n LPV
MN503044 20150915 EL03A 06 GII.17n LPV
MN502943 20180209 MWE 07 GII.17n LPV
MN502972 20180425 ASE 02 GII.17n LPV
KT864685 20150323 GI07 02 GII.17n LPV
MN502946 20180213 ASE 04 GII.17n LPV
KX764842 20160122 FL01A 03 GII.17n LPV
MN503000 20140512 EL02 03 GII.17n LPV
KU687016 20150321 GL02 06 GII.17n LPV
MN502950 20180219 FE 08 GII.17n LPV
MN502951 20180221 AME 04 GII.17n LPV
MN502956 20180411 FE 01 GII.17n LPV
99
MN503068 20160521 GL08B 01 GII.17n LPV
MN503084 20180223 DL02 02 GII.17n LPV
MN525218 20180425 ASE 23 GII.17R LPV
MN502848 20150713 GI 03 GII.17n LPV
MN502969 20180420 AME 01 GII.17n LPV
GII.17n + GII.17R_LPV
MN502982 20140327 EL06 02 GII.17o LPV
MN503043 20150330 EL07 02 GII.17o LPV
99
MN503048 20160122 FL01C 01 GII.17o LPV
MN502963 20180413 DE 03 GII.17o LPV
MN503023 20150223 EL03 01 GII.17o LPV
MN502879 20160122 FI 08 GII.17o LPV
MN502999 20140512 EL02 01 GII.17o LPV
MN503005 20140512 EL03 08 GII.17o LPV
MN503012 20140519 FL01 08 GII.17o LPV
MN503046 20160122 FL01A 01 GII.17o LPV
MN503047 20160122 FL01B 02 GII.17o LPV
MN503049 20160122 FL01D 02 GII.17o LPV
GII.17o
M87661 Norwalk GI.1
0.05

Fig S8

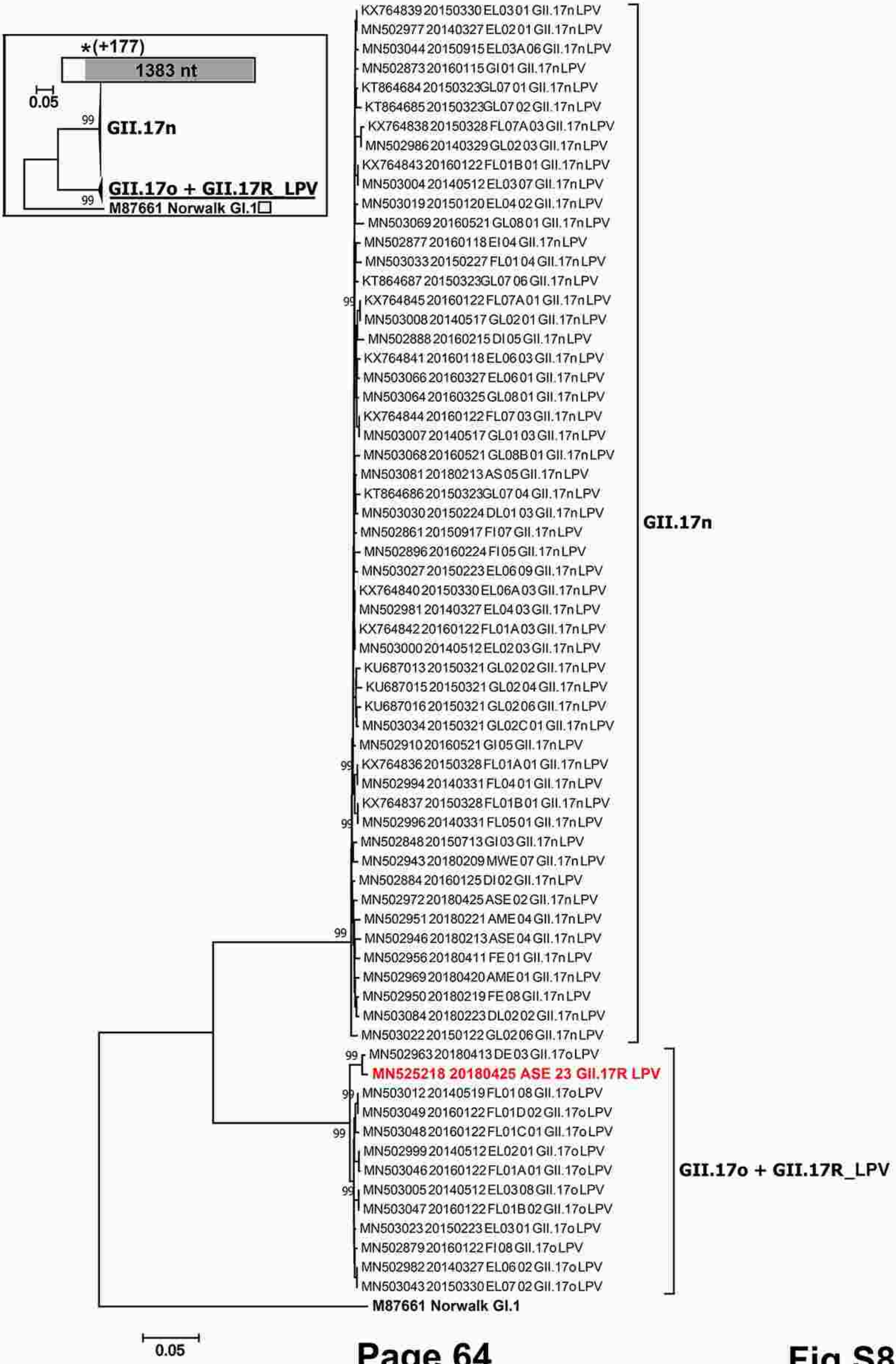

*(+177)
1383 nt
0.05
99
GII.17n
GII.17o + GII.17R_LPV
99
M87661 Norwalk GI.1
KX764839 20150330 EL03 01 GII.17n LPV
MN502977 20140327 EL02 01 GII.17n LPV
MN503044 20150915 EL03A 06 GII.17n LPV
MN502873 20160115 GI 01 GII.17n LPV
KT864684 20150323GL07 01 GII.17n LPV
KT864685 20150323GL07 02 GII.17n LPV
KX764838 20150328 FL07A 03 GII.17n LPV
MN502986 20140329 GL02 03 GII.17n LPV
KX764843 20160122 FL01B 01 GII.17n LPV
MN503004 20140512 EL03 07 GII.17n LPV
MN503019 20150120 EL04 02 GII.17n LPV
MN503069 20160521 GL08 01 GII.17n LPV
MN502877 20160118 EI 04 GII.17n LPV
MN503033 20150227 FL01 04 GII.17n LPV
KT864687 20150323GL07 06 GII.17n LPV
99
KX764845 20160122 FL07A 01 GII.17n LPV
MN503008 20140517 GL02 01 GII.17n LPV
MN502888 20160215 DI 05 GII.17n LPV
KX764841 20160118 EL06 03 GII.17n LPV
MN503066 20160327 EL06 01 GII.17n LPV
MN503064 20160325 GL08 01 GII.17n LPV
KX764844 20160122 FL07 03 GII.17n LPV
MN503007 20140517 GL01 03 GII.17n LPV
MN503068 20160521 GL08B 01 GII.17n LPV
MN503081 20180213 AS 05 GII.17n LPV
KT864686 20150323GL07 04 GII.17n LPV
MN503030 20150224 DL01 03 GII.17n LPV
MN502861 20150917 FI 07 GII.17n LPV
MN502896 20160224 FI 05 GII.17n LPV
MN503027 20150223 EL06 09 GII.17n LPV
KX764840 20150330 EL06A 03 GII.17n LPV
MN502981 20140327 EL04 03 GII.17n LPV
KX764842 20160122 FL01A 03 GII.17n LPV
MN503000 20140512 EL02 03 GII.17n LPV
KU687013 20150321 GL02 02 GII.17n LPV
KU687015 20150321 GL02 04 GII.17n LPV
KU687016 20150321 GL02 06 GII.17n LPV
MN503034 20150321 GL02C 01 GII.17n LPV
MN502910 20160521 GI 05 GII.17n LPV
99
KX764836 20150328 FL01A 01 GII.17n LPV
MN502994 20140331 FL04 01 GII.17n LPV
KX764837 20150328 FL01B 01 GII.17n LPV
99
MN502996 20140331 FL05 01 GII.17n LPV
MN502848 20150713 GI 03 GII.17n LPV
MN502943 20180209 MWE 07 GII.17n LPV
MN502884 20160125 DI 02 GII.17n LPV
MN502972 20180425 ASE 02 GII.17n LPV
MN502951 20180221 AME 04 GII.17n LPV
99
MN502946 20180213 ASE 04 GII.17n LPV
MN502956 20180411 FE 01 GII.17n LPV
MN502969 20180420 AME 01 GII.17n LPV
MN502950 20180219 FE 08 GII.17n LPV
MN503084 20180223 DL02 02 GII.17n LPV
MN503022 20150122 GL02 06 GII.17n LPV
GII.17n
99
MN502963 20180413 DE 03 GII.17o LPV
MN525218 20180425 ASE 23 GII.17R LPV
99
MN503012 20140519 FL01 08 GII.17o LPV
MN503049 20160122 FL01D 02 GII.17o LPV
99
MN503048 20160122 FL01C 01 GII.17o LPV
MN502999 20140512 EL02 01 GII.17o LPV
MN503046 20160122 FL01A 01 GII.17o LPV
99
MN503005 20140512 EL03 08 GII.17o LPV
MN503047 20160122 FL01B 02 GII.17o LPV
MN503023 20150223 EL03 01 GII.17o LPV
MN502879 20160122 FI 08 GII.17o LPV
MN502982 20140327 EL06 02 GII.17o LPV
MN503043 20150330 EL07 02 GII.17o LPV
GII.17o + GII.17R_LPV
M87661 Norwalk GI.1
0.05

Fig S8

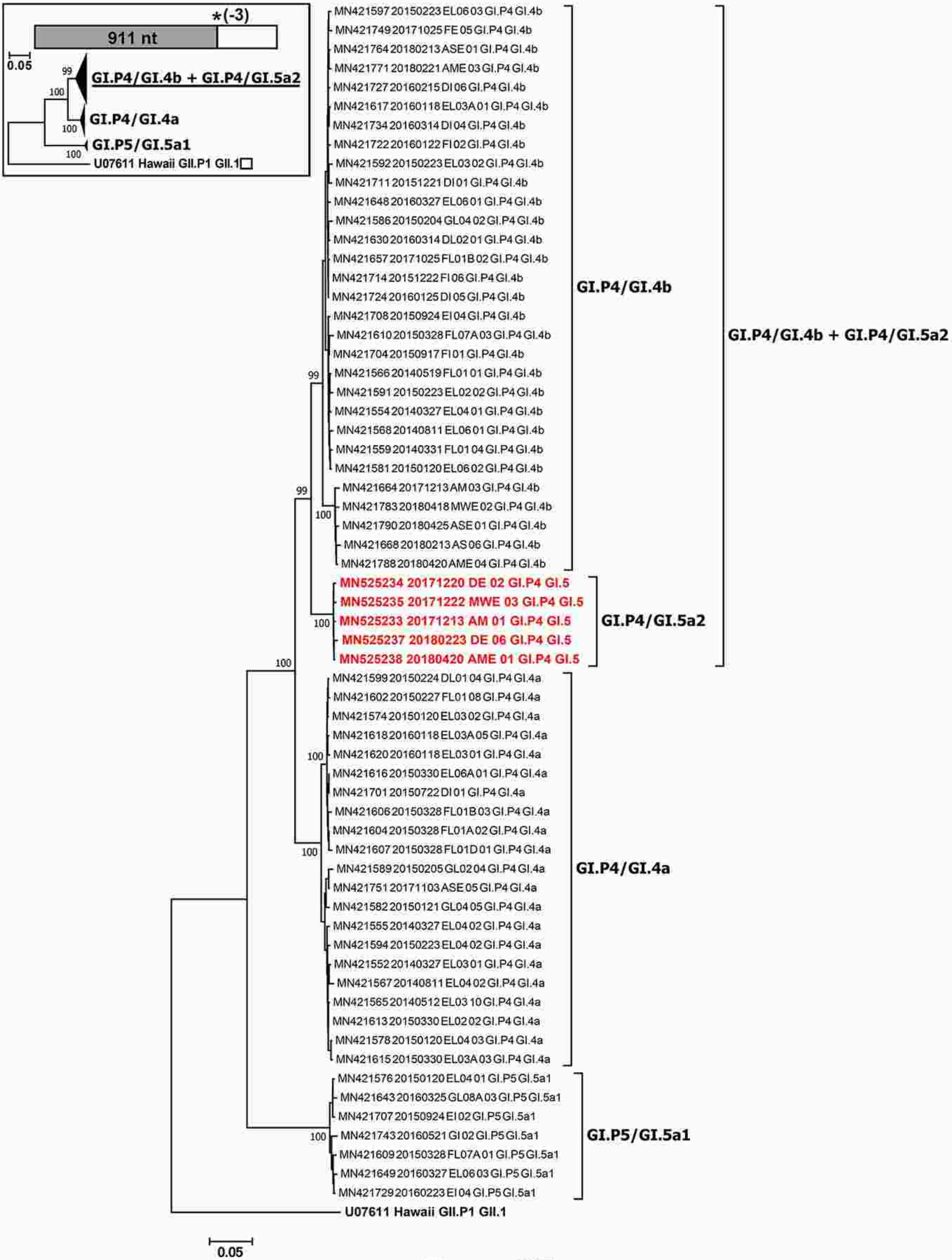

*(-3)
911 nt
0.05
99
100
GI.P4/GI.4b + GI.P4/GI.5a2
GI.P4/GI.4a
100
GI.P5/GI.5a1
100
U07611 Hawaii GII.P1 GII.1
MN421597 20150223 EL06 03 GI.P4 GI.4b
MN421749 20171025 FE 05 GI.P4 GI.4b
MN421764 20180213 ASE 01 GI.P4 GI.4b
MN421771 20180221 AME 03 GI.P4 GI.4b
MN421727 20160215 DI 06 GI.P4 GI.4b
MN421617 20160118 EL03A 01 GI.P4 GI.4b
MN421734 20160314 DI 04 GI.P4 GI.4b
MN421722 20160122 FI 02 GI.P4 GI.4b
MN421592 20150223 EL03 02 GI.P4 GI.4b
MN421711 20151221 DI 01 GI.P4 GI.4b
MN421648 20160327 EL06 01 GI.P4 GI.4b
MN421586 20150204 GL04 02 GI.P4 GI.4b
MN421630 20160314 DL02 01 GI.P4 GI.4b
MN421657 20171025 FL01B 02 GI.P4 GI.4b
MN421714 20151222 FI 06 GI.P4 GI.4b
MN421724 20160125 DI 05 GI.P4 GI.4b
MN421708 20150924 EI 04 GI.P4 GI.4b
MN421610 20150328 FL07A 03 GI.P4 GI.4b
MN421704 20150917 FI 01 GI.P4 GI.4b
MN421566 20140519 FL01 01 GI.P4 GI.4b
MN421591 20150223 EL02 02 GI.P4 GI.4b
MN421554 20140327 EL04 01 GI.P4 GI.4b
MN421568 20140811 EL06 01 GI.P4 GI.4b
MN421559 20140331 FL01 04 GI.P4 GI.4b
MN421581 20150120 EL06 02 GI.P4 GI.4b
MN421664 20171213 AM 03 GI.P4 GI.4b
MN421783 20180418 MWE 02 GI.P4 GI.4b
MN421790 20180425 ASE 01 GI.P4 GI.4b
MN421668 20180213 AS 06 GI.P4 GI.4b
MN421788 20180420 AME 04 GI.P4 GI.4b
GI.P4/GI.4b
99
99
100
MN525234 20171220 DE 02 GI.P4 GI.5
MN525235 20171222 MWE 03 GI.P4 GI.5
MN525233 20171213 AM 01 GI.P4 GI.5
MN525237 20180223 DE 06 GI.P4 GI.5
MN525238 20180420 AME 01 GI.P4 GI.5
100
GI.P4/GI.5a2
GI.P4/GI.4b + GI.P4/GI.5a2
100
MN421599 20150224 DL01 04 GI.P4 GI.4a
MN421602 20150227 FL01 08 GI.P4 GI.4a
MN421574 20150120 EL03 02 GI.P4 GI.4a
MN421618 20160118 EL03A 05 GI.P4 GI.4a
MN421620 20160118 EL03 01 GI.P4 GI.4a
MN421616 20150330 EL06A 01 GI.P4 GI.4a
MN421701 20150722 DI 01 GI.P4 GI.4a
MN421606 20150328 FL01B 03 GI.P4 GI.4a
MN421604 20150328 FL01A 02 GI.P4 GI.4a
MN421607 20150328 FL01D 01 GI.P4 GI.4a
MN421589 20150205 GL02 04 GI.P4 GI.4a
MN421751 20171103 ASE 05 GI.P4 GI.4a
MN421582 20150121 GL04 05 GI.P4 GI.4a
MN421555 20140327 EL04 02 GI.P4 GI.4a
MN421594 20150223 EL04 02 GI.P4 GI.4a
MN421552 20140327 EL03 01 GI.P4 GI.4a
MN421567 20140811 EL04 02 GI.P4 GI.4a
MN421565 20140512 EL03 10 GI.P4 GI.4a
MN421613 20150330 EL02 02 GI.P4 GI.4a
MN421578 20150120 EL04 03 GI.P4 GI.4a
MN421615 20150330 EL03A 03 GI.P4 GI.4a
100
100
GI.P4/GI.4a
MN421576 20150120 EL04 01 GI.P5 GI.5a1
MN421643 20160325 GL08A 03 GI.P5 GI.5a1
MN421707 20150924 EI 02 GI.P5 GI.5a1
MN421743 20160521 GI 02 GI.P5 GI.5a1
MN421609 20150328 FL07A 01 GI.P5 GI.5a1
MN421649 20160327 EL06 03 GI.P5 GI.5a1
MN421729 20160223 EI 04 GI.P5 GI.5a1
100
GI.P5/GI.5a1
U07611 Hawaii GII.P1 GII.1
0.05

Fig S8

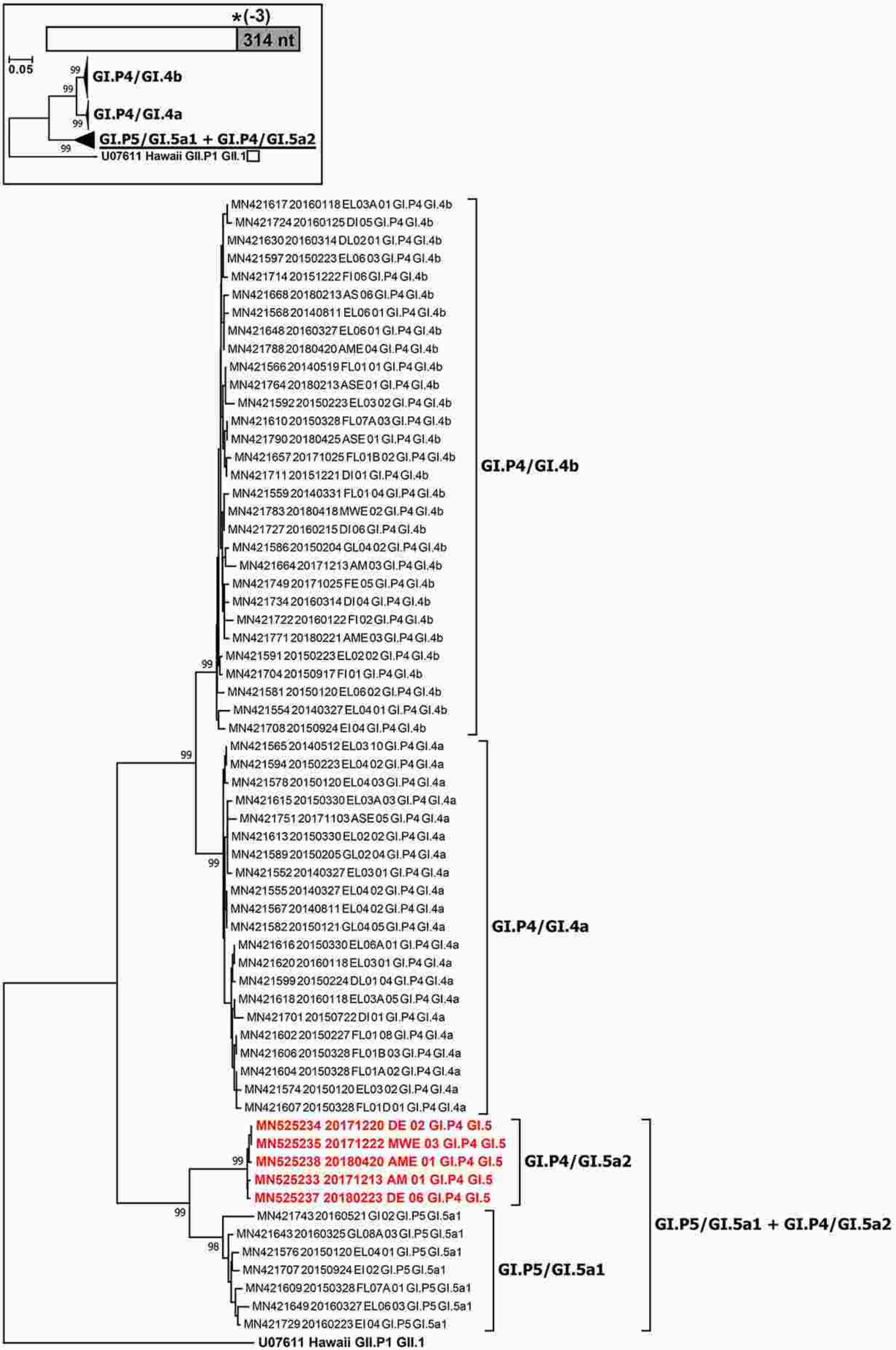

*(-3)
314 nt
0.05
99
99
99
99
GI.P4/GI.4b
GI.P4/GI.4a
GI.P5/GI.5a1 + GI.P4/GI.5a2
U07611 Hawaii GII.P1 GII.1
MN421617 20160118 EL03A 01 GI.P4 GI.4b
MN421724 20160125 DI 05 GI.P4 GI.4b
MN421630 20160314 DL02 01 GI.P4 GI.4b
MN421597 20150223 EL06 03 GI.P4 GI.4b
MN421714 20151222 FI 06 GI.P4 GI.4b
MN421668 20180213 AS 06 GI.P4 GI.4b
MN421568 20140811 EL06 01 GI.P4 GI.4b
MN421648 20160327 EL06 01 GI.P4 GI.4b
MN421788 20180420 AME 04 GI.P4 GI.4b
MN421566 20140519 FL01 01 GI.P4 GI.4b
MN421764 20180213 ASE 01 GI.P4 GI.4b
MN421592 20150223 EL03 02 GI.P4 GI.4b
MN421610 20150328 FL07A 03 GI.P4 GI.4b
MN421790 20180425 ASE 01 GI.P4 GI.4b
MN421657 20171025 FL01B 02 GI.P4 GI.4b
MN421711 20151221 DI 01 GI.P4 GI.4b
MN421559 20140331 FL01 04 GI.P4 GI.4b
MN421783 20180418 MWE 02 GI.P4 GI.4b
MN421727 20160215 DI 06 GI.P4 GI.4b
MN421586 20150204 GL04 02 GI.P4 GI.4b
MN421664 20171213 AM 03 GI.P4 GI.4b
MN421749 20171025 FE 05 GI.P4 GI.4b
MN421734 20160314 DI 04 GI.P4 GI.4b
MN421722 20160122 FI 02 GI.P4 GI.4b
MN421771 20180221 AME 03 GI.P4 GI.4b
MN421591 20150223 EL02 02 GI.P4 GI.4b
MN421704 20150917 FI 01 GI.P4 GI.4b
MN421581 20150120 EL06 02 GI.P4 GI.4b
MN421554 20140327 EL04 01 GI.P4 GI.4b
MN421708 20150924 EI 04 GI.P4 GI.4b
GI.P4/GI.4b
99
99
MN421565 20140512 EL03 10 GI.P4 GI.4a
MN421594 20150223 EL04 02 GI.P4 GI.4a
MN421578 20150120 EL04 03 GI.P4 GI.4a
MN421615 20150330 EL03A 03 GI.P4 GI.4a
MN421751 20171103 ASE 05 GI.P4 GI.4a
MN421613 20150330 EL02 02 GI.P4 GI.4a
MN421589 20150205 GL02 04 GI.P4 GI.4a
99
MN421552 20140327 EL03 01 GI.P4 GI.4a
MN421555 20140327 EL04 02 GI.P4 GI.4a
MN421567 20140811 EL04 02 GI.P4 GI.4a
MN421582 20150121 GL04 05 GI.P4 GI.4a
MN421616 20150330 EL06A 01 GI.P4 GI.4a
MN421620 20160118 EL03 01 GI.P4 GI.4a
MN421599 20150224 DL01 04 GI.P4 GI.4a
MN421618 20160118 EL03A 05 GI.P4 GI.4a
MN421701 20150722 DI 01 GI.P4 GI.4a
MN421602 20150227 FL01 08 GI.P4 GI.4a
MN421606 20150328 FL01B 03 GI.P4 GI.4a
MN421604 20150328 FL01A 02 GI.P4 GI.4a
MN421574 20150120 EL03 02 GI.P4 GI.4a
MN421607 20150328 FL01D 01 GI.P4 GI.4a
GI.P4/GI.4a
MN525234 20171220 DE 02 GI.P4 GI.5
MN525235 20171222 MWE 03 GI.P4 GI.5
99
MN525238 20180420 AME 01 GI.P4 GI.5
MN525233 20171213 AM 01 GI.P4 GI.5
MN525237 20180223 DE 06 GI.P4 GI.5
GI.P4/GI.5a2
99
MN421743 20160521 GI 02 GI.P5 GI.5a1
MN421643 20160325 GL08A 03 GI.P5 GI.5a1
98
MN421576 20150120 EL04 01 GI.P5 GI.5a1
MN421707 20150924 EI 02 GI.P5 GI.5a1
MN421609 20150328 FL07A 01 GI.P5 GI.5a1
MN421649 20160327 EL06 03 GI.P5 GI.5a1
MN421729 20160223 EI 04 GI.P5 GI.5a1
GI.P5/GI.5a1
GI.P5/GI.5a1 + GI.P4/GI.5a2
U07611 Hawaii GII.P1 GII.1

0.05

Fig S8

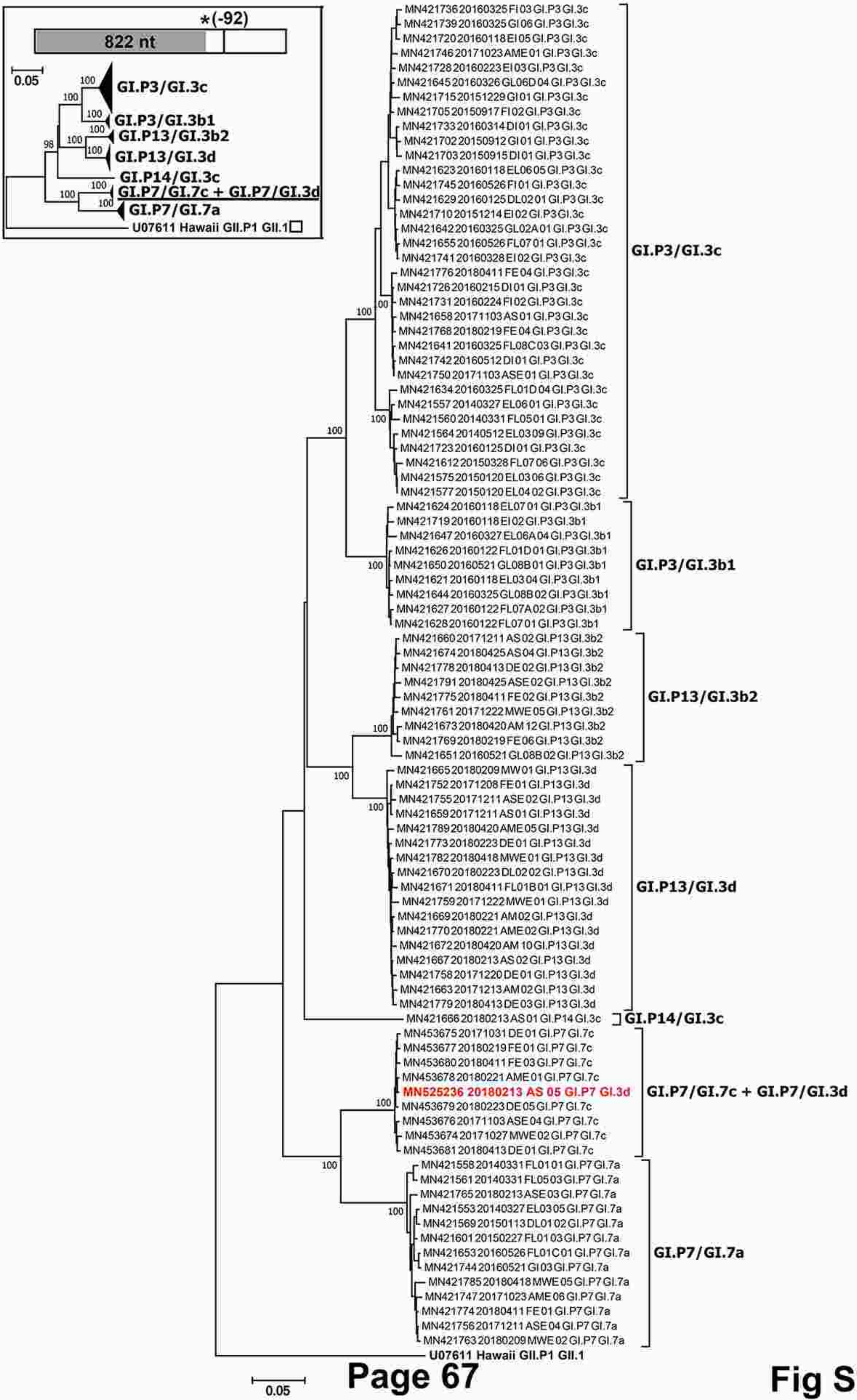
*(-92)
822 nt
0.05
100
GI.P3/GI.3c
100
100
GI.P3/GI.3b1
100
GI.P13/GI.3b2
98
100
100
GI.P13/GI.3d
GI.P14/GI.3c
100
GI.P7/GI.7c + GI.P7/GI.3d
100
100
GI.P7/GI.7a
U07611 Hawaii GII.P1 GII.1
MN421736 20160325 FI 03 GI.P3 GI.3c
MN421739 20160325 GI 06 GI.P3 GI.3c
MN421720 20160118 EI 05 GI.P3 GI.3c
MN421746 20171023 AME 01 GI.P3 GI.3c
MN421728 20160223 EI 03 GI.P3 GI.3c
MN421645 20160326 GL06D 04 GI.P3 GI.3c
MN421715 20151229 GI 01 GI.P3 GI.3c
MN421705 20150917 FI 02 GI.P3 GI.3c
MN421733 20160314 DI 01 GI.P3 GI.3c
MN421702 20150912 GI 01 GI.P3 GI.3c
MN421703 20150915 DI 01 GI.P3 GI.3c
MN421623 20160118 EL06 05 GI.P3 GI.3c
MN421745 20160526 FI 01 GI.P3 GI.3c
MN421629 20160125 DL02 01 GI.P3 GI.3c
MN421710 20151214 EI 02 GI.P3 GI.3c
MN421642 20160325 GL02A 01 GI.P3 GI.3c
MN421655 20160526 FL07 01 GI.P3 GI.3c
MN421741 20160328 EI 02 GI.P3 GI.3c
MN421776 20180411 FE 04 GI.P3 GI.3c
MN421726 20160215 DI 01 GI.P3 GI.3c
MN421731 20160224 FI 02 GI.P3 GI.3c
MN421658 20171103 AS 01 GI.P3 GI.3c
MN421768 20180219 FE 04 GI.P3 GI.3c
MN421641 20160325 FL08C 03 GI.P3 GI.3c
MN421742 20160512 DI 01 GI.P3 GI.3c
MN421750 20171103 ASE 01 GI.P3 GI.3c
MN421634 20160325 FL01D 04 GI.P3 GI.3c
MN421557 20140327 EL06 01 GI.P3 GI.3c
MN421560 20140331 FL05 01 GI.P3 GI.3c
MN421564 20140512 EL03 09 GI.P3 GI.3c
MN421723 20160125 DI 01 GI.P3 GI.3c
MN421612 20150328 FL07 06 GI.P3 GI.3c
MN421575 20150120 EL03 06 GI.P3 GI.3c
MN421577 20150120 EL04 02 GI.P3 GI.3c
00
100
100
100
GI.P3/GI.3c
MN421624 20160118 EL07 01 GI.P3 GI.3b1
MN421719 20160118 EI 02 GI.P3 GI.3b1
MN421647 20160327 EL06A 04 GI.P3 GI.3b1
MN421626 20160122 FL01D 01 GI.P3 GI.3b1
MN421650 20160521 GL08B 01 GI.P3 GI.3b1
MN421621 20160118 EL03 04 GI.P3 GI.3b1
MN421644 20160325 GL08B 02 GI.P3 GI.3b1
MN421627 20160122 FL07A 02 GI.P3 GI.3b1
MN421628 20160122 FL07 01 GI.P3 GI.3b1
100
GI.P3/GI.3b1
MN421660 20171211 AS 02 GI.P13 GI.3b2
MN421674 20180425 AS 04 GI.P13 GI.3b2
MN421778 20180413 DE 02 GI.P13 GI.3b2
MN421791 20180425 ASE 02 GI.P13 GI.3b2
MN421775 20180411 FE 02 GI.P13 GI.3b2
MN421761 20171222 MWE 05 GI.P13 GI.3b2
MN421673 20180420 AM 12 GI.P13 GI.3b2
MN421769 20180219 FE 06 GI.P13 GI.3b2
MN421651 20160521 GL08B 02 GI.P13 GI.3b2
100
GI.P13/GI.3b2
MN421665 20180209 MW 01 GI.P13 GI.3d
MN421752 20171208 FE 01 GI.P13 GI.3d
MN421755 20171211 ASE 02 GI.P13 GI.3d
MN421659 20171211 AS 01 GI.P13 GI.3d
MN421789 20180420 AME 05 GI.P13 GI.3d
MN421773 20180223 DE 01 GI.P13 GI.3d
MN421782 20180418 MWE 01 GI.P13 GI.3d
MN421670 20180223 DL02 02 GI.P13 GI.3d
MN421671 20180411 FL01B 01 GI.P13 GI.3d
MN421759 20171222 MWE 01 GI.P13 GI.3d
MN421669 20180221 AM 02 GI.P13 GI.3d
MN421770 20180221 AME 02 GI.P13 GI.3d
MN421672 20180420 AM 10 GI.P13 GI.3d
MN421667 20180213 AS 02 GI.P13 GI.3d
MN421758 20171220 DE 01 GI.P13 GI.3d
MN421663 20171213 AM 02 GI.P13 GI.3d
MN421779 20180413 DE 03 GI.P13 GI.3d
100
100
GI.P13/GI.3d
MN421666 20180213 AS 01 GI.P14 GI.3c
GI.P14/GI.3c
MN453675 20171031 DE 01 GI.P7 GI.7c
MN453677 20180219 FE 01 GI.P7 GI.7c
MN453680 20180411 FE 03 GI.P7 GI.7c
MN453678 20180221 AME 01 GI.P7 GI.7c
MN525236 20180213 AS 05 GI.P7 GI.3d
MN453679 20180223 DE 05 GI.P7 GI.7c
MN453676 20171103 ASE 04 GI.P7 GI.7c
MN453674 20171027 MWE 02 GI.P7 GI.7c
MN453681 20180413 DE 01 GI.P7 GI.7c
100
GI.P7/GI.7c + GI.P7/GI.3d
100
MN421558 20140331 FL01 01 GI.P7 GI.7a
MN421561 20140331 FL05 03 GI.P7 GI.7a
MN421765 20180213 ASE 03 GI.P7 GI.7a
MN421553 20140327 EL03 05 GI.P7 GI.7a
MN421569 20150113 DL01 02 GI.P7 GI.7a
MN421601 20150227 FL01 03 GI.P7 GI.7a
MN421653 20160526 FL01C 01 GI.P7 GI.7a
MN421744 20160521 GI 03 GI.P7 GI.7a
MN421785 20180418 MWE 05 GI.P7 GI.7a
MN421747 20171023 AME 06 GI.P7 GI.7a
MN421774 20180411 FE 01 GI.P7 GI.7a
MN421756 20171211 ASE 04 GI.P7 GI.7a
MN421763 20180209 MWE 02 GI.P7 GI.7a
100
GI.P7/GI.7a
U07611 Hawaii GII.P1 GII.1
0.05

Fig S8

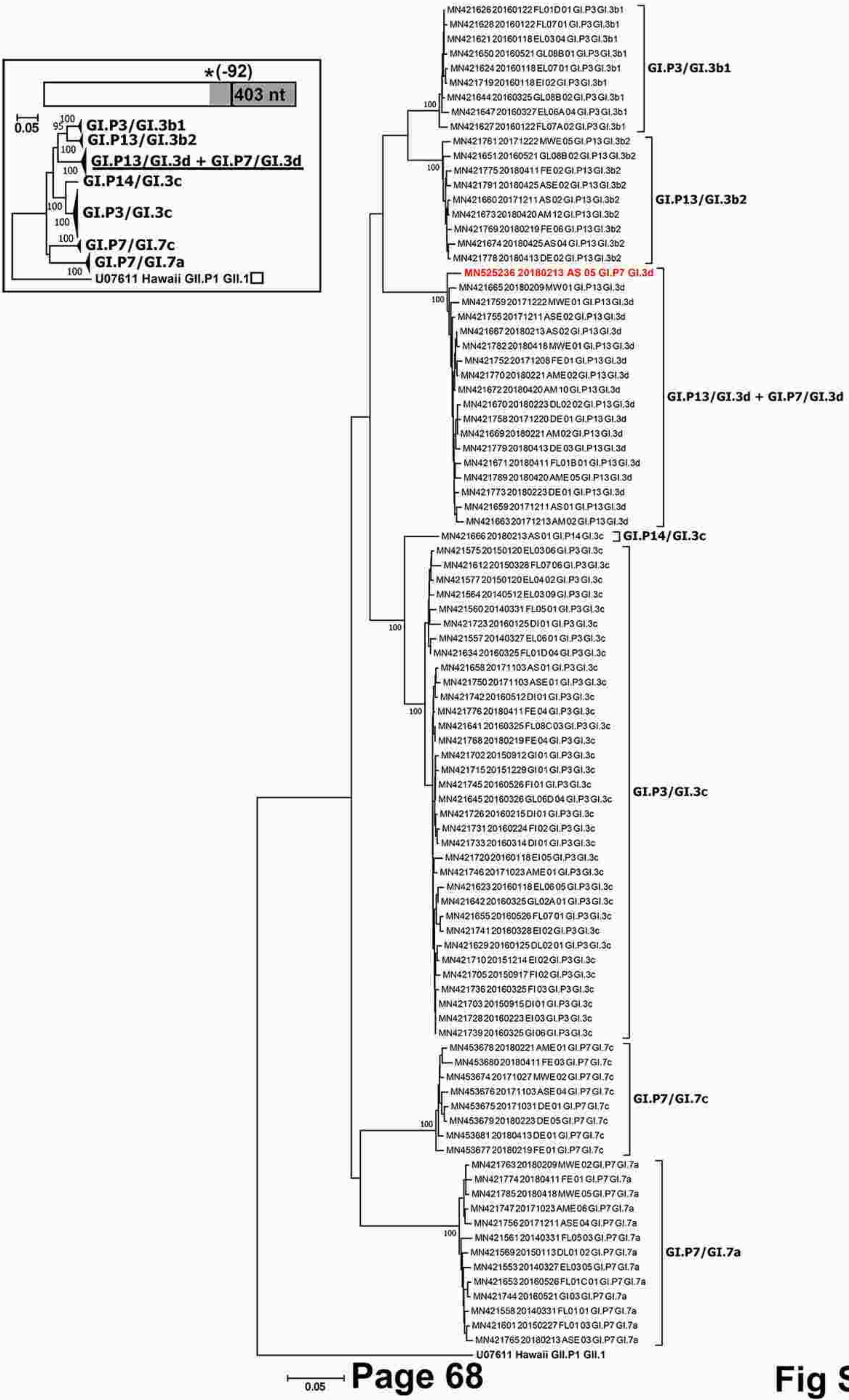

Page 68

Fig S8

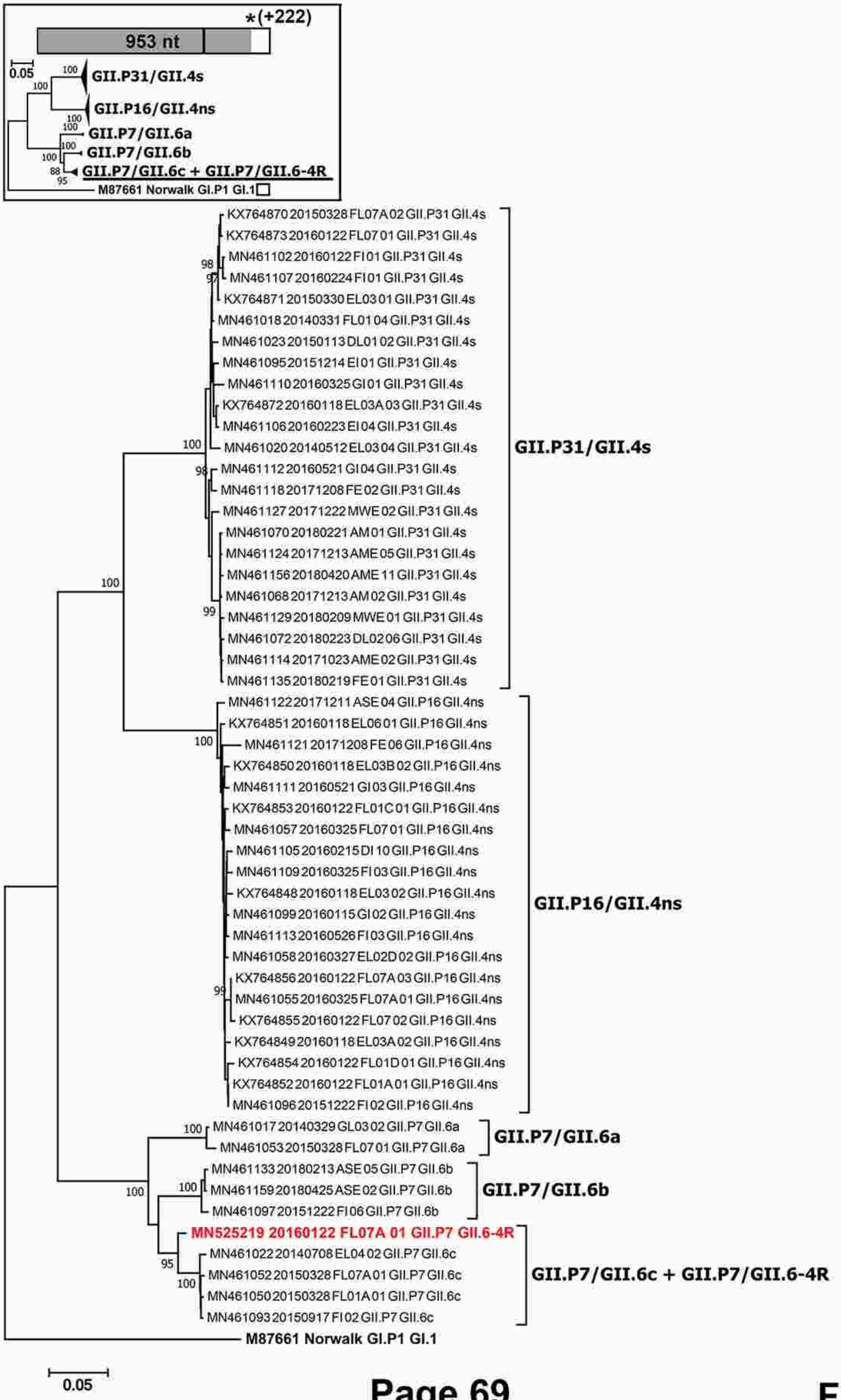
*(+222)
953 nt
0.05
GII.P31/GII.4s
GII.P16/GII.4ns
GII.P7/GII.6a
GII.P7/GII.6b
GII.P7/GII.6c + GII.P7/GII.6-4R
M87661 Norwalk GI.P1 GI.1
KX764870 20150328 FL07A 02 GII.P31 GII.4s
KX764873 20160122 FL07 01 GII.P31 GII.4s
MN461102 20160122 FI 01 GII.P31 GII.4s
MN461107 20160224 FI 01 GII.P31 GII.4s
KX764871 20150330 EL03 01 GII.P31 GII.4s
MN461018 20140331 FL01 04 GII.P31 GII.4s
MN461023 20150113 DL01 02 GII.P31 GII.4s
MN461095 20151214 EI 01 GII.P31 GII.4s
MN461110 20160325 GI 01 GII.P31 GII.4s
KX764872 20160118 EL03A 03 GII.P31 GII.4s
MN461106 20160223 EI 04 GII.P31 GII.4s
MN461020 20140512 EL03 04 GII.P31 GII.4s
MN461112 20160521 GI 04 GII.P31 GII.4s
MN461118 20171208 FE 02 GII.P31 GII.4s
MN461127 20171222 MWE 02 GII.P31 GII.4s
MN461070 20180221 AM 01 GII.P31 GII.4s
MN461124 20171213 AME 05 GII.P31 GII.4s
MN461156 20180420 AME 11 GII.P31 GII.4s
MN461068 20171213 AM 02 GII.P31 GII.4s
MN461129 20180209 MWE 01 GII.P31 GII.4s
MN461072 20180223 DL02 06 GII.P31 GII.4s
MN461114 20171023 AME 02 GII.P31 GII.4s
MN461135 20180219 FE 01 GII.P31 GII.4s
GII.P31/GII.4s
MN461122 20171211 ASE 04 GII.P16 GII.4ns
KX764851 20160118 EL06 01 GII.P16 GII.4ns
MN461121 20171208 FE 06 GII.P16 GII.4ns
KX764850 20160118 EL03B 02 GII.P16 GII.4ns
MN461111 20160521 GI 03 GII.P16 GII.4ns
KX764853 20160122 FL01C 01 GII.P16 GII.4ns
MN461057 20160325 FL07 01 GII.P16 GII.4ns
MN461105 20160215 DI 10 GII.P16 GII.4ns
MN461109 20160325 FI 03 GII.P16 GII.4ns
KX764848 20160118 EL03 02 GII.P16 GII.4ns
MN461099 20160115 GI 02 GII.P16 GII.4ns
MN461113 20160526 FI 03 GII.P16 GII.4ns
MN461058 20160327 EL02D 02 GII.P16 GII.4ns
KX764856 20160122 FL07A 03 GII.P16 GII.4ns
MN461055 20160325 FL07A 01 GII.P16 GII.4ns
KX764855 20160122 FL07 02 GII.P16 GII.4ns
KX764849 20160118 EL03A 02 GII.P16 GII.4ns
KX764854 20160122 FL01D 01 GII.P16 GII.4ns
KX764852 20160122 FL01A 01 GII.P16 GII.4ns
MN461096 20151222 FI 02 GII.P16 GII.4ns
GII.P16/GII.4ns
MN461017 20140329 GL03 02 GII.P7 GII.6a
MN461053 20150328 FL07 01 GII.P7 GII.6a
GII.P7/GII.6a
MN461133 20180213 ASE 05 GII.P7 GII.6b
MN461159 20180425 ASE 02 GII.P7 GII.6b
MN461097 20151222 FI 06 GII.P7 GII.6b
GII.P7/GII.6b
MN525219 20160122 FL07A 01 GII.P7 GII.6-4R
MN461022 20140708 EL04 02 GII.P7 GII.6c
MN461052 20150328 FL07A 01 GII.P7 GII.6c
MN461050 20150328 FL01A 01 GII.P7 GII.6c
MN461093 20150917 FI 02 GII.P7 GII.6c
GII.P7/GII.6c + GII.P7/GII.6-4R
M87661 Norwalk GI.P1 GI.1
0.05

Fig S8

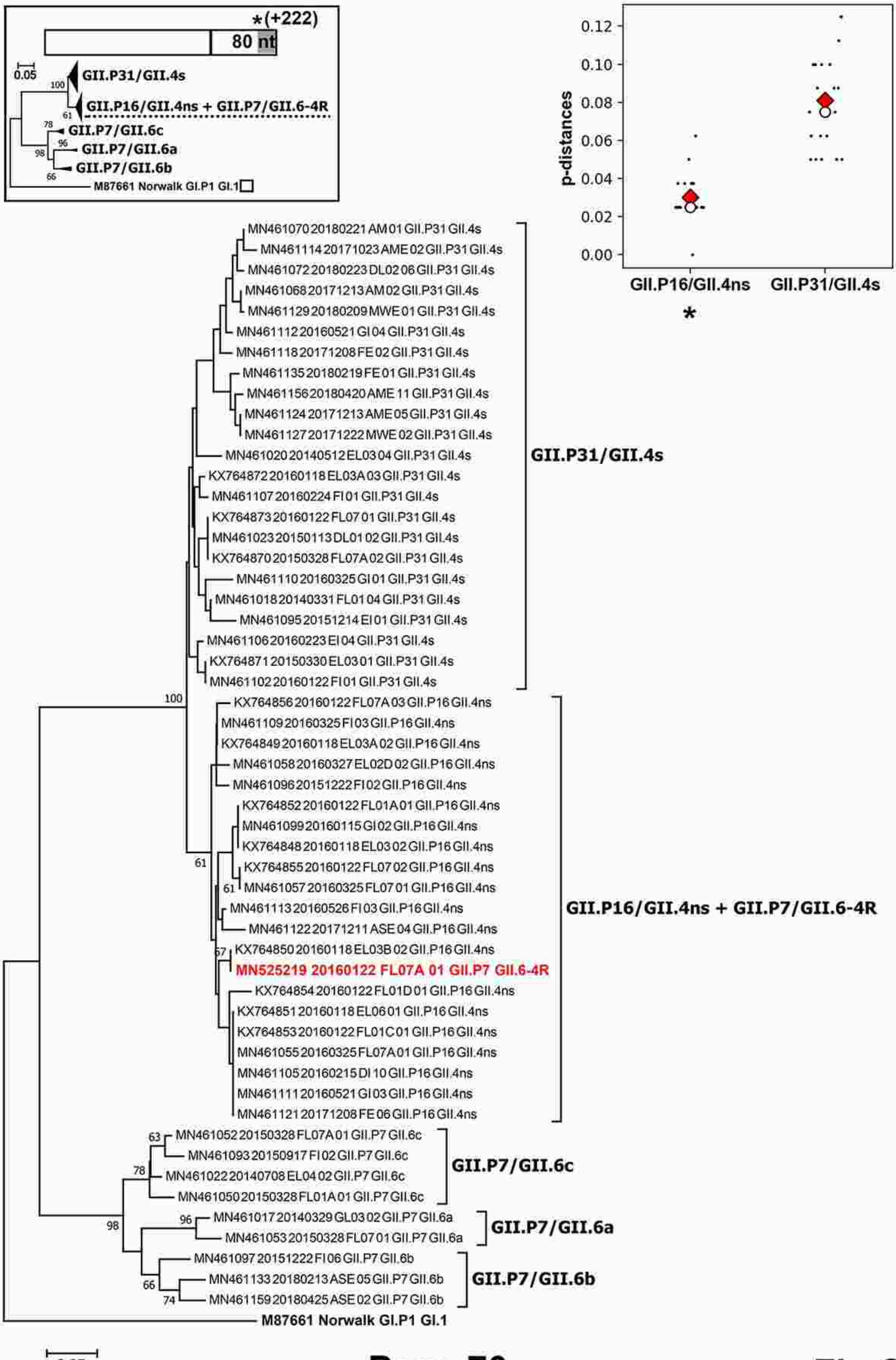

*(+222)
80 nt
0.05
GII.P31/GII.4s
GII.P16/GII.4ns + GII.P7/GII.6-4R
GII.P7/GII.6c
GII.P7/GII.6a
GII.P7/GII.6b
M87661 Norwalk GI.P1 GI.1
p-distances
0.12
0.10
0.08
0.06
0.04
0.02
0.00
GII.P16/GII.4ns
GII.P31/GII.4s
*
MN461070 20180221 AM 01 GII.P31 GII.4s
MN461114 20171023 AME 02 GII.P31 GII.4s
MN461072 20180223 DL02 06 GII.P31 GII.4s
MN461068 20171213 AM 02 GII.P31 GII.4s
MN461129 20180209 MWE 01 GII.P31 GII.4s
MN461112 20160521 GI 04 GII.P31 GII.4s
MN461118 20171208 FE 02 GII.P31 GII.4s
MN461135 20180219 FE 01 GII.P31 GII.4s
MN461156 20180420 AME 11 GII.P31 GII.4s
MN461124 20171213 AME 05 GII.P31 GII.4s
MN461127 20171222 MWE 02 GII.P31 GII.4s
MN461020 20140512 EL03 04 GII.P31 GII.4s
KX764872 20160118 EL03A 03 GII.P31 GII.4s
MN461107 20160224 FI 01 GII.P31 GII.4s
KX764873 20160122 FL07 01 GII.P31 GII.4s
MN461023 20150113 DL01 02 GII.P31 GII.4s
KX764870 20150328 FL07A 02 GII.P31 GII.4s
MN461110 20160325 GI 01 GII.P31 GII.4s
MN461018 20140331 FL01 04 GII.P31 GII.4s
MN461095 20151214 EI 01 GII.P31 GII.4s
MN461106 20160223 EI 04 GII.P31 GII.4s
KX764871 20150330 EL03 01 GII.P31 GII.4s
MN461102 20160122 FI 01 GII.P31 GII.4s
GII.P31/GII.4s
KX764856 20160122 FL07A 03 GII.P16 GII.4ns
MN461109 20160325 FI 03 GII.P16 GII.4ns
KX764849 20160118 EL03A 02 GII.P16 GII.4ns
MN461058 20160327 EL02D 02 GII.P16 GII.4ns
MN461096 20151222 FI 02 GII.P16 GII.4ns
KX764852 20160122 FL01A 01 GII.P16 GII.4ns
MN461099 20160115 GI 02 GII.P16 GII.4ns
KX764848 20160118 EL03 02 GII.P16 GII.4ns
KX764855 20160122 FL07 02 GII.P16 GII.4ns
MN461057 20160325 FL07 01 GII.P16 GII.4ns
MN461113 20160526 FI 03 GII.P16 GII.4ns
MN461122 20171211 ASE 04 GII.P16 GII.4ns
KX764850 20160118 EL03B 02 GII.P16 GII.4ns
MN525219 20160122 FL07A 01 GII.P7 GII.6-4R
KX764854 20160122 FL01D 01 GII.P16 GII.4ns
KX764851 20160118 EL06 01 GII.P16 GII.4ns
KX764853 20160122 FL01C 01 GII.P16 GII.4ns
MN461055 20160325 FL07A 01 GII.P16 GII.4ns
MN461105 20160215 DI 10 GII.P16 GII.4ns
MN461111 20160521 GI 03 GII.P16 GII.4ns
MN461121 20171208 FE 06 GII.P16 GII.4ns
GII.P16/GII.4ns + GII.P7/GII.6-4R
MN461052 20150328 FL07A 01 GII.P7 GII.6c
MN461093 20150917 FI 02 GII.P7 GII.6c
MN461022 20140708 EL04 02 GII.P7 GII.6c
MN461050 20150328 FL01A 01 GII.P7 GII.6c
GII.P7/GII.6c
MN461017 20140329 GL03 02 GII.P7 GII.6a
MN461053 20150328 FL07 01 GII.P7 GII.6a
GII.P7/GII.6a
MN461097 20151222 FI 06 GII.P7 GII.6b
MN461133 20180213 ASE 05 GII.P7 GII.6b
MN461159 20180425 ASE 02 GII.P7 GII.6b
GII.P7/GII.6b
M87661 Norwalk GI.P1 GI.1
0.05

Fig S8

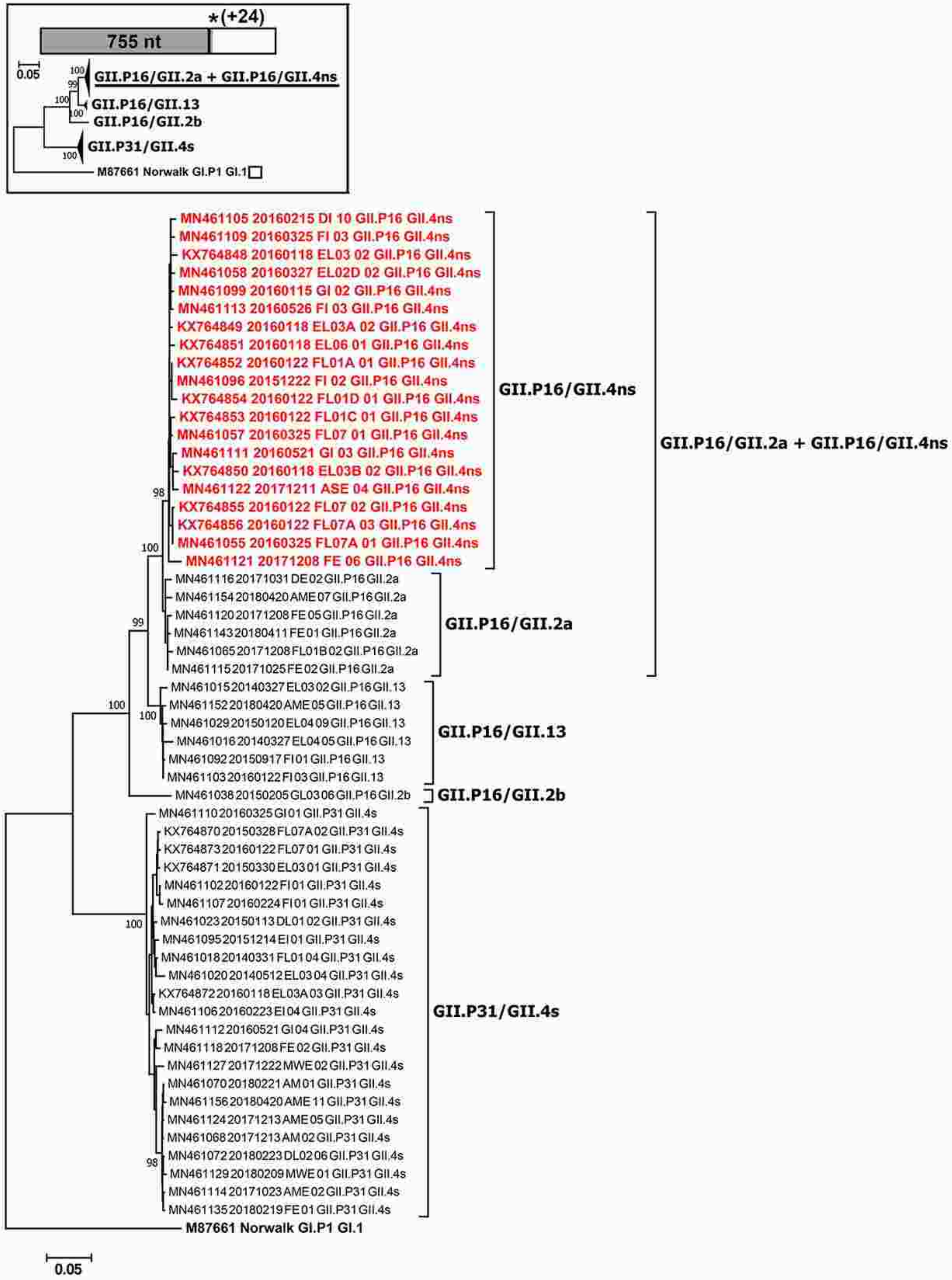

*(+24)
755 nt
0.05
GII.P16/GII.2a + GII.P16/GII.4ns
GII.P16/GII.13
GII.P16/GII.2b
GII.P31/GII.4s
M87661 Norwalk GI.P1 GI.1
MN461105 20160215 DI 10 GII.P16 GII.4ns
MN461109 20160325 FI 03 GII.P16 GII.4ns
KX764848 20160118 EL03 02 GII.P16 GII.4ns
MN461058 20160327 EL02D 02 GII.P16 GII.4ns
MN461099 20160115 GI 02 GII.P16 GII.4ns
MN461113 20160526 FI 03 GII.P16 GII.4ns
KX764849 20160118 EL03A 02 GII.P16 GII.4ns
KX764851 20160118 EL06 01 GII.P16 GII.4ns
KX764852 20160122 FL01A 01 GII.P16 GII.4ns
MN461096 20151222 FI 02 GII.P16 GII.4ns
KX764854 20160122 FL01D 01 GII.P16 GII.4ns
KX764853 20160122 FL01C 01 GII.P16 GII.4ns
MN461057 20160325 FL07 01 GII.P16 GII.4ns
MN461111 20160521 GI 03 GII.P16 GII.4ns
KX764850 20160118 EL03B 02 GII.P16 GII.4ns
MN461122 20171211 ASE 04 GII.P16 GII.4ns
KX764855 20160122 FL07 02 GII.P16 GII.4ns
KX764856 20160122 FL07A 03 GII.P16 GII.4ns
MN461055 20160325 FL07A 01 GII.P16 GII.4ns
MN461121 20171208 FE 06 GII.P16 GII.4ns
GII.P16/GII.4ns
GII.P16/GII.2a + GII.P16/GII.4ns
MN461116 20171031 DE 02 GII.P16 GII.2a
MN461154 20180420 AME 07 GII.P16 GII.2a
MN461120 20171208 FE 05 GII.P16 GII.2a
MN461143 20180411 FE 01 GII.P16 GII.2a
MN461065 20171208 FL01B 02 GII.P16 GII.2a
MN461115 20171025 FE 02 GII.P16 GII.2a
GII.P16/GII.2a
MN461015 20140327 EL03 02 GII.P16 GII.13
MN461152 20180420 AME 05 GII.P16 GII.13
MN461029 20150120 EL04 09 GII.P16 GII.13
MN461016 20140327 EL04 05 GII.P16 GII.13
MN461092 20150917 FI 01 GII.P16 GII.13
MN461103 20160122 FI 03 GII.P16 GII.13
GII.P16/GII.13
MN461038 20150205 GL03 06 GII.P16 GII.2b
GII.P16/GII.2b
MN461110 20160325 GI 01 GII.P31 GII.4s
KX764870 20150328 FL07A 02 GII.P31 GII.4s
KX764873 20160122 FL07 01 GII.P31 GII.4s
KX764871 20150330 EL03 01 GII.P31 GII.4s
MN461102 20160122 FI 01 GII.P31 GII.4s
MN461107 20160224 FI 01 GII.P31 GII.4s
MN461023 20150113 DL01 02 GII.P31 GII.4s
MN461095 20151214 EI 01 GII.P31 GII.4s
MN461018 20140331 FL01 04 GII.P31 GII.4s
MN461020 20140512 EL03 04 GII.P31 GII.4s
KX764872 20160118 EL03A 03 GII.P31 GII.4s
MN461106 20160223 EI 04 GII.P31 GII.4s
MN461112 20160521 GI 04 GII.P31 GII.4s
MN461118 20171208 FE 02 GII.P31 GII.4s
MN461127 20171222 MWE 02 GII.P31 GII.4s
MN461070 20180221 AM 01 GII.P31 GII.4s
MN461156 20180420 AME 11 GII.P31 GII.4s
MN461124 20171213 AME 05 GII.P31 GII.4s
MN461068 20171213 AM 02 GII.P31 GII.4s
MN461072 20180223 DL02 06 GII.P31 GII.4s
MN461129 20180209 MWE 01 GII.P31 GII.4s
MN461114 20171023 AME 02 GII.P31 GII.4s
MN461135 20180219 FE 01 GII.P31 GII.4s
GII.P31/GII.4s
M87661 Norwalk GI.P1 GI.1
0.05

Fig S8

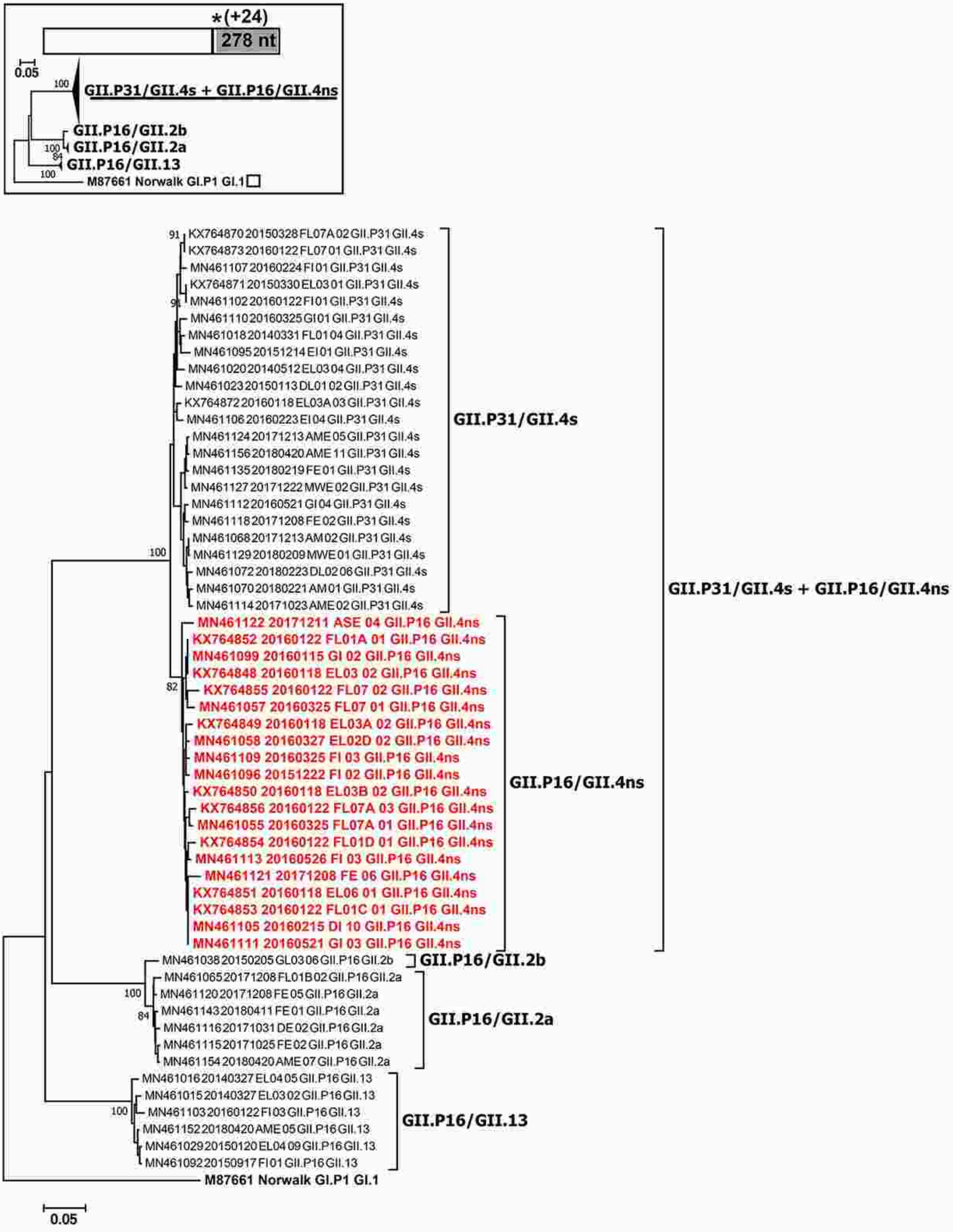
*(+24)
278 nt
0.05
100
GII.P31/GII.4s + GII.P16/GII.4ns
GII.P16/GII.2b
100
84
GII.P16/GII.2a
GII.P16/GII.13
100
M87661 Norwalk GI.P1 GI.1
91
KX764870 20150328 FL07A 02 GII.P31 GII.4s
KX764873 20160122 FL07 01 GII.P31 GII.4s
MN461107 20160224 FI 01 GII.P31 GII.4s
KX764871 20150330 EL03 01 GII.P31 GII.4s
91
MN461102 20160122 FI 01 GII.P31 GII.4s
MN461110 20160325 GI 01 GII.P31 GII.4s
MN461018 20140331 FL01 04 GII.P31 GII.4s
MN461095 20151214 EI 01 GII.P31 GII.4s
MN461020 20140512 EL03 04 GII.P31 GII.4s
MN461023 20150113 DL01 02 GII.P31 GII.4s
KX764872 20160118 EL03A 03 GII.P31 GII.4s
MN461106 20160223 EI 04 GII.P31 GII.4s
MN461124 20171213 AME 05 GII.P31 GII.4s
MN461156 20180420 AME 11 GII.P31 GII.4s
MN461135 20180219 FE 01 GII.P31 GII.4s
MN461127 20171222 MWE 02 GII.P31 GII.4s
MN461112 20160521 GI 04 GII.P31 GII.4s
MN461118 20171208 FE 02 GII.P31 GII.4s
MN461068 20171213 AM 02 GII.P31 GII.4s
100
MN461129 20180209 MWE 01 GII.P31 GII.4s
MN461072 20180223 DL02 06 GII.P31 GII.4s
MN461070 20180221 AM 01 GII.P31 GII.4s
MN461114 20171023 AME 02 GII.P31 GII.4s
GII.P31/GII.4s
MN461122 20171211 ASE 04 GII.P16 GII.4ns
KX764852 20160122 FL01A 01 GII.P16 GII.4ns
MN461099 20160115 GI 02 GII.P16 GII.4ns
KX764848 20160118 EL03 02 GII.P16 GII.4ns
82
KX764855 20160122 FL07 02 GII.P16 GII.4ns
MN461057 20160325 FL07 01 GII.P16 GII.4ns
KX764849 20160118 EL03A 02 GII.P16 GII.4ns
MN461058 20160327 EL02D 02 GII.P16 GII.4ns
MN461109 20160325 FI 03 GII.P16 GII.4ns
MN461096 20151222 FI 02 GII.P16 GII.4ns
KX764850 20160118 EL03B 02 GII.P16 GII.4ns
KX764856 20160122 FL07A 03 GII.P16 GII.4ns
MN461055 20160325 FL07A 01 GII.P16 GII.4ns
KX764854 20160122 FL01D 01 GII.P16 GII.4ns
MN461113 20160526 FI 03 GII.P16 GII.4ns
MN461121 20171208 FE 06 GII.P16 GII.4ns
KX764851 20160118 EL06 01 GII.P16 GII.4ns
KX764853 20160122 FL01C 01 GII.P16 GII.4ns
MN461105 20160215 DI 10 GII.P16 GII.4ns
MN461111 20160521 GI 03 GII.P16 GII.4ns
GII.P16/GII.4ns
GII.P31/GII.4s + GII.P16/GII.4ns
MN461038 20150205 GL03 06 GII.P16 GII.2b
GII.P16/GII.2b
MN461065 20171208 FL01B 02 GII.P16 GII.2a
100
MN461120 20171208 FE 05 GII.P16 GII.2a
MN461143 20180411 FE 01 GII.P16 GII.2a
84
MN461116 20171031 DE 02 GII.P16 GII.2a
MN461115 20171025 FE 02 GII.P16 GII.2a
MN461154 20180420 AME 07 GII.P16 GII.2a
GII.P16/GII.2a
MN461016 20140327 EL04 05 GII.P16 GII.13
MN461015 20140327 EL03 02 GII.P16 GII.13
100
MN461103 20160122 FI 03 GII.P16 GII.13
MN461152 20180420 AME 05 GII.P16 GII.13
MN461029 20150120 EL04 09 GII.P16 GII.13
MN461092 20150917 FI 01 GII.P16 GII.13
GII.P16/GII.13
M87661 Norwalk GI.P1 GI.1
0.05

Fig S8

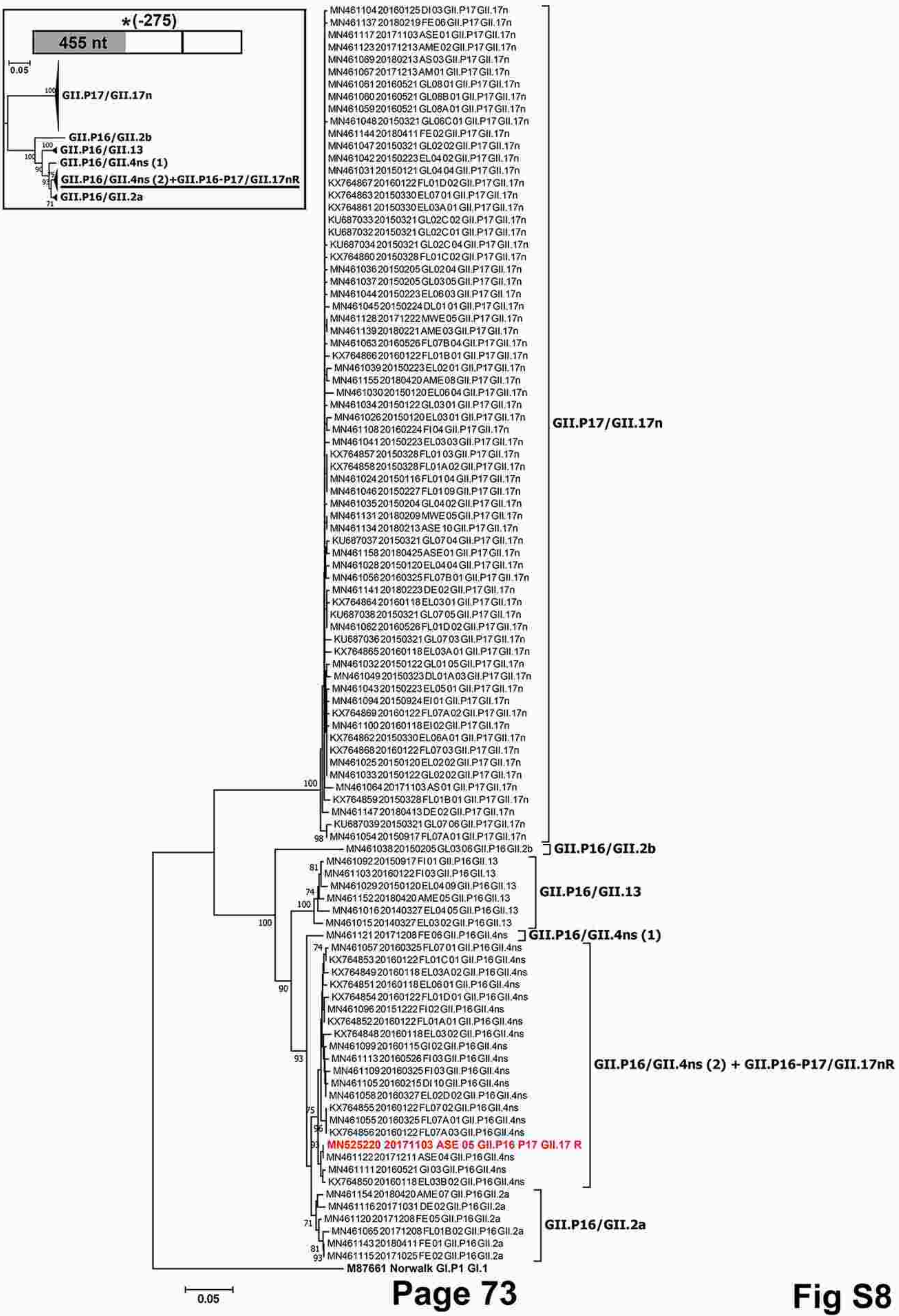

*(-275)
455 nt
0.05
100
GII.P17/GII.17n
GII.P16/GII.2b
100
GII.P16/GII.13
100
GII.P16/GII.4ns (1)
90
75
93
GII.P16/GII.4ns (2)+GII.P16-P17/GII.17nR
GII.P16/GII.2a
71
MN461104 20160125 DI 03 GII.P17 GII.17n
MN461137 20180219 FE 06 GII.P17 GII.17n
MN461117 20171103 ASE 01 GII.P17 GII.17n
MN461123 20171213 AME 02 GII.P17 GII.17n
MN461069 20180213 AS 03 GII.P17 GII.17n
MN461067 20171213 AM 01 GII.P17 GII.17n
MN461061 20160521 GL08 01 GII.P17 GII.17n
MN461060 20160521 GL08B 01 GII.P17 GII.17n
MN461059 20160521 GL08A 01 GII.P17 GII.17n
MN461048 20150321 GL06C 01 GII.P17 GII.17n
MN461144 20180411 FE 02 GII.P17 GII.17n
MN461047 20150321 GL02 02 GII.P17 GII.17n
MN461042 20150223 EL04 02 GII.P17 GII.17n
MN461031 20150121 GL04 04 GII.P17 GII.17n
KX764867 20160122 FL01D 02 GII.P17 GII.17n
KX764863 20150330 EL07 01 GII.P17 GII.17n
KX764861 20150330 EL03A 01 GII.P17 GII.17n
KU687033 20150321 GL02C 02 GII.P17 GII.17n
KU687032 20150321 GL02C 01 GII.P17 GII.17n
KU687034 20150321 GL02C 04 GII.P17 GII.17n
KX764860 20150328 FL01C 02 GII.P17 GII.17n
MN461036 20150205 GL02 04 GII.P17 GII.17n
MN461037 20150205 GL03 05 GII.P17 GII.17n
MN461044 20150223 EL06 03 GII.P17 GII.17n
MN461045 20150224 DL01 01 GII.P17 GII.17n
MN461128 20171222 MWE 05 GII.P17 GII.17n
MN461139 20180221 AME 03 GII.P17 GII.17n
MN461063 20160526 FL07B 04 GII.P17 GII.17n
KX764866 20160122 FL01B 01 GII.P17 GII.17n
MN461039 20150223 EL02 01 GII.P17 GII.17n
MN461155 20180420 AME 08 GII.P17 GII.17n
MN461030 20150120 EL06 04 GII.P17 GII.17n
MN461034 20150122 GL03 01 GII.P17 GII.17n
MN461026 20150120 EL03 01 GII.P17 GII.17n
MN461108 20160224 FI 04 GII.P17 GII.17n
MN461041 20150223 EL03 03 GII.P17 GII.17n
KX764857 20150328 FL01 03 GII.P17 GII.17n
KX764858 20150328 FL01A 02 GII.P17 GII.17n
MN461024 20150116 FL01 04 GII.P17 GII.17n
MN461046 20150227 FL01 09 GII.P17 GII.17n
MN461035 20150204 GL04 02 GII.P17 GII.17n
MN461131 20180209 MWE 05 GII.P17 GII.17n
MN461134 20180213 ASE 10 GII.P17 GII.17n
KU687037 20150321 GL07 04 GII.P17 GII.17n
MN461158 20180425 ASE 01 GII.P17 GII.17n
MN461028 20150120 EL04 04 GII.P17 GII.17n
MN461056 20160325 FL07B 01 GII.P17 GII.17n
MN461141 20180223 DE 02 GII.P17 GII.17n
KX764864 20160118 EL03 01 GII.P17 GII.17n
KU687038 20150321 GL07 05 GII.P17 GII.17n
MN461062 20160526 FL01D 02 GII.P17 GII.17n
KU687036 20150321 GL07 03 GII.P17 GII.17n
KX764865 20160118 EL03A 01 GII.P17 GII.17n
MN461032 20150122 GL01 05 GII.P17 GII.17n
MN461049 20150323 DL01A 03 GII.P17 GII.17n
MN461043 20150223 EL05 01 GII.P17 GII.17n
MN461094 20150924 EI 01 GII.P17 GII.17n
KX764869 20160122 FL07A 02 GII.P17 GII.17n
MN461100 20160118 EI 02 GII.P17 GII.17n
KX764862 20150330 EL06A 01 GII.P17 GII.17n
KX764868 20160122 FL07 03 GII.P17 GII.17n
MN461025 20150120 EL02 02 GII.P17 GII.17n
MN461033 20150122 GL02 02 GII.P17 GII.17n
100
MN461064 20171103 AS 01 GII.P17 GII.17n
KX764859 20150328 FL01B 01 GII.P17 GII.17n
MN461147 20180413 DE 02 GII.P17 GII.17n
KU687039 20150321 GL07 06 GII.P17 GII.17n
98
MN461054 20150917 FL07A 01 GII.P17 GII.17n
GII.P17/GII.17n
MN461038 20150205 GL03 06 GII.P16 GII.2b
GII.P16/GII.2b
81
MN461092 20150917 FI 01 GII.P16 GII.13
MN461103 20160122 FI 03 GII.P16 GII.13
MN461029 20150120 EL04 09 GII.P16 GII.13
74
MN461152 20180420 AME 05 GII.P16 GII.13
100
MN461016 20140327 EL04 05 GII.P16 GII.13
100
MN461015 20140327 EL03 02 GII.P16 GII.13
GII.P16/GII.13
MN461121 20171208 FE 06 GII.P16 GII.4ns
GII.P16/GII.4ns (1)
74
MN461057 20160325 FL07 01 GII.P16 GII.4ns
KX764853 20160122 FL01C 01 GII.P16 GII.4ns
KX764849 20160118 EL03A 02 GII.P16 GII.4ns
KX764851 20160118 EL06 01 GII.P16 GII.4ns
90
KX764854 20160122 FL01D 01 GII.P16 GII.4ns
MN461096 20151222 FI 02 GII.P16 GII.4ns
KX764852 20160122 FL01A 01 GII.P16 GII.4ns
KX764848 20160118 EL03 02 GII.P16 GII.4ns
MN461099 20160115 GI 02 GII.P16 GII.4ns
93
MN461113 20160526 FI 03 GII.P16 GII.4ns
MN461109 20160325 FI 03 GII.P16 GII.4ns
MN461105 20160215 DI 10 GII.P16 GII.4ns
MN461058 20160327 EL02D 02 GII.P16 GII.4ns
75
KX764855 20160122 FL07 02 GII.P16 GII.4ns
MN461055 20160325 FL07A 01 GII.P16 GII.4ns
96
KX764856 20160122 FL07A 03 GII.P16 GII.4ns
93
MN525220 20171103 ASE 05 GII.P16 P17 GII.17 R
MN461122 20171211 ASE 04 GII.P16 GII.4ns
MN461111 20160521 GI 03 GII.P16 GII.4ns
KX764850 20160118 EL03B 02 GII.P16 GII.4ns
GII.P16/GII.4ns (2) + GII.P16-P17/GII.17nR
MN461154 20180420 AME 07 GII.P16 GII.2a
MN461116 20171031 DE 02 GII.P16 GII.2a
71
MN461120 20171208 FE 05 GII.P16 GII.2a
MN461065 20171208 FL01B 02 GII.P16 GII.2a
81
MN461143 20180411 FE 01 GII.P16 GII.2a
93
MN461115 20171025 FE 02 GII.P16 GII.2a
GII.P16/GII.2a
M87661 Norwalk GI.P1 GI.1
0.05

Fig S8

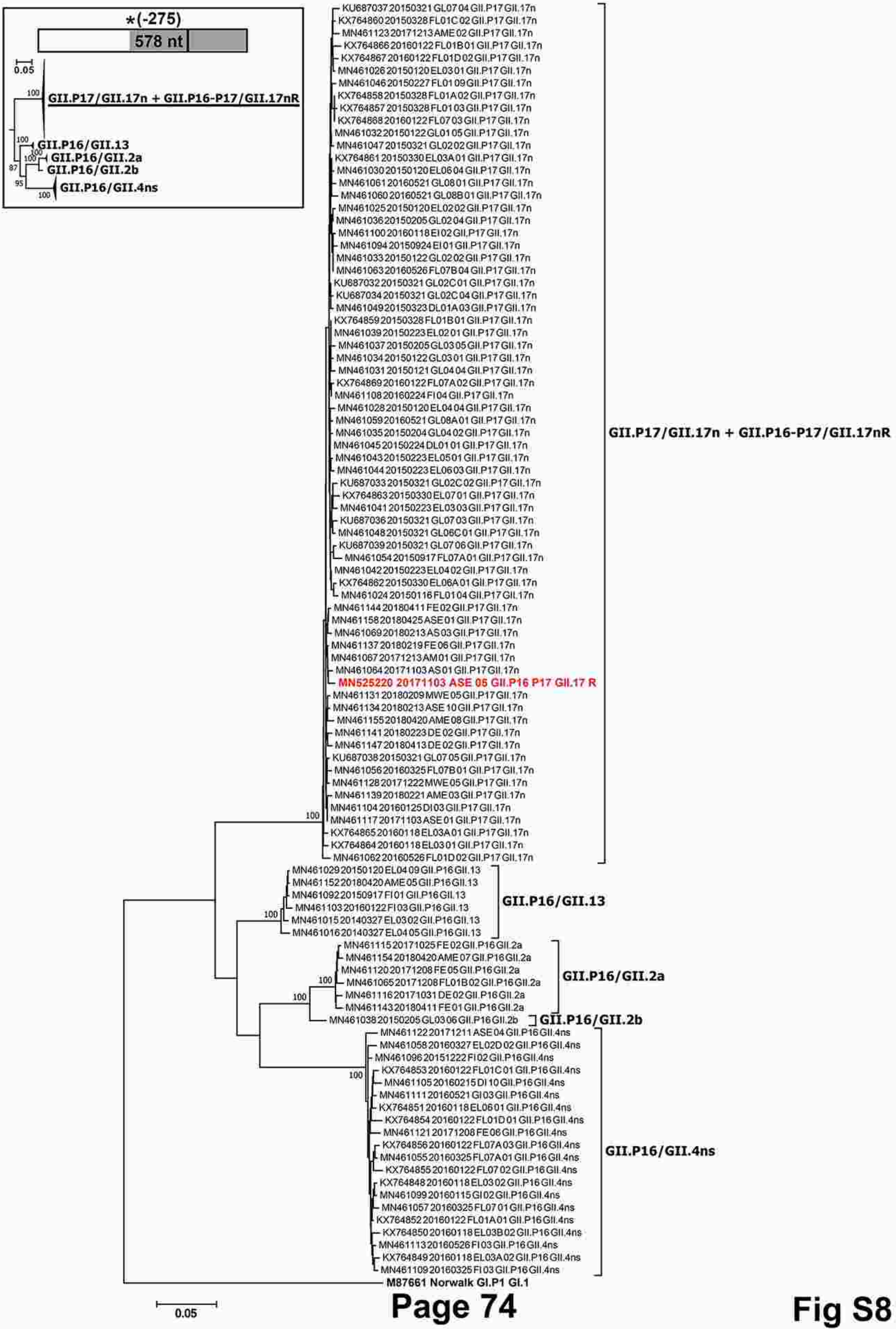

*(-275)
578 nt
0.05
GII.P17/GII.17n + GII.P16-P17/GII.17nR
GII.P16/GII.13
GII.P16/GII.2a
GII.P16/GII.2b
GII.P16/GII.4ns
100
87
95
KU687037 20150321 GL07 04 GII.P17 GII.17n
KX764860 20150328 FL01C 02 GII.P17 GII.17n
MN461123 20171213 AME 02 GII.P17 GII.17n
KX764866 20160122 FL01B 01 GII.P17 GII.17n
KX764867 20160122 FL01D 02 GII.P17 GII.17n
MN461026 20150120 EL03 01 GII.P17 GII.17n
MN461046 20150227 FL01 09 GII.P17 GII.17n
KX764858 20150328 FL01A 02 GII.P17 GII.17n
KX764857 20150328 FL01 03 GII.P17 GII.17n
KX764868 20160122 FL07 03 GII.P17 GII.17n
MN461032 20150122 GL01 05 GII.P17 GII.17n
MN461047 20150321 GL02 02 GII.P17 GII.17n
KX764861 20150330 EL03A 01 GII.P17 GII.17n
MN461030 20150120 EL06 04 GII.P17 GII.17n
MN461061 20160521 GL08 01 GII.P17 GII.17n
MN461060 20160521 GL08B 01 GII.P17 GII.17n
MN461025 20150120 EL02 02 GII.P17 GII.17n
MN461036 20150205 GL02 04 GII.P17 GII.17n
MN461100 20160118 EI 02 GII.P17 GII.17n
MN461094 20150924 EI 01 GII.P17 GII.17n
MN461033 20150122 GL02 02 GII.P17 GII.17n
MN461063 20160526 FL07B 04 GII.P17 GII.17n
KU687032 20150321 GL02C 01 GII.P17 GII.17n
KU687034 20150321 GL02C 04 GII.P17 GII.17n
MN461049 20150323 DL01A 03 GII.P17 GII.17n
KX764859 20150328 FL01B 01 GII.P17 GII.17n
MN461039 20150223 EL02 01 GII.P17 GII.17n
MN461037 20150205 GL03 05 GII.P17 GII.17n
MN461034 20150122 GL03 01 GII.P17 GII.17n
MN461031 20150121 GL04 04 GII.P17 GII.17n
KX764869 20160122 FL07A 02 GII.P17 GII.17n
MN461108 20160224 FI 04 GII.P17 GII.17n
MN461028 20150120 EL04 04 GII.P17 GII.17n
MN461059 20160521 GL08A 01 GII.P17 GII.17n
MN461035 20150204 GL04 02 GII.P17 GII.17n
MN461045 20150224 DL01 01 GII.P17 GII.17n
MN461043 20150223 EL05 01 GII.P17 GII.17n
MN461044 20150223 EL06 03 GII.P17 GII.17n
KU687033 20150321 GL02C 02 GII.P17 GII.17n
KX764863 20150330 EL07 01 GII.P17 GII.17n
MN461041 20150223 EL03 03 GII.P17 GII.17n
KU687036 20150321 GL07 03 GII.P17 GII.17n
MN461048 20150321 GL06C 01 GII.P17 GII.17n
KU687039 20150321 GL07 06 GII.P17 GII.17n
MN461054 20150917 FL07A 01 GII.P17 GII.17n
MN461042 20150223 EL04 02 GII.P17 GII.17n
KX764862 20150330 EL06A 01 GII.P17 GII.17n
MN461024 20150116 FL01 04 GII.P17 GII.17n
MN461144 20180411 FE 02 GII.P17 GII.17n
MN461158 20180425 ASE 01 GII.P17 GII.17n
MN461069 20180213 AS 03 GII.P17 GII.17n
MN461137 20180219 FE 06 GII.P17 GII.17n
MN461067 20171213 AM 01 GII.P17 GII.17n
MN461064 20171103 AS 01 GII.P17 GII.17n
MN525220 20171103 ASE 05 GII.P16 P17 GII.17 R
MN461131 20180209 MWE 05 GII.P17 GII.17n
MN461134 20180213 ASE 10 GII.P17 GII.17n
MN461155 20180420 AME 08 GII.P17 GII.17n
MN461141 20180223 DE 02 GII.P17 GII.17n
MN461147 20180413 DE 02 GII.P17 GII.17n
KU687038 20150321 GL07 05 GII.P17 GII.17n
MN461056 20160325 FL07B 01 GII.P17 GII.17n
MN461128 20171222 MWE 05 GII.P17 GII.17n
MN461139 20180221 AME 03 GII.P17 GII.17n
MN461104 20160125 DI 03 GII.P17 GII.17n
MN461117 20171103 ASE 01 GII.P17 GII.17n
KX764865 20160118 EL03A 01 GII.P17 GII.17n
KX764864 20160118 EL03 01 GII.P17 GII.17n
MN461062 20160526 FL01D 02 GII.P17 GII.17n
GII.P17/GII.17n + GII.P16-P17/GII.17nR
MN461029 20150120 EL04 09 GII.P16 GII.13
MN461152 20180420 AME 05 GII.P16 GII.13
MN461092 20150917 FI 01 GII.P16 GII.13
MN461103 20160122 FI 03 GII.P16 GII.13
MN461015 20140327 EL03 02 GII.P16 GII.13
MN461016 20140327 EL04 05 GII.P16 GII.13
GII.P16/GII.13
MN461115 20171025 FE 02 GII.P16 GII.2a
MN461154 20180420 AME 07 GII.P16 GII.2a
MN461120 20171208 FE 05 GII.P16 GII.2a
MN461065 20171208 FL01B 02 GII.P16 GII.2a
MN461116 20171031 DE 02 GII.P16 GII.2a
MN461143 20180411 FE 01 GII.P16 GII.2a
GII.P16/GII.2a
MN461038 20150205 GL03 06 GII.P16 GII.2b
GII.P16/GII.2b
MN461122 20171211 ASE 04 GII.P16 GII.4ns
MN461058 20160327 EL02D 02 GII.P16 GII.4ns
MN461096 20151222 FI 02 GII.P16 GII.4ns
KX764853 20160122 FL01C 01 GII.P16 GII.4ns
MN461105 20160215 DI 10 GII.P16 GII.4ns
MN461111 20160521 GI 03 GII.P16 GII.4ns
KX764851 20160118 EL06 01 GII.P16 GII.4ns
KX764854 20160122 FL01D 01 GII.P16 GII.4ns
MN461121 20171208 FE 06 GII.P16 GII.4ns
KX764856 20160122 FL07A 03 GII.P16 GII.4ns
MN461055 20160325 FL07A 01 GII.P16 GII.4ns
KX764855 20160122 FL07 02 GII.P16 GII.4ns
KX764848 20160118 EL03 02 GII.P16 GII.4ns
MN461099 20160115 GI 02 GII.P16 GII.4ns
MN461057 20160325 FL07 01 GII.P16 GII.4ns
KX764852 20160122 FL01A 01 GII.P16 GII.4ns
KX764850 20160118 EL03B 02 GII.P16 GII.4ns
MN461113 20160526 FI 03 GII.P16 GII.4ns
KX764849 20160118 EL03A 02 GII.P16 GII.4ns
MN461109 20160325 FI 03 GII.P16 GII.4ns
GII.P16/GII.4ns
M87661 Norwalk GI.P1 GI.1
0.05

Fig S8

FIG S8 Expanded views of compressed phylogenetic trees, based on recombination break points, of recombinant sequences with their parental sequences. Both the compressed trees and associated expanded views are shown on the same pages. In each expanded view, recombinant sequences are shown in red font. The name of each recombinant is shown at the bottom of each page. Pages 50, 54, 59, and 70 include vertical scatter plots for complementing low reliability of parental clusters with bootstrap values < 75. In each scatter plot, nucleotide distances (black dot) between recombinant sequence(s) and a parental cluster (left), and between recombinant sequence(s) and its closest non-parental cluster (right) are shown. An asterisk (*) below of the name of a lineage cluster in each vertical scatter plot indicates the existences of most closely related sequences (minimum p-distance) to the recombinant sequences in the cluster. Means (◆) and medians (○) of the distance groups are shown in each vertical scatter plot.

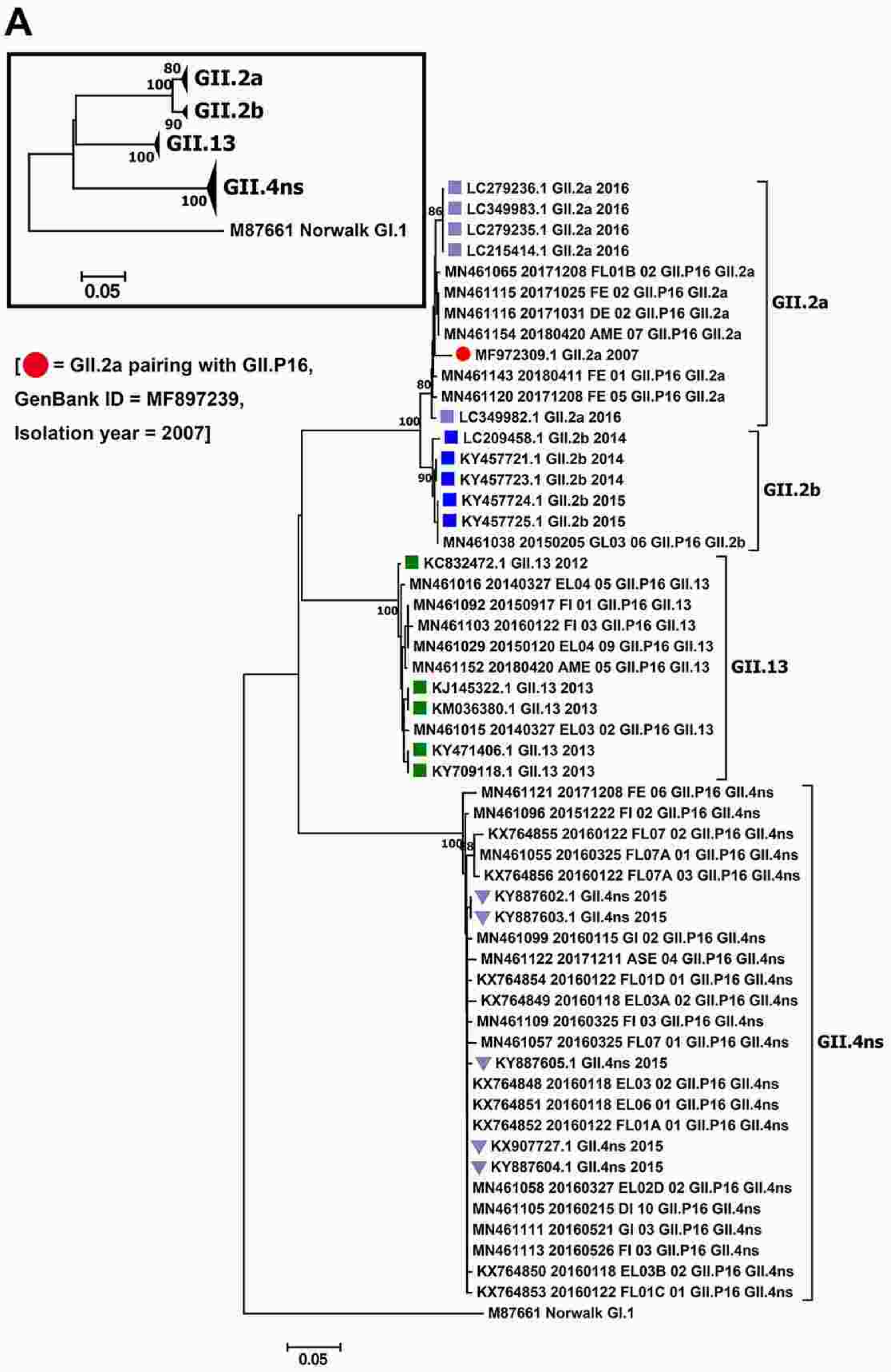
A
80
100
GII.2a
GII.2b
90
GII.13
100
GII.4ns
100
M87661 Norwalk GI.1
0.05
[● = GII.2a pairing with GII.P16,
GenBank ID = MF897239,
Isolation year = 2007]
LC279236.1 GII.2a 2016
86
LC349983.1 GII.2a 2016
LC279235.1 GII.2a 2016
LC215414.1 GII.2a 2016
MN461065 20171208 FL01B 02 GII.P16 GII.2a
MN461115 20171025 FE 02 GII.P16 GII.2a
MN461116 20171031 DE 02 GII.P16 GII.2a
MN461154 20180420 AME 07 GII.P16 GII.2a
MF972309.1 GII.2a 2007
MN461143 20180411 FE 01 GII.P16 GII.2a
80
MN461120 20171208 FE 05 GII.P16 GII.2a
100
LC349982.1 GII.2a 2016
GII.2a
LC209458.1 GII.2b 2014
KY457721.1 GII.2b 2014
90
KY457723.1 GII.2b 2014
KY457724.1 GII.2b 2015
KY457725.1 GII.2b 2015
MN461038 20150205 GL03 06 GII.P16 GII.2b
GII.2b
KC832472.1 GII.13 2012
MN461016 20140327 EL04 05 GII.P16 GII.13
MN461092 20150917 FI 01 GII.P16 GII.13
100
MN461103 20160122 FI 03 GII.P16 GII.13
MN461029 20150120 EL04 09 GII.P16 GII.13
MN461152 20180420 AME 05 GII.P16 GII.13
KJ145322.1 GII.13 2013
KM036380.1 GII.13 2013
MN461015 20140327 EL03 02 GII.P16 GII.13
KY471406.1 GII.13 2013
KY709118.1 GII.13 2013
GII.13
MN461121 20171208 FE 06 GII.P16 GII.4ns
MN461096 20151222 FI 02 GII.P16 GII.4ns
KX764855 20160122 FL07 02 GII.P16 GII.4ns
100
MN461055 20160325 FL07A 01 GII.P16 GII.4ns
KX764856 20160122 FL07A 03 GII.P16 GII.4ns
KY887602.1 GII.4ns 2015
KY887603.1 GII.4ns 2015
MN461099 20160115 GI 02 GII.P16 GII.4ns
MN461122 20171211 ASE 04 GII.P16 GII.4ns
KX764854 20160122 FL01D 01 GII.P16 GII.4ns
KX764849 20160118 EL03A 02 GII.P16 GII.4ns
MN461109 20160325 FI 03 GII.P16 GII.4ns
MN461057 20160325 FL07 01 GII.P16 GII.4ns
KY887605.1 GII.4ns 2015
KX764848 20160118 EL03 02 GII.P16 GII.4ns
KX764851 20160118 EL06 01 GII.P16 GII.4ns
KX764852 20160122 FL01A 01 GII.P16 GII.4ns
KX907727.1 GII.4ns 2015
KY887604.1 GII.4ns 2015
MN461058 20160327 EL02D 02 GII.P16 GII.4ns
MN461105 20160215 DI 10 GII.P16 GII.4ns
MN461111 20160521 GI 03 GII.P16 GII.4ns
MN461113 20160526 FI 03 GII.P16 GII.4ns
KX764850 20160118 EL03B 02 GII.P16 GII.4ns
KX764853 20160122 FL01C 01 GII.P16 GII.4ns
GII.4ns
M87661 Norwalk GI.1
0.05

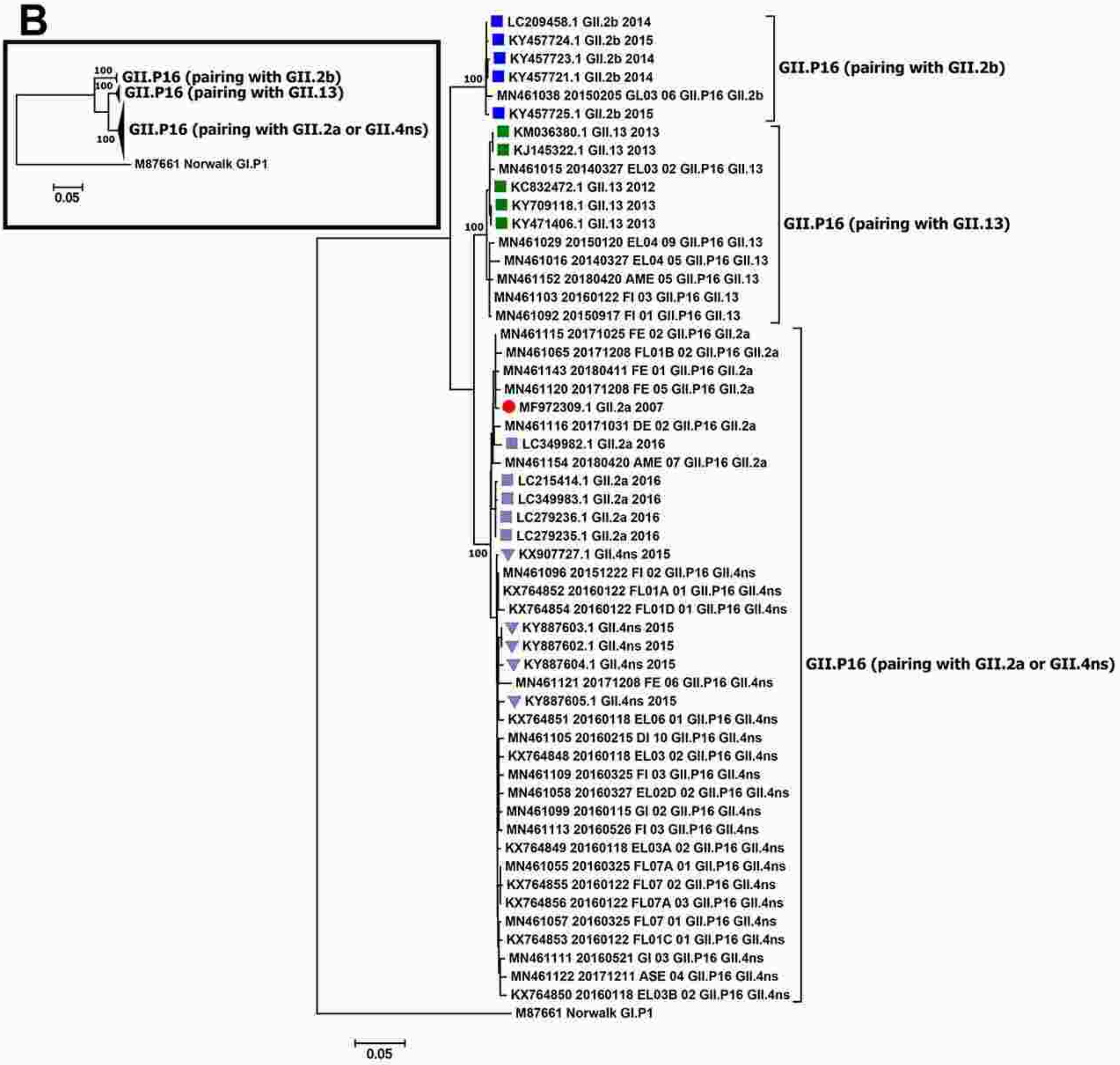

The earliest isolation year of the reference GII.P16/GII.2a (■) with the exception of the MF897239 (●) = 2016

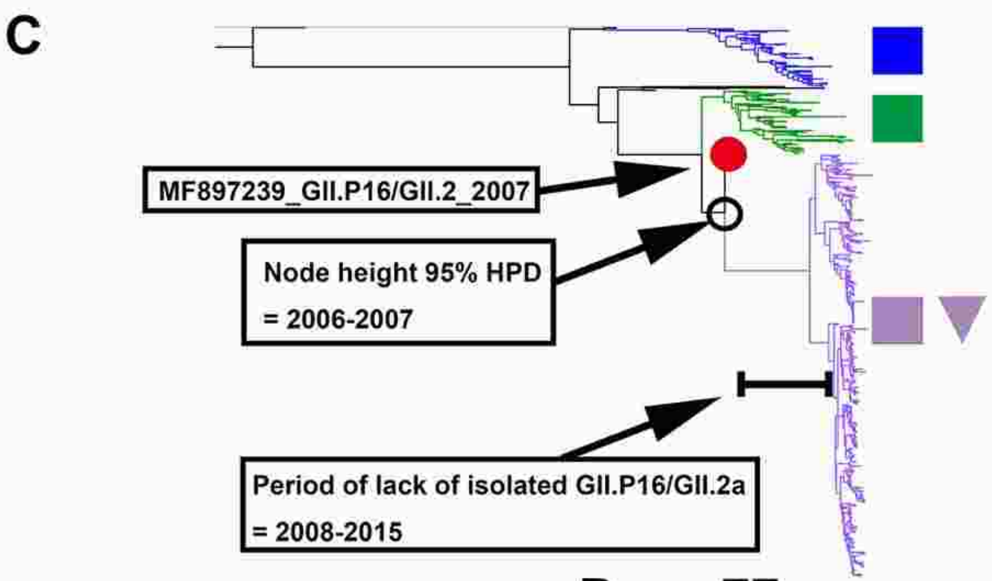

Fig S9

FIG S9 Phylogenetic trees that describe lack of isolated GII.P16/GII.2a lineage in the 2008-2015 period. **(A)** Neighbor-joining trees of genogroup II (GII) region C sequences (0.2 kb) of the isolated ORF1/2 junction sequences: GII.P16/GII.2a, GII.P16/GII.2b, GII.P16/GII.4ns, and GII.P16/GII.13. Reference sequences are shown in the expanded view of the compressed tree [(●; GII.P16/GII.2a; isolation year: 2007), (■; GII.P16/GII.2a; isolation year: 2016), (■; GII.P16/GII.2b; isolation year: 2014-2015), (■; GII.P16/GII.13; isolation year: 2012-2013), and (▼; GII.P16/GII.4ns; isolation year: 2015)]. **(B)** Neighbor-joining trees based on GII.P16 sequences (0.7 kb) of the ORF1/2 junction sequences isolated in this study. An expanded view of the compressed tree is shown including GII.P16 reference sequences, which are from the same reference sequences indicated in panel A. **(C)** An MCC tree of GII.P16 (n = 788, date of data collection = March 2019). Each marker on the right side of each clade indicates the inclusion of the reference sequences correlated with the marker in the clade. A branch of the reference sequence GII.P16/GII.2a (GenBank ID=MF897239, isolation year: 2007) is indicated by the marker (●). An indicated node 95% height posterior density (HPD, 2006-2007) describes the existence of the most recent common ancestor between MF897239 and a clade, including the GII.P16 sequences paired with GII.4ns and GII.2a.

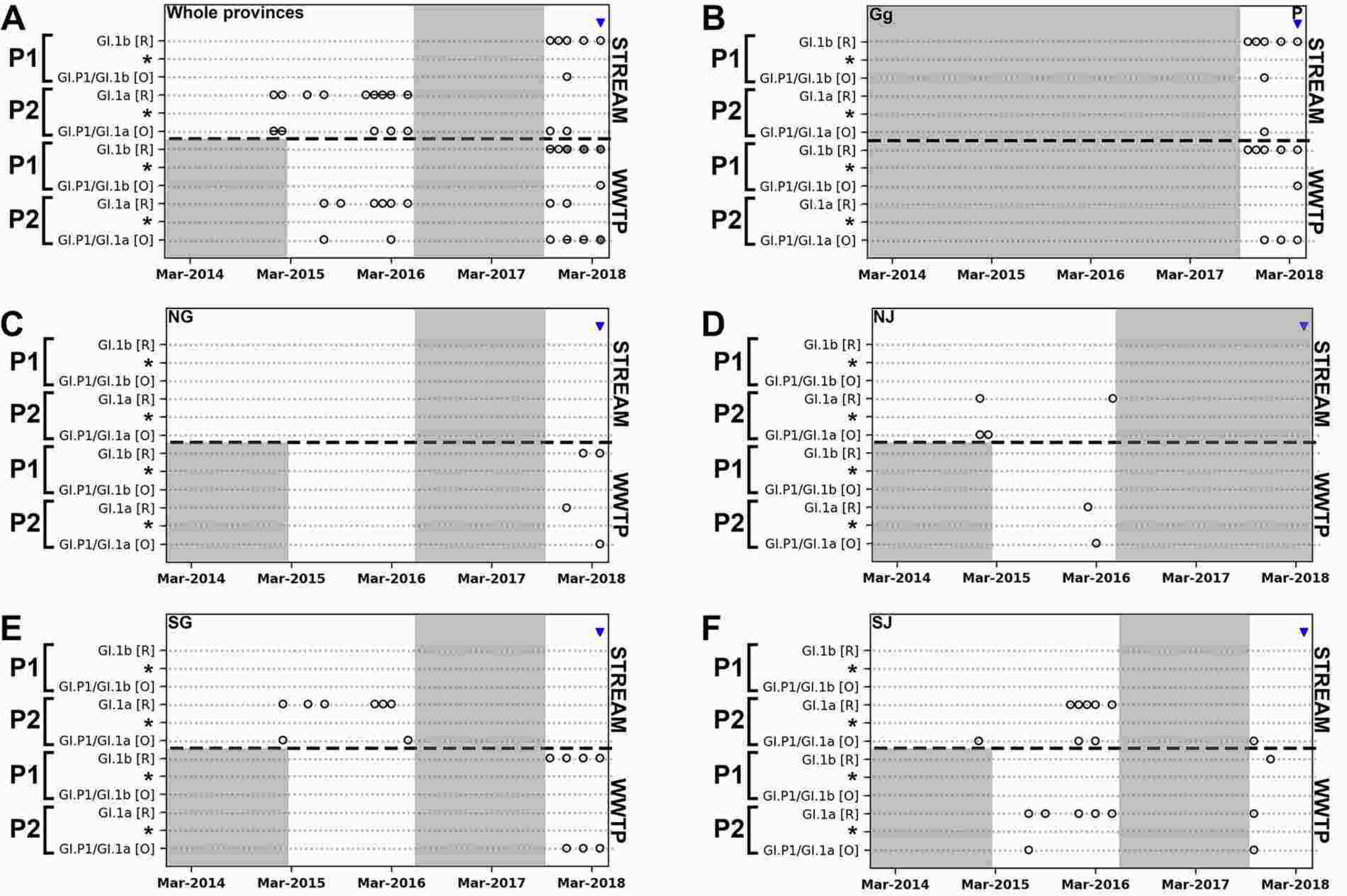

Page 79 (GI.1R)

Fig S10

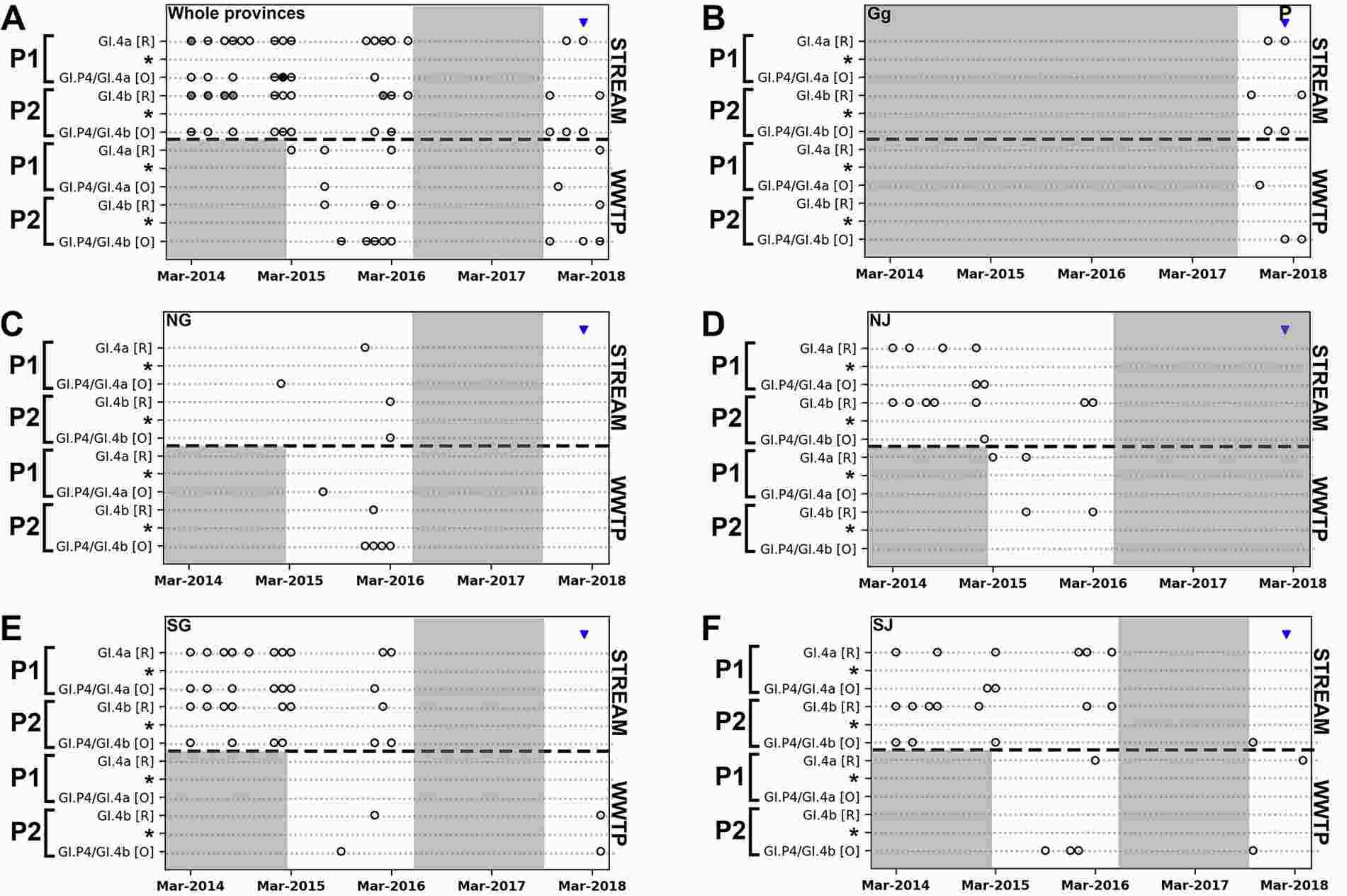

Page 80 (GI.4R)

Fig S10

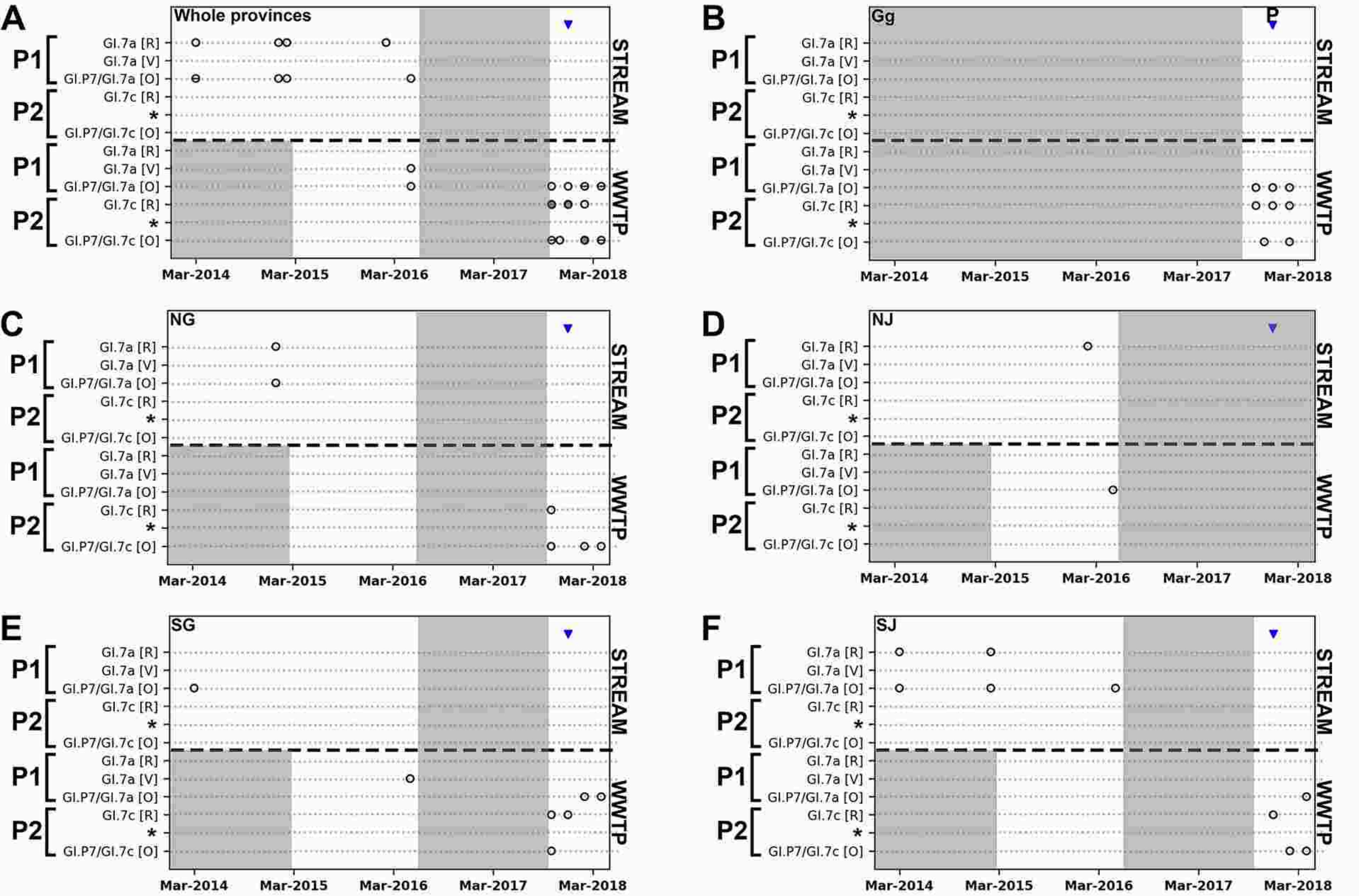
A
Whole provinces
B
Gg
P
C
NG
D
NJ
E
SG
F
SJ
P1
P2
GI.7a [R]
GI.7a [V]
GI.P7/GI.7a [O]
GI.7c [R]
*
GI.P7/GI.7c [O]
STREAM
WWTP
Mar-2014
Mar-2015
Mar-2016
Mar-2017
Mar-2018

Fig S10

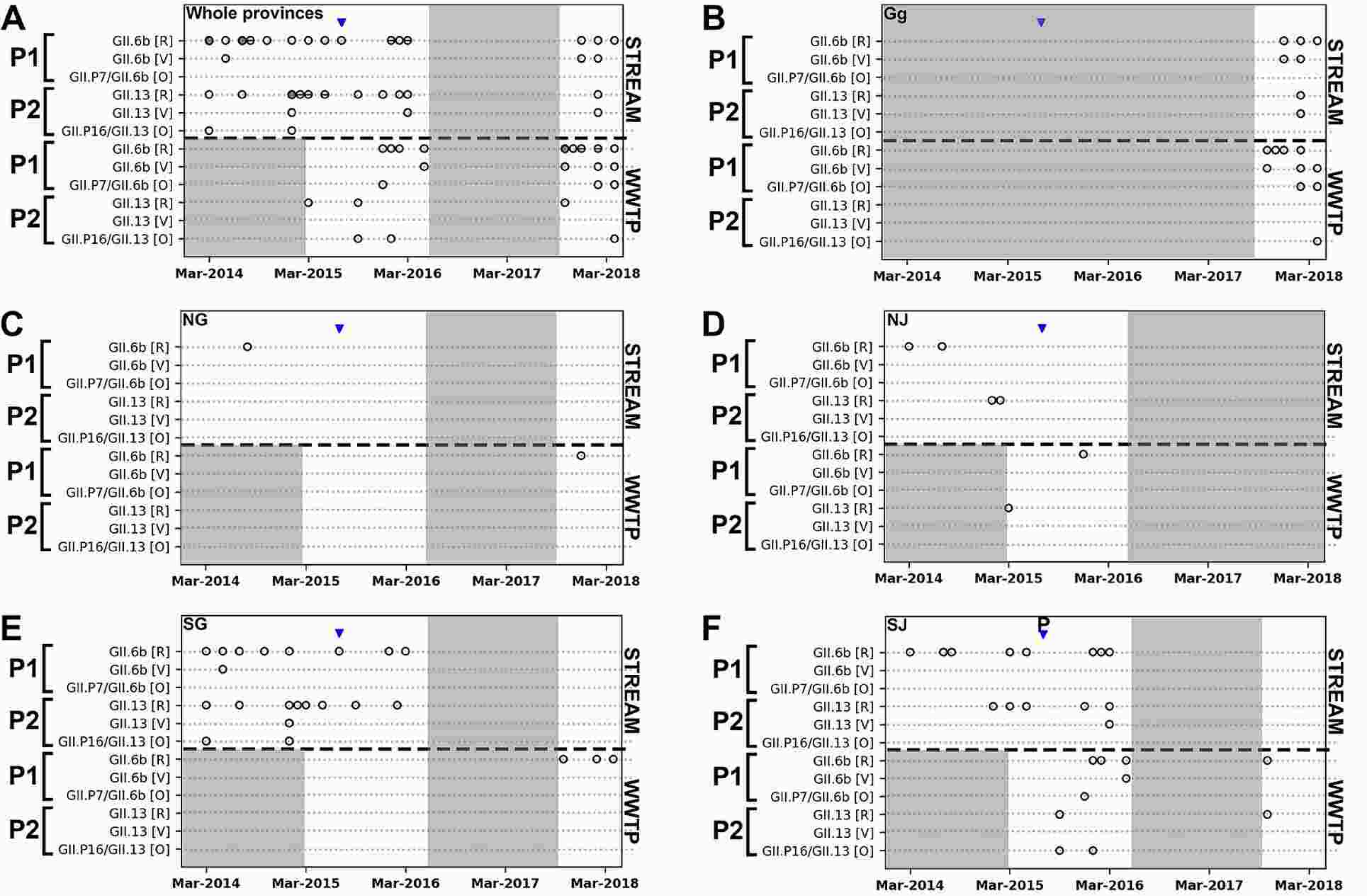

Page 82 (GII.6-13R)

Fig S10

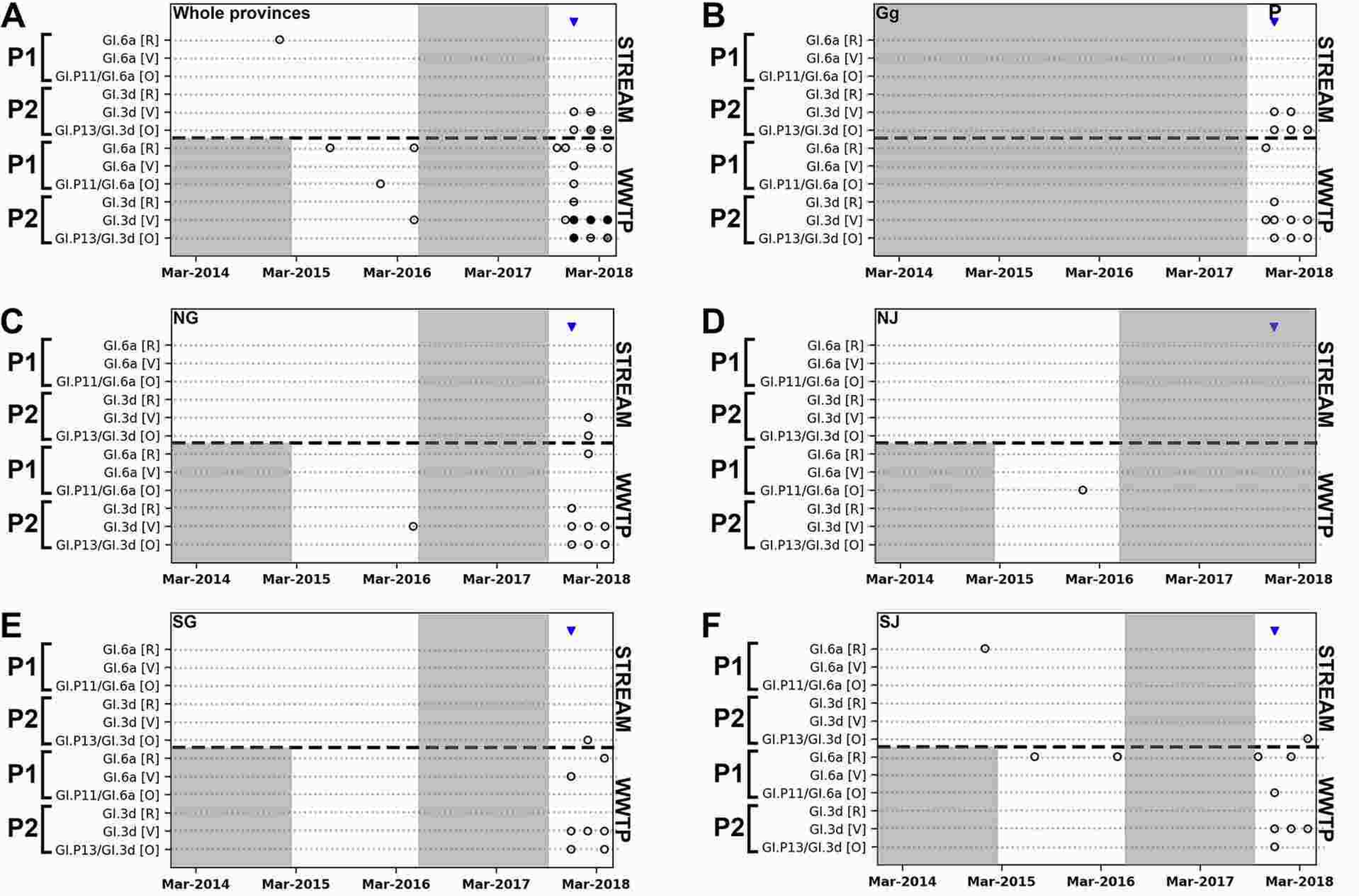

Page 83 (GI.6-3R_LPV)

Fig S10

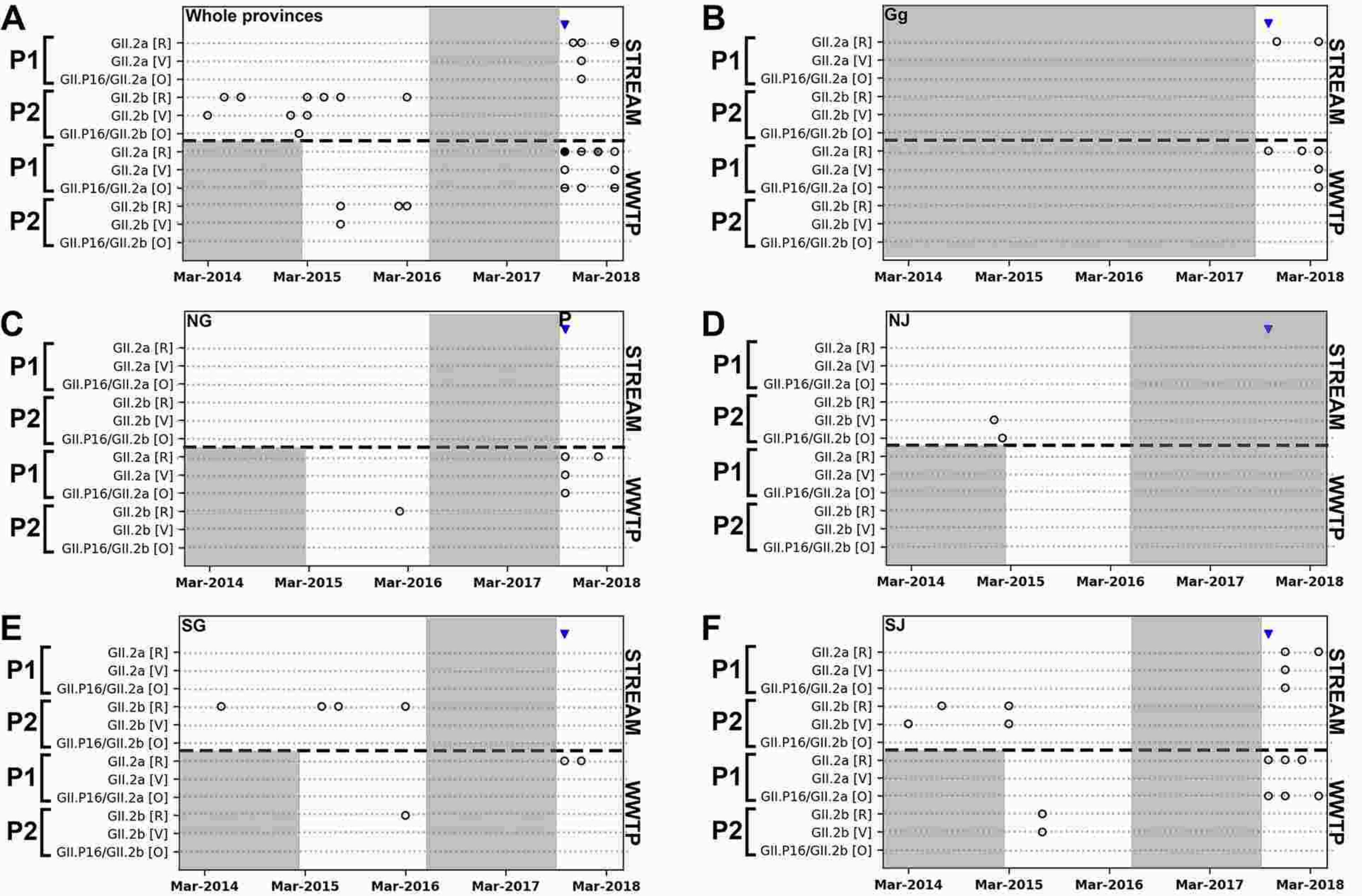
A
Whole provinces
B
Gg
C
NG
P
D
NJ
E
SG
F
SJ
P1
P2
GII.2a [R]
GII.2a [V]
GII.P16/GII.2a [O]
GII.2b [R]
GII.2b [V]
GII.P16/GII.2b [O]
STREAM
WWTP
Mar-2014
Mar-2015
Mar-2016
Mar-2017
Mar-2018

Fig S10

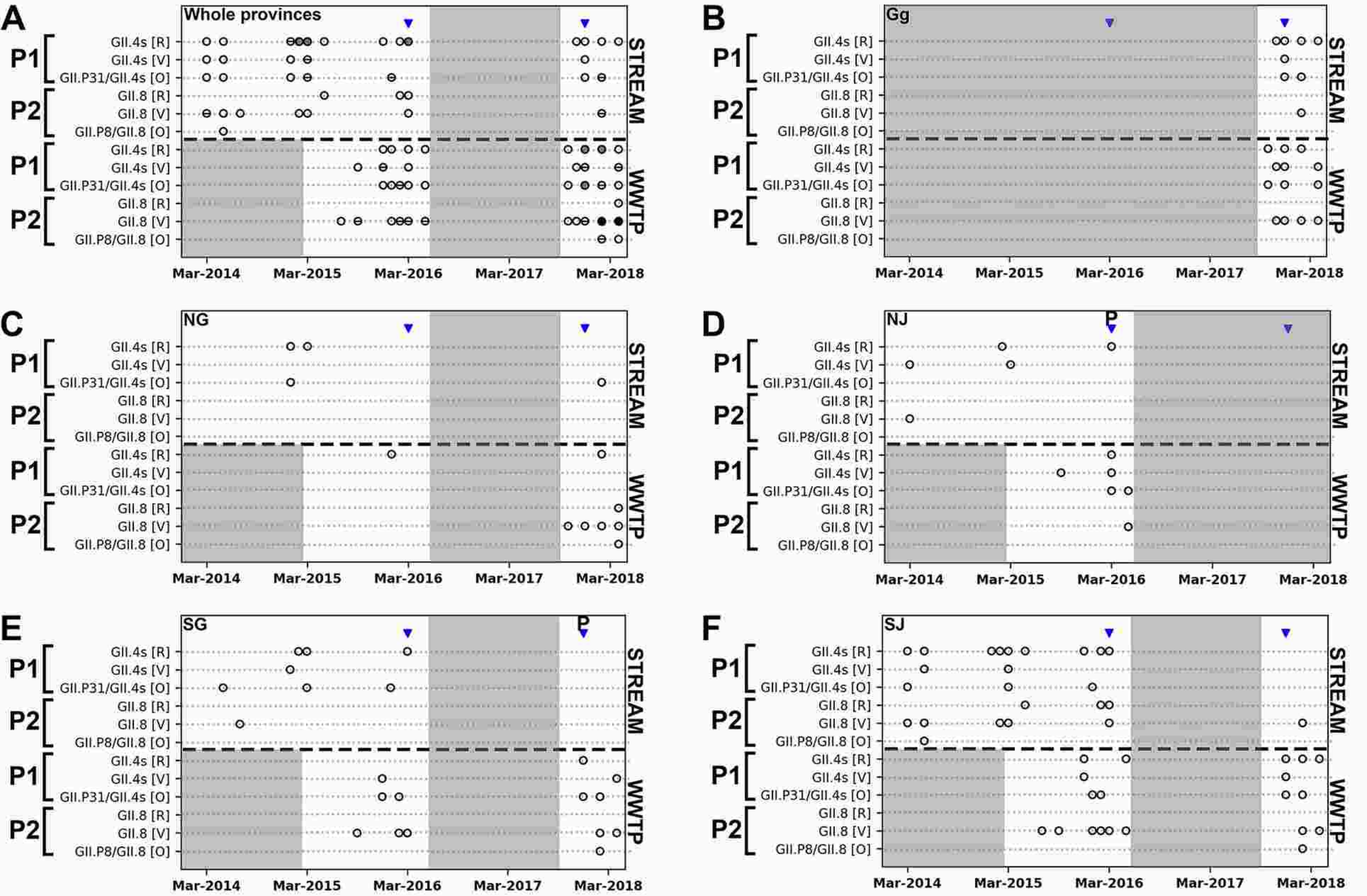

Page 85 (GII.4-8R_LPV) **Fig S10**

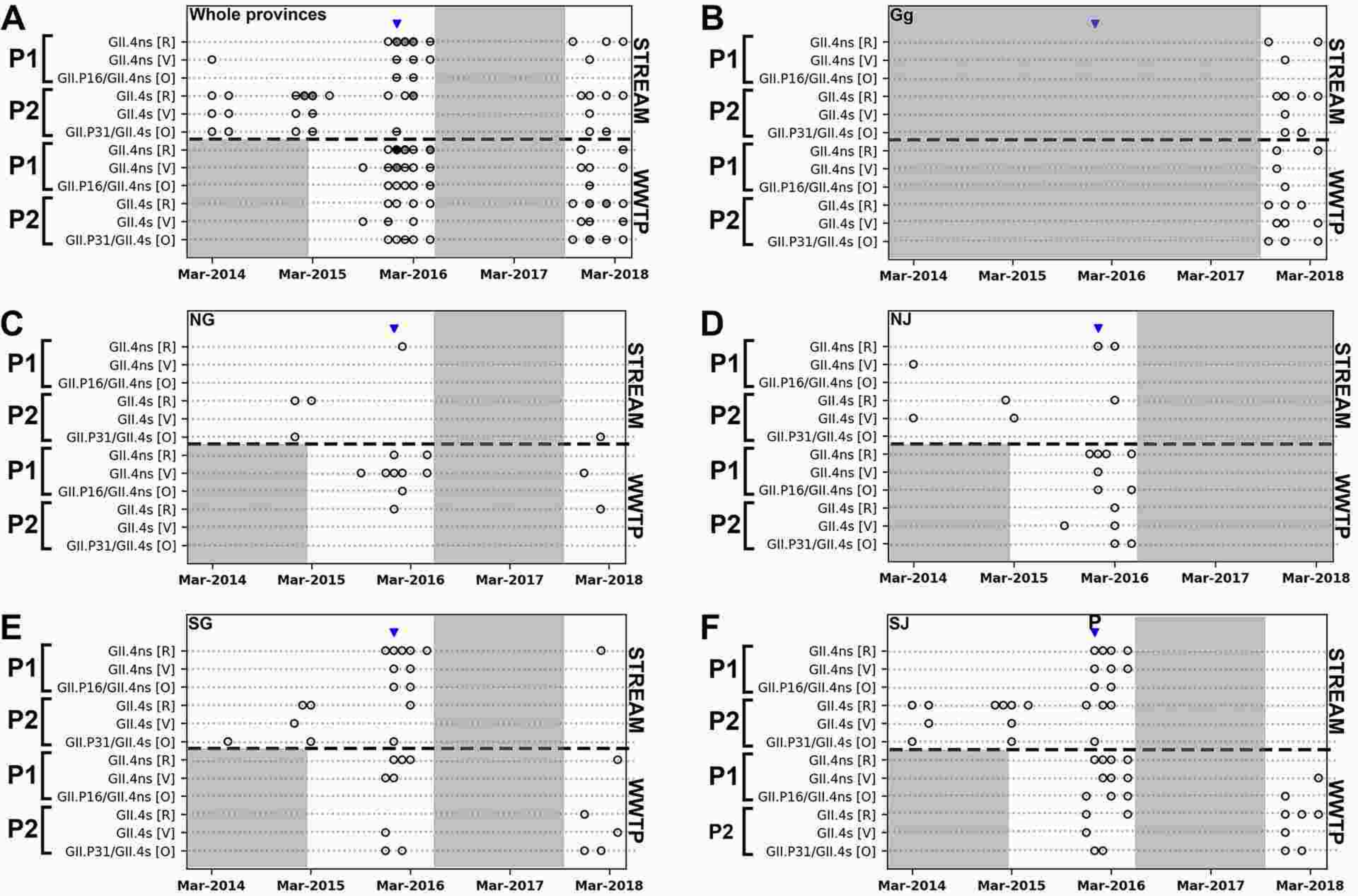

Page 86 (GII.4R_LPV)

Fig S10

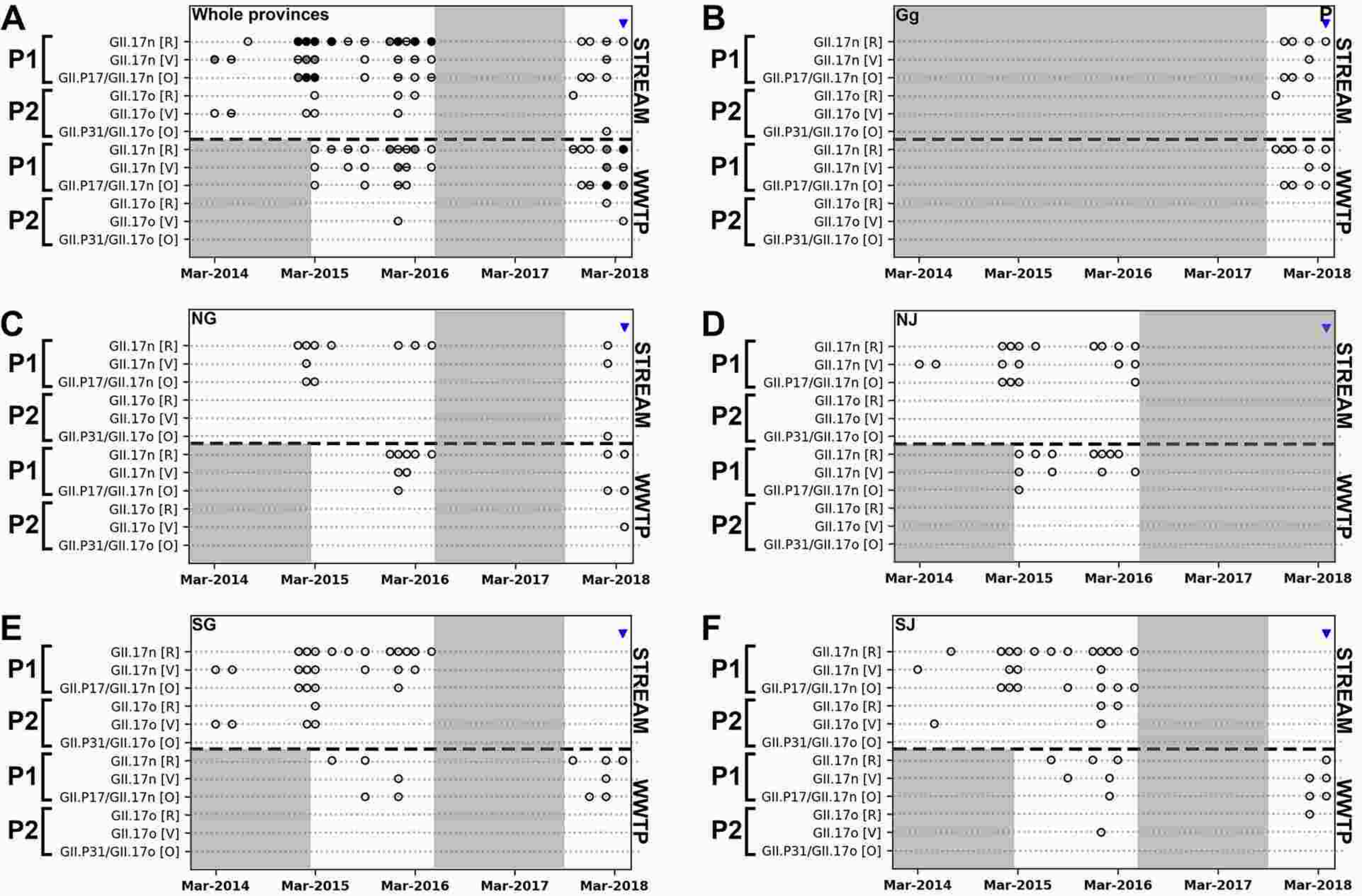

Page 87 (GII.17R_LPV)

Fig S10

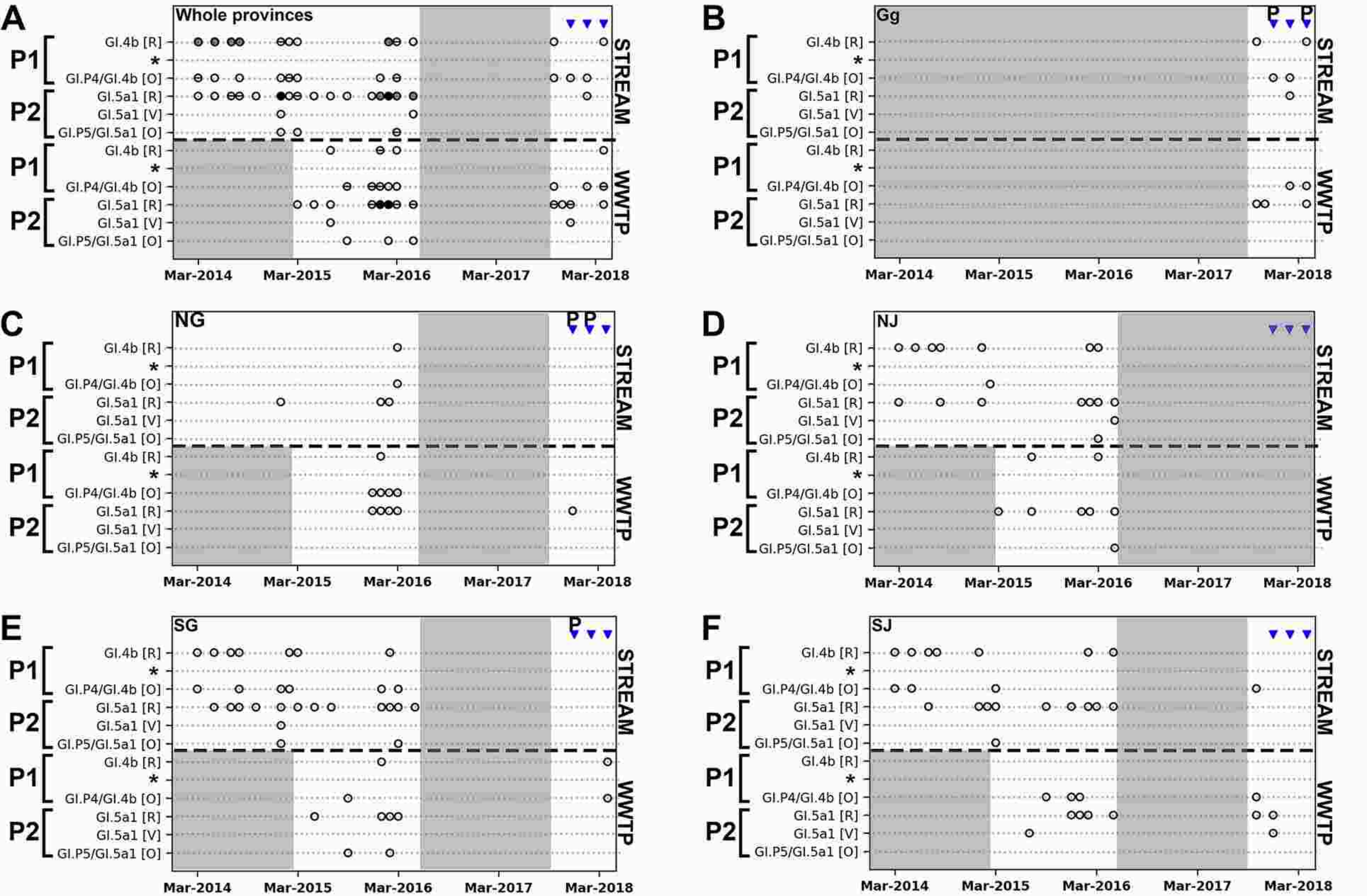

Page 88 (GI.P4/GI.5a2)

Fig S10

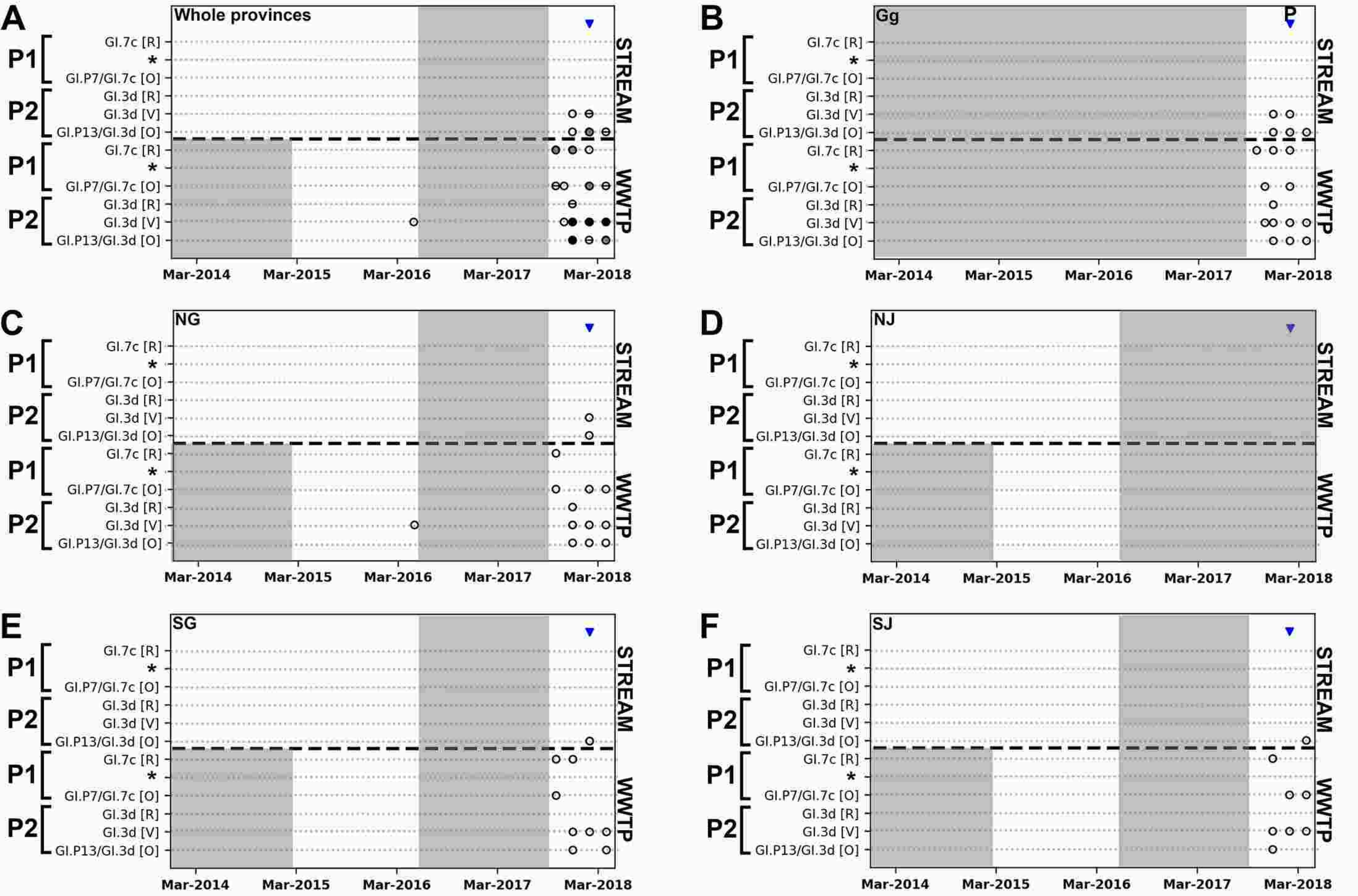

Page 89 (GI.P7/GI.3d)

Fig S10

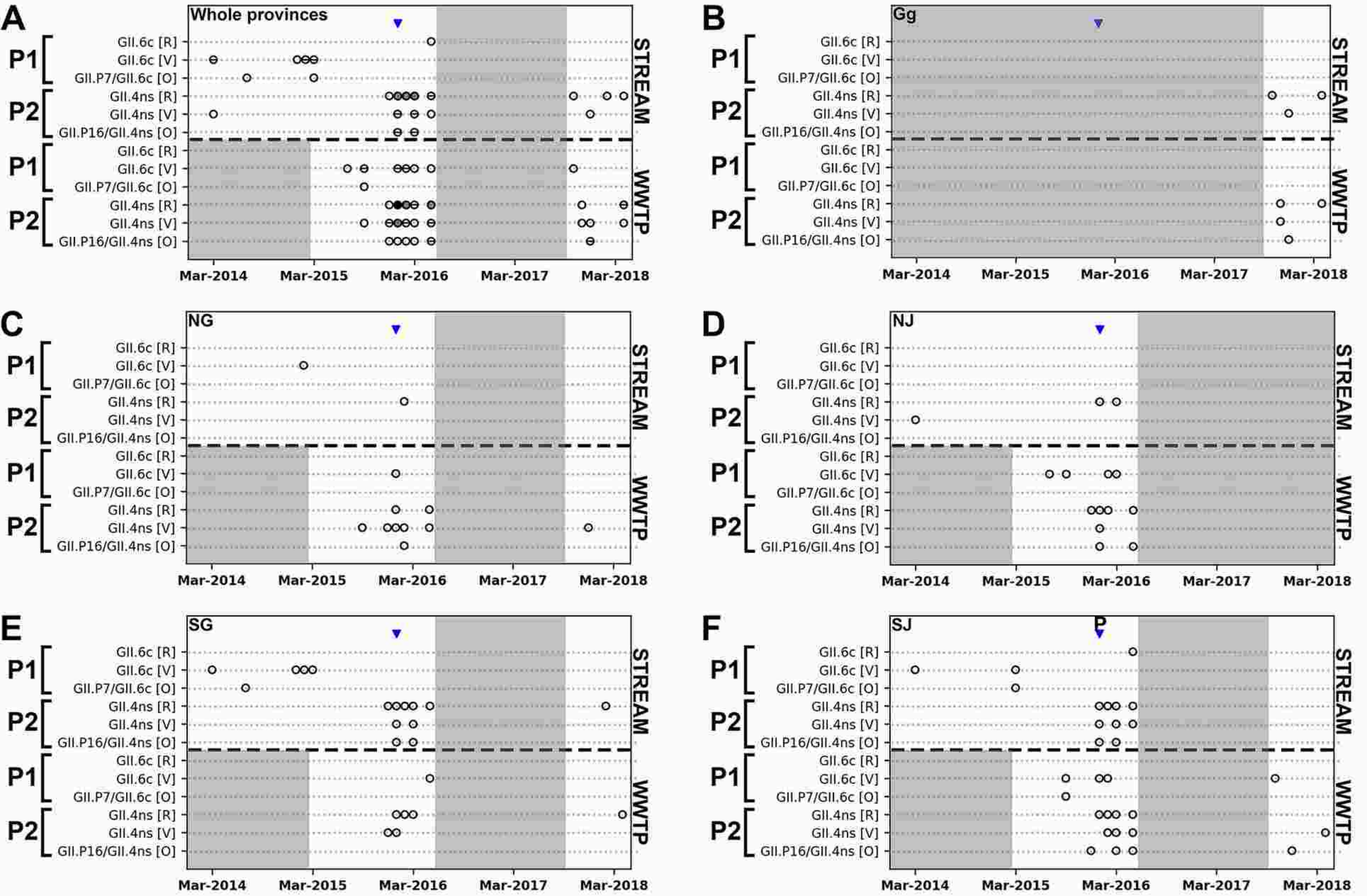

Page 90 (GII.P7/GII.6-4R) Fig S10

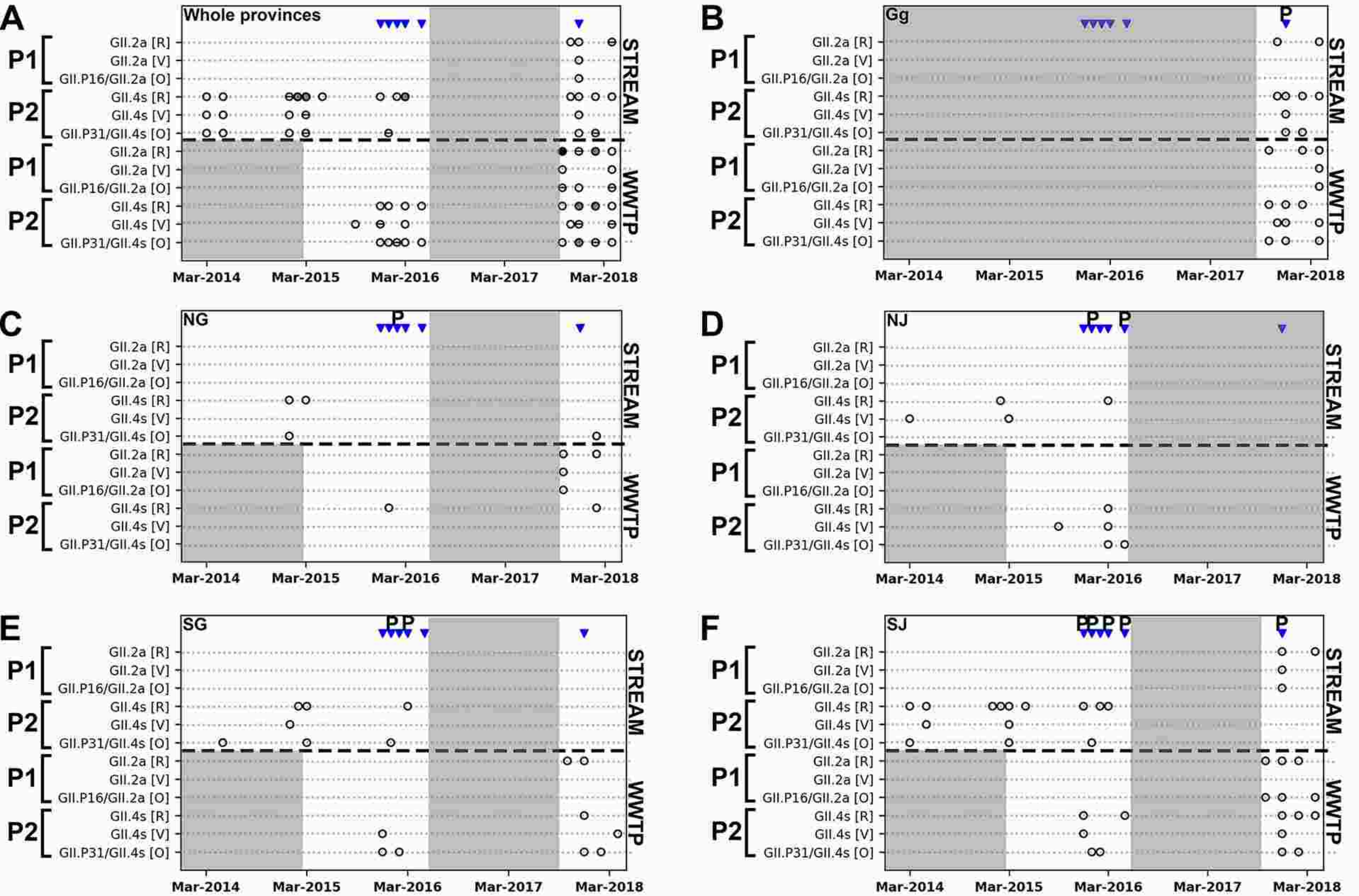

Page 91 (GII.P16/GII.4ns)

Fig S10

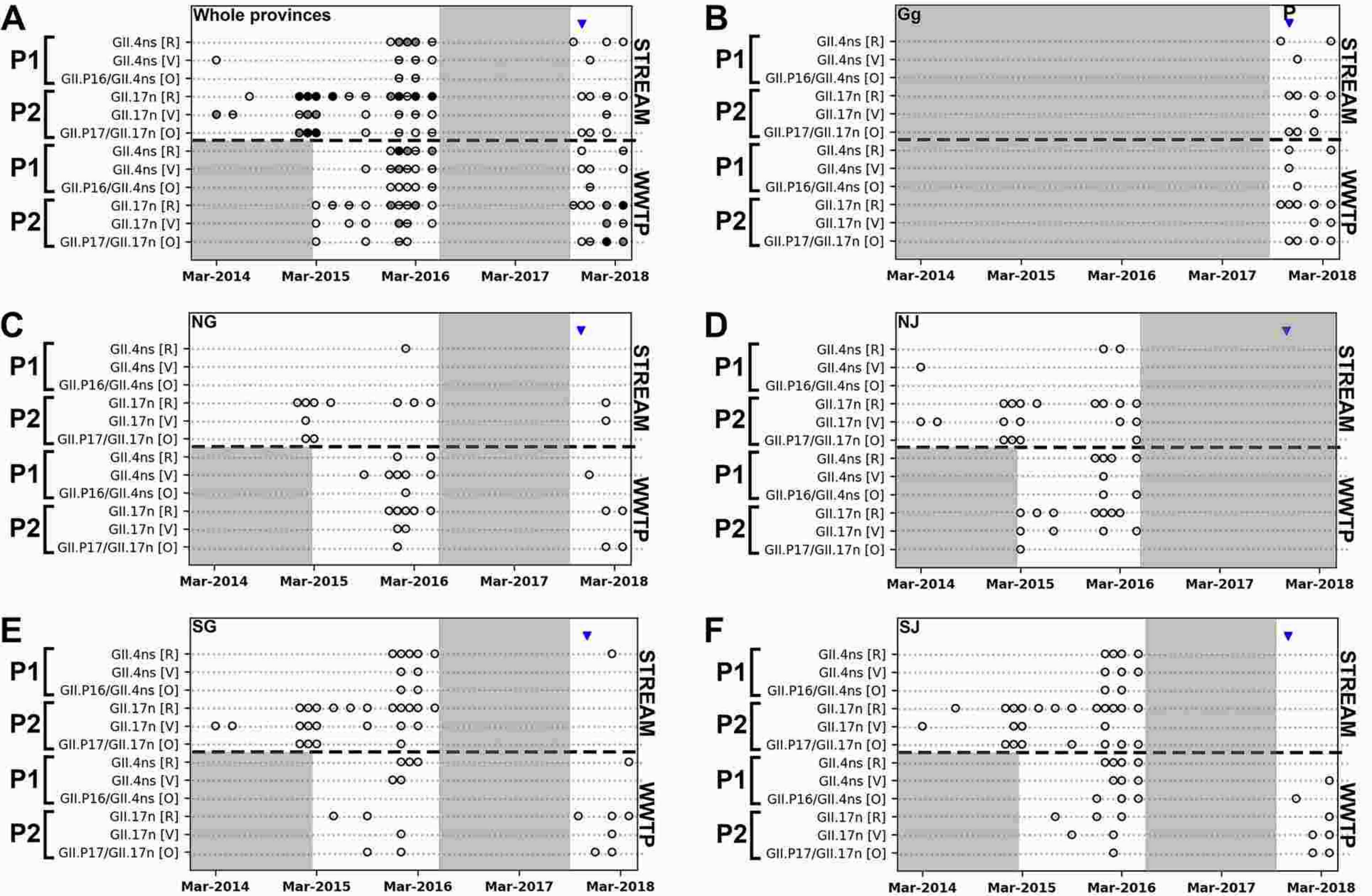

Page 92 (GII.P16-P17/GII.17nR)

Fig S10

FIG S10 Timetable plots of the occurrence of parents of each recombinant lineage of the norovirus genogroups GI or GII, based on three amplicon types in the waters of the provinces studied. **(A)–(F)** In each page, timetables describing the occurrences of both a recombinant lineage and its two-genetic parental lineages (P1 and P2) in the waters of all provinces (panel A) and each province (panels **B–F**) are shown. Abbreviated names of the provinces, Gg, NG, NJ, SG, and SJ, are located on the timetables. The name of each recombinant is shown at the bottom of each page. Lineage occurrences are presented as per water type: Stream or WWTP (waste water treatment plant). WWTP is equivalent to treated sewage effluent plus raw sewage. A dashed horizontal line separates water types in each panel. Each tick on the y-axis indicates the amplicon type of the parental lineages that occurred. The letters in square brackets indicate an amplicon type of the parental nucleotide sequence: [R] = region C; [V] = VP1; [O] = ORF1/ORF2 junction. Asterisks (*) on the y-axis indicate the absence of an isolated amplicon type during the whole study period. Circle markers with patterns indicate each positive case in a sampling month, which are indicative of the number of positive provinces in the same sampling month (white, one; horizontal line, two; grey, three; and four or more, black). An occurrence of a recombinant lineage (▼) in each province is indicated by "P". A grey box indicates a no-sampling period in each study province.

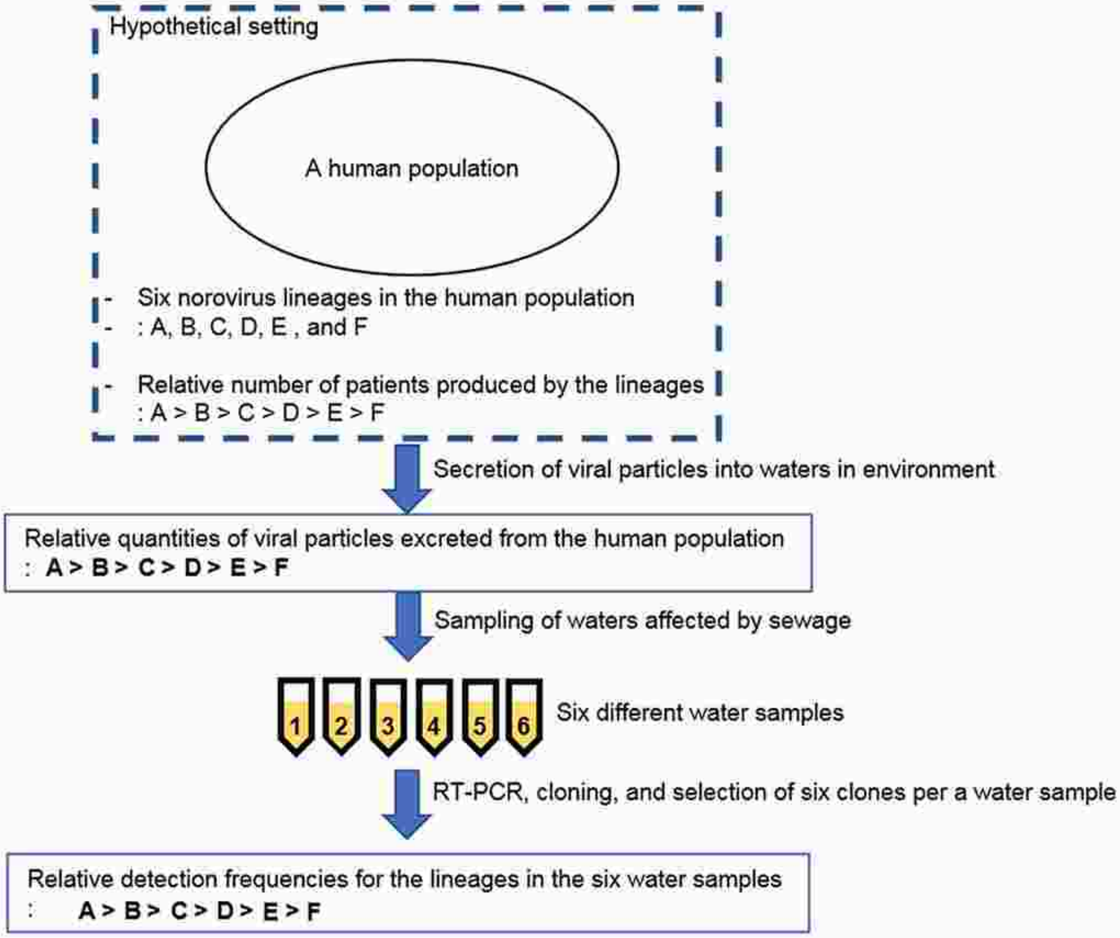

[Example data]

Detection result						
Samples	Lineages					
	A	B	C	D	E	F
1	+	+	+	N	N	N
2	+	+	N	N	+	N
3	+	+	+	+	N	+
4	+	+	+	+	N	N
5	+	+	N	+	N	N
6	+	N	+	N	+	N

+, positive; N, negative

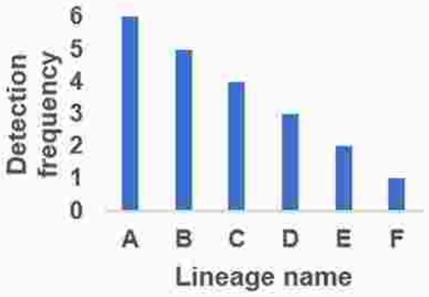

Number of co-isolated cases in identical samples

	A	B	C	D	E	F
F	1	1	1	1	0	
E	2	1	1	0		0
D	3	3	2		0	1
C	4	3		2	1	1
B	5		3	3	1	1
A		5	4	3	2	1

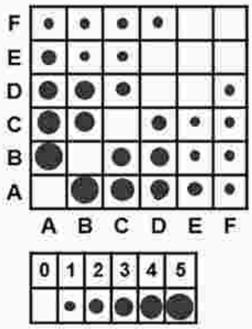

Fig S11

FIG S11 Conceptual diagrams for bubble charts displayed in Figs. 4–6.

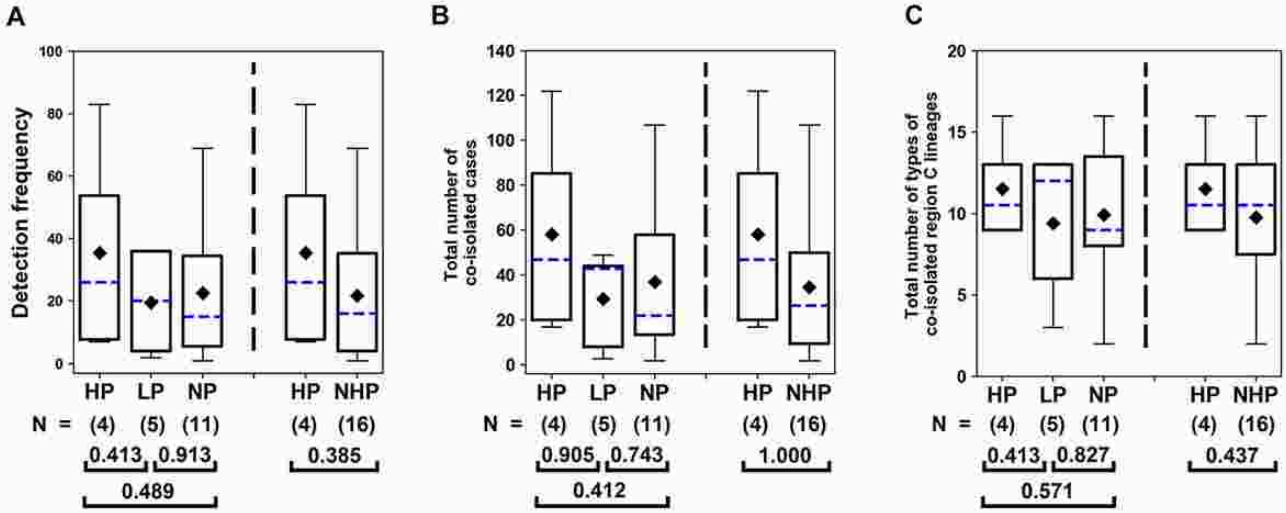
A
Detection frequency
0
20
40
60
80
100
HP
LP
NP
HP
NHP
N =
(4)
(5)
(11)
(4)
(16)
0.413
0.913
0.385
0.489
B
Total number of co-isolated cases
0
20
40
60
80
100
120
140
HP
LP
NP
HP
NHP
N =
(4)
(5)
(11)
(4)
(16)
0.905
0.743
1.000
0.412
C
Total number of types of co-isolated region C lineages
0
5
10
15
20
HP
LP
NP
HP
NHP
N =
(4)
(5)
(11)
(4)
(16)
0.413
0.827
0.437
0.571

Fig S12

FIG S12 Box plots for Fig. 5. **(A)**–**(C)** Box plots for comparing detection frequencies, the total number of co-isolated cases, and the total number of the types of co-isolated lineages in identical samples against the four groups, HP, LP, NP, and NHP. The vertical dashed-line separates compared groups in each box plot. Sample size (N) of each group is indicated below the group names. Both mean (◆) and median (--) are shown in each box. Numbers indicated on horizontal brackets describe P-values in the differences of mean ranks between the two compared groups (Mann-Whitney U test).

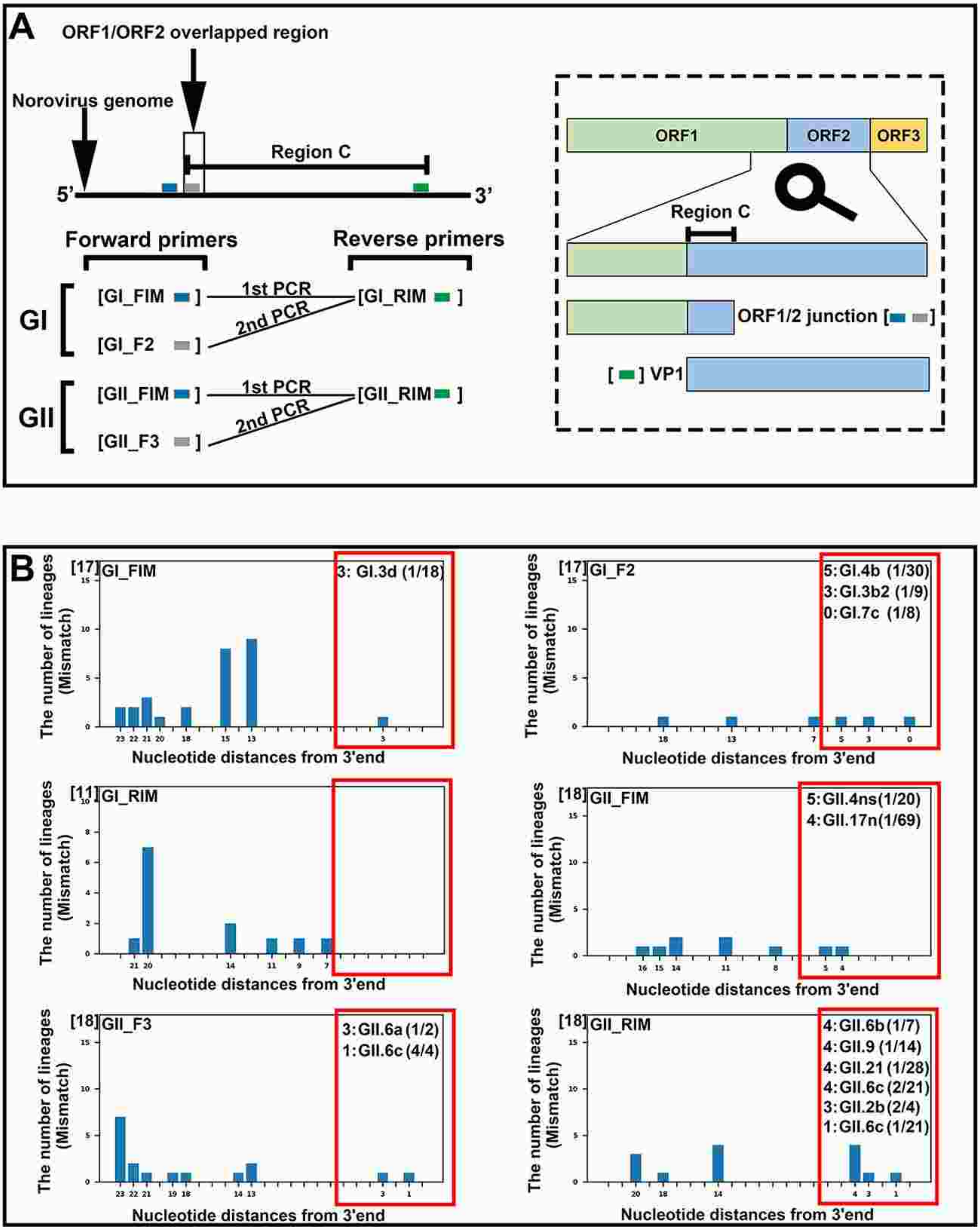
A
ORF1/ORF2 overlapped region
Norovirus genome
Region C
5'
3'
Forward primers
Reverse primers
GI
[GI_FIM]
[GI_F2]
1st PCR
2nd PCR
[GI_RIM]
GII
[GII_FIM]
[GII_F3]
[GII_RIM]
ORF1
ORF2
ORF3
Region C
ORF1/2 junction [ ]
[ ] VP1
B
[17] GI_FIM
3: GI.3d (1/18)
[17] GI_F2
5:GI.4b (1/30)
3:GI.3b2 (1/9)
0:GI.7c (1/8)
[11] GI_RIM
[18] GII_FIM
5:GII.4ns(1/20)
4:GII.17n(1/69)
[18] GII_F3
3:GII.6a (1/2)
1:GII.6c (4/4)
[18] GII_RIM
4:GII.6b (1/7)
4:GII.9 (1/14)
4:GII.21 (1/28)
4:GII.6c (2/21)
3:GII.2b (2/4)
1:GII.6c (1/21)
The number of lineages (Mismatch)
Nucleotide distances from 3'end

Fig S13

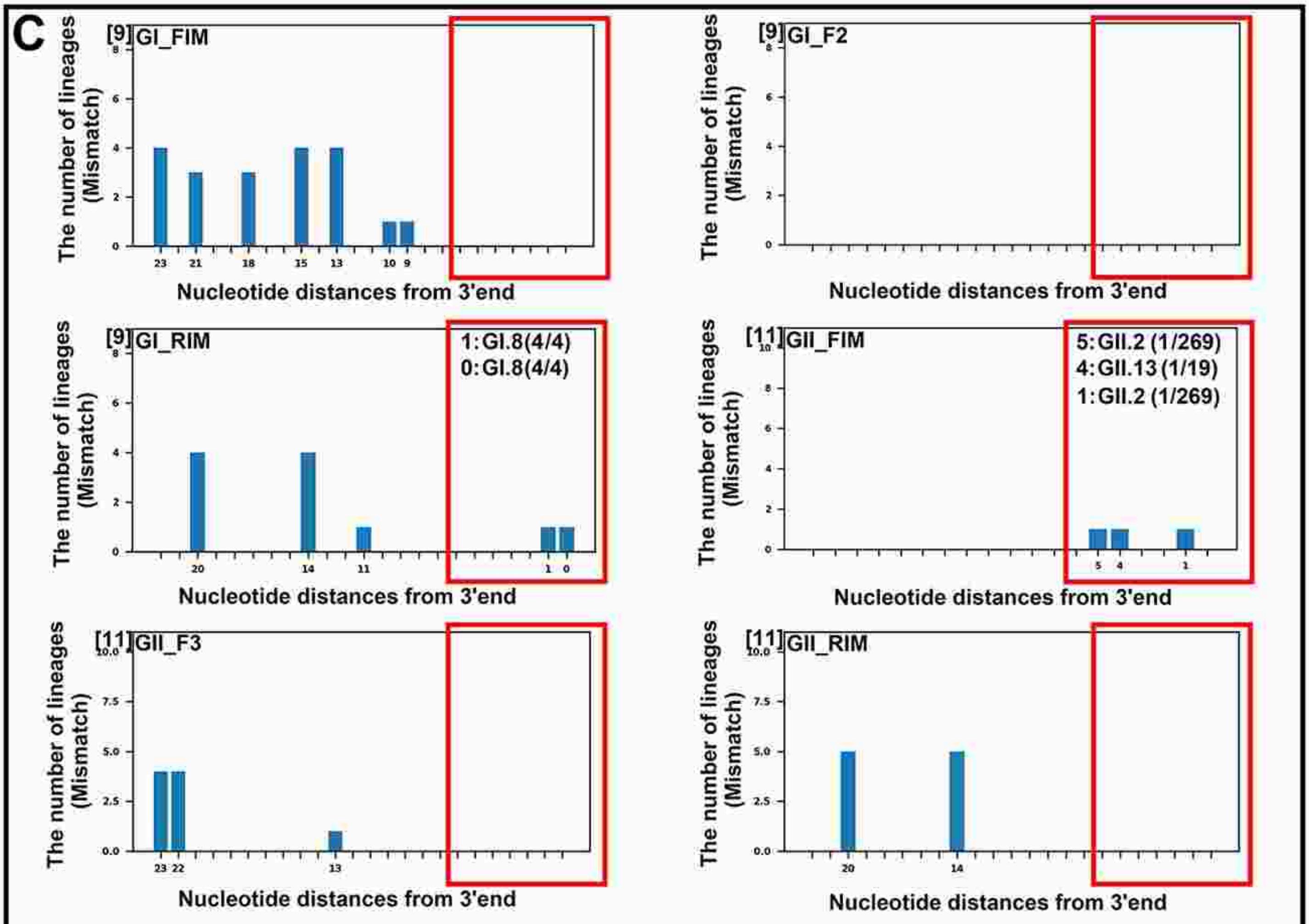

The number of sequences of GI (n=126): GI.1(n=26), GI.2(n=9), GI.3(n=42), GI.4(n=8), GI.5(n=6), GI.6(n=22), GI.7(n=6), GI.8(n=4), and GI.9(n=3)

The number of sequences of GII (n=895): GII.1(n=12), GII.2(n=269), GII.3(n=104), GII.4(n=252), GII.5(n=3), GII.6(n=45), GII.12(n=10), GII.13(n=19), GII.14(n=3), GII.17(n=173), and GII.21(n=5)

Fig S13

FIG S13 Mismatches between region C primers and their target sequences. **(A)** Target sites of the RT-PCR primers for region C amplicons in the norovirus genome of GI and GII, are indicated by colored markers. The primers are categorized by both the indicated polarities and their target genogroups. Primer pairs for PCR steps (1st PCR or 2nd PCR) are indicated by lines connecting the primer names. A dashed-line box describes the coverages of two amplicon types (VP1 and ORF1/2 junction), used to investigate mismatches with the primers (forward primers, ORF1/2 junction; reverse primers, VP1). **(B)** Vertical bar plots show mismatched positions of primers against the target sequences in isolated sequences of the two amplicon types. In the plot for each primer, the x-axis indicates mismatched positions based on nucleotide distances (descending order) estimated using position zero (0) of a base at the 3' end of the primer; y-axis indicates the number of lineage types in a genogroup showing mismatches in certain positions. A number in each square bracket at the y-axis indicates the number of investigated lineages in the corresponding genogroup. Red boxes highlight 7 nucleotide positions (0-6) assumed to be closely associated with the elongation efficiency of DNA polymerase. In the red boxes, mismatched positions, the causative lineage of mismatched positions, and the number of sequences mismatched per whole sequences are indicated, respectively. (**C)** Mismatched positions of the primers against the target sequences in the GenBank sequences are shown as vertical bar plots. The number of genotype sequences investigated in each genogroup is shown at the bottom of the panels.

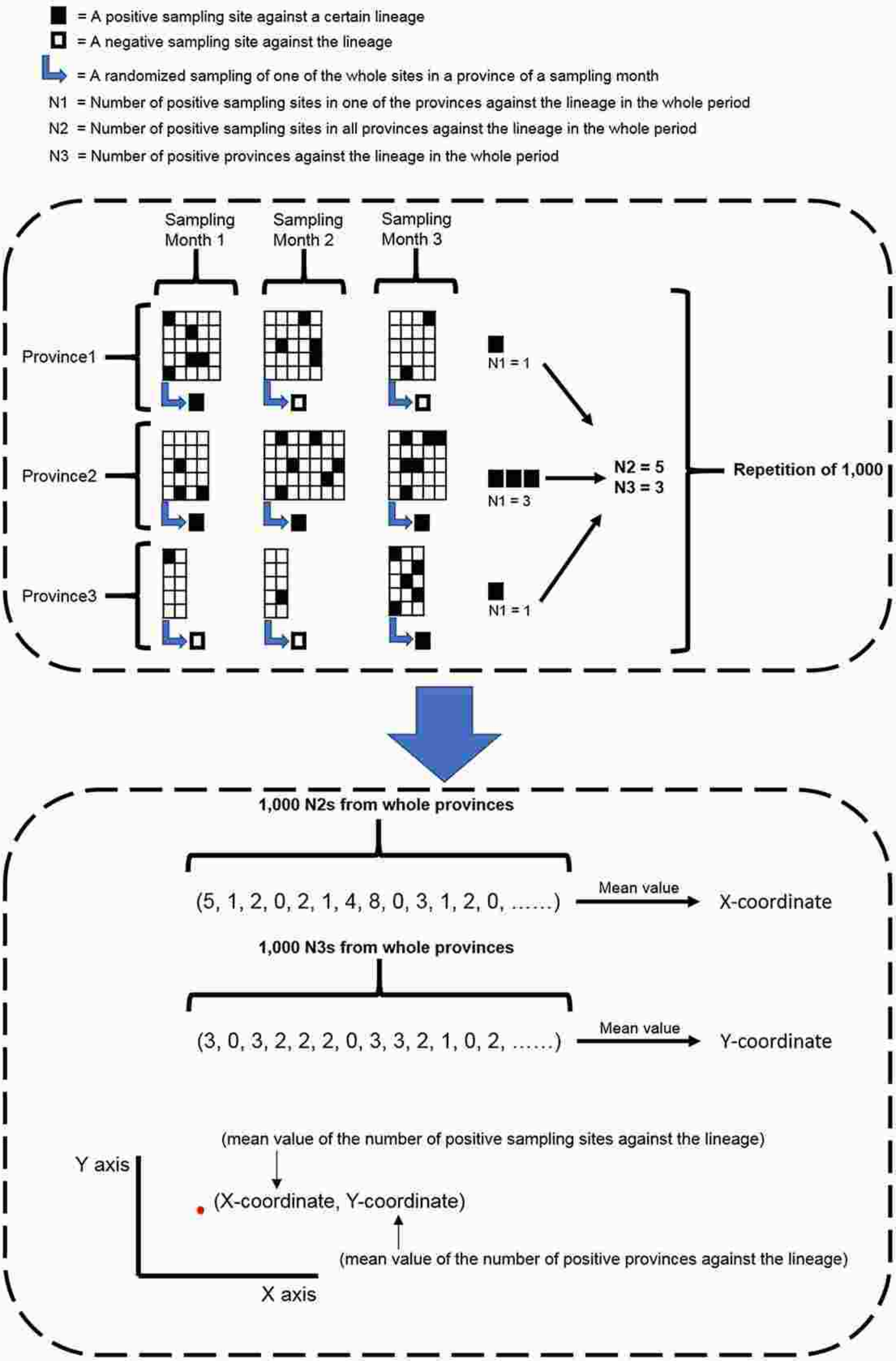
= A positive sampling site against a certain lineage
= A negative sampling site against the lineage
= A randomized sampling of one of the whole sites in a province of a sampling month
N1 = Number of positive sampling sites in one of the provinces against the lineage in the whole period
N2 = Number of positive sampling sites in all provinces against the lineage in the whole period
N3 = Number of positive provinces against the lineage in the whole period
Sampling Month 1
Sampling Month 2
Sampling Month 3
Province1
Province2
Province3
N1 = 1
N1 = 3
N1 = 1
N2 = 5
N3 = 3
Repetition of 1,000
1,000 N2s from whole provinces
(5, 1, 2, 0, 2, 1, 4, 8, 0, 3, 1, 2, 0, ...)
Mean value
X-coordinate
1,000 N3s from whole provinces
(3, 0, 3, 2, 2, 2, 0, 3, 3, 2, 1, 0, 2, ...)
Mean value
Y-coordinate
(mean value of the number of positive sampling sites against the lineage)
Y axis
(X-coordinate, Y-coordinate)
(mean value of the number of positive provinces against the lineage)
X axis

Fig S14

FIG S14 Conceptual diagrams of randomized sampling to normalize geographical/seasonal imbalances in the number of available sampling sites.

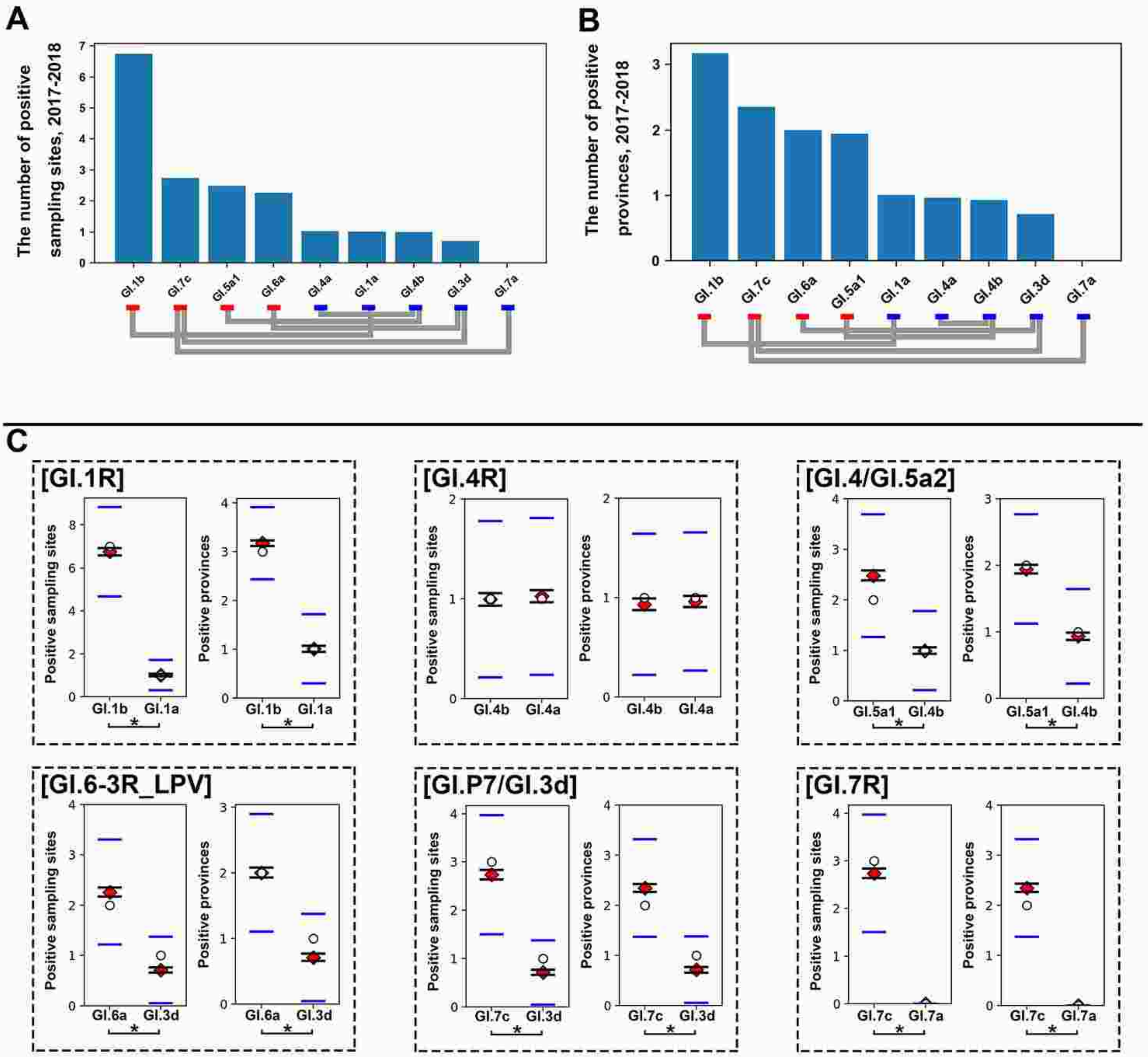
A
The number of positive sampling sites, 2017-2018
GI.1b
GI.7c
GI.5a1
GI.6a
GI.4a
GI.1a
GI.4b
GI.3d
GI.7a
B
The number of positive provinces, 2017-2018
C
[GI.1R]
[GI.4R]
[GI.4/GI.5a2]
[GI.6-3R_LPV]
[GI.P7/GI.3d]
[GI.7R]
Positive sampling sites
Positive provinces

Fig S15

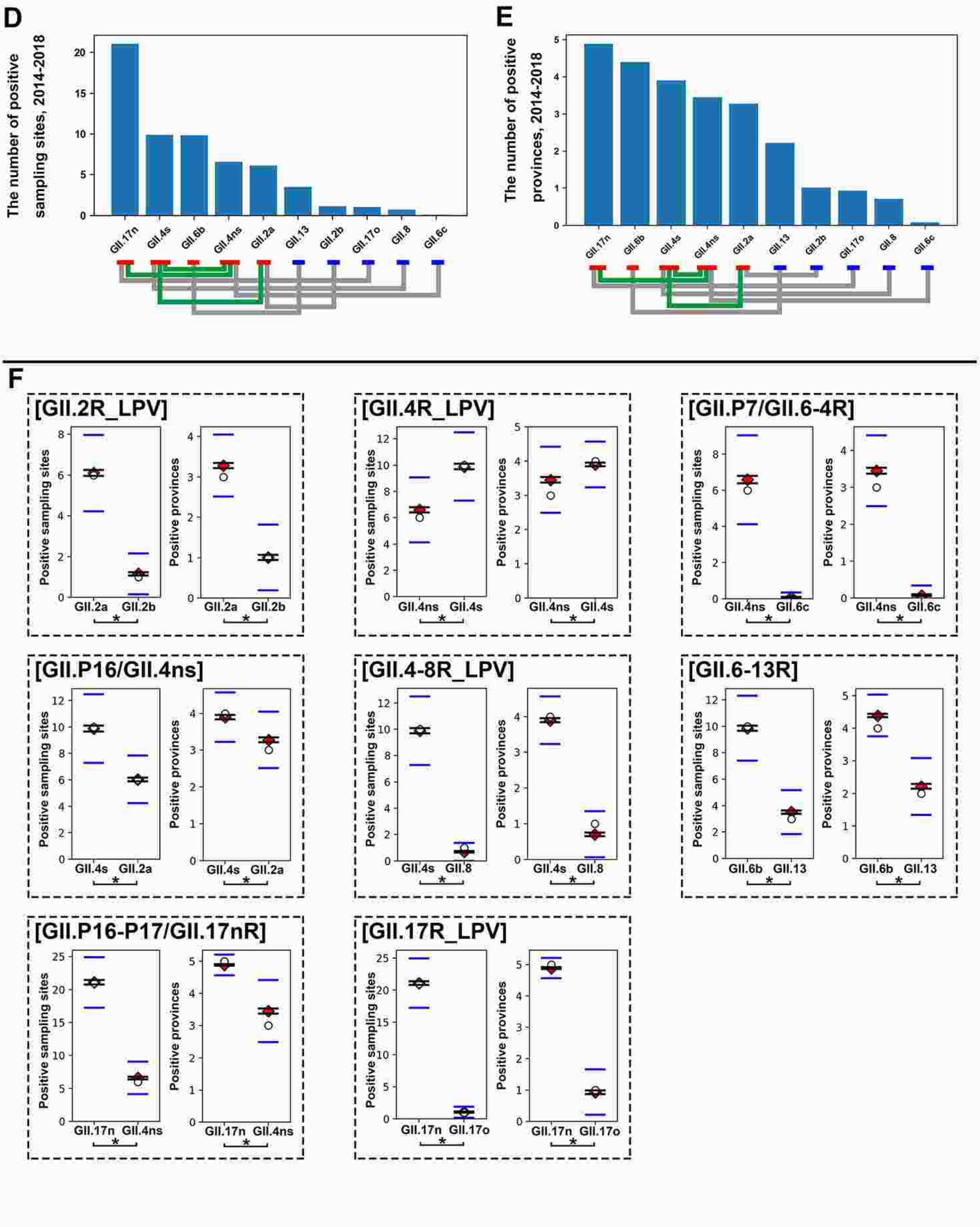
D
The number of positive sampling sites, 2014-2018
GII.17n
GII.4s
GII.6b
GII.4ns
GII.2a
GII.13
GII.2b
GII.17o
GII.8
GII.6c
E
The number of positive provinces, 2014-2018
GII.17n
GII.6b
GII.4s
GII.4ns
GII.2a
GII.13
GII.2b
GII.17o
GII.8
GII.6c
F
[GII.2R_LPV]
[GII.4R_LPV]
[GII.P7/GII.6-4R]
[GII.P16/GII.4ns]
[GII.4-8R_LPV]
[GII.6-13R]
[GII.P16-P17/GII.17nR]
[GII.17R_LPV]
Positive sampling sites
Positive provinces
GII.2a
GII.2b
GII.4ns
GII.4s
GII.6c
GII.8
GII.6b
GII.13
GII.17n
GII.17o
*

Fig S15

FIG S15 Confirmation of HP and LP categorization using mean values of randomly sampled data from both the number of positive sampling sites and the number of positive provinces. **(A)** A vertical bar plot describing the number of positive sampling sites (mean values of randomly sampled data) of the parents of recombinants from genogroup I. Colored markers below lineage names indicate re-categorized parental groups (red = HP; blue = LP). A colored polygonal line connects parental pairs of each recombinant lineage in the same manner as that in Fig. 6A. **(B)** A vertical bar plot describing the number of positive provinces (mean values of randomly sampled data) of the parents of recombinants from genogroup I. **(C)** Vertical scatter plots for comparison of mean values (◆) and median values (○) of randomly sampled data (N = 1,000 per a lineage) of two different parents of each GI recombinant. Error bars (blue, standard deviation of each mean; black, 99% confidence interval of each mean) are shown for each compared dataset. Asterisks (*) below the names of parents describe the statistical significance ($P < 0.001$) in the differences of mean ranks between the compared groups (Mann-Whitney U test). **(D)–(F)** Both vertical bar plots and vertical scatter plots of GII data are shown in the same manner as those in panels A–C.

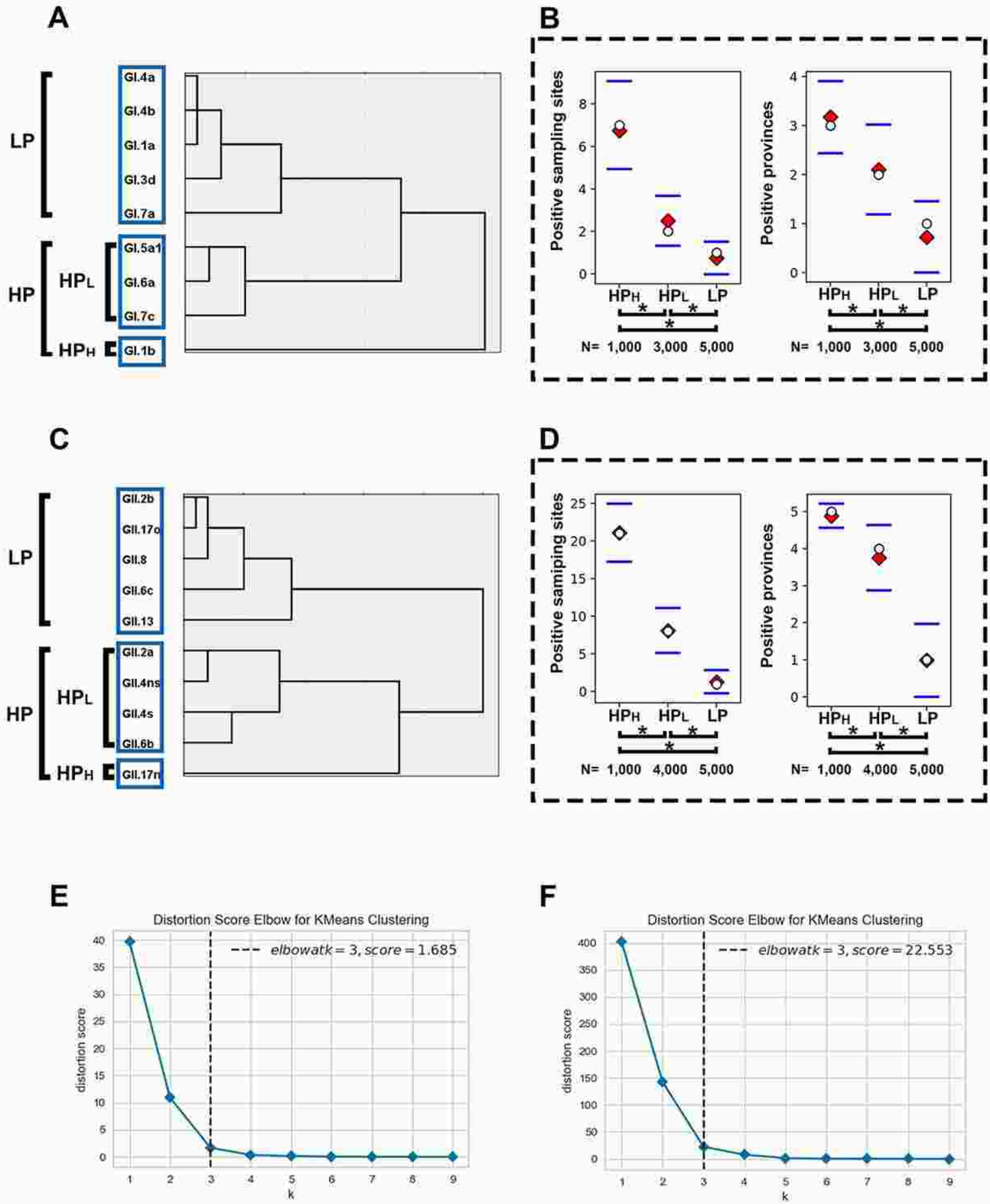
A
LP
HP
HPL
HPH
GI.4a
GI.4b
GI.1a
GI.3d
GI.7a
GI.5a1
GI.6a
GI.7c
GI.1b
B
Positive sampling sites
Positive provinces
HPH
HPL
LP
N= 1,000 3,000 5,000
C
LP
HP
HPL
HPH
GII.2b
GII.17o
GII.8
GII.6c
GII.13
GII.2a
GII.4ns
GII.4s
GII.6b
GII.17n
D
Positive samping sites
Positive provinces
HPH
HPL
LP
N= 1,000 4,000 5,000
E
Distortion Score Elbow for KMeans Clustering
elbowatk = 3, score = 1.685
distortion score
k
F
Distortion Score Elbow for KMeans Clustering
elbowatk = 3, score = 22.553
distortion score
k

Fig S16

FIG S16 Combination of hierarchical and K-means clustering identified additional groups of HP parents in the two norovirus genotypes, GI and GII. **(A)** A dendrogram of the hierarchical clustering of the mean values of GI parents in Fig. 7A. Blue boxes surrounding the names of the parents indicate groups defined by K-means clustering. The names of the groups of parents with square brackets indicate parental groups defined by the combination of two clustering algorithms. **(B)** Vertical scatter plots for comparison of values from the random sampling of two datasets (the number of the positive sampling sites; the number of the positive provinces) of the three groups, HP_H, HP_L and LP, of GI. Mean values (◆) and median values (○), and error bars (blue; standard deviation of each mean) are shown for each compared dataset. Asterisks (*) below the names of parental lineages describe the statistical significance ($P < 0.001$) in the differences of mean ranks between the compared groups (Mann-Whitney U test). The number of randomly sampled values (N) in each parental group is shown below the plots. **(C), (D)** Both the dendrogram and the vertical scatter plots for GII parents in Fig. 7B are shown in the same manner as that of the panels A and B. **(E), (F)** Line plots for defining optimal numbers of group partition in the K-means clustering of mean values of GI (panel E) and GII (panel F) (The Elbow method). Both plots are presented as raw figures generated with Python module, yellowbrick. In each plot, the x-axis provides possible partitions (K) generated from K-means clustering; y-axis indicates the total sum of squared within-distances from a centre in each partition (distortion score) of every K. Vertical dashed-lines on the x-axis in each plot indicate best-fitted numbers of K (optimal numbers of subgroups).

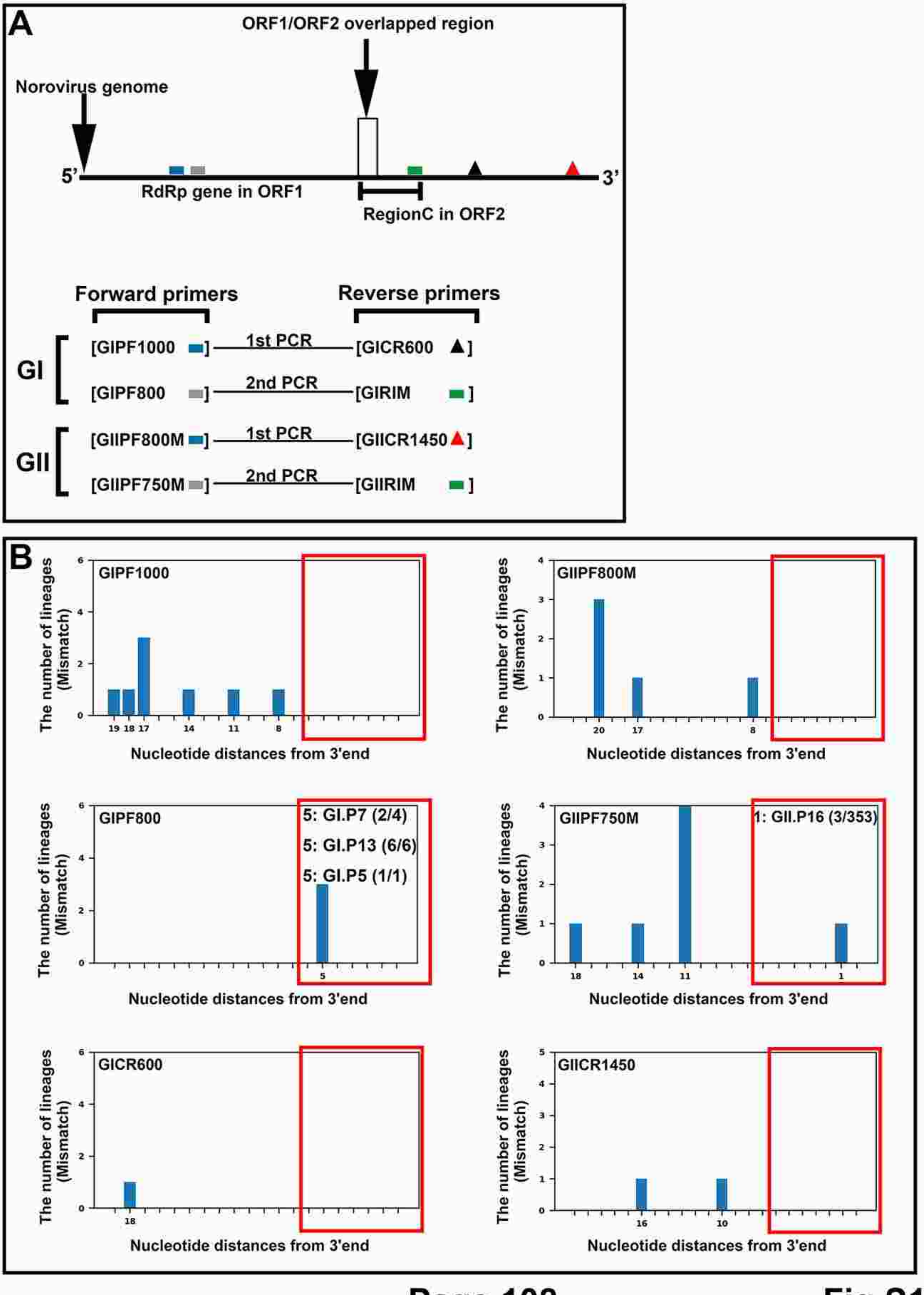
A
ORF1/ORF2 overlapped region
Norovirus genome
5'
3'
RdRp gene in ORF1
RegionC in ORF2
Forward primers
Reverse primers
GI
[GIPF1000]
1st PCR
[GICR600]
[GIPF800]
2nd PCR
[GIRIM]
GII
[GIIPF800M]
1st PCR
[GIICR1450]
[GIIPF750M]
2nd PCR
[GIIRIM]
B
GIPF1000
GIIPF800M
GIPF800
5: GI.P7 (2/4)
5: GI.P13 (6/6)
5: GI.P5 (1/1)
GIIPF750M
1: GII.P16 (3/353)
GICR600
GIICR1450
The number of lineages (Mismatch)
Nucleotide distances from 3'end

Fig S17

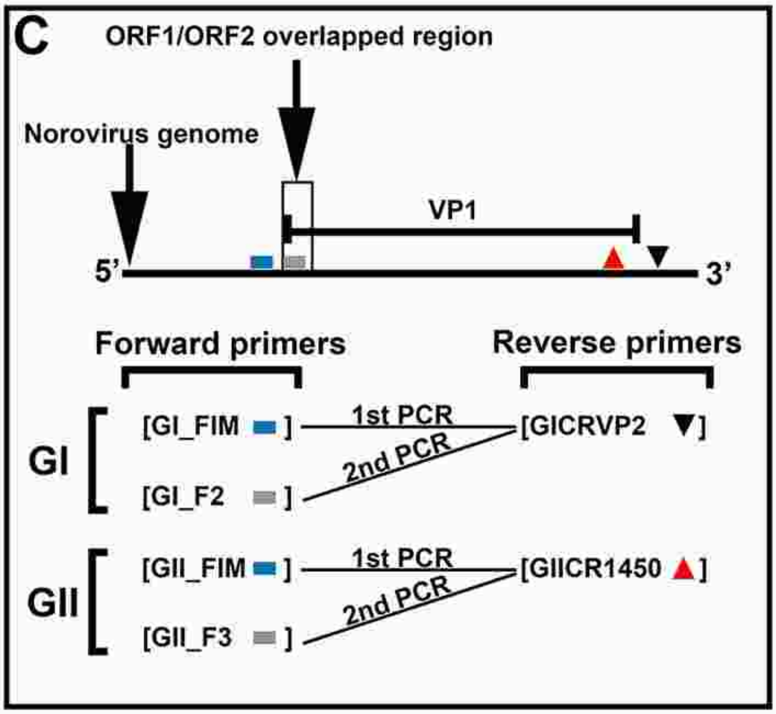

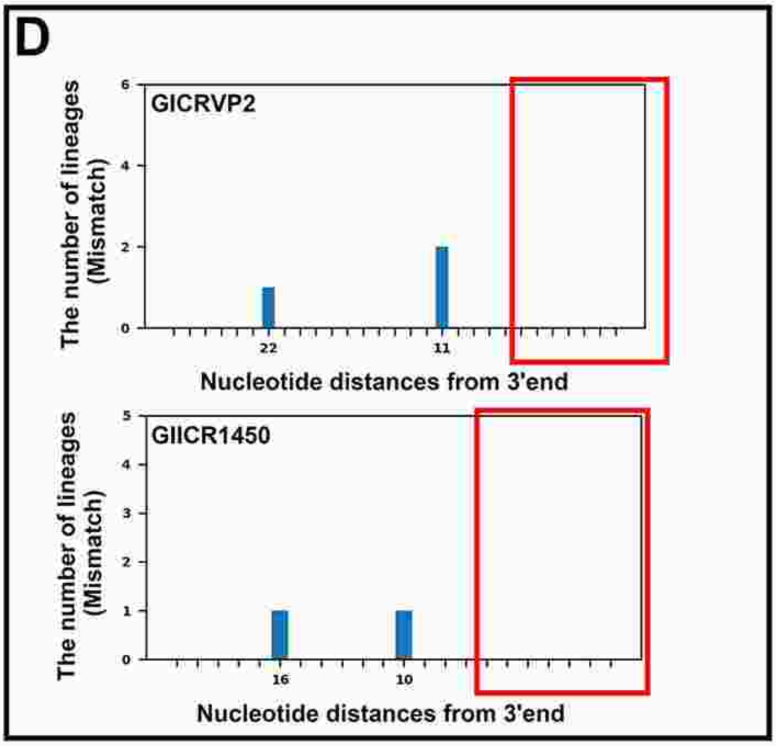

[The number of investigated nuclotide sequences of the GenBank]

GI ORF1 (n=41) : P1 (n=17), P4 (n=4), P5 (n=1), P7 (n=4), P11 (n=9), and P13 (n= 6)

GI ORF2 (n=94) : GI.1 (n=24), GI.3 (n=31), GI.4 (n=8), GI.5 (n=4), GI.6 (n=21), and GI.7 (n=6)

GII ORF1 (n=653) : P7 (n=47), P16 (n=353), P17 (n=157), and P31 (n=96)

GII ORF2 (n=757) : GII.2 (n=269), GII.4 (n=253), GII.6 (n=45), GII.13 (n=19), and GII.17 (n=171)

Fig S17

FIG S17 Mismatches between RT-PCR primers for two amplicon types (both ORF1/2 junction and VP1) and their target sequences on the parental sequences. **(A), (C)** Target sites of the RT-PCR primers on the parental sequences of the norovirus genome for two types of amplicons (the ORF1/2 junction, panel **A**; the VP1, panel **C**) from GI and GII are indicated by colored markers. The primers are categorized by both polarities and their target genogroups. The lines connecting primer names indicate primer pairs for PCR steps (1st PCR or 2nd PCR). **(B), (D)** Mismatched positions of primers against the parents (Table 1) in the GenBank sequences are shown as vertical bar plots. Fig. S13 shows vertical bar plots for region C primers not shown in this figure. In the plot for each primer, the x-axis indicates mismatched positions as nucleotide distances (descending order) based on position zero (0) of a base located at the 3' end of the primer; y-axis indicates the number lineage types in a genogroup showing mismatches at certain positions. Red boxes highlight 7 nucleotide positions (0-6) that are assumed to be more closely related to the elongation efficiency of DNA polymerase. In the red boxes, mismatched positions, the causative lineage of mismatched positions, and the number of sequences mismatched per whole sequences are indicated, respectively.

TABLE S1 Group mean distances of newly defined sub-genotypes of isolated region C sequences of both norovirus genogroup I and genogroup II

Genotype of common region C (0.3 kb)	Names of sub-genotypes	Within-group mean p-distance of each sub-genotype[a]	Between group mean p-distance of sub-genotypes in each genotype[b]	P-value[c]
GI.1	GI.1a	0.009	0.096	<0.001
	GI.1b	0.013		
GI.3	GI.3b1	0.010	0.083	<0.001
	GI.3b2	0.013		
GI.4	GI.4a	0.012	0.052	<0.001
	GI.4b	0.010		
GI.5	GI.5a1	0.017	0.085	<0.001
	GI.5a2	0.006		
GII.2	GII.2a	0.013	0.037 (GII.2a and GII.2b)[d], 0.048 (GII.2a and GII.2c)[d], and 0.053 (GII.2b and GII.2c)[d]	<0.001
	GII.2b	0.015		
	GII.2c	0.018		

[a] mean value of total distances between the sequences in a sub-lineage of a genotype

[b] mean value of total distances between the sequences of two different sub-genotypic lineages in a genotype

[c] P-value of the Mann-Whitney U test for comparing “within-group distances of sub-genotypes” with “between group distance of sub-genotypes” in a genotype

[d] between group mean distance of two indicated lineages

TABLE S2 P-values for groups in region C amplicon lineages of GII obtained via the Mann-Whitney U test

Variable	Group 1	Comparison target of group 1		
		HP	LP	NHP
DF	NP	0.005*[a]	0.913[a]	
	HP		0.016*[a]	0.002*
TC	NP	0.009*[a]	0.827[a]	
	HP		0.016*[a]	0.003*
TL	NP	0.009*[a]	0.583[a]	
	HP		0.056[a]	0.006*

DF, Detection frequency

TC, The total number of co-isolated cases in identical samples

TL, Total number of the types of co-isolated region C lineages in identical samples

* P-value lower than 0.05

[a] Significance level required to reject H0 is P < 0.017 via the Bonferroni correction

TABLE S3 P-values for groups in region C amplicon lineages of GI obtained via the Mann-Whitney U test

Variable	Group 1	Comparison target of group 1		
		HP	LP	NHP
DF	NP	0.078[a]	1.000[a]	
	HP		0.016*[a]	0.027*
TC	NP	0.056[a]	1.000[a]	
	HP		0.016*[a]	0.020*
TL	NP	0.040*[a]	0.827[a]	
	HP		0.016*[a]	0.014*

DF, Detection frequency

TC, Total number of co-isolated cases in identical samples

TL, The total number of the types of co-isolated region C lineages in identical samples

* P-value lower than 0.05

[a] significance level required to reject H0 is P < 0.017, via the Bonferroni correction

TABLE S4 Results of K-means clustering analyses

The number of specified clusters	Genogroup	Variable	Final cluster centres			F value	P value
			Cluster 1	Cluster 2	Cluster 3		
Two	I	Positive sampling sites	1.40	6.75	-	26.94	0.001
		Positive provinces	1.24	3.17	-	5.36	0.054
		Clustered lineages	1a, 3d, 4a, 4b, 5a1, 6a, 7a, and 7c	1b	-	-	-
	II	Positive sampling sites	4.33	21.09	-	16.54	0.004
		Positive provinces	2.22	4.89	-	2.55	0.149
		Clustered lineages	2a, 2b, 4a, 4ns, 6b, 6c, 8, 13, and 17o	17n	-	-	-
Three	I	Positive sampling sites	0.75	2.50	6.75	105.98	<0.001
		Positive provinces	0.72	2.10	3.17	25.72	0.001
		Clustered lineages	1a, 3d, 4a, 4b, and 7a	5a1, 6a, and 7c	1b	-	-
	II	Positive sampling sites	1.30	8.11	21.09	64.34	<0.001
		Positive provinces	0.99	3.75	4.89	25.97	0.001
		Clustered lineages	2b, 6c, 8, 13, and 17o	2a, 4s, 4ns, and 6b	17n	-	-

- Not Available

TABLE S5 Primers for the amplification of three amplicon types

Genetic locus	Genogroup	Primer pair	Purpose
ORF2 (Region C, 0.3 kb)	I	GI-F1M and GI-R1M	One-step RT-PCR (first PCR)
		GI-F2 and GI-R1M	Semi-nested PCR (secondary PCR)
	II	GII-F1M and GII-R1M	One-step RT-PCR (first PCR)
		GII-F3 and GII-R1M	Semi-nested PCR (secondary PCR)
ORF2 (1.5-1.6 kb)	I	GI-F1M and GICRVP2	One-step RT-PCR (first PCR)
		GI-F2 and GICRVP2	Semi-nested PCR (secondary PCR)
	II	GII-F1M and GIICR1450	One-step RT-PCR (first PCR)
		GII-F3 and GIICR1450	Semi-nested PCR (secondary PCR)
ORF1-ORF2 (1.0-1.2 kb)	I	GIPF1000 and GICR600	One-step RT-PCR (first PCR)
		GIPF800 and GI-RIM	Nested PCR (secondary PCR)
	II	GIIPF800M and GIICR1450	One-step RT-PCR (first PCR)
		GIIPF750M and GII-RIM	Nested PCR (secondary PCR)

TABLE S6 Nucleic acid amplification conditions

<table>
<tr><th rowspan="2">Amplification processes</th><th colspan="2">Amplicon types</th></tr>
<tr><th>Region C</th><th>Both VP1 and ORF1/2 junction</th></tr>
<tr><td rowspan="2">One-step RT-PCR (first PCR)</td><td>[Mixture condition]
RNA extract (5.0 µL)
1-Step PCR ReddyMix (25.0 µL, (Thermo Fisher Scientific, Waltham, MA, USA)
RT enhancer (2.5 µL)
20 µM forward primer (2.0 µL)
20 µM reverse primer (2.0 µL)
Verso Enzyme Mix (1.0 µL)
Deionised sterile water (12.5 µL)</td><td>[Mixture condition]
Same as the mixture condition for region C</td></tr>
<tr><td>[RT-PCR cycle]
1. 45°C for 30 min
2. 94°C for 5 min
3. 35 cycles of amplification (94°C for 30 s, 55°C for 30 s, and 72°C for 1 min 30 s)
4. 72°C for 7 min</td><td>[RT-PCR cycle]
1. 45°C for 30 min
2. 94°C for 5 min
3. 35 cycles of amplification (94°C for 1 min, 47°C for 1 min, and 72°C for 3 min)
4. 72°C for 5 min.</td></tr>
<tr><td rowspan="2">Secondary PCR</td><td>[Mixture condition]
Product of One step RT-PCR (5.0 µL)
Top DNA polymerase (1.0 µL Bioneer, Daejeon, South Korea)
10× buffer (5.0 µL)
2.5 mM dNTPs (4.0 µL)
22.5 µM forward primer (2.0 µL)
22.5 µM reverse primer (2.0 µL)
Deionised sterile water (31.0 µL)</td><td>[Mixture condition]
Same as the mixture condition for region C</td></tr>
<tr><td>[PCR cycle]
1. 94°C for 5 min
2. 25 cycles of amplification (94°C for 30 s, 55°C for 30 s, and 72°C for 1 min 30 s)
3. 72°C for 7 min</td><td>[PCR cycle]
1. 94°C for 5 min
2. 29 cycles of amplification (94°C for 1 min, 47°C for 1 min, and 72°C for 2 min 30 s)
3. 72°C for 5 min</td></tr>
</table>

Protocol

[Contents]

- **1. Flow charts for construction of standard recombinants (STRs)**
  1.1 Overview of selection of reference sequences
  - 1.1.1 Construction of neighbor joining trees and selection of GI sequence pairs showing minimum branch length in the trees
  - 1.1.2 Construction of neighbor joining trees and selection of GII sequence pairs showing minimum branch length in the trees

- 1.2 Construction of the standard recombinants (STRs)
  - 1.2.1 Construction of the STRs for RDP4 analysis of ORF2
  - 1.2.2 Construction of the STRs for RDP4 analysis of ORF1
  - 1.2.3 Construction of the STRs for RDP4 analysis of the ORF1/2 junction

- **2. RDP4 analysis using the STRs**
- 2.1 Estimation of recombination events in the isolated ORF2 sequences
- 2.2 Estimation of recombination events in the isolated ORF1 sequences
- 2.3 Estimation of recombination events in the isolated ORF1/2 junction sequences

- **3. Hypothetical examples of detection of recombinant candidate detections satisfying the number of positive signals at the STRs**

1. Flow charts for construction of standard recombinants (STRs)

1.1 Overview of selection of reference sequences

Phylogenetic reference sequences of two norovirus genogroups (J Vinjé, 2015) (2) possessing sufficient coverage compared to the isolated amplicon sequences in this study		
**Genogroup**	**ORF1 genotypes**	**Both ORF2 genotypes and ORF2 sub-genotypes**
**I**	**P1, P2, P4, P6, Pb, Pc, and Pf (N=7)**	**1, 2, 3a, 3b, 3c, 3d, 4, 5a, 5b, 6a, 6b, 7a, 7b, 7c, 8, and 9 (N=16)**
**II**	**P1, P2, P3, P5, P6, P7, P8, P11, P12, P13, P15, P16, P18, P20, P21, P22, Pa, Pc, Pe, Pf, Pg, Ph, Pj, Pm, and Pn (N=25)**	**1, 2, 3a, 3b, 3c, 4_Bristol, 4_NewOrleance, 4_Apeldoorn, 4_NSW001P, 4_Sydney, 5, 6a, 6b, 6c, 7, 8, 9, 10, 11, 12, 13, 14, 15, 16, 17, 18, 19, 20, and 22 (N =29)**

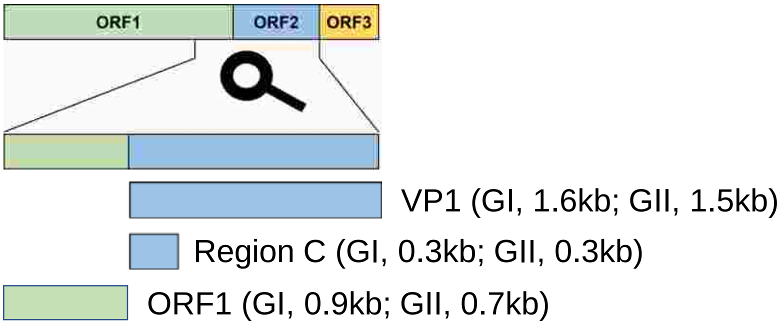

Prepared reference sequences that correspond to the coverage of the isolated sequences in this study

5' 3' Region C | 5' 3' ORF1 | 5' 3' VP1

Neighbor joining tree
(substitution model: number of difference)

- Selection of a pair of sequences that showed highest shared identity between two different genotype sequences in the tree.
- Selection of a pair of sequences that showed highest shared identity between two different sub-genotype sequences of a single genotype in the tree with the exception of ORF1 (lack of suggested sub-genotype for the ORF1).
- If the STR is not detected in subsequent RDP4 analysis, sequence pairs showing a lower degree of shared identity (as high as possible) are selected

1.1.1 Construction of neighbor joining trees and selection of GI sequence pairs showing minimum branch length in the trees

[GI_ORF1 tree]

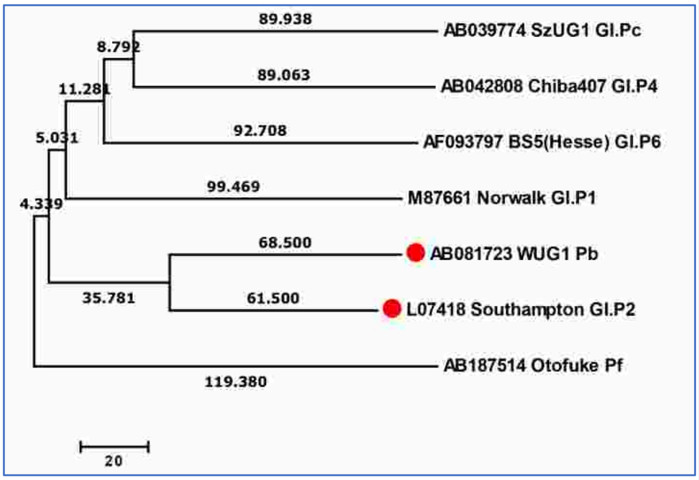

[GI_ORF2_VP1 tree]

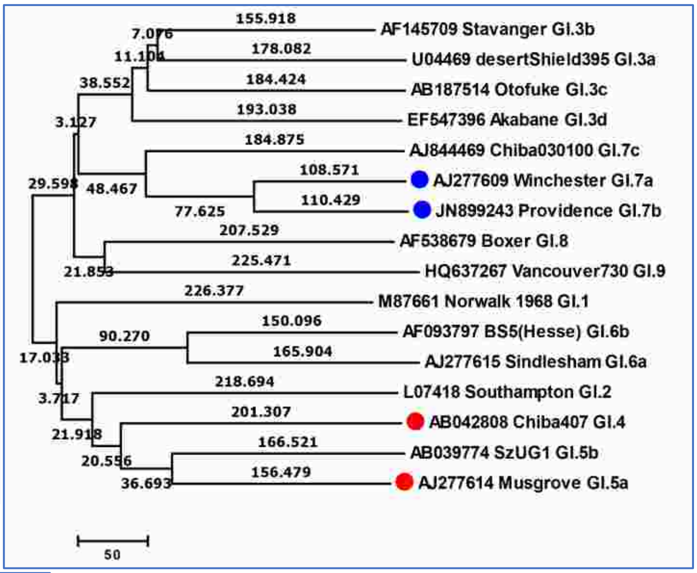

[GI_ORF2_region C tree]

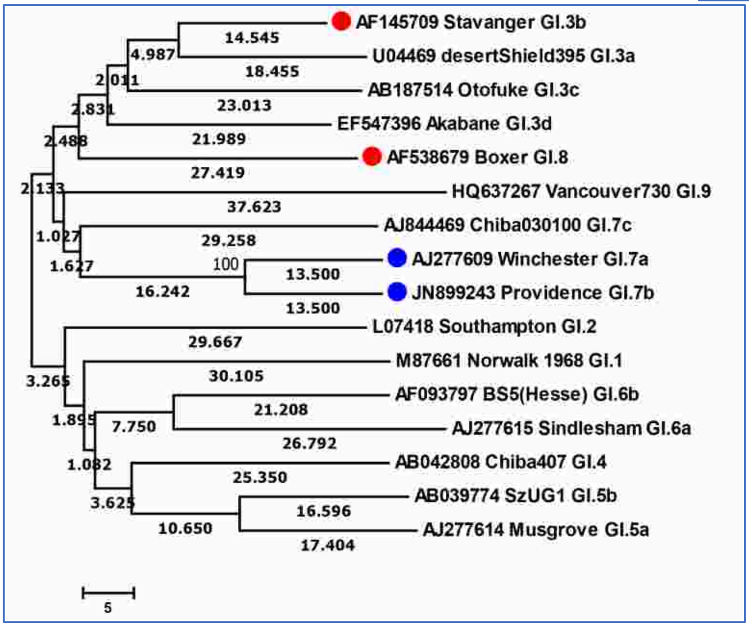

	ORF1	RegionC (ORF2)		VP1 (ORF2)	
Recombination type of pair	Inter genotypic	Inter genotypic	Intra genotypic	Inter genotypic	Intra genotypic
Selected sequence pair	AB081723 WUG1 Pb<br>L07418 Southampton GI.P2	AF145709 Stavanger GI.3b<br>AF538679 Boxer GI.8	AJ277609 Winchester GI.7a<br>JN899243 Providence GI.7b	AB042808 Chiba407 GI.4<br>AJ277614 Musgrove GI.5a	AJ277609 Winchester GI.7a<br>JN899243 Providence GI.7b
Lineage names of the sequence pair	GI.Pb and GI.P2	GI.3b and GI.8	GI.7a and GI.7b	GI.4 and GI.5a	GI.7a and GI.7b

1.1.2 Construction of neighbor joining trees and selection of GII sequence pairs showing minimum branch length in the trees

[GII_ORF1 tree]

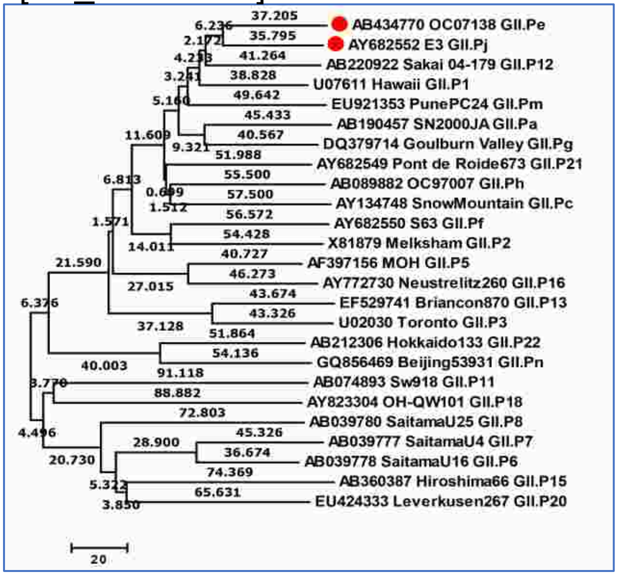

[GII_ORF2_VP1 tree]

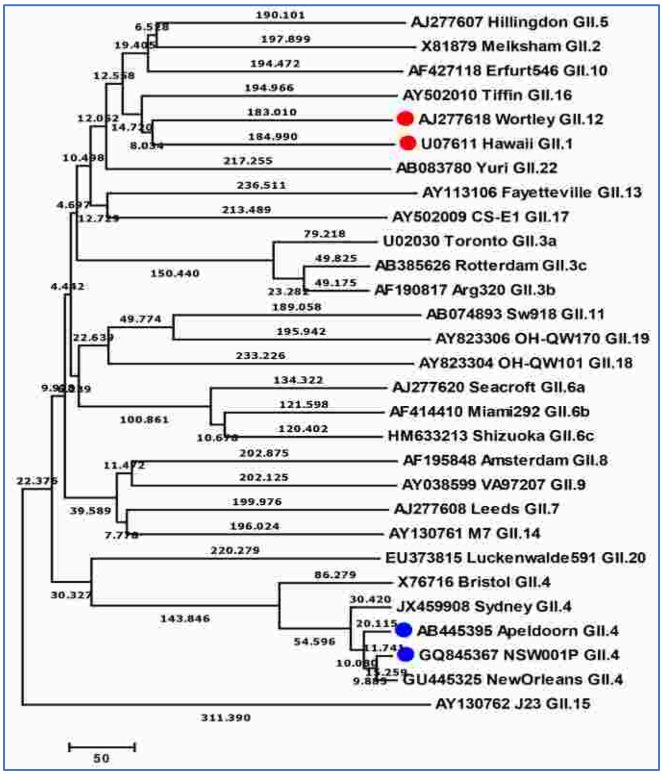

[GII_ORF2_region C tree]

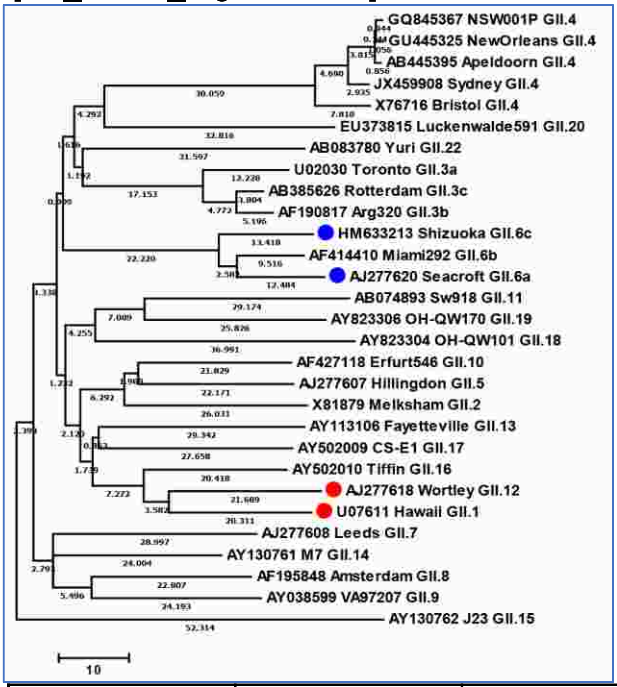

	ORF1	RegionC		VP1	
Recombination type of pair	Inter genotypic	Inter genotypic	Intra genotypic	Inter genotypic	Intra genotypic
Selected sequence pair	AB434770 OC07138 GII.Pe<br>AY682552 E3 GII.Pj	AJ277618 Wortley GII.12<br>U07611 Hawaii GII.1	HM633213 Shizuoka GII.6c<br>AJ277620 Seacroft GII.6a	AJ277618 Wortley GII.12<br>U07611 Hawaii GII.1	GQ845367 NSW001P GII.4<br>AB445395 Apeldoorn GII.4
Lineage names of the sequence pair	GII.Pe and GII.Pj	GII.12 and GII.1	GII.6c and GII.6a	GII.12 and GII.1	GII.4 NSW001P and GII.4 Apeldoorn

1.2 Construction of the standard recombinants (STRs)

1.2.1 Construction of the STRs for RDP4 analysis of ORF2

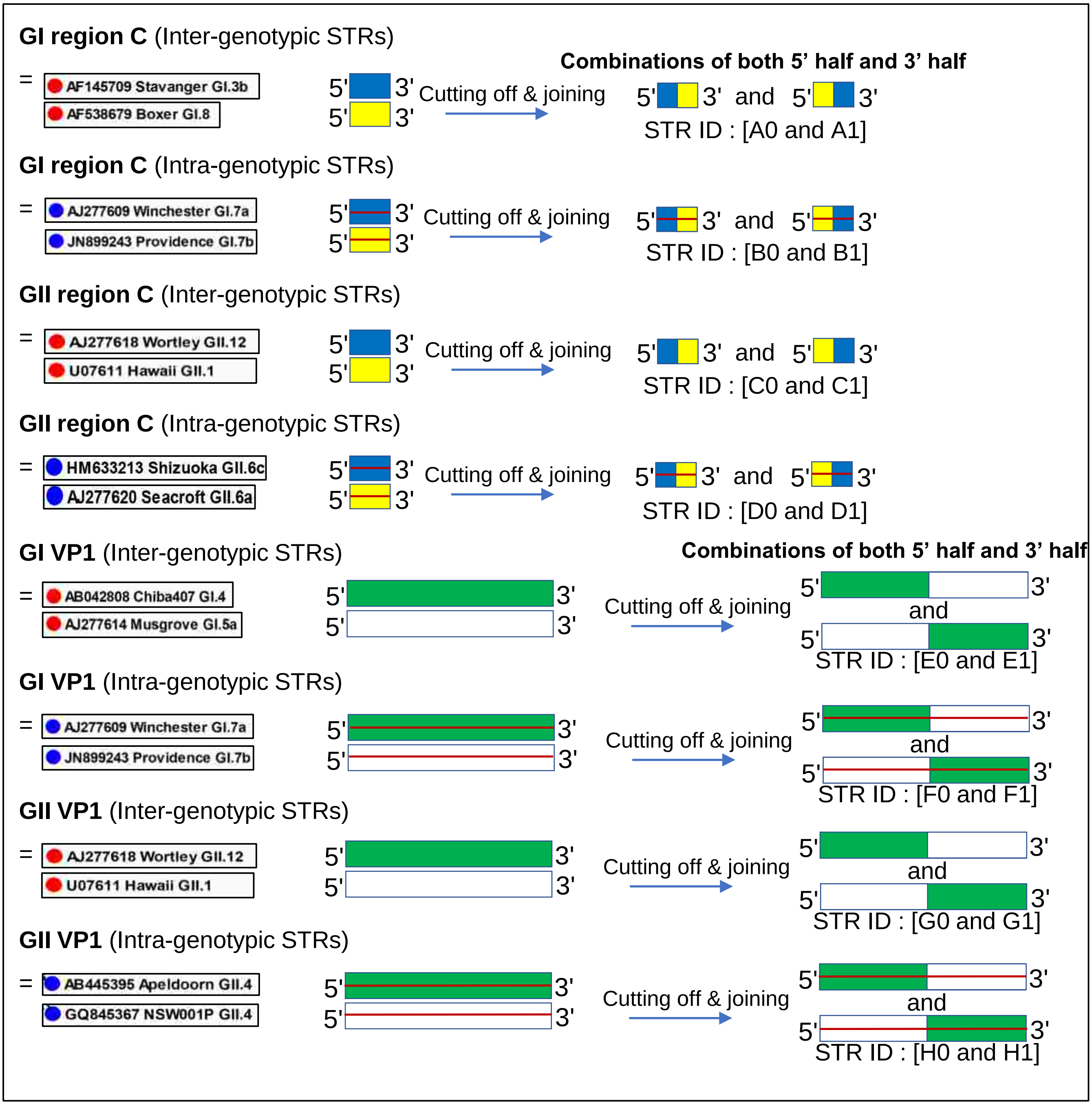

1.2.2 Construction of the STRs for RDP4 analysis of ORF1

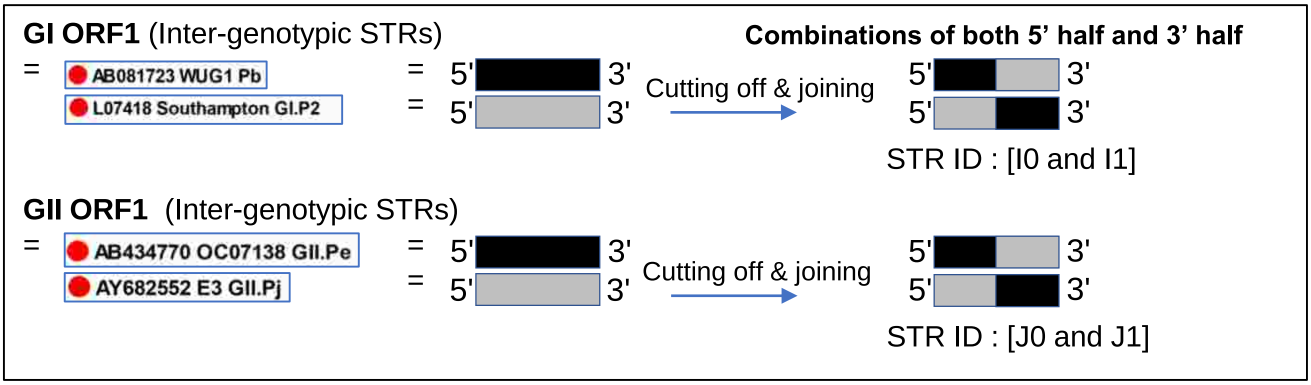

1.2.3 Construction of the STRs for RDP4 analysis of the ORF1/2 junction

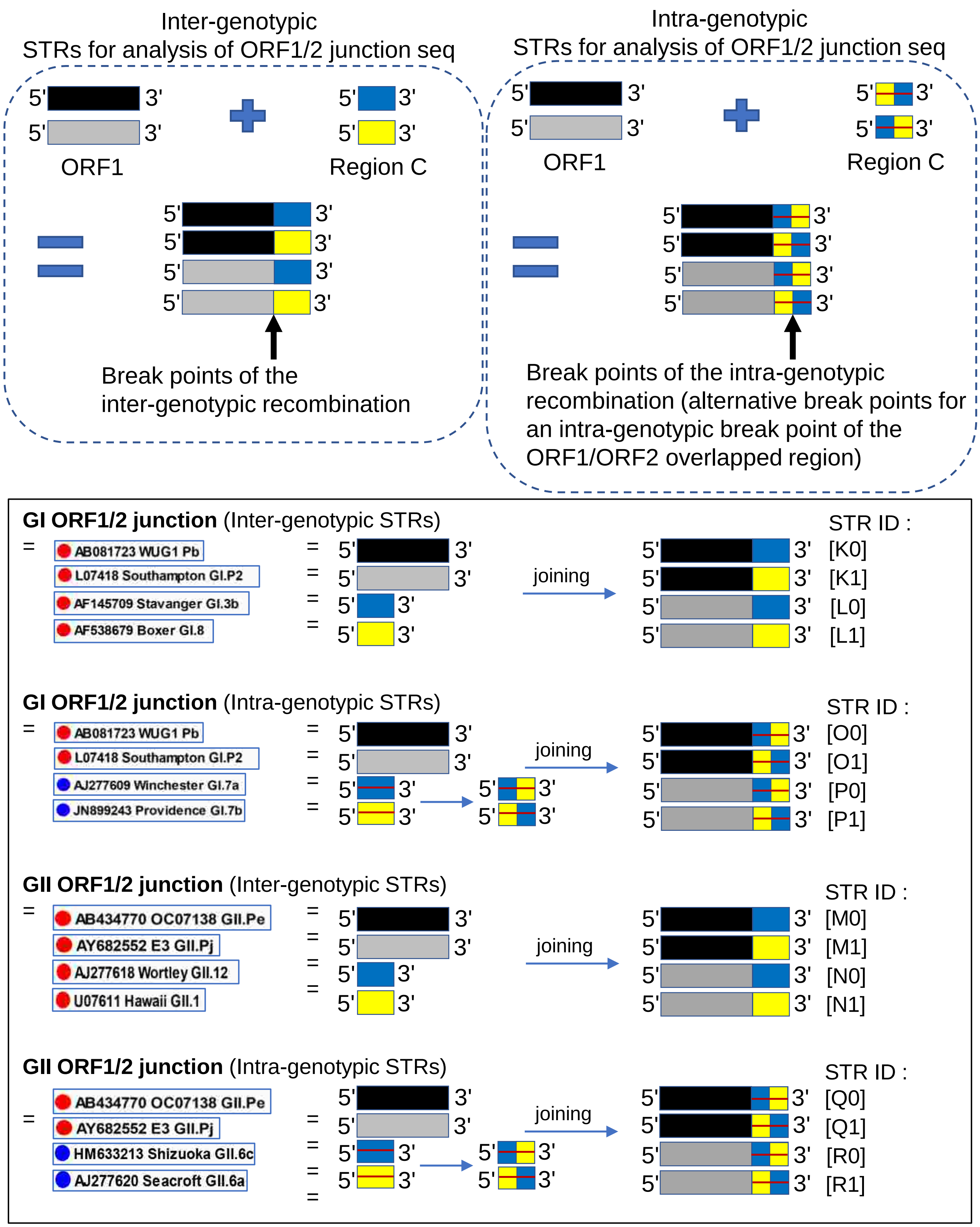

2. RDP4 analysis using the STRs

2.1 Estimation of recombination events in the isolated ORF2 sequences

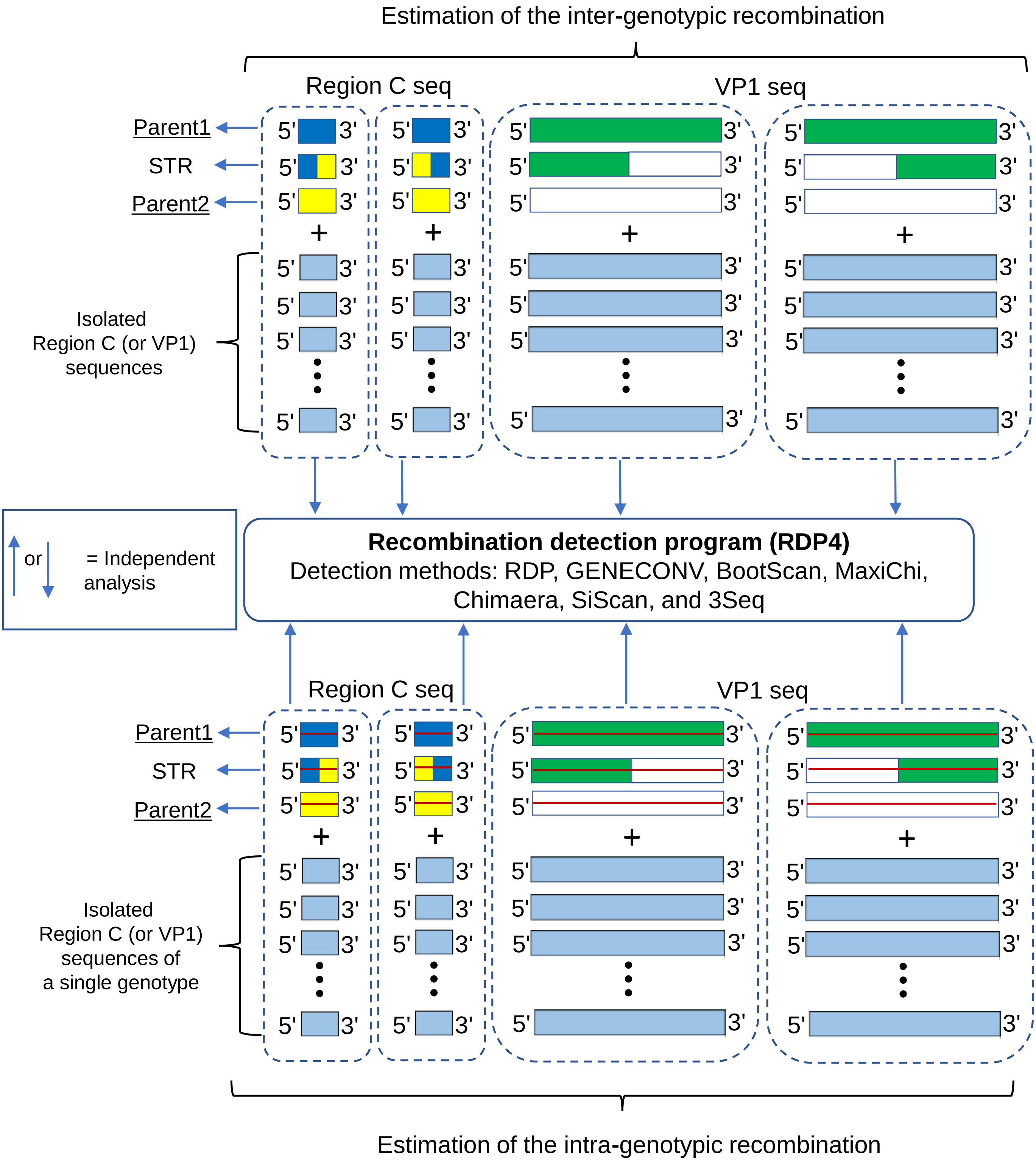

2.2 Estimation of recombination events in the isolated ORF1 sequences

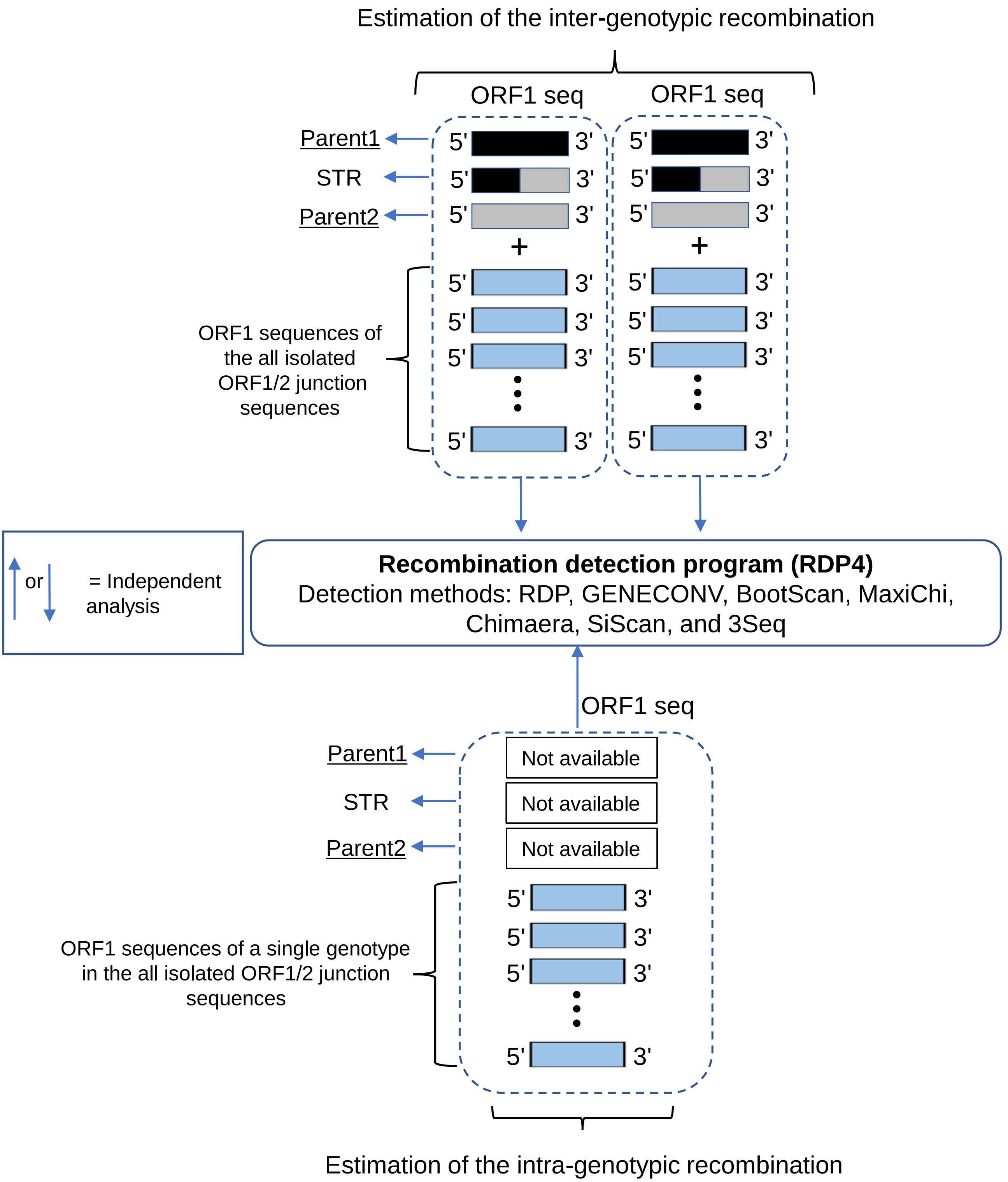

2.3 Estimation of recombination events in the isolated ORF1/2 junction sequences

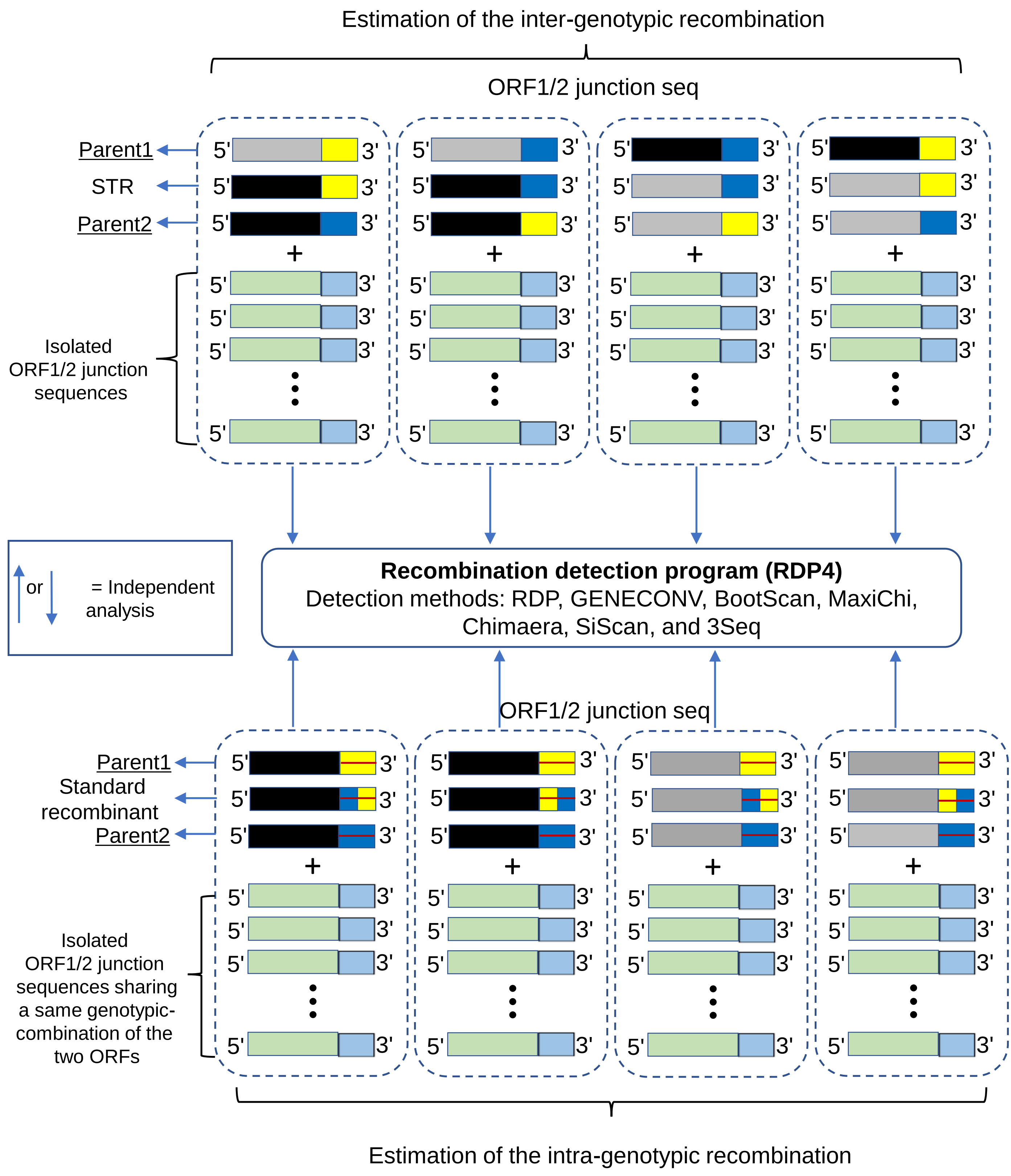

3. Hypothetical examples of detection of recombinant candidate detections satisfying the number of positive signals at the STRs

Flow chart of the hypothetical examples

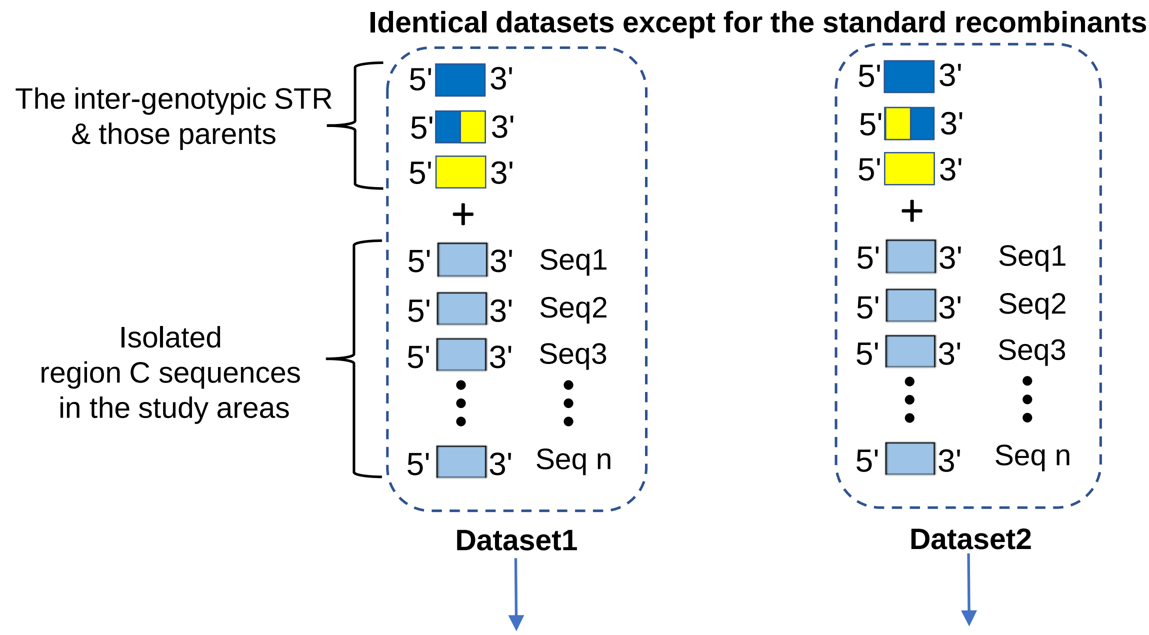

Analysis in the RDP4 using the seven methods

Dataset1:

- Amplicon type of STR : **RegionC (GI)**
- Recombination type of STR : **Inter-genotypic**
- The number of detection methods showing positive signals at STR = **Five methods**
- The number of positive signals of STR showing acceptable values (P < 0.01) among the **five methods** = **Four methods**

Possible recombinant candidates showing four or more positive methods (P < 0.01) : seq1, seq28, and seq67

Dataset2:

- Amplicon type of STR : **RegionC (GI)**
- Recombination type of STR : **Inter-genotypic**
- The number of detection methods showing positive signals at STR = **Six methods**
- The number of positive signals of STR showing acceptable values (P < 0.01) among the **six methods** = **Two methods**

Possible recombinant candidates showing two or more positive methods (P < 0.01) : seq20, seq28, seq50, seq67, and seq88

Possible inter-genotypic recombinant candidates of the GI region C sequences : seq1, Seq20, seq28, seq50, seq67, and seq88 in the whole region C sequences

Confirmation of the recombination breakpoints via neighbor joining trees (Additional filtering out of the false positives)

References (Supplemental material)

1. Chhabra P, de Graaf M, Parra GI, Chan MC, Green K, Martella V, Wang Q, White PA, Katayama K, Vennema H, Koopmans MPG, Vinjé J. 2019. Updated classification of norovirus genogroups and genotypes. J Gen Virol 100:1393-1406.
2. Vinjé J. 2015. Advances in laboratory methods for detection and typing of norovirus. J Clin Microbiol 53:373-81.
